# Meditation or exercise for preventing acute respiratory infection (MEPARI-2): A randomized controlled trial

**DOI:** 10.1371/journal.pone.0197778

**Published:** 2018-06-22

**Authors:** Bruce Barrett, Mary S. Hayney, Daniel Muller, David Rakel, Roger Brown, Aleksandra E. Zgierska, Shari Barlow, Supriya Hayer, Jodi H. Barnet, Elisa R. Torres, Christopher L. Coe

**Affiliations:** 1 University of Wisconsin Department of Family Medicine and Community Health, Madison, WI, United States of America; 2 University of Wisconsin School of Pharmacy, Madison, WI, United States of America; 3 University of Colorado Department of Medicine–Rheumatology Division, Fort Collins, CO, United States of America; 4 University of New Mexico Department of Family & Community Health, Albuquerque, NM, United States of America; 5 University of Wisconsin School of Nursing, Design & Statistics Unit, Madison, WI, United States of America; 6 University of Wisconsin Department of Population Health Sciences, Madison, WI, United States of America; 7 University of Mississippi Medical Center, School of Nursing, Madison, WI, United States of America; 8 University of Wisconsin Department of Psychology, Harlow Center for Biological Psychology, Madison, WI, United States of America; TNO, NETHERLANDS

## Abstract

**Background:**

Practice of meditation or exercise may enhance health to protect against acute infectious illness.

**Objective:**

To assess preventive effects of meditation and exercise on acute respiratory infection (ARI) illness.

**Design:**

Randomized controlled prevention trial with three parallel groups.

**Setting:**

Madison, Wisconsin, USA.

**Participants:**

Community-recruited adults who did not regularly exercise or meditate.

**Methods:**

1) 8-week behavioral training in mindfulness-based stress reduction (MBSR); 2) matched 8-week training in moderate intensity sustained exercise (EX); or 3) observational waitlist control. Training classes occurred in September and October, with weekly ARI surveillance through May. Incidence, duration, and area-under-curve ARI global severity were measured using daily reports on the WURSS-24 during ARI illness. Viruses were identified multiplex PCR. Absenteeism, health care utilization, and psychosocial health self-report assessments were also employed.

**Results:**

Of 413 participants randomized, 390 completed the trial. In the MBSR group, 74 experienced 112 ARI episodes with 1045 days of ARI illness. Among exercisers, 84 had 120 episodes totaling 1010 illness days. Eighty-two of the controls had 134 episodes with 1210 days of ARI illness. Mean global severity was 315 for MBSR (95% confidence interval 244, 386), 256 (193, 318) for EX, and 336 (268, 403) for controls. A prespecified multivariate zero-inflated regression model suggested reduced incidence for MBSR (p = 0.036) and lower global severity for EX (p = 0.042), compared to control, not quite attaining the p<0.025 prespecified cut-off for null hypothesis rejection. There were 73 ARI-related missed-work days and 22 ARI-related health care visits in the MBSR group, 82 days and 21 visits for exercisers, and 105 days and 24 visits among controls. Viruses were identified in 63 ARI episodes in the MBSR group, compared to 64 for EX and 72 for control. Statistically significant (p<0.05) improvements in general mental health, self-efficacy, mindful attention, sleep quality, perceived stress, and depressive symptoms were observed in the MBSR and/or EX groups, compared to control.

**Conclusions:**

Training in mindfulness meditation or exercise may help protect against ARI illness.

**Limitations:**

This trial was likely underpowered.

**Trial registration:**

Clinicaltrials.gov NCT01654289

## Introduction

Acute respiratory infection (ARI), including common cold, influenza, and influenza-like-illness, is very common, and leads to substantive morbidity, mortality, and economic harms. Evidence suggests that psychological, social and behavioral factors influence susceptibility to ARI illness [1–4]. For example, people with higher levels of perceived stress [5–8], emotional reactivity [9], recent stressful life events [10,11], and negative emotional styles [12,13] are at greater risk for ARI illness. Numerous observational studies and a few small randomized trials suggest that exercise helps prevent ARI illness [14–16].

Given these associations, we designed the first MEPARI trial [17] (Meditation or Exercise to Prevent Acute Respiratory Infection) to test whether training in mindfulness-based stress reduction (MBSR) or sustained moderate intensity exercise would reduce the incidence, duration, severity and impact of all-cause ARI. That 3-group n = 154 randomized trial found 33%, 43%, and 60% proportional reductions in incidence, duration and global severity of ARI illness after MBSR training, and 29%, 42%, and 31% reductions in the exercise (EX) group, respectively, compared to the controls [18–20]. There were 16 days of work lost to ARI illness among those randomly assigned to MBSR training, 32 days in the EX group, and 67 days among controls [21]. To test effects of MBSR and EX on adaptive immunity, participants received trivalent influenza vaccinations, and were then tested for antigen specific IgG (serum), IgA (nasal wash), and IL-10 and IFN-gamma from antigen-stimulated leukocytes. No between-group differences were found among those immune biomarkers [22].

The MEPARI-2 trial was designed to replicate and extend findings from the first MEPARI trial. The primary aim was to evaluate potential preventive benefits of meditation or exercise training on the incidence, duration, and severity of all-cause ARI illness, along with ARI-related absenteeism, health care utilization, and medication use. Secondary aims included evaluation of: a) potential pro-inflammatory mediators, and b) psychosocial outcomes including depressive symptoms, general mental and physical health, mindful attention, perceived stress, positive and negative emotion, self-efficacy, sleep quality, and social support.

## Methods

The MEPARI-2 trial was conducted in Madison, Wisconsin, USA, and was approved by the University of Wisconsin Institutional Review Board’s human subjects committee. Adults aged 30 to 69 years were recruited using a variety of community advertising techniques, screened by telephone interview, and then met twice in person for baseline assessment, written informed consent, and enrollment. Allocation to the 3 treatment groups was accomplished using sealed envelopes, based on randomization codes generated by an independent statistician using variable block sizes. While participants could not be blinded to interventions, investigators remained masked to group assignment until after the last participant exited.

To be eligible, prospective participants had to answer “Yes” to either “Have you had at least 2 colds in the last 12 months?” or “On average do you get at least 1 cold per year?” Prospective participants were excluded if they scored 14 or higher on the PHQ-9 depression screen [23], were currently practicing or had prior training in meditation, or if they exercised vigorously ≥ 1 time per week or moderately ≥ 2 times per week, following CDC BRFSS criteria [24]. Current or anticipated use of antibiotics, antivirals, immunoactive medications, malignancy, and autoimmune disease were also exclusionary.

Following procedures nearly identical to the first MEPARI trial [18], participants were randomized to: 1) 8-week training in mindfulness-based stress reduction (MBSR), taught by experienced instructors following standardized MBSR curricula [17]; 2) matched 8-week training in progressive moderate intensity exercise (EX); or 3) observational control. Training classes in MBSR or EX were matched in terms of location, class time (2.5 hours per week), homework practice assigned (20 to 45 minutes per day), and class size (14 to 16 people). Exercise classes were taught by experienced exercise instructors. In addition to the 8 weekly classes, a 5 hour weekend retreat was held for both EX and MBSR participants. Adequate (per protocol) participation was defined as attending at least 5 of the 9 training opportunities.

The trial was conducted from 2012 to 2016, with 4 annual cohorts. Screening occurred in the summer, with enrollment and randomization in August, followed by MBSR or EX training in September and October. Participants were followed through May of the following year using computerized weekly self-report, periodic in-person visits, and close surveillance during ARI illness. Weekly self-reports included daily minutes of MBSR or EX practice. Exercise minutes were defined as moderate or vigorous following accepted criteria [25]. Mindfulness practice minutes were categorized as formal or informal as: “Formal practice is when you schedule specific time to just do that particular activity. For example, scheduling 15 minutes to sit and focus on your breath is formal meditation practice. Taking a moment to notice your breath during your work day is informal practice. Scheduling time to take a walk for the purpose of practicing meditation is formal practice. Walking mindfully from your kitchen to the living room is informal practice.”

The primary outcome was global severity of ARI illness, defined as area-under-the-time-severity-curve. Daily self-reports on the Wisconsin Upper Respiratory Symptom Survey (WURSS-24) [26–28] assessed symptom severity and quality-of-life impact. The beginning of each ARI illness episode was defined by: A) answering “Yes” to either: “Do you think you are coming down with a cold?” or “Do you think you have a cold” and B) reporting at least one of the following symptoms: nasal discharge, nasal congestion, sneezing, or sore throat; and C) scoring at least 2 points on the Jackson scale [29]. The last time the participant reported symptoms indicating they were still ill defined the end of the illness. At least 2 days in a row had to meet criteria to be classified as an ARI illness episode. Time of self-report was recorded so that ARI duration was assessed in hours and minutes and then converted to decimalized days. Area-under-curve global severity was computed using trapezoidal approximation with WURSS-24 scores the y-axis and duration the x-axis.

Assessment included ARI-related absenteeism and health care utilization, virus identification, and inflammatory biomarker levels during ARI illness. Secondary outcomes also included several psychosocial domains assessed by validated self-report instruments at baseline, then 3 or 4 times after intervention. These assessed: general mental and physical health (SF-12) [30], perceived stress (PSS-10) [31], sleep quality (PSQI) [32], self-efficacy (MSES, ESES) [33,34], mindful awareness (MAAS) [35], positive and negative emotion (PANAS) [36], perceived social support (SPS) [37], and the sense of feeling loved (www.fammed.wisc.edu/feeling-loved; validation paper under review). Five important personality traits (BFI) [38], the social network (SNI) [39], and co-morbidities (Seattle Index) [40] were also assessed, but were not expected to be influenced by interventions. The Global Physical Activity Questionnaire (GPAQ) [41] was used to assess self-reported physical activity in all 3 groups. To minimize burden on participants, administration of instruments was staggered.

Blood and nasal wash samples were collected at baseline, 1 and 4 months after the 8-week interventions, and approximately 24–72 hours into each ARI episode. Biomarkers included: interleukin-6 (IL-6), interleukin-8 (IL-8), high sensitivity C-reactive protein (CRP), procalcitonin (PCT), and interferon-gamma-induced protein 10 (IP-10) [42]. Neutrophil counts were done on nasal wash during ARI episodes only. Procalcitonin was dropped after 96 of 99 consecutive samples had non-detectable values [43]. Blood samples were collected via standard venipuncture. Nasal wash samples were collected by nasal lavage. All samples were aliquoted and frozen at -80°C. Serum or nasal IL-6, IL-8, and IP-10 concentrations were measured using enzyme-linked immunosorbent assay (ELISA; R&D Systems, Minneapolis, MN). Glycosylated hemoglobin (HbA1c) and CRP were measured by the CLIA-certified U.W. Hospital laboratory using ion exchange chromatograph with spectrometric detection and turbidimetric assay, respectively. Blood pressure and body mass index (BMI) were assessed at baseline, and at several follow-up time points. All participants received influenza vaccine.

Participants used a nasal swab at home as soon as ARI illness criteria were confirmed, and then came to the hospital lab for nasal lavage and blood draw. Polymorphonuclear (PMN) leukocytes in the nasal wash were counted using a standard hemocytometer and expressed as neutrophils per milliliter (PMN/mL). Viruses were identified from both swabs and nasal wash, using high-throughput multiplex PCR methods developed at the University of Wisconsin [44]. A positive result from either sample was considered sufficient for viral identification.

Data are presented in the manuscript as means with standard deviations (or 95% confidence intervals) for measurements with distributions consistent with normality. For skewed measurements, medians and interquartile ranges are reported. Nonparametric statistical tests are used to compare groups: the Kruskal-Wallis test using Wilcoxon scores for continuous variables and the Pearson chi-square test for categorical variables. Between-group contrasts of ARI outcomes include differences in means (for measures consistent with normal distribution) or shifts in location (using Hodges Lehmann estimation for skewed measures). Comparing intervention to control groups, the Wilcoxon signed rank test is used to for the highly skewed cytokine data, and the Wald chi-square test in negative binomial regression is used to compare the number of ARI illnesses per person. ARI outcomes are also portrayed as proportional differences in rates (equivalent to relative risk reduction). The control group is used as reference for all between-group comparisons.

The primary efficacy analysis was done using the same zero-inflated multivariate regression model employed in the first MEPARI trial [17], which incorporates a logistic sub-model for people who do not experience ARI illness (zeroes), and a linear sub-model accounting for variability in the continuous outcome measures (global severity, duration-of-illness) [45]. Pre-specified covariates were: age, gender, body mass index, smoking status, highest level of education achieved, comorbidity, neuroticism, conscientiousness, general physical health, and general mental health. Primary comparisons are between: 1) EX and control, and 2) MBSR and control, with the level of statistical testing set *a priori* at ≤0.025 one-sided for null hypothesis rejection. Secondary analyses are considered statistically significant at alpha <0.01 for evidence-of-effect, and at <0.05 for hypothesis-generation or cautious support of effect based on two-sided tests. The target sample size of n = 396 for this phase two trial was informed by data from the first MEPARI trial [18], using alpha = 0.025 and beta = 0.80, and one-sided testing, for ARI illness primary outcomes.

SAS version 9.4 was used for data cleanup and most statistical analyses (unless otherwise specified). Stata was used for zero-inflated models and missing-at-random evaluations. When data was found to satisfy the missing-completely-at-random criteria [46], data were imputed using Stata MICE multiple imputation methods [47,48]. (Overall, there was less than 2% missing data.) Due to skewness of primary outcomes, data were transformed the log10 scale before modelling. Data cleaning, transformation, missing-at-random evaluation, and imputation were conducted by statisticians blinded to allocation group.

## Results

**Fig 1:**
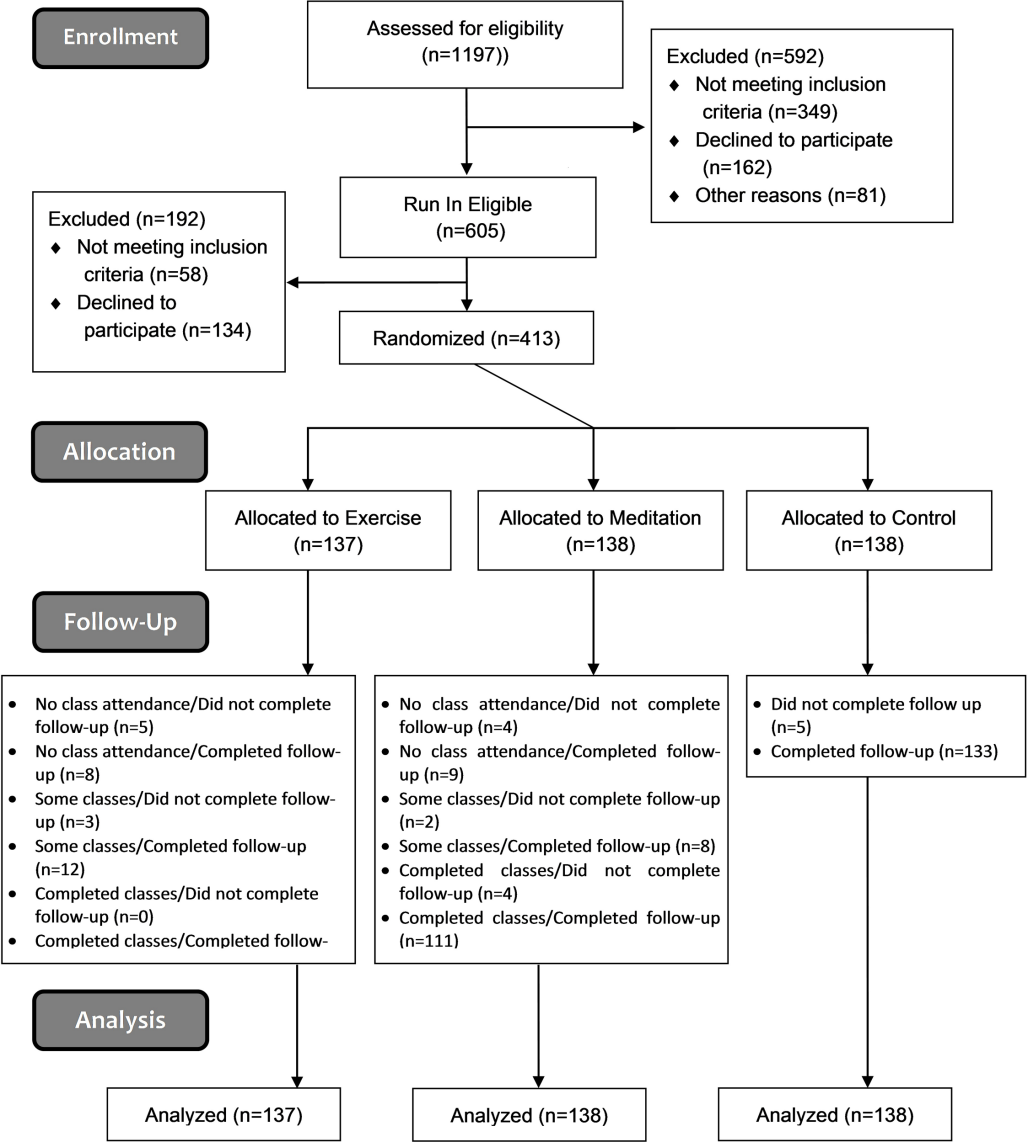
Primary intention-to-treat efficacy analyses includes all participants (n = 413).

Of 1197 persons screened, 413 were randomized, and 390 completed the trial (Fig 1). Participants tended to be female (76%), white (85%), educated (77% completed college), and middle aged (mean age 49.6 ± SD 11.6 years) (Table 1). Of 138 assigned to MBSR, 83% attended at least 5 of the 9 training opportunities. For the EX group, 80% met this predefined per protocol criterion. Weekly self-reports of day-to-day EX and MBSR practice found that 79% of exercisers and 62% of meditators achieved target rates of 150 minutes/week of practice for at least half of the 37 weeks monitored. High rates of MBSR and EX practice continued throughout 37 weeks of observation.

**Table 1:** Demographic and psychosocial characteristics of study population.

**Characteristic**	**Exercise**	**Meditation**	**Control**
Sample, N	137	138	138
Age (years), mean ± SD	49.1 ± 11.4	49.2 ± 11.2	50.7 ± 12.1
Female, n (%)	107 (78.1)	105 (76.1)	101 (73.2)
Current Smoker, n (%)	9 (6.6)	6 (4.3)	11 (8.0)
Race, n (%)			
White/Caucasian	105 (76.6)	121 (88.3)	123 (89.1)
Black/African American	14 (10.2)	5 (3.6)	6 (4.3)
Asian	8 (5.8)	5 (3.6)	3 (2.2)
Other/More Than One Race	10 (7.3)	6 (4.4)	6 (4.3)
Hispanic Ethnicity, n (%)	5 (3.8)	11 (8.1)	8 (6.0)
BMI (kg/m^2^), mean ± SD	29.3 ± 7.0	29.8 ± 7.8	29.0 ± 6.6
College Graduate or Higher, n (%)	108 (78.8)	106 (76.8)	102 (73.9)
Income > $50,000, n (%)	79 (58.1)	85 (63.4)	85 (62.5)
Systolic BP (mmHg), mean ± SD	122 ± 15	120 ± 16	124 ± 17
Diastolic BP (mmHg), mean ± SD	75 ± 9	74 ± 8	76 ± 9
Instruments, mean ± SD			
BFI: Agreeableness	37.4 ± 5.5	37.4 ± 5	37.7 ± 5.4
BFI: Conscientiousness	36.1 ± 5.6	36.3 ± 5.3	35.6 ± 5.8
BFI: Openness	40.4 ± 5.3	40.1 ± 5.4	39.2 ± 6.3
BFI: Extraversion	27.1 ± 6.9	27.4 ± 6.2	26.8 ± 5.9
BFI: Neuroticism	20.6 ± 6.3	20.3 ± 5.9	20.8 ± 5.7
SF12: Physical Health	51.4 ± 8.4	51.2 ± 8	51.4 ± 8.3
SF12: Mental Health	47.9 ± 10.4	48.0 ± 10	47.6 ± 9.9
SPS Social Support	83.2 ± 9.8	83.5 ± 10.1	83.3 ± 9.3
SNI: Network Diversity	6.3 ± 2	6.3 ± 1.8	6.3 ± 1.8
SNI: Potential Contacts	24.2 ± 9.8	23.6 ± 8.9	23.6 ± 8.1
SNI: Number of Roles	7.3 ± 1.9	7.5 ± 1.8	7.2 ± 1.8
PANAS Positive	35.4 ± 6.7	35.1 ± 7	33.9 ± 7.5
PANAS Negative	18.6 ± 6.8	18.2 ± 6.2	18.6 ± 6.7
PSS10 Perceived Stress	13.3 ± 6.6	13.1 ± 6.4	12.4 ± 5.9
PHQ9 Depressive Symptoms	2.9 ± 2.9	2.4 ± 2.4	2.9 ± 3.1
PSQI Sleep Quality	6.2 ± 3.6	5.8 ± 3.3	5.7 ± 3.3
MAAS Mindful Attention	4.3 ± 0.8	4.1 ± 0.8	4.3 ± 0.7
MSES Mindful Self-Efficacy	97.3 ± 14.7	97.6 ± 15.7	96.8 ± 14.6
ESES Exercise Self-Efficacy	112.4 ± 38.6	112.5 ± 38.3	116.0 ± 38.8
Stanford Presenteeism	20.3 ± 5.3	20.4 ± 5.4	20.1 ± 5.2
Instruments, median (IQR)			
Feeling Loved Score	365 (340–385)	369 (333–389)	370 (340–389)
GPAQ (MET-hrs/wk)	560 (160–1320)	730 (240–1940)	1020 (320–2400)
SIC Comorbidity Score	2.0 (1.0–4.0)	2.0 (1.0–4.0)	3.0 (1.0–4.0)
Biomarkers, median (IQR)			
IL-6 (serum) (pg/mL)	1.8 (1.1–2.9)	1.6 (1.0–2.8)	1.7 (1.0–2.9)
IL-6 (nasal) (pg/mL)	1.0 (0.6–2.1)	1.3 (0.8–2.2)	1.1 (0.5–2.3)
IL-8 (pg/mL)	157 (79–271)	167 (86–313)	194 (87–352)
IP-10 (pg/mL)	156 (126–198)	141 (117–183)	152 (127–198)
hsCRP (pg/mL)	1.4 (0.6–3.9)	1.6 (0.7–4.5)	1.5 (0.7–4.8)
HbA1c (pg/mL)	5.6 (5.3–5.8)	5.5 (5.2–5.7)	5.6 (5.3–5.8)

Abbreviations: SD = standard deviation, IQR = interquartile range, BP = blood pressure, BFI = big five inventory, SF12 = medical outcomes study short form, SPS = social provisions scale, SNI = social network index, PANAS = positive and negative affect schedule, PSS = perceived stress scale, PSQI = Pittsburg sleep quality index, MAAS = mindfulness attention awareness scale, MSES = mindfulness self-efficacy scale, ESES = exercise self-efficacy scale, GPAQ = global physical activity questionnaire, SIC = Seattle index of comorbidity, HbA1c = hemoglobin A1c, hsCRP = high sensitivity C-reactive protein, IL = interleukin, IP = interferon gamma-induced protein.

In the MBSR group, there were 112 ARI episodes and 1045 days of ARI illness, compared to 120 episodes and 1010 illness days in the EX group, and 134 episodes with 1210 days of ARI illness for controls (Table 2; Table 2 is based on full n = 413 data set.). Mean global severity was 256 for MBSR (95% confidence interval 176, 335), 224 (161, 287) for EX, and 326 (240, 412) for control. The mean number of ARI episodes per person randomized was 0.81 (0.66, 0.97) for MBSR, 0.88 (0.72, 1.03) for EX, and 0.97 (0.80, 1.15) for control. The mean number of ARI days (duration) per person was 7.6 (5.6, 9.6) for MBSR, 7.4 (5.7, 9.1) for EX, and 8.8 (6.8, 10.7) for control. For the 58% of participants who did experience an ARI, the mean duration and global severity were 7.1 days and 315 severity points for MBSR, 7.0 and 256 for EX, and 7.1 and 336 for control, respectively. The prespecified zero-inflated multivariate regression model suggested reduced incidence for MBSR (p = 0.036) and lower global severity for EX (p = 0.042), compared to controls. Model parameters and coefficients are available in S1 File.

**Table 2:** Main ARI-related outcomes.

Outcome	Exercise (n = 137)	Meditation (n = 138)	Control (n = 138)	Control vs Exercise^1^^,^^2^	Control vs Meditation^1^^,^^2^
**Total # ARIs**	120	112	134		
**# ARIs per person, mean (95% CI) **	0.88 (0.72,1.03)	0.81 (0.66, 0.97)	0.97 (0.80,1.15)	0.10 (-0.14, 0.33) p = 0.42	0.16 (-0.07, 0.39) p = 0.17
**Incidence rate (# ARIs per person-year), (95% CI)**	1.2 (0.3, 6.0)	1.1 (0.2, 5.8)	1.3 (0.3, 6.0)	0.08 (-0.16, 0.31)	0.15 (-0.08, 0.38)
**# People with at least one ARI, n (%)**	84 (61%)	74 (54%)	82 (59%)	-0.03 (-0.22, 0.16) p = 0.75	0.10 (-0.10, 0.29) p = 0.33
**Total days of ARI Illness**	1010	1045	1210		
**# days of ARI illness, mean (95% CI)**	7.4 (5.7, 9.1)	7.6 (5.6, 9.6)	8.8 (6.8, 10.7)	1.4 (-1.2, 4.0) p = 0.33	1.2 (-1.6, 4.0) p = 0.65
**ARI global severity, mean (95% CI)**	224 (161, 287)	256 (176, 335)	326 (240, 412)	102 (-4, 208) p = 0.21	70 (-46, 186) p = 0.22
**ARI-related missed work days (total)**	81	73	105		
**ARI-related missed work (hours per person) mean (95% CI)**	3.8 (1.9, 5.7)	4.0 (2.4, 5.6)	5.0 (2.9,7.1)	1.2 (-1.6, 4.0) p = 0.13	1.0 (-1.6, 3.6) p = 0.48
**Total # of medical visits**	21	22	24		
**# Visits to primary care clinic**	11	9	19		
**# Visits to specialty care clinic**	1	4	2		
**# Visits to urgent care**	4	9	2		
**# Visits to hospital/emergency department**	4	0	1		
**# Visits to other**	0	0	0		
**Total # of medications used**	266	300	381		
**Medication use (per ARI), mean (95% CI)**					
**Mean # prescription medications**	0.1 (0.0, 0.1)	0.2 (0.1, 0.2)	0.2 (0.1, 0.3)	0.12 (0.01, 0.23) p = 0.10	0.04 (-0.09, 0.17) p = 0.53
**Mean # OTC medications**	2.1 (1.8, 2.5)	2.6 (2.2, 3.0)	2.7 (2.4, 3.0)	0.6 (0.1, 1.0) p = 0.002	0.1 (-0.4, 0.6) p = 0.38
**Mean # all medications**	2.2 (1.8, 2.6)	2.8 (2.3, 3.2)	2.9 (2.6, 3.2)	0.7 (0.2, 1.2) p = 0.001	0.1 (-0.4, 0.7) p = 0.37
**ARI economic cost^3^ (per person) ($), mean (95% CI)**	$119 ($62, $175)	$140 ($83, $197)	$163 ($95, $231)	$44 ($-48, $136) p = 0.19	$23 ($-72, $118) p = 0.18
**Biomarkers^4^ (per ARI episode), median (IQR)**	91	90	119		
**hsCRP (pg/mL)**	4.2 (1.5, 9.1)	5.7 (1.4, 12.1)	4.2 (1.2, 12.8)	-0.1 (-1.2, 1.0)^6^ p = 0.475	-0.1 (-1.6, 0.6)^6^ p = 0.145
**Neutrophils (pg/mL)**	6.0 (1.0, 26.0)	4.0 (1.0, 25.0)	8.0 (1.0, 38.0)	0.0 (-2.0, 2.0)^5^	1.0 (0.0, 4.0)^5^
**IL-6 (serum) (pg/mL)**	2.8 (1.9, 4.7)	3.5 (1.8, 5.6)	2.7 (1.5, 4.7)	-0.2 (-0.7, 0.4) ^6^ p = 0.545	-0.5 (-1.2, 0.1)^6^ p = 0.275
**IL-6 (nasal) (pg/mL)**	25 (5, 91)	29 (6, 81)	16 (4, 67)	-3.7 (-13.7, 0.9)^6^ p = 0.135	-3.0 (-14.4, 1.1)^6^ p = 0.185
**IL-8 (pg/mL)**	728 (319, 1450)	472 (231, 886)	551 (296, 1342)	-95 (-253, 52)^6^ p = 0.035	104 (-9, 229)^6^ p = 0.565
**IP-10 (pg/mL)**	257 (173, 391)	298 (171, 444)	232 (164, 350)	-25 (-63, 11)^6^ p = 0.025	-40 (-89, -1)^6^ p = 0.015
**Virus identification: # ARIs4, n (%)**	91 (76%)	88 (79%)	118 (88%)		
**# ARIs found to have any virus present, n (%)**	64 (70%)	63 (72%)	72 (61%)	-0.09 (-0.21, 0.04) p = 0.16^1^	-0.11 (-0.23, 0.03) p = 0.12^1^
**Specific Virus, n (%)^7^**	68	65	76		
**Adenovirus (AV)/Bocavirus (BoV)**	1 (1.1%)	1 (1.4%)	2 (1.7%)		
**Coronavirus (CoV)**	12 (13%)	15 (17%)	16 (14%)		
**Enterovirus (EV)/Rhinovirus (HRV)**	43 (47%)	28 (32%)	41 (35%)		
**Influenza**	1 (1%)	10 (11.4%)	9 (7.6%)		
**MPV/RSV/PIV**	11 (12.1%)	11(12.5%)	8 (6.8%)		

IQR = interquartile range, CI = confidence interval, AUC = area under the curve, ARI = acute respiratory infection, hsCRP = high sensitivity C-reactive protein, IL = interleukin, IP = interferon gamma-induced protein, OTC = over the counter, MPV = human metapneumovirus, RSV = respiratory syncytial virus, PIV = human parainfluenza virus.

^1^ Between-group comparison p-values comes from: a) Wald Chi-Square test in negative binomial regression for # of ARIs per person; b) Kruskal-Wallis test comparing nonparametric Wilcoxon scores for continuous outcomes (ARI duration, global severity); and c) Wald Chi-square test in logistic regression (for any ARI and any virus).

^2^ Between group differences are computed as control group minus treatment group with 95% confidence intervals.

^3^ Economic costs were calculated by summing the estimated salary lost from missing work, cost of medical provider visits, and cost of medications used.

^4^ Lab visits were within first 72 hours of ARI. Reasons for missing data include: lab closed on weekends, participant unable to get in, etc.

^5^ Comparisons are for between group change from baseline (reference group: Control). Neutrophils were not collected at baseline, so no change from baseline could be computed. See Table 1 for baseline biomarkers values.

^6^ Hodges-Lehmann estimation for shift in location, with 95% CIs.

^7^ More than one virus was identified in 10 separate ARIs. In the EX group, one ARI yielded both BoV and CoV, two ARIs had CoV and HRV, and one ARI had EV/HRV and PIV. In the MBSR group, one ARI yielded EV/HRV and CoV, and one ARI had EV/HRV and PIV (parainfluenza virus). In the control group, one ARI yielded HRV and BoV, one ARI had MPV (metapneumovirus) and CoV, one ARI had RSV (respiratory syncytial virus) and HRV, and one ARI yielded CoV and BoV.

There were 73 ARI-related days-of-missed-work and 22 ARI-related health care visits in the MBSR group, 81 days and 21 visits for EX, and 105 days and 24 visits for control. Mean ARI-related economic costs (including the cost of absenteeism) were $140 ($83, $197) for MBSR, $119 ($62, $175) for EX, and $163 ($95, $231) for control. Trends towards reduced absenteeism and ARI-related costs for both EX and MBSR were not statistically significant. On average, controls used 2.9 (2.6, 3.2) medications per ARI episode, similar to 2.8 (2.3, 3.2) medications for those in the MBSR condition. Exercisers used fewer medications than controls during ARI episodes (2.2 vs. 2.9; p = 0.001). Total ARI-related economic costs were slightly lower in both EX and MBSR groups compared to control.

Scores on the self-report questionnaires suggested improvements in mental health for both intervention groups, as shown in Table 3 (Table 3 includes all available data for time points represented;baseline values are in Table 1). General mental health (SF12) was significantly (p<0.05) improved in the MBSR group at 3 of 4 time points and trended towards improvement at all time points for both EX and MBSR groups. Significant reductions in perceived stress (PSS10) were seen at 3 of 4 time points in both EX and MBSR groups. Sleep quality (PSQI) was significantly improved at all time points for EX and trended towards improvement at all time points for MBSR. Self-efficacy (MSES, ESES) was significantly improved in both EX and MBSR at 5 of 6 time points for MBSR, and all time points for EX. Depressive symptoms (PHQ9) were significantly lower in the EX group at 2 of 3 time points, and trended towards benefit for MBSR. Mindful attention (MAAS) was significantly improved in both MBSR and EX groups, compared to control, at all 4 follow-up time points, from November to April. As expected, self-reported physical activity (GPAQ) was much higher in the EX group than in either MBSR or control. Positive and negative emotion (PANAS), perceived social support (SPS), and the sense of feeling loved were not significantly influenced by either MBSR or EX.

**Table 3:** Secondary outcomes by follow-up visits.

	Exercise				Meditation				Control			
	November/December	January	February/March	April/Exit^1^	November/December	January	February/March	April/Exit^1^	November/December	January	February/March	April/Exit^1^
**Self-Reported Instruments** (between-group comparison of change from baseline)												
**MAAS Mindful Attention**	4.4**	4.4**	4.5**	4.5**	4.3**	4.4**	4.4*	4.4**	4.2	4.2	4.3	4.3
	(2.6, 5.8)	(2.6, 5.8)	(2.7, 6.0)	(2.6, 6.0)	(2.9, 5.6)	(2.8, 5.6)	(2.8, 5.9)	(2.8, 5.9)	(2.7, 5.7)	(2.6, 5.8)	(2.7, 6.0)	(2.7, 5.9)
**SF12: Physical Health**	51.7	51.7	51.6	51.9	51.2	51.6	51.4	50.5	52.0	51.6	50.8	51.5
	(34.8, 69.2)	(33.5, 69.8)	(31.4, 70.2)	(32.2, 70.9)	(34.8, 69.3)	(35.7, 67.5)	(35.3, 66.4)	(31.4, 71.7)	(36.0, 68.1)	(34.1, 69.2)	(33.2, 68.4)	(33.9, 69.2)
**SF12: Mental Health**	47.6	47.7	47.6	47.8	48.0	49.2*	49.3*	49.7**	45.5	45.5	46.2	45.9
	(23.5, 67.5)	(24.5, 66.6)	(24.8, 67.7)	(26.2, 65.6)	(27.7, 63.3)	(29.0, 62.1)	(28.0, 64.5)	(25.2, 66.5)	(24.5, 66.4)	(24.8, 66.2)	(25.6, 66.9)	(25.1, 66.6)
**SPS Social Support**	82.4	82.1	83.8	83.1	82.5	82.8	83.6	83.8	81.4	81.6	82.2	82.6
	(60.8, 101.9)	(61.7, 101.4)	(61.2, 103.2)	(62.2, 103.1)	(60.1, 102.6)	(60.4, 102.7)	(60.8, 103.7)	(61.5, 103.7)	(59.3, 103.5)	(61.6, 101.5)	(60.9, 103.5)	(62.6, 102.7)
**PSS10 Perceived Stress**	13.0**	12.3**	12.4**	13.3	13.0*	12.4*	12.3**	11.6**	14.0	13.8	13.6	13.4
	(0.6, 27.5)	(0.2, 27.3)	(0.5, 26.8)	(-0.5, 27.4)	(2.5, 25.5)	(2.6, 24.9)	(1.3, 25.9)	(0.8, 26.0)	(1.6, 26.5)	(1.0, 26.5)	(1.1, 26.2)	(0.6, 26.2)
**PSQI Sleep Quality**	5.4**	5.5*	5.4*	5.3**	5.5	4.9	5.2	5.1	6.0	5.8	5.6	5.8
	(-0.2, 12.2)	(-0.5, 12.0)	(-0.9, 12.2)	(-0.4, 12.0)	(-0.3, 12.4)	(-0.9, 12.4)	(-1.2, 12.5)	(-0.1,11.8)	(-0.6, 12.6)	(-0.7, 12.2)	(-0.7, 12.0)	(-1.3, 13.0)
**GPAQ Exercise**^2^	1560**	1380**	1210**	1440**	700	480	680	980	670	540	600	1080
	(860, 2770)	(600, 2320)	(600, 2210)	(640, 2940)	(120, 1692)	(0, 1260)	(180, 1560)	(240, 2460)	(160, 1560)	(40, 1560)	(4, 1740)	(360, 2740)
**MSES Mindful** **Self-Efficacy**	99.0**		99.0*	99.9**	100.7**		101.4**	102.6**	94.8		94.8	95.1
	(63.3, 126.4)		(63.8, 125.8)	(66.6, 123.6)	(65.3, 124.3)		(65.4, 124.1)	(63.8, 126.5)	(64.5, 125.1)		(62.0, 127.5)	(63.0, 127.3)
**ESES Exercise** **Self-Efficacy**	104.6**		104.3**	104.4**	92.7		96.3*	94.3*	86.0		84.3	84.6
	(13.8, 158.2)		(11.2, 157.4)	(13.4, 155.8)	(14.8, 157.3)		(12.6, 156.0)	(8.9, 160.2)	(17.8, 154.3)		(11.2, 157.4)	(13.3, 155.9)
**PANAS Positive**	34.8		34.5	34.9	35.1		34.8	35.8	33.6		32.7	33.2
	(17.9, 49.3)		(17.5, 47.9)	(17.6, 48.7)	(18.8, 48.4)		(20.3, 45.1)	(20.1, 46.2)	(18.6, 48.5)		(18.1, 47.3)	(18.2, 48.1)
**PANAS Negative**	17.9		17.7	17.9	17.7		17.5	17.4	18.7		19.0	17.9
	(5.8, 31.6)		(6.4, 31.6)	(5.6, 30.3)	(8.2, 29.3)		(7.9, 30.1)	(7.1, 28.8)	(7.5, 30.0)		(6.5, 31.4)	(7.2, 28.7)
**PHQ9 Depressive Symptoms**	4.3*		4.1*	4.1	4.4		3.9	3.6	5.3		4.9	4.5
	(-2.2, 12.8)		(-2.8, 12.5)	(-2.2, 11.2)	(-2.2, 12.8)		(-2.7, 12.4)	(-2.9, 12.0)	(-3.2, 13.7)		(-3.2, 12.9)	(-3.2, 12.2)
**Stanford Presenteeism**	24.1		24.3	24.2	23.8		23.8	24.0	23.1		23.0	23.6
	(13.2, 32.9)		(13.9, 32.2)	(14.0, 33.3)	(13.5, 32.6)		(14.6, 31.4)	(14.4, 32.9)	(13.7, 32.4)		(13.8, 32.2)	(15.2, 32.1)
**Feeling Loved**^2^	366.5		363.0	370.0	377.0		372.5	380.0	365.0		362.0	361.0
	(338.5, 389.5)		(339.0, 387.0)	(342.0, 388.0)	(342.0, 390.0)		(345.0, 390.0)	(350.0, 390.0)	(330.0, 381.0)		(340.0, 385.0)	(340.0, 385.0)
**Body Measurements** (between-group comparison of change from baseline)												
**BMI** **(kg/m**^**2**^ **)**	29.3*		29.3	29.2	29.7		29.7		29.2		28.9	29.1
	(15.1, 43.3)		(14.6, 43.3)	(14.6, 43.6)	(14.0, 44.4)		(14.0, 44.4)		(16.1, 42.3)		(16.3, 41.6)	(15.9, 42.3)
**Systolic BP**^3^ **(mmHg)**	124 (99, 157) /122 (95, 157)		120	121	122		122		126		124	125
			(97, 152)	(95, 154)	(93, 159)		(93, 159)		(98, 154)		(93, 156)	(93, 157)
**Diastolic BP**^3^ **(mmHg)**	77 (61, 96) / 75 (58, 95)		76	76	75		75		77		77	77
			(61, 93)	(62, 93)	(58, 95)		(58, 95)		(60, 93)		(61, 92)	(61, 93)
**Biomarkers**^2^ (within-group comparison of change from baseline)												
**IL-6 (serum)** **(pg/mL)**^2^	1.7		1.6		1.6		1.7		1.5		1.	
	(1.0, 3.0)		(0.9, 2.5)		(0.9, 3.2)		(0.9, 2.6)		(1.0, 2.6)		(1.1, 3.0)	
**IL-6 (nasal)** **(pg/mL)**^2^	1.8		1.6#		1.7##		1.6#		1.3##		1.6##	
	(0.8, 2.9)		(0.8, 2.8)		(0.8, 3.9)		(0.9, 2.6)		(0.6, 2.8)		(0.8, 3.6)	
**IL-8** **(pg/mL)**^2^	214##		227#		238##		258##		255##		248##	
	(127, 354)		(158, 414)		(144, 356)		(157, 424)		(140, 446)		(155, 390)	
**IP-10** **(pg/mL)**^2^	139##		145#		140		149		148		147	
	(116, 172)		(111, 190)		(116, 183)		(114, 192)		(121, 205)		(123, 192)	
**hsCRP** **(pg/mL)**^2^	1.7		1.3		1.7		1.3##		1.6		1.4	
	(0.7, 3.3)		(0.6, 3.2)		(0.8, 4.8)		(0.7, 3.3)		(0.7, 4.4)		(0.6, 3.9)	
**HbA1c** **(pg/mL)**^2^	5.5		5.5		5.6##		5.5		5.6##		5.5	
	(5.3, 5.8)		(5.3, 5.9)		(5.3, 5.8)		(5.2, 5.8)		(5.4, 5.9)		(5.3, 5.8)	

Mean (95% confidence interval) unless otherwise noted.

See Table 1 for baseline values.

Abbreviations: SPS = social provisions scale, PANAS = positive and negative affect schedule, PSS = perceived stress scale, PSQI = Pittsburg sleep quality index, MAAS = mindfulness attention awareness scale, MSES = mindfulness self-efficacy scale, ESES = exercise self-efficacy scale, GPAQ = global physical activity questionnaire, BP = blood pressure, HbA1c = hemoglobin A1c, hsCRP = high sensitivity C-reactive protein, IL = interleukin, IP = interferon gamma-induced protein.

^1^Exit visits occurred late May or early June.

^2^Median (interquartile range).

^3^ Blood pressures were collected in November at the flu shot visit and at the December in-clinic follow-up visit. All other measures were collected at only one month out of the two months listed.

Between group change from baseline comparison for that visit (reference group: Control group change from baseline):

* < 0.05,

** < 0.01 (nonparametric Wilcoxon scores in the Kruskal-Wallis test).

Biomarkers Only: Within treatment group change from baseline comparison for that visit (reference group: baseline for that treatment group):

# < 0.05,

## < 0.01 (nonparametric Wilcoxon Signed Rank Test.

Inflammatory biomarkers from nasal wash and blood collected during ARI illness are shown in Table 2. Biomarker levels at the two standardized follow-up visits in December and March when participants were not ill are shown in Table 3 (baseline values in Table 1). While all biomarkers did increase as expected during ARIs, statistically significant between-group differences were only observed for IP-10, where both MBSR (p = 0.01) and EX (p = 0.02) seemed to enhance IP-10 response to ARI, and in IL-8, where EX may have increased IL-8 response (p = 0.03) (Table 2). Biomarker levels at standardized non-ill time points were not significantly different when comparing between treatment groups (Table 3). There were, however, a few within-group-over-time comparisons that were statistically significant. For example, median CRP for MBSR decreased from 1.6 mg/L at baseline to 1.3 in March (p = 0.002); similar changes did not occur in the EX or control groups. A subgroup analysis of the 177 people with CRP ≥2.0 at baseline found that 38.3% in the MBSR group experienced an ARI, compared to 64.4% of control participants (p = 0.004). One other potentially relevant observation was that in the EX group, median IP-10 decreased from 156 pg/mL at baseline to 146 in March (p = 0.017); corresponding reductions were not seen in either the MBSR or control group.

**Fig 2:**
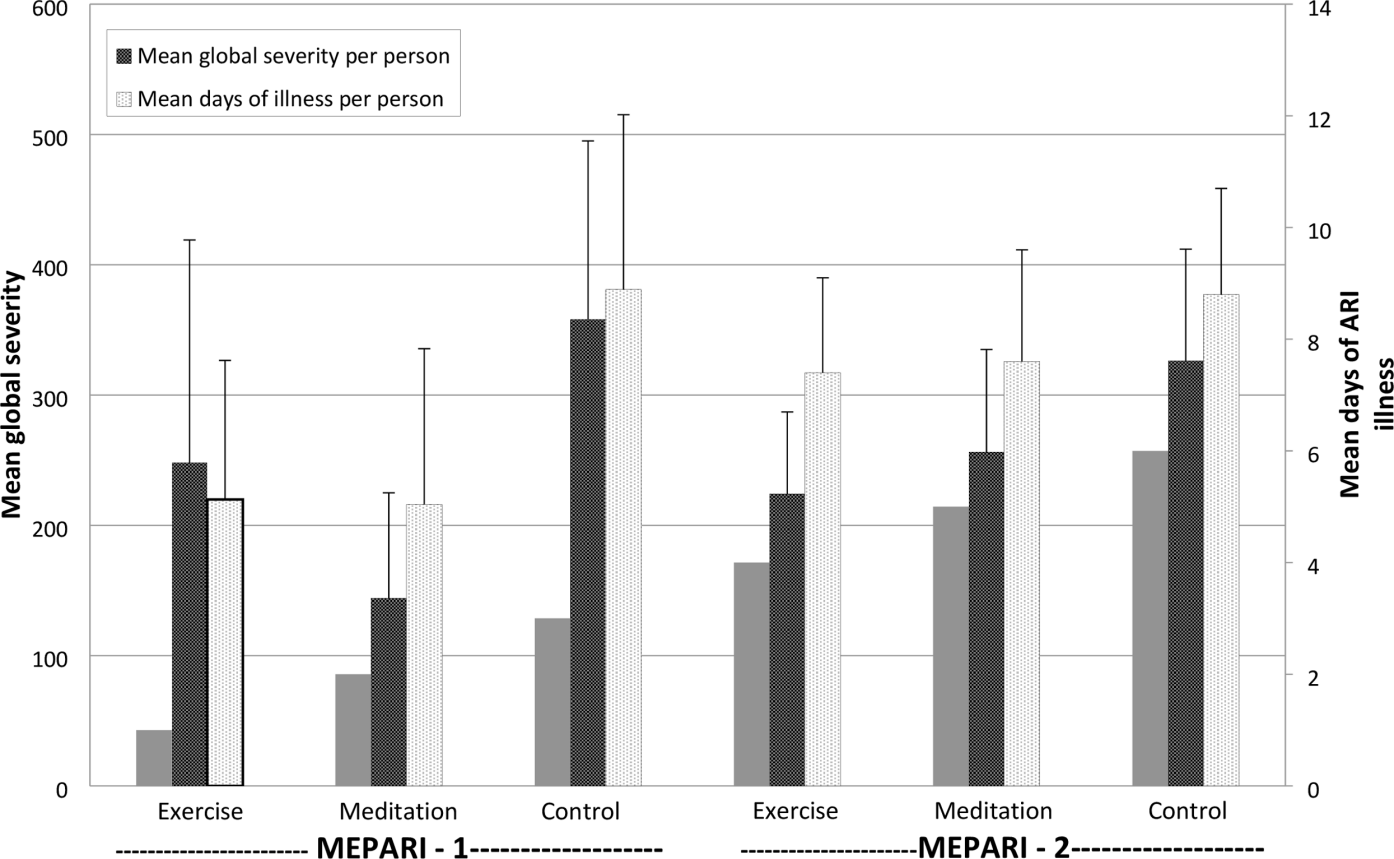
ARI = acute respiratory infection; Global severity = Area under time severity curve, calculated as trapezoidal approximation using daily scores on Wisconsin Upper Respiratory Infection Symptom Severity (WURSS-24) for y-axis and duration of ARI illness as x-axis; Error lines indicate 95% confidence intervals.

Comparing the two MEPARI trials, it is apparent that the magnitude of observed ARI reduction was larger in the first trial (Table 4 and Fig 2). In the first MEPARI, proportional rate reductions of ARI incidence, days-of-illness, and global severity were 33%, 43% and 60% for MBSR, and 29%, 42%, and 31% for EX, compared to controls. The corresponding proportional rate reductions for MEPARI-2 were 16%, 14%, and 21% for MBSR, and 10%, 16%, and 31% for EX, respectively. Compared to control, ARI-related absenteeism was 76% lower for MBSR and 48% lower for EX in the first MEPARI, but only 30% lower for MBSR and 21% lower for EX in MEPARI-2. Pooling the two datasets, we found that unadjusted estimates of proportional reductions of incidence, days-of-illness, and global severity were 20%, 22%, and 33% for MBSR and 14%, 23%, and 31% for EX, compared to control. Pooled estimates for reductions in ARI-related absenteeism were 48% for MBSR and 32% for EX. Pooling individual level data also allowed an exploratory meta-analysis using zero-inflated multivariate regression models. These models suggested a reduction in ARI incidence attributable to MBSR (p = 0.044), and a decrease in total days of illness for EX (p = 0.004), but no statistically significant effect on global severity. See S1 File for model parameters and coefficients.

**Table 4:** Main ARI-Illness outcomes for the MEPARI and MEPARI-2 trials.

ARI-Related Outcome	MEPARI (n = 154)					MEPARI-2 (n = 413)					Pooled MEPARI and MEPARI-2 (n = 562)				
	CTL	MBSR	%DR^1^	EX	%DR^1^	CTL	MBSR	%DR^1^	EX	%DR^1^	CTL	MBSR	%DR^1^	EX	%DR^1^
n	51	51		47		138	138		137		189	189		184	
Had a ARI (n)	28	21	25	17	34	82	74	10	84	-3	110	95	14	101	6
Total # ARIs	40	27	33	26	29	134	112	16	120	10	174	139	20	146	14
Total Days with ARI	453	257	43	241	42	1210	1045	14	1010	16	1663	1302	22	1241	23
# Viruses identified	20	16	20	8	57	76	65	14	65	14	96	81	16	74	21
Mean ARI Global Severity (AUC) (per person)^2^	358	144	60	248	31	326	256	21	224	31	334	225	33	230	31
Mean ARI Global Severity (AUC) (per ARI)^3^	456	271	41	449	1.5	336	315	6	256	24	363	306	16	290	20
Total Days of Missed Work due to ARI	67	16	76	32	48	105	73	30	82	21	172	89	48	114	32
Total # of ARI-Related Healthcare Visits	16	10	38	15	-2	24	22	8	21	12	40	32	20	36	5

ARI = acute respiratory infection; CTL = control; MBSR = mindfulness-based stress reduction; EX = exercise;

Global severity = area under curve, y-axis WURSS-24 daily score, x-axis ARI illness duration

^1^%DR = Percent Difference in Rate, calculated as (control rate–intervention rate) / (control rate) *100 (equivalent to relative risk reduction for incidence)

^2^ per person with zeros if the participant did not have an ARI. %DR = Percent Difference, calculated as (control-intervention)/control*100

^3^ per ARI (no zeros). %D = Percent Difference, calculated as (control-intervention)/control*100.

### Conclusions/interpretation

The first MEPARI trial found statistically and clinically significant reductions in ARI illness for participants randomly assigned to 8 weeks of MBSR training, compared to observational controls [18]. In that trial there was evidence of reduced absenteeism for both mindfulness and matched exercise training [21], and trends towards an advantage of mindfulness over exercise [20]. The MEPARI-2 trial was designed to replicate and extend those findings, but enrolled people aged 30 to 69 years, rather than ≥ 50 years, as was done with the first trial, a decision driven by the absenteeism findings. Consistent with the first trial, we found the incidence, duration, and global severity of ARI illness in the MEPARI-2 trial were lower for both MBSR and EX groups than for control. The prespecified primary efficacy zero-inflated regression model suggested lower ARI incidence for MBSR (p = 0.036) and reduced global severity for EX (p = 0.042). However, neither of these met the p≤0.025 threshold for proof-of-efficacy that was set *a priori*. Nevertheless, we interpret the consistent pattern of apparent benefits across the two trials to suggest preventive effects ranging from 14–33% proportional reductions in ARI illness, and 32–48% reductions in ARI-related absenteeism (Table 4; Table 4 and S1 File are based on full n = 413 data set).

The question of clinical significance is more complex and nuanced than that for statistical significance. Much of this complexity lies in the fact that different people value potential benefits and harms differently, resulting in a range of benchmark values for “minimal important difference” [49–51], a concept which we suggest should be reframed as “sufficiently important difference” or “smallest worthwhile effect,” conceptual entities that consider harms as well as benefits [52–54]. We have previously conducted benefit harm trade-off studies to estimate clinical significance for ARI *treatment*, finding that 20–25% reductions in duration or severity of ARI illness would justify common treatments for most people [55–57]. However, we are not aware of any such work addressing *preventive* modalities.

Comparison to influenza vaccination (flu shots) may help put MEPARI results in perspective. Flu shots are known to reduce influenza, with published estimates of proportional reductions in symptomatic illness, medical visits, and absenteeism ranging from 13% to 70% [58–62]. Magnitude of benefit observed in various trials seems to depend mostly on the annual vaccine match, and the virulence of influenza strains. One of the more rigorous influenza vaccination trials found that those randomized to placebo actually had significantly fewer days of influenza-like illness (957 for placebo vs 1374 for flu shot; p = 0.01) during the 1997–98 winter, whereas during the 1998–99 winter those receiving flu shots experienced fewer ARI illness days (592) compared with placebo (920), as predicted (p<0.001) [60]. With this in mind, we hesitate to conclude that the larger benefits observed in the first MEPARI trial were due primarily to the older age of participants; year-to-year variability and other factors were likely involved.

While flu shots confer protection only against influenza, the evidence presented here suggests that mindfulness and exercise training may reduce ARI illness in general, regardless of etiological agent. In the two MEPARI trials, representing five cold-and-flu seasons, only 22 of 253 viral identifications (8.8%) were influenza. Other studies concur that while upwards of 50% of people may experience an ARI illness in a given year, the risk of influenza-specific illness is generally less than 10% [60,63–65].

The main limitations of this study are related to sample selection, sample size, and heterogeneity of outcomes assessed. As with all randomized trials, the sample was comprised of people who were willing to take part in all aspects of the study regardless of group assignment, drawing in participants who may not be fully representative of those at most risk for ARI illness in general. The target sample size was determined using the results of the first MEPARI trial, which had observed 29% to 60% proportional reductions in the incidence, duration and overall severity of ARI illness. Thus, this follow-up phase 2 trial was not statistically powered to detect the smaller 10% to 30% proportional reductions actually observed. Lastly, the assessment of ARI prevention is complicated by the large amount of annual variation in ARI illness, in terms of etiological agents circulating, and in terms of the incidence, duration and severity of symptomatic illnesses resulting.

A final important consideration relates to health impacts beyond ARI illness. Exercise is known to benefit people with diabetes and cardiovascular disease [66,67], may protect against cancer [68], and appears to confer psychological benefit [69–71]. Mindfulness training likewise appears to have pleiotropic effects, with reasonably strong data supporting its capacity to alleviate stress, anxiety, depression, and pain [72–77], and some preliminary evidence for cardiovascular and metabolic conditions [78–82]. In a clinical world characterized by multi-morbidity [83], interventions impacting a variety of conditions should be judged on the sum of their benefits, and not only on their effects on specific illnesses. Consistent with *a priori* hypotheses, MEPARI-2 found that mindfulness training led to significant reductions in stress, and to improvements in general mental health, mindful attention, and self-efficacy. In this study, exercise training also reduced stress and depressive symptoms, and improved sleep quality, self-efficacy, and mindful attention, all of which can be considered beneficial.

In summary, the evidence from the MEPARI-2 trial is consistent with modest reduction in ARI illness attributable to both MBSR and EX training. The magnitude of observed benefit is similar to that from accepted medical interventions such as influenza vaccination and should be considered in light of potential health benefits beyond ARI. Additional research into the benefits of exercise and meditation is certainly warranted, perhaps in higher risk or sicker populations, where there are more health benefits to gain. Until that research is accomplished, we feel it justifiable to advocate for both mindfulness and exercise, as benefits appear likely, and risks minimal.

## Supporting information

S1 File Tables. (PDF)

S1 Protocol (PDF)

S2 File Appendices. (PDF)

S1 Checklist CONSORT checklist of information for a randomized trial. (DOC)

S2 Checklist TIDieR (Template for Intervention Description and Replication) checklist. (DOCX)
