# Supplementary material for: Meditation or exercise for preventing acute respiratory infection (MEPARI-2): A randomized controlled trial
Source: PLoS One. 2018 Jun 22;13(6):e0197778. doi: 10.1371/journal.pone.0197778 (PMC6014660; doi:10.1371/journal.pone.0197778)
Supplement: S1 File — (PDF) [file pone.0197778.s001.pdf]

Supplemental Table A: MEPARI and MEPARI-2 Trials (Separately and Pooled) Zero-Inflated Models for ARI Duration Days

**Count Predictors: TOTDAYS[1,3]**	**MEPARI**		**MEPARI-2**		**Pooled Trial Data**	
	**Estimate[2] (SE)**	**P-value**	**Estimate[2] (SE)**	**P-value**	**Estimate[2] (SE)**	**P-value**
Group 1: Exercise	-0.013 (0.20)	0.47	-0.11 (0.09)	0.11	-0.11 (0.04)	**0.004**
Group 2: Meditation	-0.43 (0.23)	0.033	0.013 (0.09)	0.45	-0.012 (0.04)	0.76
Cohort (2)	-0.26 (0.20)	0.10	-0.096 (0.11)	0.19	-0.16 (0.07)	0.021
Cohort 3	-	-	-0.15 (0.11)	0.09	-0.12 (0.06)	0.035
Cohort 4	-	-	0.076 (0.11)	0.25	-0.23 (0.05)	<0.001
(Cohort 5)	-	-	-	-	-0.35 (0.06)	<0.001
(Cohort 6)	-	-	-	-	-0.078 (0.06)	0.17
Age	0.013 (0.017)	0.22	0.000 (0.004)	0.46	0.001 (0.002)	0.34
Smoking Status	-0.35 (0.22)	0.059	-0.33 0.23)	0.07	-0.45 (0.08)	<0.001
Education	0.023 (0.10)	0.41	-0.005 (0.053)	0.46	-0.028 (0.019)	0.14
BMI	0.010 (0.014)	0.24	-0.006 (0.006)	0.16	0.004 (0.002)	0.068
SF-12 Physical Health	-0.004 (0.012)	0.37	-0.008 (0.006)	0.10	-0.009 (0.002)	<0.001
SF-12 Mental Health	0.007 (0.013)	0.30	0.001 (0.005)	0.42	-0.003 (0.002)	0.09
Sex	-0.023 (0.18)	0.45	-0.175 (0.096)	0.034	-0.11 (0.04)	0.006
BFI: Conscientiousness	-	-	0.001 (0.008)	0.44	-	-
BFI: Neuroticism	-	-	0.009 (0.008)	0.11	-	-
SIC Comorbidity	-	-	0.044 (0.024)	0.035	-	-
Intercept: **TOTDAYS**	1.71 (1.81)	0.17	2.30 (0.70)	<0.001	2.98 (0.21)	<0.001
**Predictors of Excess Zero: TOTDAYS#1**[3]						
Group 1: Exercise	0.82 (0.45)	0.034	0.11 (0.25)	0.33	0.22 (0.21)	0.29
Group 2: Meditation	0.50 (0.42)	0.12	0.43 (0.24)	0.036	0.41 (0.20)	0.044
Cohort (2)	0.084 (0.39)	0.42	-0.34 (0.28)	0.12	0.19 (0.32)	0.55
Cohort 3	-	-	0.06 (0.28)	0.42	-0.51(0.30)	0.09
Cohort 4	-	-	0.29 (0.28)	0.15	-0.70 (0.29)	0.015
(Cohort 5)	-	-	-	-	-0.21 (0.29)	0.47
(Cohort 6)	-	-	-	-	-0.015 (0.20)	0.96
Age	0.049 (0.028)	0.041	0.027 (0.011)	0.009	0.033 (0.009)	<0.001
Smoking Status	-2.53 (1.11)	0.011	0.49 (0.49)	0.16	-0.29 (0.38)	0.45
Education	-0.087 (0.21)	0.34	-0.29 (0.12)	0.009	-0.24 (0.10)	0.016
BMI	-0.038 (0.031)	0.11	-0.019 (0.014)	0.09	-0.010 (0.013)	0.41
SF-12 Physical Health	0.015 (0.024)	0.26	-0.037 (0.014)	0.004	-0.005 (0.011)	0.63
SF-12 Mental Health	0.016 (0.029)	0.29	-0.016 (0.013)	0.10	-0.000 (0.009)	0.97
Sex	0.021 (0.45)	0.48	0.42 (0.24)	0.043	0.35 (0.21)	0.09
BFI: Conscientiousness	-	-	0.008 (0.020)	0.34	-	-
BFI: Neuroticism	-	-	-0.025 (0.021)	0.12	-	-
SIC Comorbidity	-	-	-0.006 (0.064)	0.46	-	-
Intercept: **TOTDAYS#1**	-3.24 (3.34)	0.17	2.30 (1.71)	0.09	-0.88 (1.09)	0.42

SE=standard error of estimate; BMI = body mass index; SF-12 = 12-item Medical Outcomes Study Short Form; TOTDAYS = total days of acute respiratory infection.

Notes: Group 1 (exercise =1: control or meditation =0); group 2 (meditation =1: control or exercise=0); sex (male=1: female=0); smoking status (current smoker=1: nonsmoker=0)

MEPARI: cohort (2) (cohort 2 =1: cohort 1=0 (ref))

MEPARI-2: cohort (2) (cohorts 1,3,4 =0: cohort 2=1); cohort 3 (cohorts 1,2,4 =0: cohort 3=1); cohort 4 (cohorts 1,2,3 =0: cohort 4=1)

Pooled: cohort (2) (cohorts 1,3,4,5,6 =0: cohort 2=1); cohort 3 (cohorts 1,2,4,5,6 =0: cohort 3=1); cohort 4 (cohorts 1,2,3,5,6 =0: cohort 4=1) ; cohort 5 (cohorts 1,2,3,4,6 =0: cohort 5=1) ; cohort 6 (cohorts 1,2,3,4,5 =0: cohort 6=1); cohort 1 from MEPARI is reference group, cohorts 3-6 are MEPARI-2 cohorts.

P-values < 0.025 are statistically significant.

1 Box Cox transformations (log transformations) were used before modeling Total Days.

2 Negative parameter estimates for the count predictors indicate lesser severity (fewer days); positive parameter estimates for the predictors of excessive zeros indicate a greater likelihood to NOT have an ARI during the trial period.

3TOTDAYS (Poisson model); TOTDAYS#1 (zero-inflated model).

Supplemental Table B. MEPARI and MEPARI-2 Trials (Separately and Pooled) Zero-Inflated Models for ARI Global Severity (AUC)

**Count Predictors: AUC[1,3]**	**MEPARI Estimate[2] (SE)**	**P-value**	**MEPARI-2 Estimate[2] (SE)**	**P-value**	**Pooled Trial Data Estimate[2] (SE)**	**P-value**
Group 1: Exercise	-0.16 (0.32)	0.31	-0.23 (0.14)	0.042	-0.043 (0.090)	0.63
Group 2: Meditation	-0.74 (0.32)	**0.010**	0.033 (0.15)	0.41	-0.050 (0.088)	0.57
Cohort (2)	-0.46 (0.26)	0.40	-0.39 (0.15)	0.005	-0.24 (0.18)	0.17
Cohort 3	-	-	-0.34 (0.15)	0.013	-0.17 (0.14)	0.20
Cohort 4	-	-	0.095 (0.18)	0.30	-0.27 (0.14)	0.052
(Cohort 5)	-	-	-	-	-0.30 (0.14)	0.034
(Cohort 6)	-	-	-	-	-0.23 (0.14)	0.11
Age	0.026 (0.025)	0.15	0.005 (0.006)	0.20	-0.002 (0.004)	0.64
Smoking Status	-0.028 (0.34)	0.47	-0.14 0.33)	0.33	-0.27 (0.15)	0.079
Education	0.038 (0.15)	0.40	-0.077 (0.077)	0.16	-0.006 (0.046)	0.12
BMI	0.025 (0.024)	0.15	-0.006 (0.009)	0.27	-0.002 (0.005)	0.73
SF-12 Physical Health	-0.021 (0.016)	0.10	-0.030 (0.009)	<0.001	-0.005 (0.005)	0.33
SF-12 Mental Health	0.004 (0.019)	0.42	0.011 (0.008)	0.065	-0.005 (0.004)	0.21
Sex	-0.003 (0.26)	0.50	-0.32 (0.14)	0.012	-0.16 (0.10)	0.12
BFI: Conscientiousness	-	-	0.019 (0.012)	0.053	-	-
BFI: Neuroticism	-	-	0.010 (0.012)	0.21	-	-
SIC Comorbidity	-	-	0.073 (0.040)	0.031	-	-
Intercept: **AUC**	5.51 (2.27)	0.008	7.34 (1.09)	<0.001	1.55 (0.50)	0.002
**Predictors of Excess Zero: AUC#1**[3]						
Group 1: Exercise	0.83 (0.45)	0.032	0.12 (0.25)	0.33	0.68 (0.37)	0.069
Group 2: Meditation	0.60 (0.42)	0.079	0.42 (0.23)	0.036	0.42 (0.40)	0.29
Cohort (2)	0.16 (0.40)	0.34	-0.33 (0.28)	0.12	-0.17 (0.47)	0.72
Cohort 3	-	-	0.072 (0.28)	0.40	-1.13 (0.51)	0.025
Cohort 4	-	-	0.28 (0.28)	0.15	-2.26 (1.15)	0.050
(Cohort 5)	-	-	-	-	-1.09 (0.56)	0.051
(Cohort 6)	-	-	-	-	-0.57 (0.46)	0.21
Age	0.05 (0.028)	0.038	0.027 (0.011)	0.009	0.056 (0.023)	0.015
Smoking Status	-2.51 (1.10)	0.012	0.48 (1.06)	0.14	-15.2 (824)	0.99
Education	-0.041 (0.21)	0.42	-0.29 (0.12)	0.009	-0.36 (0.19)	0.064
BMI	-0.038 (0.031)	0.11	-0.019 (0.014)	0.09	-0.028 (0.024)	0.23
SF-12 Physical Health	0.018 (0.024)	0.23	-0.037 (0.014)	0.004	-0.011 (0.020)	0.61
SF-12 Mental Health	0.016 (0.029)	0.31	-0.016 (0.012)	0.10	-0.022 (0.017)	0.20
Sex	0.062 (0.45)	0.45	0.43 (0.24)	0.037	-0.22 (0.57)	0.70
BFI: Conscientiousness	-	-	0.008 (0.019)	0.34	-	-
BFI: Neuroticism	-	-	-0.025 (0.021)	0.11	-	-
SIC Comorbidity	-	-	-0.008 (0.063)	0.45	-	-
Intercept: **AUC#1**	-3.76 (3.36)	0.13	2.29 (1.70)	0.09	0.065 (0.194)	0.97

AUC = area under time-severity curve (global severity); SE=standard error of estimate; BMI = body mass index; SF-12 = 12-item Medical Outcomes Study Short Form.

Notes: Group 1 (exercise =1: control or meditation =0); group 2 (meditation =1: control or exercise=0); sex (male=1: female=0); smoking status (current smoker=1: nonsmoker=0)

MEPARI: cohort (2) (cohort 2 =1: cohort 1=0 (ref))

MEPARI-2: cohort (2) (cohorts 1,3,4 =0: cohort 2=1); cohort 3 (cohorts 1,2,4 =0: cohort 3=1); cohort 4 (cohorts 1,2,3 =0: cohort 4=1)

Pooled: cohort (2) (cohorts 1,3,4,5,6 =0: cohort 2=1); cohort 3 (cohorts 1,2,4,5,6 =0: cohort 3=1); cohort 4 (cohorts 1,2,3,5,6 =0: cohort 4=1) ; cohort 5 (cohorts 1,2,3,4,6 =0: cohort 5=1) ; cohort 6 (cohorts 1,2,3,4,5 =0: cohort 6=1); cohort 1 from MEPARI is reference group, cohorts 3-6 are MEPARI-2 cohorts.

P-values < 0.025 are statistically significant.

[1] Box Cox (log) transformation was used before modeling AUC.

[2] Negative parameter estimates for the count predictors indicate lesser severity; positive parameter estimates for the predictors of excessive zeros indicate a greater likelihood to NOT have an ARI during the trial period.

[3]AUC (Poisson model); AUC#1 (zero-inflated model).
