# Supplementary material for: Meditation or exercise for preventing acute respiratory infection (MEPARI-2): A randomized controlled trial
Source: PLoS One. 2018 Jun 22;13(6):e0197778. doi: 10.1371/journal.pone.0197778 (PMC6014660; doi:10.1371/journal.pone.0197778)
Supplement: S1 Protocol — (PDF) [file pone.0197778.s002.pdf]

DEPARTMENT OF
FAMILY MEDICINE
University of Wisconsin
School of Medicine and Public Health

RESEARCH PROTOCOL
MANUAL

Meditation or Exercise for Preventing Acute Respiratory Infection (MEPARI-2)

A phase II parallel 3-group randomized controlled trial of the preventive effects of meditation or exercise on acute respiratory infection

May 29, 2015

**Principal Investigator:**	Bruce Barrett MD PhD<br>University of Wisconsin-Madison<br>Department of Family Medicine<br>1100 Delaplaine Court<br>Madison, WI 53715
**Co-Investigators**	Mary Hayney PharmD, Christopher Coe PhD, Daniel Muller MD PhD, David Rakel MD, Roger Brown PhD, Aleksandra Zgierska MD PhD, Zhengjun Zhang PhD
**Consultants**	James Gern MD, David Nieman PhD, Richard Davidson PhD, Marlon Mundt PhD
**Behavioral trainings**	Mindfulness Program and Sports Medicine Program<br>University of Wisconsin Hospitals and Clinics
**Project coordinators**	Shari Barlow, Michele Gassman MA
**Data management**	Tola Ewers MS, Don Thomson, Larissa Zakletskaia MA
**Funding Sponsor:**	National Institutes of Health, National Center for Complementary and Alternative Medicine
**Protocol Number:**	1 R01 AT006970-01

Initial version: March 16, 2012
Amended: April 13, 2012 [see Appendix I for amendment details, all minor]
Amended: July 18, 2012
Amended: July 27, 2013
Amended: August 15, 2014
Amended: May 29, 2015

SIGNATURE PAGE

The signature below constitutes the approval of this protocol and the attachments, and provides the necessary assurances that this study will be conducted according to all stipulations of the protocol, including all statements regarding confidentiality, according to local legal and regulatory requirements and applicable US federal regulations and ICH guidelines, and according to the University of Wisconsin Health Sciences Institutional Review Board requirements.

All key personnel (all individuals responsible for the design and conduct of this study) have completed Human Subjects Protection Training.

Principal Investigator: Bruce Barrett MD PhD

Signed: ______________________________ Date: May 29, 2015

TABLE OF CONTENTS page

PROTOCOL SUMMARY…… 1

1. **INTRODUCTION**…… 2
   - 1.1 BACKGROUND…… 2
     - 1.1.1 Acute Respiratory Infection…… 2
     - 1.1.2 Influenza…… 3
     - 1.1.3 Stress and Health…… 3
     - 1.1.4 Mindfulness Meditation…… 3
     - 1.1.5 Exercise…… 3
   - 1.2 PRELIMINARY TRIAL…… 3
   - 1.3 MAIN RESULTS PRELIMINARY TRIAL…… 4
2. **STUDY OBJECTIVES** and **SPECIFIC AIMS** …… 4
3. **STUDY DESIGN**…… 5
   - 3.1 GENERAL DESIGN…… 5
   - 3.2 CONCEPTUAL FRAMEWORK…… 5
   - 3.3 MULTIDISCIPLINARY RESEARCH TEAM…… 6
   - 3.4 PRIMARY STUDY ENDPOINTS…… 6
   - 3.5 SECONDARY STUDY ENDPOINTS…… 6
4. **SAMPLE SELECTION** …… 7
   - 4.1 INCLUSION/ EXCLUSION CRITERIA…… 7
     - 4.1.1 Inclusion criteria…… 7
     - 4.1.2 Exclusion criteria…… 8
   - 4.2 PARTICIPANT RECRUITMENT AND SCREENING…… 8
     - 4.2.1 Retention…… 8
     - 4.2.2 Study Promotion…… 8
     - 4.2.3 Screening…… 8
     - 4.2.4 Run-in trial for adherence assessments…… 9
   - 4.3 DATA COLLECTION AND FOLLOW-UP FOR WITHDRAWN SUBJECTS…… 9
5. **STUDY INTERVENTIONS**…… 9
   - 5.1 STUDY ARMS…… 9
     - 5.1.1 Mindfulness Meditation…… 9
     - 5.1.2 Exercise…… 9
     - 5.1.3 Wait-list Control…… 10
   - 5.2 RANDOMIZATION…… 10
   - 5.3 BLINDING…… 10
   - 5.4 PARTICIPANT COMPLIANCE AND MONITORING…… 10
     - 5.4.1 Weekly monitoring and adherence assessment…… 10
     - 5.4.2 Exercise and meditation practice monitoring…… 10
6. **STUDY MEASUREMENTS & PROCEDURES**…… 11
   - 6.1 STUDY PARTICIPANT OVERVIEW…… 11
   - 6.2 PROJECT TIMELINE…… 11
   - 6.3 MEASURES OF ARI ILLNESS…… 12
     - 6.3.1 Definition of ARI illness…… 12
     - 6.3.2 WURSS-24 The Wisconsin Upper Respiratory Symptom Survey…… 12
     - 6.3.3 RIDL…… 12
     - 6.3.4 Viral identification…… 12
     - 6.3.5 Pro-inflammatory Cytokine…… 12
     - 6.3.6 Inflammatory tendency…… 12
     - 6.3.7 Polymorphonuclear neutrophil count…… 13
     - 6.3.8 Glycosylated hemoglobin (HgA1C) …… 13
   - 6.4 PSYCHOSOCIAL AND PHYSICAL HEALTH MEASURES…… 13
     - 6.4.1 Alcohol and tobacco use (TLFB Timeline Followback Method) …… 13
     - 6.4.2 Body Mass Index (BMI)…… 13
     - 6.4.3 Demographics…… 13
     - 6.4.4 Seattle Index of Comorbidity (SIC) …… 13
     - 6.4.5 Big Five Inventory (BFI) …… 13
     - 6.4.6 Health care utilization and antibiotics prescribed …… 13
     - 6.4.7 Days of work and school missed to illness…… 14
     - 6.4.8 Economic outcomes…… 14

6.4.9 Absenteeism/Presenteeism…… 14
6.4.10 Depression Screen (PHQ-9)…… 14
6.4.11 Health-related quality of life (SF-12)…… 14
6.4.12 Pittsburgh Sleep Quality Index (PSQI)…… 14
6.4.13 Positive and Negative Affect Schedule (PANAS)…… 14
6.4.14 Perceived Stress Scale (PSS-10)…… 14
6.4.15 Social Provisions Scale (SPS)…… 15
6.4.16 Social Network Index (SNI)…… 15
6.4.17 Feeling Loved (FL)…… 15
6.4.18 Exercise Self-Efficacy Scale (ESES)…… 15
6.4.19 Mindfulness-based Self Efficacy Scale (MSES)…… 15
6.4.20 Mindfulness Attention Awareness Scale (MAAS)…… 15
6.4.21 Global Physical Activity Questionnaire (GPAQ)…… 15
6.4.22 Mindfulness practice and exercise daily tracking log…… 15
6.4.23 Expectancy…… 15
6.4.24 Blood pressure…… 15
6.4.25 Accelerometry and breath-counting in final cohort …… 15

6.5 TIMELINE FOR BASELINE MEASURES, COVARIATES & OUTCOMES…… 16
6.6 WRITTEN INFORMED CONSENT …… 17
6.7 STUDY VISIT CHECKLISTS…… 17
6.8 CLINICAL RESEARCH UNIT…… 17
6.8.1 CRU Overview…… 17
6.8.2 Nursing Services…… 17
6.8.3 Admission to CRU…… 17
6.8.4 Billing: Office of Clinical Trials…… 17
6.8.5 Physician's Orders…… 17
6.9 PHARMACEUTICAL RESEARCH CENTER…… 17
6.9.1 Influenza Vaccination…… 17

7. STATISTICAL PLAN…… 18
7.1 SAMPLE SIZE DETERMINATION…… 18
7.2 STATISTICAL METHODS…… 19
7.2.1 Loss to follow up…… 19
7.2.2 Analysis overview…… 19
7.2.3 Descriptive analyses…… 19
7.2.4 Global severity…… 19
7.2.5 Primary efficacy analysis…… 19
7.2.6 Secondary analyses…… 19
7.2.7 Subgroup analyses…… 20
7.2.8 Longitudinal analysis…… 20
7.2.9 Intention to treat analysis…… 20
7.2.10 Process analysis (mediation)…… 20
7.2.11 Economic analyses…… 20
7.2.12 Absenteeism/Presenteeism…… 20

8. PROTECTION OF HUMAN SUBJECTS…… 21
8.1 OVERVIEW…… 21
8.2 RISKS AND BENEFITS…… 21
8.2.1 Risks and benefits summary…… 21
8.2.2 Risks and benefits to society…… 21
8.2.3 Adverse effects…… 22
8.2.4 Safety of trivalent inactivated influenza vaccine…… 22
8.2.5 Safety of exercise…… 22
8.2.6 Safety of meditation…… 22
8.2.7 Adventitious findings…… 22
8.2.8 Nasal wash…… 23
8.2.9 Nasal swabs…… 23
8.2.10 Phlebotomy…… 23
8.2.11 Biological specimens…… 23

8.3 INCLUSION OF WOMEN AND MINORITIES…… 23
8.3.1 Women…… 23

8.3.2 Minorities…… 23
8.4 INCLUSION OF CHILDREN…… 24
8.5 DATA AND SAFETY MONITORING PLAN…… 24
8.6 VULNERABLE POPULATIONS……24
8.6.1 Pregnancy……25
8.7 ADVERSE EVENTS……25
8.8 REPORTING SERIOUS ADVERSE EVENTS……25
8.9 PROTOCOL REGISTRATION…… 25
8.10 IMPORTANCE OF THE KNOWLEDGE TO BE GAINED…… 25

9. DATA HANDLING AND RECORD KEEPING…… 26
9.1 CONFIDENTIALITY……26
9.1.1 Protection of subject privacy…… 26
9.1.2 Database protection…… 26
9.1.3 Confidentiality during AE reporting…… 26
9.2 HEALTH INFORMATION…… 26

10. STUDY FINANCES…… 26
10.1 FUNDING SOURCE…… 26
10.2CONFLICT OF INTEREST…… 27
10.3SUBJECT COMPENSATION…… 27
10.4COSTS TO STUDY PARTICIPANTS…… 27

11. PUBLICATION PLAN…… 27

12. REFERENCES…… 28

APPENDICES

Appendix A: STUDY INTERVENTIONS
Meditation
Exercise

Appendix B: QUESTIONNAIRE INSTRUMENTS
Wisconsin Upper Respiratory Symptom Survey (WURSS-24)
Alcohol and Tobacco Use Report Form (TimeLine Followback)
Demographics
Seattle Index of Co-Morbidity (SIC)
Big Five Inventory (BFI)
Health Care Utilization (HCU)
Stanford Presenteeism Scale (StPS)
PHQ-9 (Depression screen)
General physical and mental health (SF-12)
Pittsburgh Sleep Quality Index (PSQI)
Positive and Negative Affect Schedule (PANAS)
Perceived Stress Scale (Cohen PSS-10)
Social Provisions Scale (SPS)
Social Network Index (SNI)
Feeling Loved (FL)
Exercise Self-Efficacy Scale (ESES)
Mindfulness-Based Self Efficacy Scale (MSES)
Mindfulness Attention Awareness Scale (MAAS)
Global Physical Activity Questionnaire (GPAQ)
Expectancy Ratings
Meditation Log
Exercise Log

Appendix C: QUESTIONAIRRE SCORING

Appendix D: LAB PROTOCOLS
- Viral Identification
- Interleukins 6 and 8 (IL-6, IL-8)
- C-Reactive protein (CRP), Procalcitonin (PCT)
- Interferon-gamma-induced protein 10 (IP-10)
- Nasal neutrophils

Appendix E: STUDY PROCEDURES
- Schedule of Assessments
- Questionnaire Cover Sheets
- Study Visit Checklists
- Telephone Screening Card
- Physician’s Orders
- Nasal Lavage Information Sheet
- Nasal Wash Lab Work Information Sheet
- PRC Protocol Summary
- CRU Application
- Information and Summary Sheet
- Informed Consent Form

Appendix F: DATA and SAFETY MONITORING PLAN
- CRU DSMP Sheet
- NCCAM Data and Safety Monitoring Plan

Appendix G: RECRUITING MATERIALS/CALENDAR

Appendix H: STATISTICAL ANALYSIS
- Zero-inflated multivariate regression models
- Mediation
- Power

Appendix I: STUDY LOG: DECISIONS, MODIFICATIONS and CLARIFICATIONS

Appendix J: ACCELEROMETRY & BREATH COUNTING

Protocol Summary

Background

Preliminary evidence suggests that 8-week training programs in meditation and exercise lead to reductions in incidence, duration and severity of acute respiratory infection (ARI) illness.[1]

Methods

In this parallel 3-group phase 2 trial, women and men aged 30 to 69 will be randomized to: 1) an 8-week behavioral training program in mindfulness meditation, 2) an intensity, duration and location-matched 8-week program in sustained moderate intensity exercise, or 3) a wait-list observational control group. Recruitment will target those who do not exercise regularly, and have not had training in meditation.

Outcomes

The primary outcome will be severity-weighted total days of ARI illness as assessed by self-reports on the validated Wisconsin Upper Respiratory Symptom Survey (WURSS-24). Number of ARI illness episodes, total days of ARI illness, ARI-related visits to health care facilities, and time lost from work and school due to ARI illness will be assessed as secondary outcomes. Weekly computer-assisted telephone monitoring for ARI illness will be conducted by personnel blinded to intervention group. Blood and nasal wash samples will be obtained at baseline, one month after the end of the 8-week interventions, and once again three months later. Blood and nasal wash samples will be obtained approximately 24-72 hours into each ARI episode. Nasal wash samples will be tested with multiplex PCR (polymerase chain reaction) to identify etiological agents. Serum and nasal wash will be analyzed for interleukin-6, interleukin-8, C-reactive protein, procalcitonin, and interferon-gamma-induced protein 10. These inflammatory biomarkers will serve as objective indicators of disease severity to compare with illness severity self-reported on the WURSS-24. Study participants will fill out validated questionnaires assessing perceived stress, self-efficacy, mindfulness, social support, and general mental and physical health at baseline and at least twice after behavioral trainings. Inflammatory biomarkers and psychosocial indicators will be analyzed as potential mediators of causal pathways leading from behavioral training interventions to ARI illness outcomes.

Timeframe / logistics

This will be a 5-year project, with 4 yearly cohorts of n=99 per cohort randomized into 3 groups of n=33 each. Assuming 9% loss to follow-up, the final sample size will be n=360 study participants, with n=120 in each comparison group. Enrollment, randomization and study interventions will begin in September of each year. Participants will be monitored by weekly self-report through May. Summers will be used for data cleaning, preliminary analyses, and for recruiting the next year's cohort.

Analysis

Analyses will proceed as follows: 1) descriptive analyses of all variables; 2) assessment and potential response to outliers and missing data; 3) calculation of area-under-curve severity weighted days of ARI illness; 4) primary efficacy analyses; and 5) secondary analyses, including assessment of intervention impact on secondary outcomes, longitudinal analyses, and process (mediation) analyses. Primary efficacy will be assessed using zero-inflated multivariate regression models. Generalized estimating equations, random-effects pattern-mixture models, and hierarchical linear models will be used to assess longitudinal effects, interactions, and covariate mediation.

1 Introduction

Acute respiratory infection (ARI), including common cold and influenza, is a leading cause of morbidity and mortality, and has a major economic impact.[2-7] Both mental and physical health are linked to ARI burden.[8-12] For example, people who report more negative emotion and higher stress are more likely to get ARI.[13-15] Exercise affects the immune system, improves physical and mental health, and may protect against ARI illness.[16-21] Mindfulness meditation reduces perceived stress,[22-27] influences the immune system,[28-35] and may protect against ARI.[1] Our own recent NCCAM-funded trial randomized 154 people to 3 groups: 1) meditation, 2) moderate intensity exercise, or 3) wait-list control. For the 149 people followed to study completion, there were 40 ARI episodes and 453 days of illness in the control group, 27 episodes and 257 days of ARI illness in the meditation group, and 26 episodes and 241 days of ARI illness in the exercise group.[1] Corresponding reductions in ARI-related work days lost to ARI illness were observed. The proposed research will build upon these findings with refined methodology in a larger sample to: A) determine whether these findings are replicable, and B) investigate potential explanatory pathways.

This research will use state-of-the-art randomized controlled trial (RCT) methodology to assess potential effects of meditation or exercise on ARI outcomes. Four cohorts of n=99 people each (total n=396) will be randomized to 3 groups: 1) an 8-week training program in mindfulness meditation, 2) an attention, duration and location-matched program in progressive exercise, or 3) a non-interventional wait-list control group. Each cohort will be observed for 8 months comprising the annual cold and flu season. The primary outcome will be area-under-the-curve severity-weighted days of ARI illness, with severity assessed using the validated Wisconsin Upper Respiratory Symptom Survey (WURSS-24). Secondary outcomes will include ARI-related health care visits, work time lost to ARI illness, and total work time lost.

Blood and nasal wash samples will be collected at baseline, 1 and 4 months after the end of the 8-week intervention, and approximately 24-72 hours into each ARI episode. These samples will be assayed for neutrophil count and pro-inflammatory cytokine levels: interleukin-6 (IL-6), interleukin-8 (IL-8), C-reactive protein (CRP), procalcitonin (PCT), and interferon-gamma-induced protein 10 (IP-10) levels). [Procalcitonin assays were dropped in 2014 due to all nondetectable values] Nasal wash samples collected during ARI illness episodes will also be tested for nucleic acid using multiplex PCR (polymerase chain reaction) methods.[36]

Self-report measures of perceived stress, positive and negative emotion, self-efficacy, social support, sleep quality, mindfulness, and general mental and physical health will be used to assess potential pathways through which interventions may exert influence on primary outcomes. A zero-inflated Poisson regression model will test whether interventions influence severity and duration of ARI illness episodes. Potential mediating effects of psychological and physical health domains will be assessed using appropriate mediation (process) analysis statistical models.

Acute infection from influenza and other respiratory viruses leads to much human suffering and loss of economic productivity. Our own evidence suggests that training in either meditation or exercise may lead to substantial reductions in ARI disease burden and work absenteeism.[1] In addition to testing whether our findings are replicable in a larger sample with refined methodology, this proposed comparative effectiveness translational research will investigate mechanisms of action and provide initial estimates of cost-effectiveness. If positive findings are confirmed, this line of research could have direct and immediate impact on public and private health-related policies and clinical practice, as well as on scientific understanding of respiratory infection.

1.1 Background

1.1.1 Acute respiratory infection (ARI) is responsible for tremendous health burden.[2-7] While influenza (flu) is often classified separately, its symptoms are usually indistinguishable from those produced by other etiological agents,[37-42] which include rhinovirus, coronavirus, parainfluenza, respiratory syncytial virus, adenovirus, enterovirus, and metapneumovirus.[43-48] In the U.S. alone, non-influenza ARI accounts for total economic impact of about $40 billion, putting ARI in the top 10 most expensive illnesses.[3;49-52] Available treatments are only minimally effective at reducing symptoms.[53-55] Immunization strategies are impractical as there are hundreds of antigenically distinct viral strains. Conventional preventive strategies are limited to contact avoidance and hand washing.[56]

1.1.2 Influenza is the most serious of the viral respiratory infections, and continues to be a major cause of morbidity and mortality. Each year, approximately 36,000 deaths[57;58] and more than 500,000 hospitalizations[59] in the U.S are associated with influenza infection. Influenza vaccination (flu shot) is widely accepted as efficacious and even cost effective,[57-64] but is imperfect. Seroprotection rates range from 60-80% in healthy younger adults[65-67] to perhaps 40 to 60% in the elderly.[68-75] A 2006 review concluded that flu shots have a "clinical efficacy in the elderly of 17-53%."[60]

1.1.3 Stress, inflammation and health Relationships between psychological stress and physical health are complex, but well-documented.[76-81] Perceived stress has been linked to depression,[82-84] cardiovascular disease,[85-88] and mortality.[89-92] Most relevant to this proposal, stress has been linked to various immune-inflammatory processes,[93-98] and to ARI illness.[8;14;15;99-108] Stressed people have higher levels of "stress homones"[109-111] and may respond to infection with higher levels of pro-inflammatory cytokines, including C-reactive protein (CRP),[112-114] procalcitonin (PCT),[115-121] and interferon-gamma induced protein 10 (IP-10).[122-128] For stressed people who are at higher risk for ARI illness, exercise appears to mediate that risk.[129] Our own preliminary research suggests that trainings in mindfulness based stress reduction may work to lower the incidence, duration and severity of ARI illness.[1]

1.1.4 Mindfulness meditation is a technique used to train the attention and enhance awareness. The concept of "mindfulness" refers to the nonjudgmental awareness of - and attention to - bodily sensations, thoughts, and emotions as they are happening in the present moment. The most common mindfulness meditation training program taught in medical settings is known as Mindfulness Based Stress Reduction (MBSR).[130-132] MBSR facilitators emphasize self-appreciation, compassion, and empathy, which may bring their own salutary effects. MBSR has been reported to reduce stress and anxiety,[22;26;27;133-137] psychological distress,[138] pain,[130]depression,[139;140] and to increase quality of life among people with chronic illness.[141] No study before ours has examined the effects of MBSR on ARI illness, or in the context of comparison to effects of exercise.

1.1.5 Exercise is known to positively influence health,[142-145] and to predict measures of both immunity[146-158] and respiratory infection.[159-166] Nevertheless, few prospective studies have assessed the effects of exercise interventions on ARI illness or immune mechanisms. Of those that have, results have tended to be positive.[16;167-169] For example, Chubak et al. randomized n=115 overweight post-menopausal women to either 45 minutes per day of moderate intensity exercise, or to a low-intensity stretching program (control).[16] Over a 12-month observation period, 30.2% of those randomized to exercise reported at least one ARI illness episode, compared to 48.4% of the control group (p = 0.03).[16] As another example, study Consultant David Nieman followed n=1,002 men and women for 12 weeks, and found an average of 8.18 days of ARI illness in the lowest exercise tertile, compared to 4.41 days in the highest exercise tertile (p<0.001).[168] Nevertheless, most research to date has been limited by the lack of randomization, sample size, outcomes measured, choice of control group, and observation period. The trial proposed here would provide both methodological rigor and adequate statistical power to detect meaningful effects on ARI illness.

1.2 Preliminary Trial

The research protocol described here follows directly from a recently completed NCCAM-funded study (1R01AT004313) in which we randomized n=154 people to 3 groups: 1) 8-weeks of training in mindfulness meditation, 2) matched 8-weeks of training in exercise, or 3) wait list control. A total of 94 participants were randomized in September 2009, and 60 more in January 2010. Only 5 withdrew early, with 149 followed through May 2010. There were 27 ARI episodes and 257 days of ARI illness in the meditation group (n=51) and 26 episodes and 241 ARI illness days for exercise (n=47), compared to 40 episodes and 453 ARI illness days for the control group (n=51). Mean area-under-curve global severity was 144 for meditation, 248 for exercise, and 358 for control. Comparing meditation to control, one-sided T-test yielded P=0.034 for illness days and p=0.0042 for global severity. Comparing exercise to control, corresponding p-values were 0.032 for illness days and 0.16 for global severity. Adjusting for covariates with zero-inflated multivariate regression models, both total days of illness (p=0.033) and global severity (p=0.010) appeared to be lower for meditation, but not for exercise (p = 0.47 and p=0.31, respectively). There were 16 ARI-related health care visits and 67 sick days lost to work in the control group, compared to 15 visits and 32 sick days for exercise and 10 visits and 16 sick days for meditation. Multiplex polymerase chain reaction (PCR) confirmed virus in 19 of the control episodes, 14 for meditation, and 8 for exercise. The only 2 cases of confirmed influenza were in the control group. Neutrophil counts and interleukin-8 (IL-8) assays provided corroborating evidence that observed benefits were unlikely to be due to biased-self-report biases.[1]

1.3 Main results preliminary trial

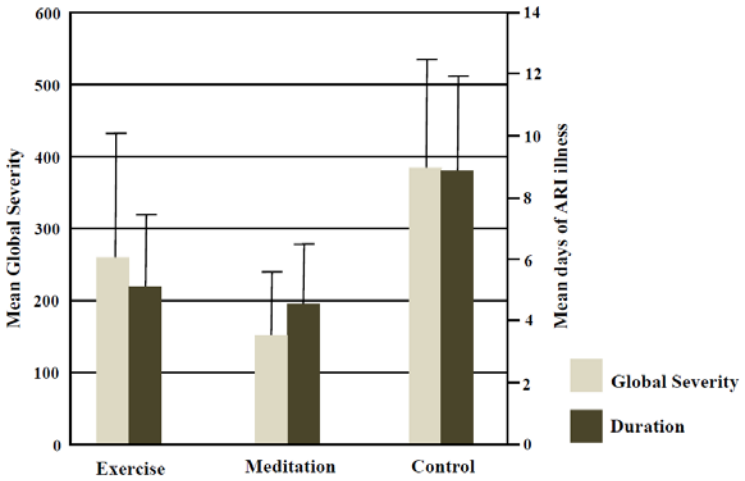

Figure 1 - Effects of exercise and meditation training on ARI illness days (duration) and area-under-curve global severity. Error bars are 95% confidence intervals.

Table 1 Preliminary trial main results

Main Results	EX	MM	CTL
Consented and randomized	51	51	52
Followed to main outcomes	47	51	51
People with ARI episodes	17*	21	28
# of ARI illness episodes	26	27	40
Total days of ARI illness	241*	257*	453
Mean duration of episodes	5.13*	5.04*	8.89
Mean AUC global severity	248*	144*	358
Episodes positive for virus	8	14	19
Mean interleukin-8	694	910	658
Mean neutrophil count	104	108	110
ARI-related health care visits	15	10	16
Total sick days lost to work	92	99	145
ARI-related sick days	32*	16*	67

EX=exercise; MM=mindfulness meditation; CTL=control
* = significant at $p \leq 0.05$ with 1-sided 2-group contrast

2. Study Objectives

To the best of our knowledge, the preliminary research described above is the first randomized trial to assess potential influences of mindfulness meditation on ARI illness, and the first to use a validated outcome measure to assess effects of exercise on ARI illness. It is also the first to compare both meditation and exercise to a valid control group, allowing head-to-head comparative effectiveness assessment. The proposed work is also innovative in terms of the degree of rigor employed in ARI surveillance and verification, and in the number and quality of immune and inflammatory biomarkers assayed. Finally, the proposed research will assess several psychosocial domains, both as potentially important outcomes, but also as mediational processes that could help to explain the causal pathways leading from behavioral interventions to ARI outcomes.

The primary goal of this project is to determine whether behavioral training in mindfulness meditation or moderate intensity sustained exercise will lead to reductions in acute respiratory infection (ARI) illness, such as common cold and influenza like illness. Our preliminary findings suggest substantial benefit of these interventions in terms of reduced incidence, duration and severity of ARI illness, with corresponding reductions in days of work lost to illness.[1] If the proposed research confirms these findings, there will be major implications for public and private health-related policy and practice, as well as for scientific knowledge regarding health maintenance and disease prevention.

Specific aims

1. **Determine whether an 8-week training program in mindfulness meditation, as compared to the control group, will lead to significant reductions in incidence, duration, and severity of ARI illness.**
2. **Determine whether an 8-week training program in moderate intensity sustained exercise, as compared to the control group, will lead to reductions in incidence, duration, and severity of ARI illness.**
3. **Assess whether any observed reductions in ARI illness are accompanied by fewer ARI-related health care visits and less time lost to productive work (reduced absenteeism).**
4. **Compare the potential benefits of mindfulness meditation to those from moderate intensity sustained exercise.**
5. **Discern potential mediating factors and causal pathways that might help explain how these interventions lead to improved ARI illness-related outcomes.**

3. Study Design

3.1 General Design

This will be a parallel, three group, phase 2 randomized controlled trial assessing the impact of training in meditation or exercise, compared to control, on incidence, duration and severity of ARI illness. Participants will be women and men aged 30 to 69 years who: report at least one ARI per year on average, and are willing and able to be randomized and follow through on all aspects of the protocol. The primary outcome will be severity-adjusted total days of ARI illness during one cold and flu season (September through May). Exercise and meditation training will be held in the same facility, and will be matched by in-class contact time (2 ½ hours/week) and out-of-class practice time (45 minutes daily). It is hoped that participants will continue their mindfulness or exercise practice long term. In order to standardize risk, all participants will receive influenza immunization (flu shots).

From consent onward, participants will be monitored once weekly using computer-assisted self-report ARI surveillance methods. Jackson criteria[170-172] will be used to define the beginning of each ARI episode. Respiratory infection daily logs (RIDLs) incorporating the WURSS-24 instrument[173-176] will be filled out once daily from the beginning of each ARI episode until the participant is no longer symptomatic. Study personnel administering these assessments will be blinded to intervention group status. Once ARI illness is identified, lab visits will be arranged. Prior to the lab visit, participants will self-swab both nostrils, bringing the swab to the lab visit.

Nasal wash and blood samples will be collected at baseline, 1 and 4 months after 8 week interventions, and once with each ARI episode. Samples will be analyzed for interleukin-6 (IL-6), interleukin-8 (IL-8), C-reactive protein (CRP), procalcitonin (PCT), and interferon-gamma-induced protein 10 (IP-10). Nasal swabs and wash collected during each ARI illness episode will be assessed for viral and bacterial RNA/DNA using multiplex PCR.[36]

This will be a 5-year project, with 4 yearly cohorts of n=99 per cohort randomized into 3 groups of n=33 each. Assuming 9% loss to follow-up, the final sample size will be n=360 study participants, with n=120 in each comparison group. Enrollment, randomization and study interventions will begin in September of each year, and participants will be monitored by weekly self-report through May. Summers will be used for data cleaning, preliminary analyses, and for recruiting the next year's cohort.

3.2 Conceptual framework

Incidence, duration and severity of viral ARI illness are influenced by both psychosocial and physiological factors. Sedentary lifestyle, stress, and negative emotions are risk factors for ARI, and may be causal influences. The brain, body, and immune system are highly integrated, with many known and hypothesized mechanisms of action.[177-180] Psychological factors, apparently working through neuro-immune and inflammatory pathways, are associated with various measures of viral ARI.[181-188] These potential pathways, combined with possible interactions and feedback loops, provide for a conceptual framework of almost limitless complexity. To simplify this complexity we will focus on verifiable ARI-related health outcomes and a few select physical, mental, and social health domains.

Figure 2 – Potential causal pathways

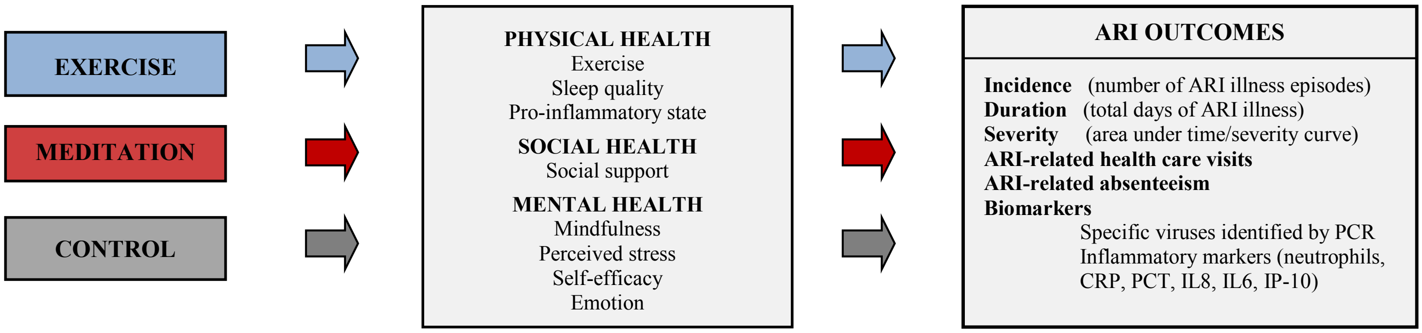

Hypothesized pathways leading from the proposed behavioral interventions to acute respiratory infection (ARI) outcomes

3.3 Multidisciplinary Research Team

Co-Investigators and Consultants for the proposed study have extensive expertise and experience relevant to this proposal. **Bruce Barrett MD PhD** led the first MEPARI study, developed and validated the Wisconsin Upper Respiratory Symptom Survey[173;176;189] and has extensively researched patient-oriented outcomes of respiratory infections.[174;175;190-193] **Roger Brown PhD** has worked with Dr. Barrett for over a decade, and is a world class statistician and methodologist.[194-197] **Christopher Coe PhD** is a highly regarded researcher and scholar of psychoneuroimmunology and behavioral medicine.[198-200] **Richard Davidson PhD** is a well-known, leading expert on meditation and its physiological correlates.[201-206] **James Gern MD** is an expert in respiratory immunology,[207-213]and has developed a highly regarded PCR multiplex array for viral identification.[36] **Mary Hayney PharmD** is an experienced researcher of psychological and immunological aspects of influenza.[214-220] **David Nieman DrPH FACSM** is a leading expert in the effects of exercise on immunity and respiratory infections.[154;163;164;168;221;222] **Daniel Muller MD PhD** is an expert in psychoneuro-immunology and has extensive experience with clinical trials, including meditation.[30;219;223-225] A study by Drs. **Davidson** and **Muller** showed that training in meditation increased pre-frontal brain activation and improved psychological and immune-related outcomes.[30] Work by Drs. **Hayney** and **Muller** reported associations of psychological and interpersonal health indicators with immune biomarkers such as IFN-γ and IL-10.[226] **David Rakel MD** is a leading expert on integrative medicine, and heads the UW Integrative Medicine Program, where mindfulness trainings occur.[227-232] **Aleksandra Zgierska MD PhD** is supported by an NIH K23 grant. She works on mindfulness meditation for alcohol and drug disorders,[233;234] and has reported corresponding changes in psychology self-report and IL-6 levels in alcoholics after 8-week trainings in MBSR.[235] **Zhengjun Zhang PhD** is expert in several areas of advanced statistical methodology, with major strengths in the biostatistics of clinical research.[192;193;236-239] This team obtained funding (1R01AT004313) from the National Center for Complementary and Alternative Medicine (NCCAM) at the National Institutes of Health, conducted the preliminary study during the 2009-2010 cold and flu season, with results publishedJuly 9 2012.[1]

3.4 Primary Study Endpoints

The primary goal of this project is to determine whether behavioral training in mindfulness meditation or moderate intensity sustained exercise can lead to reductions in acute respiratory infection (ARI) illness, such as common cold and influenza like illness. The primary outcome will be severity-weighted total days of ARI illness (global severity), calculated as trapezoidal approximation to area under the time severity curve during ARI illness, with severity assessed once daily using self-reports on the validated Wisconsin Upper Respiratory Symptom Survey (WURSS-24).

3.5 Secondary Study Endpoints

Computer-assisted weekly monitoring will assure that ARI illness episodes are detected, and will serve to document secondary outcomes, including health care utilization and work or school absenteeism. Visits to health care facilities and time lost from work and school will be documented, then classified as ARI-related (or not) by personnel blinded to allocation. Questionnaire measures assessing perceived stress, self-efficacy, sleep quality, depression, and general mental and physical health will also be analyzed as secondary outcomes. Degree of stress reduction, mindfulness, positive and negative emotion, social support, self-efficacy and sleep quality will be analyzed as potential mediators of effects of interventions on outcomes.

Laboratory assessed objective measures will primarily serve to corroborate self-reports of disease severity, but will also be analyzed as potential mediators of effects of behavioral interventions on ARI illness incidence, duration, and severity. As potential mediators, pro-inflammatory cytokines (CRP, PCT, IL-6, IL-8, IP-10) will be assessed as change from baseline to one month after the 8 week behavioral interventions finish. These will serve as indicators of a pro-inflammatory state. [Procalitonin assays stopped in 2014 due to nondetectable values] Repeating these assays 3 months later will assess whether pro-inflammatory changes from baseline will be sustained. Cytokines from samples taken during ARI illness will be assessed as corroborating biomarkers of disease severity. Identification of viral agents using multiplex PCR will also serve to corroborate ARI self-reports.

4. Sample selection

This study aims to assess potential ARI-preventive effects of mindfulness or exercise in a general working age population sample of people who sometimes get colds. People with previous mindfulness training and those with substantive exercise practices are excluded, as are those with immune conditions or who take certain medications. Additional inclusion and exclusion criteria are aimed at making sure that those entering the study are able and willing to follow through with the various aspects of the protocol.

4.1 Inclusion/ Exclusion Criteria

Table 2 Inclusion and exclusion criteria for target sample population

Inclusion Criteria	Exclusion Criteria
1) 30-69 years old	1) Current/recent regular meditation practice or prior training
2) Report ≥ 2 colds in past 12 months, or ≥ 1 cold per year on average	2) Exercising moderately ≥ 2 or vigorously ≥ 1 time per week (CDC/BRFSS classification system)[240]
3) Meets the American Heart Association guidelines for exercise[241-243]	3) Participant is pregnant or plans to become pregnant
4) Ability to adhere to study protocol and willingness to be randomized to 1 of 3 groups	4) Physical, medical or mental condition precluding adherence to study protocol –questionable cases will be reviewed by the study physician
5) PHQ-9 depression screen score ≤ 14	5) True contraindication for influenza vaccine, or refusal to accept influenza vaccination
6) Basic English fluency and literacy	6) Current or anticipated use of: antibiotics, antivirals, immunomodulators (e.g., steroids, chemotherapy)
7) Successful completion of run-in period	7) Immune deficiency or autoimmune disease (HIV/AIDS, lupus, rheumatoid arthritis, multiple sclerosis, inflammatory bowel disease)

4.1.1 Inclusion criteria

1) Aged 30-69 years at study entry.

2) Must answer “Yes” to either “Have you had at least 2 colds in the last 12 months?” and/or “On average do you get at least 1 cold per year?”

3) Prospective participants must meet the American Heart Association guidelines[241-243] for suitability for an exercise program. Prospective participants will be advised (but not required) to seek their physicians’ advice before enrollment. Please see 8.2.5 Safety of Exercise.

4) Self-reported ability and willingness to follow through with either exercise or meditation training, or neither, according to randomized allocation, and to participate in blood draws, nasal wash, self-report questionnaires, and weekly monitoring for 9 months.

5) A score of 14 or lower on the PHQ-9 depression screen, self-reported at baseline (entrance to run-in trial).

6) Fluency and literacy in English language sufficient for understanding the study protocol and completing questionnaires.

7) Successful completion of tasks during run-in period, including 2 in-person appointments, 1 phone contact, 1 set of homework questionnaires, and baseline nasal wash and blood draw.

4.1.2 Exclusion Criteria

1) Current or recent use of meditative practice, or previous meditation training. Assessed by answering "Yes" to any of the following questions: Do you meditate on a regular basis? In the last year, have you meditated at least weekly for 2 or more months in a row? Have you ever been trained in meditation? Have you ever been involved in a mindfulness class or mindfulness practice?

2) Potential participants must not engage in moderate exercise more than twice per week or vigorous exercise more than once per week, as assessed by the following questions adapted from the Behavioral Risk Factor Surveillance System (BRFSS) classification[240] system: On average, how many times per week do you engage in moderate recreational activities such as walking, tennis doubles, ballroom dancing, weight training, or similar activities that last at least 20 minutes per occasion? A) Less than 1 time per week; B) 1 time per week; C) 2 times per week; D) 3 times per week; E) $\geq$4 times per week. How many times per week do you engage in vigorous sport and recreational activities such as jogging, swimming, cycling, singles tennis, aerobic dance or other similar activities lasting at least 20 minutes per occasion? A) Less than 1 time per week; B) 1 time per week; C) 2 times per week; D) 3 or more times per week.

3) Women who are pregnant at screening or plan to become pregnant during the course of the study (determined by self-report) will be excluded. Women who become pregnant any time during the course of the trial will not be dropped and will continue to be followed throughout the duration of the study. Please see 8.6 Vulnerable Populations.

4) Physical, medical or mental condition(s) precluding adherence to study protocol. Conditions include: malignant disease (prospective participants' physicians to advise and Dr. Barrett, and/or designee Dr. Rakel or Dr. Muller, to make final decision); and function-impairing psychopathology (prospective participants' psychiatrist or psychologist to advise). Questionable cases will be reviewed by the study physicians.

5) True contraindication for influenza vaccine (flu shots) or refusal to accept influenza vaccine. Subjects will be asked to verify they have a) no known egg allergy, b) no prior reaction to influenza vaccine, and c) never been told they have Guillain-Barre Syndrome.

6) Current use or forecasted need for immunoactive drugs (eg. steroids, immunosuppressants, chemotherapy); nonsteroidal antiinflammatories will be allowed.

7) Immune deficiency or auto-immune disease (eg. HIV/AIDS, lupus, rheumatoid arthritis, multiple sclerosis, inflammatory bowel disease). Co-Investigator Dr. Muller will advise in questionable cases.

4.2 Participant Recruitment and Screening

4.2.1 Retention Our experience suggests that >90% of participants will complete the intervention training, follow-up, and provide primary and secondary outcome measures. Our research team has the capability and experience to recruit, enroll and monitor the proposed number of participants (n=396 over 5 years), with a retention rate of ≥ 90% (n=360 retained). A previous NCCAM-funded trial led by Dr. Barrett enrolled 719 and retained 713 people.[237;244] The preliminary trial on which this proposal is based enrolled 154 and retained 149 people.[1]

4.2.2 Study promotion will include flyers, posters, and brochures, posted in medical settings and in the community (e.g., through media such as newspaper, radio, press releases, website, email), and mailings of post cards and letters.

4.2.3 Screening Prospective participants who passed phone screening will be met in-person for informed consent and enrollment in a 2-week run-in trial. Screening will continue year-round, with main emphasis on the period from June to August. Prospective participants will call the advertised study phone number to be screened by study personnel using scripted protocol. Potentially eligible and willing, interested participants will be scheduled for a run-in trial.

4.2.4 Run-in trial for adherence assessment Prospective participants who passed phone screening will be met in-person for informed consent and enrollment in a 2-week run-in trial. Run-in tasks will include in-person baseline questionnaires, at least one phone contact, self-report questionnaires at home, and finally a follow-up in-person appointment, which in most cases will serve as the consent visit for the main trial, and which will include a nasal wash and blood draw. Based on our preliminary study, we expect that approximately 70-75% of the eligible run-in participants will consent and be enrolled in the main trial.

4.3 Data Collection and Follow-up for Withdrawn Subjects

Intention-to-treat (ITT) analysis methods will be used to take account of all randomized participants, including those lost to follow-up. The ITT approach: 1) preserves the effects of randomization, and 2) addresses the practical impact of interventions better than "per protocol" analyses. Our approach to ITT will involve random-effects pattern-mixture modeling, where participants will be divided into groups depending on their missing-data patterns. First, analysis of missing data will assess the level and possible reasons for missingness.[245] Second, modeling of data patterns will be used to adjust the longitudinal intervention analysis using the pattern-mixture modeling approach.[246]

5. Study Interventions

5.1 Study Arms

5.1.1 Mindfulness meditation Training will consist of a standardized 8-week Mindfulness Based Stress Reduction (MBSR) program, including 2½ hour weekly sessions and regular at-home daily practice.[132] The MBSR program guides participants from body sensation awareness (body scan), through stretching and breathing to sitting (and lying down) meditation. Didactic sessions center on awareness of physical, emotional, cognitive, and interpersonal responses to stress. In addition to weekly group sessions at U.W. Research Park, participants are asked to practice at home for 45 minutes per day, and to log minutes of daily practice time. A half day meditation "retreat" on a weekend day at the end of week 6 will allow participants to practice their skills. After the 8-week intervention, participants will be asked to continue meditation at ≥150 minutes/week, in sessions of at least 10 minutes each. They will record their practice daily on a paper log and will enter their practice minutes once weekly into an on-line survey. The Mindfulness-based Self Efficacy Scale (MSES)[247] and MAAS[248;249] will serve as additional measures of mindfulness and practice. The MBSR intervention will be delivered by trained instructors, all with extensive experience teaching MBSR at the UW Integrative Medicine Program. Since 1994, more than 2,000 people have completed MBSR training through this program. Katherine Bonus, MA, is the MBSR Program Director and is expected be one of the meditation instructors for our study. The UW MBSR program has demonstrated excellent retention and adherence in both general enrollees and research-based samples. In a sample of 505 general enrollees, more than 86% completed the full 8 weeks and reported significant decreases in both medical and psychological symptoms, comparable to effect sizes noted in a meta-analysis of MBSR trials.[250] In a trial of MBSR among women with fibromyalgia led by co-investigators Muller and Coe, 34 of 42 participants who received MBSR training continued meditation practice after 1½ years of follow-up. See Appendix A.

5.1.2 Exercise The exercise program will match the meditation program in duration (8 weeks), attention (weekly 2½ hour group sessions), intensity (daily 45 minute at-home practice), and at the U.W. Research Park location. See Appendix A. The proposed intervention structure is consistent with many standardized exercise programs.[242;251;252] Participants randomized to exercise who are deemed "high-risk" based on the American Heart Association guidelines will undergo ECG-monitored exercise testing to assess safety (see "safety of exercise" in protection of human subjects section). They will record their exercise daily on a paper log and will enter their exercise minutes once weekly into an on-line survey. Exercise training will primarily focus on walking or jogging, activities that are convenient, easy to teach and do not require special equipment. Individualized programs will be developed for those who have access to specific equipment, are unable to do walking/jogging, or prefer different types of exercise (e.g. biking, swimming, etc). Based on 2008 guidelines, the target will be sustained moderate intensity exercise for at least 150 minutes per week.[242;243] Borg's Rating of Perceived Exertion (RPE) will be used to guide and monitor exercise intensity, using a target RPE level of 12 to 16 points.[253;254] In addition to exercise logs documenting minutes-per-week of moderate and strenuous exercise, participants will complete the Global Physical Activity Questionnaire (GPAQ) at baseline, at the end of the 8-week trainings, and every other month thereafter.[255;256] Each weekly exercise session will include 1½ hours of didactic and 1 hour of group exercise in the U.W. Research Park Sports Medicine Fitness Center. The didactic portion will consist of a check-in period to review the previous week's activities, a brief presentation on exercise techniques and effects, a discussion of behavioral change principles and strategies, and a brief wrap-up focused on discussing the next-week exercising goals and logistics.

A half day exercise retreat designed to match the meditation retreat will occur the weekend of week 6. The retreat will include didactics, group discussion and activities, and individualized exercise practice. After the 8-week intervention, participants will be expected to continue moderate intensity exercise at ≥150 minutes/week, in sessions of at least 10 minutes each.

5.1.3 Wait-list control At enrollment, one third of participants will be randomly assigned to a usual care wait-list non-interventional control group. Apart from not attending any of the specific meditation or exercise training sessions, those in the control group will be treated in essentially the same manner as "experimental" participants. This will include email/telephone contact every week and monthly questionnaires. Control participants will not complete daily logs of exercise or meditation during the study period; however, they will fill out MAAS, MSES, ESES and the GPAQ questionnaires on an every other month basis. Control participants will be offered free meditation training after they complete their study participation, assistance with finding appropriate exercise training, or $300 cash. They will be reminded of this occasionally throughout the study to help maintain adherence to protocol.

5.2 Randomization

Randomized allocation will provide each participant a 33.3% chance of assignment to each of the 3 study arms. Each sequentially enrolled participant will receive the next sequentially numbered sealed envelope containing randomization codes generated by the study statistician using permuted variable-sized block randomization (SAS software). The key linking codes to intervention category will be kept blinded from co-investigators, study personnel and analysts until after the first stages of statistical analysis have been completed.

5.3 Blinding and Expectation

Investigators, most study personnel and analysts will be blinded to allocation status until the compilation of the first stages of statistical analysis. However, study participants cannot be blinded to the group status. Potential reporting bias will be minimized by framing the study in an expectancy-neutral manner, and by assessing and controlling for expectancies toward meditation and exercise. Prior to and following randomization, participants will be asked to rate their belief in the ability of meditation and exercise to protect against ARI using pre-tested expectancy-related questions.[257 258] See Appendix B. Participants will be instructed and regularly reminded to self-report in an unbiased manner and not to reveal their group status to the data collection staff.

5.4 Participant Compliance Monitoring

5.4.1 Weekly monitoring and adherence assessment Each participant will be assigned a study representative who will serve as his/her primary contact, to build rapport and enhance protocol adherence. The primary contact cannot feasibly be blinded to allocation. To minimize potential bias, we will employ blinded-to-allocation personnel to assist participants with documenting and classifying ARI illness, sick days, and clinic visits. For most subjects, weekly computer-assisted self-reports will be used to assess ARI illness. For those without internet access, weekly telephone calls will be scheduled. As soon as an ARI illness episode is verified by criteria (See 6.3.1 below), the participant will self-administer a nasal swab, and arrange a clinic visit where nasal wash will be obtained. During ARI episodes, participants will fill out respiratory infection daily logs (RIDLs), including both WURSS-24 self-assessments and questions documenting health care utilization and days lost to work.

5.4.2 Exercise and meditation practice monitoring will be accomplished using paper daily practice logs, which will be filled out and/or entered on-line once weekly. See Appendices A, B and E. Similar daily practice logs have been used in research studies by Dr. Davidson at the University of Wisconsin and by Dr. Nieman at Appalachian State University. These data will verify that those assigned to trainings actually practice, and will facilitate dose-response, mediation, and sub-group analyses.

6. Study Measurements & Procedures

6.1 Study Participant Overview

A total of 99 participants will be randomized to each yearly cohort (33 participants per intervention group). Over 4 years, 396 participants will be randomized (132 per group). Assuming 9% loss to follow-up, 360 (120 in each of the 3 groups) will complete the protocol and provide main outcome data. Before each yearly cohort begins, an adherence-assessing run-in phase will take place. Based on the preliminary trial, we estimate that 490 will need to be screened and 137 entered in the run-in trial to achieve the 99 people consented into each yearly cohort. Nasal wash and venous blood will be drawn at baseline, one month after 8-week sessions, and 3 months later, and tested for IL-6 and IL-8. Questionnaires assessing psychosocial domains and mental and physical health will be completed at baseline, at the end of the 8-week intervention and then monthly. Blood will be tested for serum concentration of procalcitonin, C-reactive protein, IP-10 and other inflammatory biomarkers. [Procalcitonin assays were dropped in 2014 due to all nondetectable values] Participants will be monitored for ARI illness throughout the cold and flu season (approximately 8 months), using a web-based weekly reporting system. Participants who have not logged on within 8 days to report whether they had any ARI symptoms will be sent daily email reminders until they do so. If 10 days have elapsed since last report, participants will be called by study personnel. For those who do not have access to the web, data will be collected on paper, and weekly contacts will be made by telephone. Each ARI illness episode will be assessed by daily self-report on respiratory infection daily logs (RIDLs) that include the validated WURSS-24 questionnaire.[173;176;189] At the beginning of each ARI illness episode, participants will self-swab both nostrils and arrange a lab visit to collect nasal wash and blood (within 72 hours of first symptom). Both nasal swab and wash will be tested with PCR multiplex for viral identification.

In order to standardize ARI risk across our sample, we will require and provide conventional seasonal trivalent inactivated influenza vaccine to all study participants. This will be given the week following the end of the 8-week sessions.

6.2 Project Timeline

This will be a 5-year project, with 4 yearly cohorts of n=99 persons randomized into 3 groups of n=33 each. Recruitment will start upon UW Institutional Review Board (IRB) approval and continue until study goals are reached. Real time data inspection, verification and cleaning will allow rapid analysis and dissemination of results. The planned project timeline and a timeline of each year cohort are depicted below.

Figure 3 Study Timelines

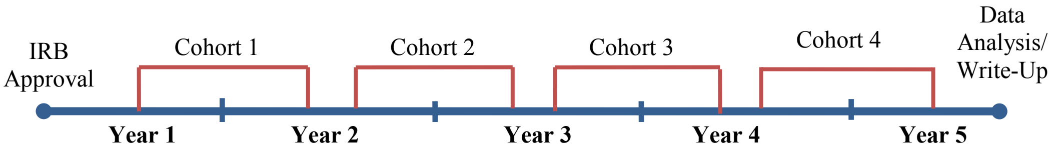

Yearly cohort timeline

June	July	Aug	Sept	Oct	Nov	Dec	Jan	Feb	Mar	Apr	May
Recruitment		Run-in, Baseline BD, NW	Mindfulness, exercise behavioral training		Flu shot	BD, NW			BD, NW		Exit
		Consent									

BD=Blood Draw
NW=Nasal Wash

ARI surveillance and monitoring --→
Monthly self-reported psychosocial outcomes ---→

6.3 Measures of ARI illness

6.3.1 Definition of ARI illness The beginning of each ARI illness episode will be defined by: 1) answering "Yes" to either: "Do you think you have a cold" or "Do you think you are coming down with a cold?" AND 2) reporting at least 1 of 4 cold symptoms or synonyms: nasal discharge (runny nose); nasal obstruction (plugged or congested); sneezing; or sore (scratchy) throat, AND 3) scoring at least 2 points on the Jackson scale. The Jackson score is calculated by summing 8 symptom scores (sneezing, headache, malaise, chilliness, nasal discharge, nasal obstruction, sore throat and cough) rated , 0=absent, 1=mild, 2=moderate, and 3=severe.[170-172] In order for these symptoms to be classified as an ARI illness episode (and analyzed as such), at least 2 days in a row must meet these criteria. From the first day of ARI illness and forward each participant will fill out a daily WURSS-24 until they answer "No" to the question "Do you think that you are still sick with this respiratory infection?" for 2 days in a row. The last day the participant answers "Yes" will be the last day classified as ARI illness and included in the calculation of severity-weighted days of ARI illness.

6.3.2 WURSS-24 - The Wisconsin Upper Respiratory Symptom Survey (WURSS) is a validated questionnaire evaluating ARI-related symptom severity and quality of life impact.[259] Since WURSS was first developed by Dr. Barrett and colleagues in 2002,[189] more than 125 institutions in 37 countries have used the WURSS in their research. Initially, a 44-item version (WURSS-44) was assessed for reliability, responsiveness and importance to patients.[173] Psychometric analyses guided item reduction to yield the WURSS-21 which has been independently validated.[176] The WURSS-24 includes all WURSS-21 questions and 3 additional items assessing fever, headache and body aches - symptoms characteristic of influenza-like illness.[260] Appendix B has questionnaires. Appendix C describes scoring.

6.3.3 RIDL - Participants will be provided with Respiratory Infection Daily Log (RIDL) questionnaire booklets at enrollment, and will be instructed on appropriate use. Weekly computer-assisted monitoring will remind people to be on the lookout for cold or flu symptoms. When cold symptoms appear, participants will call study personnel, who will determine whether the symptoms meet the Jackson criteria described above. If so, participants will begin to fill out WURSS-24 questions in the RIDL booklets and answer questions about healthcare utilization and missed work days, will self-swab their nostrils, and will make an appointment at the laboratory for nasal wash and blood sample collection.

6.3.4 Viral identification will be done in Dr. Gern's lab, where high-throughput PCR-based multiplex methods have been developed and authenticated, and are able to identify nearly all of the pathogens associated with ARI illness [36;261-264] See Appendix D for lab protocols. In our preliminary trial, only 40 of 79 nasal wash samples yielded positive viral identifications. To enhance yield, the proposed trial will assess 2 samples, one done by self-swab at home, and the other by nasal wash at lab. We will also improve sample processing and include newly developed viral types. Dr. Gern's published data report that up to 91.4% of nasal washes from community-acquired ARI can yield positive viral IDs.[265]

6.3.5 Pro-inflammatory cytokines Laboratory-assessed objective measures will primarily serve to corroborate self-reports of disease severity. C-reactive protein (CRP) and procalcitonin (PCT) are well-established indicators of disease severity during respiratory infection, and can be measured in serum as well as in nasal wash.[113;115;118-120] Concentrations of interleukin-6 (IL-6)[266-271] and interleukin-8 (IL-8)[272-276] in nasal wash have been shown to correlate with illness severity. More recently, interferon-gamma-induced protein 10 (IP-10) has been shown to be measurably increased in both serum and nasal wash during times of acute viral ARI.[122-128] Inflammatory cytokines will be measured by ELISA methods in laboratories directed by Dr. Coe and Dr. Hayney. See Appendix D.

6.3.6 Inflammatory tendency The same array of pro-inflammatory cytokines will also be analyzed as indicators of low level inflammation or pro-inflammatory tendency and as potential mediators of effects of behavioral interventions on ARI illness incidence, duration, and severity. The importance of CRP, PCT and IL-6 has been underscored by the ability of these pro-inflammatory biomarkers to predict mortality.[277-285] As potential mediators, pro-inflammatory cytokines (CRP, PCT, IL-6, IL-8, IP-10) will be assessed as change from baseline to one month after the 8 week behavioral interventions finish. [Procalcitonin assays were stopped in 2014 due to nondetectable values] Repeating these assays 3 months later will assess whether potential pro-inflammatory changes resulting from interventions will be sustained.

6.3.7 Polymorphonuclear neutrophil count in nasal mucus is a relatively well-established indicator of inflammation of the nasal epithelium.[286-290] Neutrophil counts correlate to symptom severity, viral titer and cytokine levels.[174;291] Neutrophil count has been done by our study team more than a thousand times. The University of Wisconsin Hospital and Clinics (UWHC) Central Lab successfully conducted neutrophil counts for our preliminary trial. A standardized protocol has been established for their lab. Neutrophil counts will be done on nasal wash collected during ARI episodes. See Appendix D.

6.3.8 Glycosylated hemoglobin (HgA1C) Regular exercise is known to reduce hemoglobin A1C, a widely accepted indicator of average blood glucose levels.[142;292-294] There are at least two preliminary reports suggesting that mindfulness meditation might reduce HgA1C.[295;296] To explore these possibilities, we will assess HgA1C at baseline, 1 month after interventions, and again 3 months later.

6.4 Self-reported physical, social and mental health measures (see Table 3 below for schedule of use)

6.4.1 Alcohol and tobacco use Tobacco use is associated with depressed immune function and increased rate and severity of acute respiratory infection (ARI).[297] Overuse of alcohol also appears to be associated with immune system depression and increased levels of ARI illness.[297] To assess and monitor both tobacco and alcohol use, we will use the validated and widely used Timeline Followback method (TLFB).[298-302] We will consider tobacco use and alcohol overuse as secondary outcomes of potential importance. Baseline tobacco use will be used as a covariate in multivariate efficacy analyses.

6.4.2 Body Mass Index (BMI) Body habitus is associated with many disease processes, and may be related to immune function and susceptibility to respiratory infection. Height will be assessed at baseline only. Weight will be measured at baseline, 1 and 4 months post-intervention, and at exit. Baseline BMI will be calculated and used as a covariate in statistical models. BMI will also be considered a secondary outcome of potential importance.

6.4.3 Demographic indicators Socioeconomic status is related to health and disease, including incidence and severity of respiratory infection.[102;303;304] Demographic indicators to be assessed will include age, sex, years of education completed, household income, and number of children under the age of 18 living in the home. Age, sex and education will be used as covariates in multivariate efficacy analyses.

6.4.4 Seattle Index of Comorbidity (SIC) People with diabetes, cardiovascular disease and pulmonary disease are known to have increased risks when infected with influenza or other respiratory viruses.[305-307] The SIC is a simple 8-item measure shown to predict hospitalization and mortality.[308] We will add items on allergy and asthma, as these illnesses are known to be related to severity of ARI. The modified SIC will be assessed at baseline and exit, and used as a covariate to control for possible influences of chronic disease on ARI outcomes. Relationships of individual items to outcomes and to other co-variates will be explored.

6.4.5 Big Five Inventory (BFI) Research on personality and health has been underway for some time, leading to various conceptual structures of state and trait psychological domains.[309-312] The Big Five taxonomy has helped clarify and organize the links between personality, health behaviors, illness and mortality across the lifespan.[313-315] Of the five dimensions measured (openness, conscientiousness, extraversion, agreeableness, and neuroticism), we will use baseline "conscientiousness" and "neuroticism" scores on the Big Five Inventory[316] to gauge propensity for self-report bias on instrument completion activities, and to control for between person differences in multivariate efficacy models.

6.4.6 Health care utilization and **antibiotics prescribed** Evaluation and treatment of ARI illness is very costly and often associated with unnecessary prescriptions, especially antibiotics. For this study, we plan to document total number of health care visits, ARI-related health care visits, and ARI-related prescriptions, including antibiotics. Each weekly communication will include the question, "Have you seen a doctor or visited a clinic, hospital or urgent care center?" Persons answering "Yes" will be asked the reason for the visit. Those answers will then be classified by study personnel as either "Related," or "Unrelated" to ARI illness, including upper respiratory infection, influenza, pharyngitis, acute sinusitis, bronchitis, and pneumonia. All questionable cases will be verified by inspection of medical records (with case-specific participant permission). Prescriptions for antibiotics, prescription cough medicines, influenza antivirals and other ARI medications will be documented, as will self-reported use of nonprescription medications such as analgesics, antihistamines, decongestants, cough suppressants, and expectorants.

6.4.7 Days of work and school missed to illness Weekly interviews will ask about work or school missed, and the reasons for that absenteeism. Missed work/school will be quantified to the nearest half-day, and will be categorized as "Related" or "Unrelated" to ARI illness. This will be done by asking people their reasons for missing work, and by comparing to Jackson criteria and WURSS-24 scores. Medical records will be inspected as necessary. However, we will not attempt to verify self-reported absenteeism by work logs or communication with supervisors. The research specialist who monitors and responds to weekly computer-assisted monitoring of ARI illness, classifying health care utilization, and missed school/work will be blinded to experimental group status.

6.4.8 Economic outcomes assessed will include costs associated with employment (absenteeism, presenteeism), health care utilization, and medications used during ARI illness. Income will be assessed at the hourly wage level for each participant using standard methods used by the Bureau of Labor Statistics.[317] Time lost to work (absenteeism) and costs of health care utilization will be assessed weekly, and classified as ARI-related or not, using refined methods piloted in the first MEPARI trial. Costs due to absenteeism will be assessed for each person using hourly wage and self-reported absenteeism. Costs of ARI-related health care visits will be determined using U.W. Health's billing database, seeking charges for ARI-coded encounters (ICD-9 codes 460, 461.1, 461.2, 461.8, 461.9, 462, 463, 465.9, 466, 480.9, 487.1, 487.8, and 490). Costs of medications named by participants will be calculated using data from drugstore.com, with cross-checking of prices in local pharmacies. All of these determinations will be done by research personnel blinded to group allocation.

6.4.9 Absenteeism/Presenteeism At enrollment we will assess employment, including type of work, hours per week worked, and compensation, assessed as hourly wage. Each week we will ask about any missed work, ascertain number of hours missed, and assess and classify reasons for missing work as either ARI-related or not ARI-related. The person making the classification will be blinded to allocation. Beyond missed work (absenteeism), illnesses such as ARI can decrease energy and focus at work,[260] leading to lost work productivity[318] from reduced "presenteeism."[319] To refine economic impact analysis, we will assess self-reported ability to perform work using the Stanford Presenteeism Scale (StPS).[320;321] The standard 1-month recall version will be administered at baseline, then 1, 3 and 5 months after interventions. A modified version with illness-specific recall will be used at end of each ARI episode, at the end of the RIDL questionnaire booklet (see 6.3.3 above).

6.4.10 Depression screen (PHQ-9) The PHQ-9 is a widely used and well-validated depression screen,[322-327] and also demonstrates good responsiveness.[328;329] In our study, prospective participants with PHQ-9 scores of ≥15 will be excluded (and referred to appropriate clinical care). PHQ-9 scores will be assessed as secondary outcomes.

6.4.11 Health-related quality of life (SF-12) Also known as the Medical Outcomes Study Short Form, this 12-item questionnaire is commonly used to measure overall health, including physical (SF12-P) and mental health (SF12-M) subscales. It has been extensively assessed for reliability, responsiveness and criterion validity.[330-334] In our study, it will be used to assess potential changes in general physical and mental health due to interventions, and as a covariate to control for baseline between-person differences in multivariate efficacy analyses.

6.4.12 Pittsburgh Sleep Quality Index (PSQI) Sleep quality has been linked to several important quality of life and health outcomes. The PSQI is widely used and has been assessed for reliability and validity.[335-337] In this study, improved sleep is a potential mediator of intervention effects, and a potentially important outcome on its own.

6.4.13 Positive and Negative Affect Schedule (PANAS) The widely used PANAS scale reliably assesses both positive and negative affect (emotion).[338] Self-reported positive and negative emotion have long been known to be independent predictors of psychological and physical health.[339] In the ARI setting, positive and negative emotion predict not only symptom expression, but actual infection as indicated by viral shedding.[9;13;340] In an RCT setting, PANAS scores improved after MBSR training, ($p < 0.05$) as compared to controls.[30]

6.4.14 Perceived Stress Scale (PSS-10) The PSS-10 has been validated in multiple studies.[100-103;341-343] PSS scores predict rates of viral infection among volunteers inoculated with rhinovirus, and correlate with physiologic and self-report indicators of ARI illness, including nasal IL-6 level.[14;99-102] Because stress reduction is one of the hypothesized mechanisms of action, we have expanded our study population to include working-age participants, who we presume are more stressed.

6.4.15 Social Provisions Scale (SPS) [344-346] assesses perceived social support, which has been linked with a host of health and illness indicators.[347-352] The SPS is a 24-item index assessing 6 domains of social health: attachment, social integration, reassurance of worth, reliable alliance, guidance, and opportunity for nurturance. The SPS, developed by Russell and Cutrona,[344-346] predicts both immunological[353;354] and psychosocial outcomes.[355;356]

6.4.16 Social Network Index (SNI) will serve to quantify social network size in order to help characterize social support. Cohen's research using the SNI suggests that the number of social contacts is predictive of susceptibility to ARI.[10;357] The SNI will also serve as an index of interpersonal contacts that could serve to transmit ARI virus. The SNI will be modified to document the number and ages of the children with whom participants have contact.

6.4.17 Feeling Loved (FL) In addition to the validated perceived social support measures described above, we will use two novel questions with Yes/No response options: A) Do you feel loved? B) Do you love yourself? and two questions with visual analogue (VAS) response scales: How loved do you feel? How much do you love yourself? The ends of each 100mm VAS scale will be bounded by "not at all" and "very, very much."

6.4.18 Exercise Self Efficacy (ESES) Self-efficacy has been defined as "the belief in one's capabilities to organize and execute the courses of action required to manage prospective situations."[358] The ESES scale was developed based on work by Bandura and colleagues,[359-363] and has been validated by Shin,[364;365] Kroll,[366] and Everett.[363] For our study, the ESES will be used to verify results of the exercise intervention, and to help explain potential mediational effects of exercise.

6.4.19 Mindfulness-based Self-Efficacy Scale (MSES) Research aimed at defining and assessing the concept of "mindfulness" is well underway, with several questionnaire instruments available.[367-374] The MSES is one of the more recent questionnaires, developed by Cayoun and Freestun to assess effects of MBSR training on perceived self-efficacy.[247] The MSES assesses 7 domains related to mindfulness self-efficacy, including behavior, cognition, interoception, affect, interpersonal, avoidance and mindfulness. The MSES will provide a nice counterpart to the ESES to help distinguish effects of interventions on ARI outcomes.

6.4.20 Mindfulness Attention Awareness Scale (MAAS) For our study, we will use the 15-item MAAS[248;249] to assess effects of MBSR training, and to help understand/explain potential mediating influences of mindfulness on our major outcomes. The MSES and MAAS instruments will also serve as an intervention check, in that scores are expected to change more among those randomized to meditation than in the exercise or control groups.

6.4.21 Global Physical Activity Questionnaire (GPAQ) The GPAQ was developed and validated through the World Health Organization, and displays excellent reliability and responsiveness characteristics.[255;256] GPAQ scores will be used to assess degree-of-change resulting from exercise training, and for dose-dependency and mediation analyses.

6.4.22 Mindfulness practice and exercise daily tracking log After the 8-week intervention, participants assigned to the meditation group will be asked to continue meditation at ≥150 minutes/week, in sessions of at least 10 minutes each. Similarly, those assigned to the exercise group will be asked to continue moderate intensity exercise at ≥150 minutes/week, in sessions of at least 10 minutes each. Using modified versions of practice logs developed at the University of Wisconsin by Dr. Davidson (meditation) and at Appalachian State by Dr. Niemann (exercise), study participants will record their practice once daily on a paper log and will enter their practice minutes once weekly through an on-line web-based data collection portal.

6.4.23 Expectancy In order to assess and potentially control for intervention-related expectancy, we will ask participants about their attitudes towards meditation and exercise before and after randomization, after the 8-week behavioral trainings, and at exit. See Appendix B.

6.4.24 Blood pressure Blood pressure is a well-recognized health indicator. There is some reason to believe that stress reduction or regular exercise might reduce blood pressure. In this study, blood pressure will be assessed at baseline and at both standardized follow-up periods using standard calibrated sphygmomanometers. Blood pressure will be analyzed as a secondary outcome using methods described in Section 7.

6.4.25 Accelerometry & Breath-counting The final 4th cohort will include an optional sub-study in which willing participants will do breath-counting assessment and wear an accelerometer at baseline and at two post-intervention follow-ups. See Appendices I & J.

6.5 Timeline for Baseline Measures, Covariates and Outcomes

Table 3 Timeline for baseline measures, covariates and outcomes

Domain	Measure	1°	2°	M	C	T0	T1	T2	T3	T4	T5	T6	T7	ARI
Primary outcome = Acute respiratory infection (ARI)	ARI surveillance (weekly)	X					X	X	X	X	X	X	X	X
	AUC global severity	X					X	X	X	X	X	X	X	X
	Total days of ARI illness	X					X	X	X	X	X	X	X	X
Economic impact is major secondary outcome	ARI-related clinic visits		X			X	X	X	X	X	X	X	X	X
	Work/school absenteeism		X			X	X	X	X	X	X	X	X	X
	Presenteeism (StPS)		X			X		X		X		X		X
	Viral identification		X											X
Biomarkers	Interleukin 6		X	X		X		X			X			X
	Interleukin 8		X	X		X		X			X			X
	IFN-induced Protein 10		X	X		X		X			X			X
	C-reactive Protein		X	X		X		X			X			X
	Procalcitonin [dropped 2014][		X	X		X		X			X			X
	Neutrophil			X										X
	HgA1C		X			X		X			X			
	Blood pressure		X			X		X			X			
Perceived stress	PSS-10		X	X		X	X		X		X		X	
Self-efficacy	ESE; MSES		X	X		X		X		X		X		
Sleep quality	PSQI		X	X		X	X		X		X		X	
Mindfulness	MAAS			X		X	X		X		X		X	
Positive emotion	PANAS			X		X		X		X		X		
Negative emotion	PANAS			X		X		X		X		X		
Social support	SPS			X		X	X		X		X		X	
Social contacts	SNI			X		X		X		X		X		
Feeling loved	FL			X		X		X		X		X		
Depression	PHQ-9		X			X		X		X		X		
Exercise	GPAQ			X		X	X		X		X		X	
MM EX practice	Weekly surveillance			X			X	X	X	X	X	X	X	
Physical health	SF-12 (P)		X		X	X	X		X		X		X	
Mental health	SF-12 (M)		X		X	X	X		X		X		X	
Comorbidity	Seattle index				X	X							X	
Body type	Height/Weight (BMI)		X		X	X		X			X		X	
Smoking	Timeline followback		X		X	X	X		X		X		X	
Alcohol	Timeline followback		X			X	X		X		X		X	
Personality	Big Five Inventory (BFI)				X	X							X	
Expectancy	Thinking ahead					X	X						X	
Demographics	Age/sex/education/ income				X	X							X	
**Domain**	**Measure**	**1°**	**2°**	**M**	**C**	**T0**	**T1**	**T2**	**T3**	**T4**	**T5**	**T6**	**T7**	

Computer-assisted weekly surveillance will: 1) seek/assess acute respiratory infection (ARI) episodes, 2) monitor exercise and mindfulness meditation practice, and 3) assess for indicators of economic impact (health care visits, absenteeism.) Self-reported outcomes will be assessed at baseline and then post-intervention following the schedule above.

Biological samples (nasal wash, blood) will be assessed at baseline, 1 month after interventions, and 3-months later.

T0 = Baseline, consent visit, before randomization

T1 = Time 1. First week after end of 8-week sessions. T2 = one month after 8 week interventions finish

T1, T2, T3, T4, T5, T6, T7 = Monthly self-assessments

T7 = Final self-assessment and exit interview.

ARI = Measures to be assessed during each illness episode

1° = Primary outcome = global severity (area-under-time-severity-curve for all days of ARI illness)

2° = Secondary outcomes

C = Covariates measured at baseline to be used in primary efficacy statistical models to control for between-person variability

M = Covariates to be analyzed as potential mediators

BMI = body mass index

See Appendix B for copies of questionnaire instruments and Appendix C for scoring information

6.6 Written Informed Consent

Written informed consent will be obtained in person from all participants. Separate written consent will be prepared for the run-in trial and for the main study. Enrollment interviews, informed consent, and all subsequent interactions will be conducted in private settings, with only study staff and the research participant present. All data related to participation will be kept confidential. Paper and electronic records with personally-identifying information will be destroyed at the end of the study. Participants will have the opportunity to give us permission to keep their name and contact them about future research opportunities. See Section 8 and Appendix F for additional details of human subjects' protection.

6.7 Study Visit Checklists

Study visit checklists have been developed for the current study as tools used to ensure consistency and help study coordinators make sure all scheduled assessments/events are done for each particular study visit/time point. Please see Appendix E for a complete list of study visit checklists.

6.8 Clinical Research Unit (CRU)

6.8.1 CRU Overview Supported by the NIH through a Clinical and Translational Science Award (CTSA), the CRU's mission is to offer an optimal setting for investigators to conduct safe, controlled state-of-the-art research. The Center has provided excellent services to the research community of the UW. It is centrally located, with free parking for participants, and is easily accessed by car or bus. The CRU, located within the UW Hospital and Clinics, provides personnel, supplies and equipment support necessary to perform high quality clinical research. The CRU recently expanded to 18 inpatient and outpatient rooms in its 6th floor center with a core staff of highly-trained research nurses and a sample processing facility. The CRU is the hub for federally-funded, investigator-initiated, and industry studies that need the in-hospital location, safety and expertise of the 15-member RN, BSN, and Nurse Practitioner staff.

6.8.2 Nursing Services CRU nurses will conduct nasal wash and blood draw for the baseline, 1 and 4 month post-intervention visits. Services include obtaining height/weight for BMI calculation, conducting nasal washes and blood draws and preparing samples to send to the UWHC Central Lab and Hayney lab for processing, and administering the influenza vaccination. A detailed list of services provided is in the CRU application, as well as the Nasal Lavage Information Sheet and Nasal Wash Labwork Information Sheet. See Appendix E.

6.8.3 Admission to CRU Admission to the CRU requires all study participants to be registered with the UW Hospital and Clinics (UWHC). All screened, eligible research subjects will be registered with UWHC per UWHC registration requirements prior to being scheduled at the CRU using a Reservation Request Form. Please see Appendix E for detailed Admission and Billing Procedures.

6.8.4 Billing: Office of Clinical Trials (OCT) For federally funded and other investigator-initiated studies, nursing services and rooms at the CRU are provided at no cost to Institute for Clinical and Translational Research (ICTR) members, including Dr. Barrett and some co-investigators. Additionally, while there are no fees for nursing services and rooms at the CRU, ancillary services (including laboratory samples processed at the UW Core Lab) are awarded at $33 per patient per CRU visit, not to exceed $8,500 annually per research study. As ancillary services may exceed the award, billing procedures are coordinated through the institution's Office of Clinical Trials (OCT).

6.8.5 Physician's Orders Standard physician's orders are provided to the CRU nursing staff for all research study visits. Orders ensure all research participant's receive the same assessments for each visit.

6.9 Pharmaceutical Research Center (PRC)

6.9.1 Influenza Vaccination The PRC reviews study feasibility, prepares budget estimates, and manages clinical research drug distribution. All clinical drug research protocols within UW Hospital and Clinics, including the CRU, must be coordinated through the PRC. For this study PRC will prepare influenza vaccine syringes and deliver to the CRU for administration. Influenza vaccination will be administered at the CRU by registered nurses. Please see Appendix E for PRC Protocol Summary.

7. Statistical Plan

7.1 Sample Size Determination

The planned study size of n=396 consenting individuals randomized to 3 groups leading to n=360 people completing the protocol (n=120 per group) is based on the results of our preliminary trial.[1] For primary efficacy analyses, null hypotheses will be rejected if interventions are superior to control at a $p \leq 0.025$, using one-sided testing, based on two-way contrasts between: 1) meditation vs. control and 2) exercise vs. control. One-sided testing is supported by our own data and by available scientific literature. Given these parameters and our preliminary data, the sample size of n=120 per group should provide adequate power. The observed raw difference in area-under-curve global severity between the meditation and control groups was 59.9% yielding a standardized effect size of 0.55. For the exercise vs. control, the raw difference was 30.5% and the effect size 0.20. Comparing exercise to meditation, the standardized effect size was 0.24. Based on these data, Figure 4 below shows the relationship of power to sample size for the global severity and duration primary outcomes, for each of the specified main contrasts. However, when taking into account the zero-inflated and skewed nature of ARI illness data, and potential missing data and intention to treat considerations, actual power may be less. Given limitations on resources available, and a desire to minimize chances of both Type 1 and Type 2 errors, we feel that the proposed sample size will be adequate for this phase 2 randomized controlled human subjects clinical trial.

Figure 4

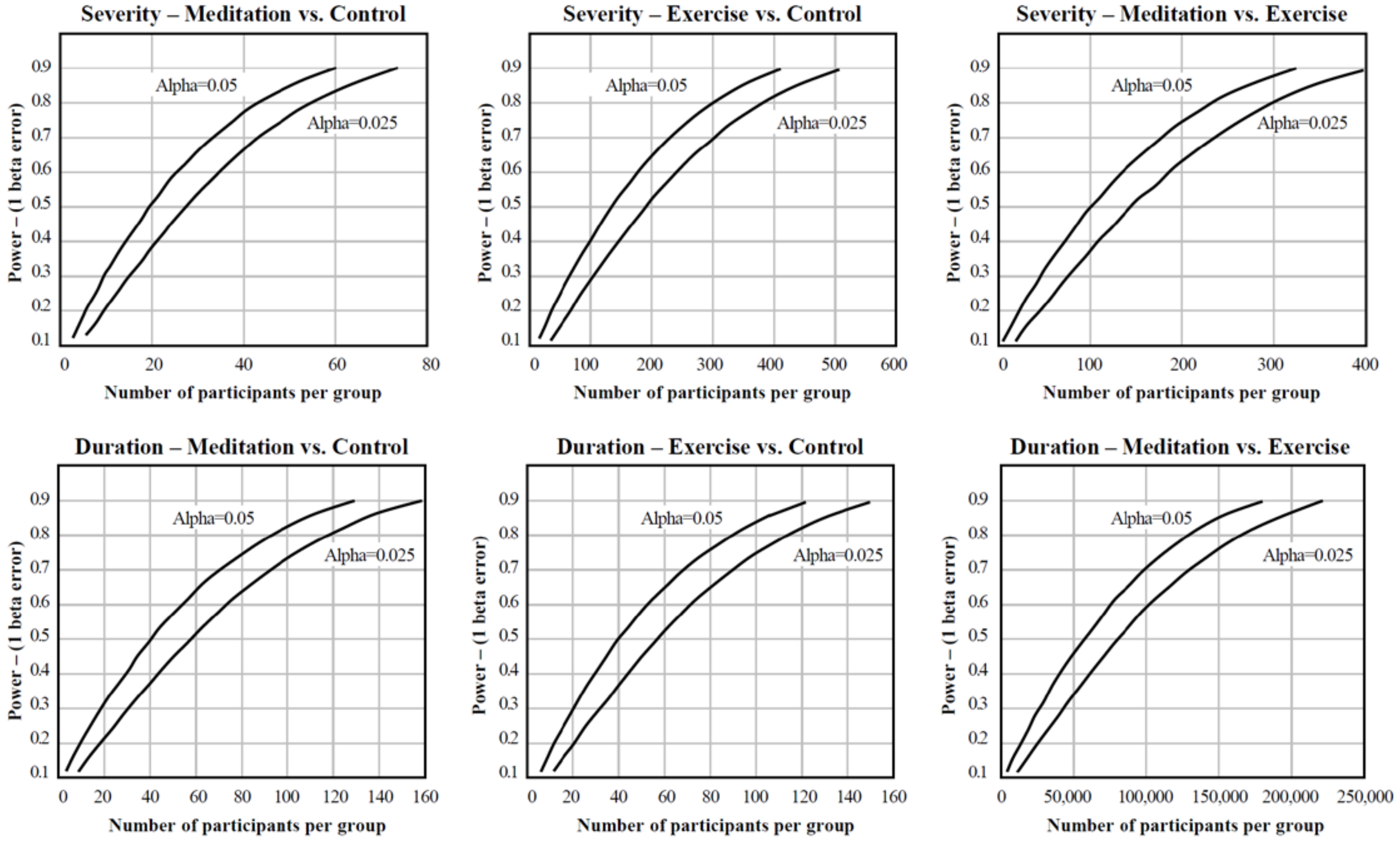

These power graphs are generated from data gathered during the first MEPARI trial, and do not take into account minimal important difference considerations. Additional data on power available in Appendix H.

7.2 Statistical Methods

7.2.1 Loss to follow-up In Dr. Barrett's first echinacea trial, 96% of enrolled participants were substantively adherent to protocol.[375] In our recently published NCCAM-sponsored trial (1R01AT001428) testing echinacea, placebo, and doctor-patient-interaction,[376] only 6 of the N=719 people enrolled (<1%) withdrew or were lost to follow-up.[244] In the preliminary trial described above, we maintained a 97% retention rate (5 of the 154 participants withdrew). For the proposed study, we conservatively estimate a 9% dropout rate, yielding N=90 finishers per year. Each year, we expect to screen 490 people and enroll 137 in the run-in trial in order to achieve 99 entered in the main trial and 90 finishers. For the full 4 year trial, this equates to 1,960 screened, 548 entered in the run-in phase, and 396 randomized.

7.2.2 Analysis overview Analyses will proceed as follows: 1) descriptive analyses of central tendency and dispersion for all variables; 2) assessment and potential response for missing data; 3) bivariate and multivariate analyses to assess relationships among variables; 4) calculation of ARI global severity for individual participants; 5) primary efficacy analysis of effects of interventions on primary outcomes; and 6) secondary analyses, including assessment of intervention impact on secondary outcomes, longitudinal analyses, and process (mediation) analyses.

7.2.3 Descriptive analyses A necessary first step in analysis is to describe the data in a way that is both concise and meaningful. For each variable, a 5-number summary (mean, median, standard deviation, standard error, skewness) and a graphical box plot will be constructed. Bivariate relationships will be explored using scatter plots and correlation coefficients. Distributional assumptions will be tested, and variable transformations will be applied if necessary. Missing data will be analyzed for the level and possible reasons for missingness. Where appropriate, imputation methods may be applied. These decisions and preliminary statistical analyses will be done prior to unblinding.

7.2.4 Global severity Area-under-curve ARI global severity days for each participant will be calculated using daily WURSS-24 scores across all days of ARI illness. Daily WURSS scores are calculated by summing items #2 through #23 on the WURSS-24. Duration is assessed in hours and minutes, then converted to decimalized days. Area-under-the-curve is calculated using trapezoidal approximation, where the X-axis = duration and the Y-axis = severity.

7.2.5 Primary efficacy analysis will contrast total days of illness and area-under-curve global severity in each intervention group with corresponding values in the control group. Unadjusted contrasts will be done by t-test, using variable transformation if skewness requires. Adjusted analyses will be based on zero-inflated regression models taking into account the episodic and variable nature of ARI illness.[377;378] Zero-inflated regression models incorporate a logistic sub-model to account for people without ARI illness, and a linear sub-model to account for the variability in continuous measures of severity and duration. These will employ conservative estimates with standard error inflated using Huber/White maximum likelihood estimation.[379;380] Co-variates to be controlled for in this model will include: age, sex, body mass index, smoking status, comorbidity, highest level of education achieved, neuroticism, conscientiousness, general physical health and general mental health. The null hypotheses of "no effect on primary outcome" will be rejected in favor of alternative hypothesis of "some effect" if p-values are ≤0.025. Confidence intervals will be constructed for all outcome variables, allowing estimation of effect size. Zero-inflated regression models for primary efficacy analyses will be done using M-Plus version 6.1or similar software. See Appendix H for additional details.

7.2.6 Secondary efficacy analyses Influence of interventions on secondary outcomes will be assessed using ANOVA-based multivariate regression models using SAS software.[381-383] Adjustment for multiple comparisons will be incorporated, and interpretation will be cautious.[238;384-388] In general, we will want to see relationships with p<0.01 in order to justify tentative null hypothesis rejection. Pre-planned secondary efficacy analyses will include effects of interventions on: 1) absenteeism, 2) health care utilization, 3) general physical health (SF-12), 4) general mental health (SF-12); 5) perceived stress (PSS-10), 6) self-efficacy (MSES, ESE), 7) sleep quality (PSQI), 8) body weight (BMI), 9) blood pressure and 10) pro-inflammatory cytokines. One-sided testing will be based on the underlying hypotheses that the behavioral trainings lead to improved physical and mental health, sleep quality and self-efficacy, and to reductions in absenteeism, health care utilization, stress, body weight, blood pressure and proinflammatory cytokines. Specific hypotheses related to these secondary analyses can be stated as: **7.2.6.1** Those in the intervention groups will display reduced absenteeism, as compared to control. **7.2.6.2** Those in the intervention groups will display reduced health care utilization, as compared to control. **7.2.6.3** Those in the intervention groups will display improved physical health, as compared to control. **7.2.6.4** Those in the intervention groups will display improved mental health, as

compared to control. **7.2.6.5** Those in the intervention groups will display reduced perceived stress, as compared to control. **7.2.6.6** Those in the intervention groups will display improved self-efficacy, as compared to control. **7.2.6.7** Those in the intervention groups will display improved sleep quality, as compared to control. **7.2.6.8** Those in the intervention groups will display reduced BMI, as compared to control. **7.2.6.9**Those in the intervention groups will have lower blood pressure compared to control. **7.2.6.10** Those in the intervention groups will have lower cytokine levels compared to control, both at standardized follow-up, and during ARI episodes.

7.2.7 Subgroup analyses We will, in general, consider sub-group or other exploratory analyses to be hypothesis-generating. However, pre-planned secondary efficacy analyses will be conducted on the following sub-groups: 1) those who attend at least 7 of the 8 weekly training sessions, 2) those who continue to exercise or meditate for an average of at least 60 minutes per week after the trainings end, 3) those who at baseline have perceived stress scores at least 14 points (PSS-10). One-sided testing will be based on the underlying hypotheses that: A) behavioral trainings are more likely to work for those who attend classes, and B) the interventions will be more effective for more highly stressed people.

7.2.8 Longitudinal analysis Several of our secondary outcomes will be measured at multiple time points. Analysis of longitudinal data is complicated by including dependencies among successive observations made on the same individual.[389-391] To address these difficulties, we will employ a general linear mixed model for repeated measures using various covariance structures (e.g., autoregressive one, compound symmetry, etc.) for error terms for continuously measured secondary outcomes.[392] For discrete measures (binary or count data), a Generalized Estimating Equations (GEEs) approach[393-398] will provide a practical method with reasonable statistical efficiency to analyze such data.[393;394] We expect to use SAS version 9.0 or higher software for these secondary analyses.

7.2.9 Intention-to-treat (ITT) analysis methods will be used to take account of all randomized participants, including those lost to follow-up. The ITT approach: 1) preserves the effects of randomization, and 2) addresses the practical impact of interventions better than "per protocol" analyses. Our approach to ITT will involve two strategies. First, for sporadically missing primary outcome WURSS-24 data within ARI episodes, expectancy-maximizing multiple imputation methods will be used for data that meets missing at random criteria.[399] Secondly, to take account of those lost to follow-up, we will proportionally randomize non-completers to logistic and linear portions of the ZIM model, with imputation based on within-group data for the linear sub-model. All imputation or other data manipulation will be done by analysts blinded to allocation group status. See Appendix H.

7.2.10 Process analysis (mediation) Intervention research is often confounded by 2 types of variables - background variables and mediating process variables.[197;400] We are always concerned about controlling confounding background variables in an intervention study, and we should be equally concerned about the variables that make up the process of the intervention--i.e., the mediating conditions. In mediation analysis, we do not wish to control mediating variables as much as we wish to estimate their contribution to the final outcome of the program. An analysis that encompasses the mediational process (i.e., the theory of intervention) should mirror the actual program and provide information concerning its strengths and weaknesses. This analysis would subsequently provide another level of information regarding the possible "successes" and/or "failures" of the behavioral interventions, and provide guidance for further development and enhancement. For this study, we will follow principles and methodologies outlined by Judd,[401] Co-Investigator Brown,[197] and Yuan.[402] See Appendix H for additional details regarding process analysis.

7.2.11 Economic analyses will focus on costs associated with employment, health care utilization, and medications used during ARI illness episodes. See sections C.6.4.6 through C.6.4.9 above. Because economic data such as these tend to be non-normally distributed and can lead to erroneous results, we will employ a Monte Carlo probability model using bootstrap sampling with replacement.[403] This will enhance estimation of variability and hence better precision and confidence. Clinic visit charges (Level 2, 3 or 4) will be based on a Dirichlet distribution. Medication costs will also employ distributional sampling, as we trust subjects to report which medications they used, but not to be able to accurately estimate costs or dosages over time. Wages lost to missed work will be individually-specific, and hence not require bootstrap sampling. Economic models will be further refined using estimates of lost work productivity derived from data self-reported on the Stanford Presenteeism Scale[320;321] administered to participants who go to work during ARI episodes.

7.2.12 Absenteeism/Presenteeism At enrollment we will assess employment, including type of work, hours per week worked, and compensation, assessed as hourly wage. Each week we will ask about any missed work, ascertain number of

hours missed, and assess and classify reasons for missing work as either ARI-related or not ARI-related. To refine economic impact analysis, we will assess self-reported performance using the **Stanford Presenteeism Scale**.[320;321] Presenteeism will be considered a secondary outcome, and will be used to adjust economic impact in exploratory models.

8. Protection of Human Subjects

8.1 Overview This proposed study will be submitted to and approved by the Human Subjects Committee of the Health Sciences Institutional Review Board of the University of Wisconsin – Madison (UW HS-IRB), and will be in compliance with HIPAA and all other federally mandated human subjects regulations.[404] All members of the research team will complete the protection of human subjects' tutorial required by the UW HS-IRB's Human Subjects Committee prior to the study's initiation. All named UW Co-Investigators, Consultants, and current Project Personnel have in fact already completed this tutorial. All study personnel, including research assistants, will be trained in confidentiality, informed consent procedures, and other aspects of human subject protection.

The UW HS-IRB has approved several human subjects' studies with Dr. Barrett as PI. Previously approved studies include a randomized trial of echinacea,[375] an NIH-NCCAM-R01 trial testing echinacea, placebo, and doctor-patient interaction,[244;376;405] several studies developing and validating the Wisconsin Upper Respiratory Symptom Survey.[173-176;259] The most recently complete MEPARI trial on which this proposal is based was approved and monitored by UW HS-IRB. Dr. Barrett has been a voting member of UW HS-IRB since 2006, but would be recused from voting on this proposal.

Participation in the proposed study will be completely voluntary. Participants will be informed of their right to refuse to participate or to withdraw at any time for any reason. Prisoners, pregnant women and mentally impaired persons will not be included. Competent persons aged 30 to 69 meeting other eligibility criteria will be welcomed, regardless of gender, ethnicity, religion or socioeconomic status. Participants will be encouraged to ask any questions at any time for any reason, and to seek outside counsel when appropriate. Participants will be told that this study in no way substitutes for medical care, and that they should contact their health care provider if they feel any need to do so, for any reason related or unrelated to the study. Participants will also be encouraged (but not required) to consult their primary care physician prior to enrollment in this study.

8.2 Risk and Benefits

8.2.1 Risks and Benefits Summary Apart from flu shots, hand-washing, smoke avoidance, and maintenance of general health, there are no known safe and effective prevention strategies for acute viral respiratory infection. Interventions to be tested are low risk, and more likely to confer benefit than harm. Influenza vaccination (flu shots) will be provided free of charge to all participants, and could be considered a benefit. Participants will not be required to forego any proven or accepted preventions or treatments, and in fact will be encouraged to seek appropriate medical attention for any condition, including respiratory infection, and to continue their regular health care practices. Antibiotics, anti-influenza antivirals, and other ARI treatments will not be disallowed, but instead will be tracked as secondary outcomes. In general, participants will be asked to continue the study interventions they are assigned, and to forego the study interventions they are not assigned. However, they will be informed that they remain free to make their own health care, behavioral and life style decisions. In particular, exercise will not be discouraged for participants in the meditation or wait-list control group, as exercise is known to confer some health benefit. However, we will ask that participants in the exercise and control group refrain from starting a meditation program, and that all participants refrain from starting any purportedly immune-modifying supplements (eg, echinacea, ginseng, zinc, elderberry, etc.), unless advised to do so by their physicians. If any participants do choose to actively begin an intervention they were not assigned (i.e. a person randomized to exercise chooses to take the mindfulness meditation course instead), we will ask them to continue in the study, and will use that "cross-over" information in secondary analyses using the principles outlined in Section 4.3 of the proposal.

8.2.2 Risks and benefits to society As mentioned above, apart from flu shots, hand-washing, and maintenance of general health, there are no known safe and effective prevention strategies for acute viral respiratory infection. Results of the preliminary trial suggest that substantive public health benefit could results from the proposed behavioral training interventions. Regardless of whether the proposed trial confirms these findings, the research will yield useful data for health scientists, and perhaps for clinicians, policy makers, and citizens. Apart from potential lost opportunity costs in

funding this research instead of other proposals, we cannot identify any significant risks to public health. Instead, we feel that the overall societal benefit risk ratio is clearly positive.

8.2.3 Adverse effects There were no adverse effects seen during the preliminary trial. Neither the UW HS-IRB nor the Data and Safety Monitoring Committee (DSMC) felt that there was a need to monitor and test for any specific potential adverse effects. Nevertheless, potential adverse effects will be monitored in the proposed research. We will ask about any exercise-related injuries or accidents. Using open-ended questions, we will ask about possible or potential adverse effects of either meditation or exercise. Evidence regarding potential adverse effects will be reviewed at regular bi-weekly Co-Investigator team meetings, and at DSMC meetings. Any reported potential adverse effect categorized as "moderate" or "severe" will be reviewed immediately by at least one of the study's physicians, who will decide whether to immediately contact the DSMC or IRB (per their reporting requirements).

8.2.4 Safety of trivalent inactivated influenza vaccine Influenza vaccination has a clearly advantageous benefit-risk profile, with approximately 100 million doses of influenza vaccine administered annually in the U.S.[57;61-64] Risks associated with influenza immunization include discomfort at the injection site, which occurs in up to 60% of people getting flu shots. Guillain-Barré Syndrome has been temporally associated with influenza immunization with an estimated incidence of 1-2 cases per million doses of vaccine. Theoretically, all vaccines carry the very remote risk of anaphylaxis. Nevertheless, nearly all competent and respected sources agree that flu shot benefits greatly outweigh potential harms.

8.2.5 Safety of exercise The broad body of literature strongly suggests that it is safer to exercise than to not exercise, for nearly all people in nearly all disease and risk categories.[142-145;406;407] Nevertheless, it is possible that progressing too rapidly in an exercise program could carry morbidity or even mortality risks, including musculoskeletal injury and potential cardiovascular complications. Therefore, we will follow American Heart Association approved guidelines for suitability for an exercise program,[241-243] and will suggest (but not mandate) that prospective participants seek their physician's advice before enrollment. All participants with a diagnosis of coronary heart disease (including history of infarction, angina, acute coronary syndrome, or congestive heart failure), stroke, or peripheral vascular disease who are randomized to exercise will undergo a submaximal treadmill exercise test with ECG monitoring prior to participation. Those with diabetes and ≥ 2 other risk factors (dyslipidemia, HTN, smoking, family history of cardiovascular event [heart attack, stroke] prior to age 55), will also undergo electrically monitored exercise sessions. This exercise stress test will be used primarily for developing an appropriate and safe individually-tailored exercise program. Anyone showing ST segment changes consistent with acute myocardial ischemia will be referred to appropriate medical care in a timely manner. Results of this sub-max stress testing will be made available to study participants' physicians upon request and with permission. In general, exercise intensity for this study will be in the moderate to somewhat vigorous range (50-70% aerobic capacity based on estimated maximal heart rate), which has been shown to be safe and effective for health maintenance. Emergency equipment and trained personnel are available at the UW Sports Medicine Fitness Center. Participants will be informed about warning signs and symptoms and when to stop exercising and/or seek medical attention when exercising outside of the training facilities.

8.2.6 Safety of meditation To the best of our knowledge, there are no known significant negative effects in relation to mindfulness meditation. For the purposes of this study, we will assume that mindfulness meditation training carries no significant risks. However, participants assigned to the meditation group will be monitored using the same rigorous methods as everyone else, and if any suggestive evidence of adverse effects emerges, we will consult with study physicians, meditation training staff, DSMC and/or IRB and determine how to proceed.

8.2.7 Adventitious findings None of the study's procedures or measures is designed for clinical diagnosis or treatment. Participants will be told they should not use any aspect of the study for medical or health decision purposes. The most likely "adventitious findings" from this study can be classified as potentially related to 1) depression or other mental health disorders, or 2) abnormal vital signs or chest symptoms potentially related to heart or lung disease, or 3) abnormal lab results. Of all study labs, only HgA1C results will be given to participants. Any potential clinical care or medical decision-making related to HgA1C will be the responsibility if the participant and his/her clinician(s).

Formal screening for depression with the PHQ-9 will be part of the eligibility screening process. Anyone excluded by this test (score of 15 or higher) will receive advice that they should contact a licensed physician, psychiatrist or psychologist. Participants scoring 10 to 14 on the PHQ-9, while eligible for the study, will receive similar advice. The PHQ-9 also asks

respondents to rate themselves as follows: "Over the last two weeks, how often have you been bothered by any of the following problems?" Response range is "not at all", "several days," "more than half the days," and "nearly every day". The last item is "Thinking that you would be better off dead or that you want to hurt yourself in some way." All respondents reporting any response greater than "Not at all" for this item will be given advice to seek help from a physician, a clinical psychologist or a psychiatrist. They will be told that this study does not constitute clinical care in any way, and that the enrolling research specialist does not have training in physical or mental health, but that s/he has been told that anyone reporting anything other than "Not at all" for this item should be advised that this may be a sign of clinical depression, and therefore will be referred to an appropriate health provider. If the situation is urgent, the participant or potential participant will be referred to an urgent care center or emergency department. If the situation is emergent, the research assistant will dial 9-1-1 and/or seek immediate local assistance. Four practicing physicians (Barrett, Rakel, Muller, Zgierska) will share a call schedule for this study, so that at least one will usually be available to guide research specialists or speak with research participants, should the need arise.

For those randomized to the exercise intervention, the following adventitious findings may be observed: abnormal heart rate (pulse), respiratory rate, or blood pressure. Additionally, it is possible that some participants might experience chest pain, pressure or palpitations, and/or abnormal degree of shortness of breath, nausea, or other unexpected or worrisome symptoms. The exercise physiologist will monitor and report any abnormal changes, and may directly contact one of our MDs on staff (Barrett, Rakel, Muller, Zgierska). If the symptoms or signs constitute an emergency, exercise physiology staff will dial 9-1-1 and/or provide immediate aid.

It is theoretically possible the mindfulness practices may evoke psychological responses that will require psychological support. These could occur during a mindfulness training session, during any other session, and at home. The mindfulness teachers are trained in identifying participants who may require psychological support, and after consultation with a study MD, the participant will be referred to additional psychological support.

8.2.8 Nasal wash is a simple, minimal risk, and widely practiced means of obtaining nasal fluid. Participants will be informed of the potential risks of discomfort with the nasal wash, and will be free to refuse these or any other procedures for any reason.

8.2.9 Nasal swabs Self-administered nasal swabs have been used safely and effectively in previous clinical studies.[408] Copan manufactures one of the most commonly used sterile nasal flocked swabs for collecting respiratory virus samples from mucosal surface of nasal epithelium. The swabs have a protective “collar” that keeps the flocked cotton surface from entering more than 4 cm into the nasal cavity. Participants will be provided with verbal, written and illustrated instructions at the baseline visit, and will complete one practice self-swab at the enrollment visit.

8.2.10 Phlebotomy Blood draws are necessary in order to allow us to measure pro-inflammatory cytokines, both from baseline to standardized follow-up, and once during each ARI episode. This will be done by trained and experienced phlebotomists at the Clinical Research Unit in the U.W. Hospitals and Clinics. See section 6.8. Phlebotomy blood draw may cause brief pain, and mild bruising. Tenderness from the blood draws may be present for a few days afterward.

8.2.11 Biologic specimens We plan to keep the biological specimens for up to 5 years after the last participant has exited from the study, and to destroy the code linking specimen to identifiable participant within 2 years of last subject exit.

8.3 Inclusion of Women and Minorities

8.3.1 Women Women will be included in this study, and encouraged to participate. We expect that more than 50% of our sample will be female. The preliminary MEPARI trial had a female participation rate of 82% (122 of 149 participants). Other previous studies directed by Dr. Barrett have had female participation rates in the range of 50% to 80%. We expect similar female participation rates in the proposed study.

8.3.2 Minorities All adults aged 30 to 69 years will be potentially eligible for this study, regardless of race, ethnicity, religion or other social or cultural considerations. Individuals from minority groups will be actively recruited, and will be encouraged to participate. We will strive toward substantial minority representation, and will target a major portion of our advertising budget and promotion activities towards ethnic minority neighborhoods in the greater Madison area.

Specifically, we will advertise in the 2 main newspapers in the Madison area that targeted minority audiences, *The Madison Times* (African American) and *The Voz Latina* (Hispanic/Latino). We will also focus staff-directed efforts towards minority communities. These include mailings, flyers, announcements at community meetings, and talks to community groups. Finally, we plan to connect with parish/church health staff in congregations serving largely minority populations, and to meet with key personnel in organizations such as Centro Hispano of Dane County, The Latino Health Council, the Urban League of Greater Madison, and the Madison Urban Ministry.

In spite of the fact that Madison is an overwhelmingly white community, recruitment techniques used in Dr. Barrett's research have been reasonably successful in the past. For example, the first echinacea trial included 15% non-white participants.[375] Our community-based study to develop the Wisconsin Upper Respiratory Symptom Survey included 16% African Americans, 3% Hispanic/Latino, and 3% Native Americans.[173;189] Our NCCAM-funded R01 trial (Physician Echinacea Placebo, PEP) enrolled 63 African Americans, 22 Hispanics, and 16 Native Americans. While the preliminary MEPARI trial had only 8 minority participants, we expect that the proposed trial will do better, as the preliminary trial restricted participation to those aged ≥50. The age range of 30 to 69 should result in higher minority participation rates.

8.4 Inclusion of Children

This study is targeted to women and men aged 30 to 69 years, and will not include children. Our choice of age range to include is based on several considerations. From a public health standpoint, interventions that can potentially enhance resistance and prevent or ameliorate ARI illness should be directed toward – and tested in – populations that could receive the most benefit. Because of the cost-effectiveness component, working-aged adults are an obvious choice. Secondly, the interventions to be used have not been developed for – or tested among – children. While mindfulness meditation training and moderate intensity exercise activities such as regular brisk walking may indeed be immune modulating and health-enhancing for children, there is very little experience and data to rely on in this area. Our research team, including meditation and exercise trainers, has a proven track record recruiting and retaining adult participants. Including adults aged 30-69 will provide a sample population more likely to be economically productive, and susceptible to ARI illness. Likewise, they are likely to adhere to the interventions and to benefit from them.

8.5 Data and Safety Monitoring Plan

A Data and Safety Monitoring Committee (DSMC) will oversee human subjects recruitment and monitoring, and will function independently of the principal investigator and co-investigators. We expect that this committee will function similarly to that established for the first MEPARI trial, and will choose to meet once or twice yearly to monitor recruitment and retention, and to provide oversight and guidance in relation to safety, bioethics, and other human subjects concerns that may arise . The DSMC for MEPARI-2 will consist of four UW faculty: 1) Paul Hutson PharmD (pharmacy, clinical research); Nasia Safdar MD (infectious disease), 3) Tom Cook PhD (biostatistics), and 4) Margo Hoover-Regan MD (pediatric oncology, clinical trials). See Appendix F for full Data Safety and Monitoring Plan. Study progress and safety will be reviewed during bi-weekly co-investigator meetings (more frequently if needed). Progress reports, including patient recruitment, retention/attrition, and AEs will be provided to at the end of each 8-week intervention and then again at the conclusion of each yearly cohort. While there are no "expected" adverse events in this trial, any "unexpected" adverse events will be monitored in a timely fashion.

8.6 Vulnerable Populations

The proposed study will not enroll pregnant women, human fetuses and neonates, children, prisoners, or any other individual considered vulnerable by NIH or IRB standards. Current pregnancy or plan to become pregnant during the course of the study will be excluded by self-report. Women who become pregnant any time during the course of the trial will not be dropped and will continue to be followed throughout the duration of the study. Children are excluded because of the nature of the mindfulness and exercise interventions, and because one focus of the study is to assess potential benefits in terms of reduced workplace absenteeism. The rationale for excluding women who are pregnant or plan to become pregnant is two-fold: 1) pregnancy may influence the immune system and/or susceptibility to respiratory infection, and 2) pregnancy, labor and childbirth might interfere with participants' ability to adhere to the study protocol.

8.6.1 Pregnancy Current pregnancy or plan to become pregnant during the course of the study will be excluded by self-report. We do not plan to actively monitor for new pregnancies. We will, however, ask women to let us know if they become pregnant. Women who become pregnant during the study will be invited to continue participating. None of the study activities are deemed to add any significant risk to pregnancy. Exercise and influenza immunization are recommended for pregnant women and those planning to become pregnant. Blood draws, nasal wash, nasal swab and questionnaires do not add significant risk. If and when a pregnancy occurs we will ask whether the participant has a family doctor or obstetrician. If not, we will strive to provide appropriate advice and assistance in locating one. Pregnant women will be encouraged to seek the advice of their clinician(s) in regards to participation in the study.

8.7 Adverse Events (AEs)

There were no adverse effects seen during the preliminary trial. Neither the UW HS-IRB nor the Data and Safety Monitoring Committee (DSMC) felt that there was a need to monitor and test for any specific potential adverse effects. Nevertheless, potential adverse effects will be monitored in the proposed research. We will ask about any exercise-related injuries or accidents. Using open-ended questions, we will ask about possible or potential adverse effects of either meditation or exercise. Evidence regarding potential adverse effects will be reviewed at regular bi-weekly Co-Investigator team meetings, and at DSMC meetings. Any reported potential adverse effect categorized as “moderate” or “severe” will be reviewed immediately by at least 2 of the study’s physicians, who will decide whether to immediately contact the DSMC or IRB (per their reporting requirements).

8.8 Reporting Serious Adverse Events (SAEs)

SAEs that are unanticipated, serious, and/or possibly related to the study intervention will be reported to the Data and Safety Monitoring Committee, HS-IRB, CRU, and NCCAM in accordance with their requirements. Anticipated SAEs or those unrelated to the study intervention will be reported to the same entities in accordance with requirements.

8.9 Protocol Registration

Upon approval by the UW HS-IRB, the Principal Investigator will register the proposed study in ClinicalTrials.gov. Both of Dr. Barrett’s NCCAM-sponsored trials (1R01AT001428 and 1R01AT004313) were registered, with ClinicalTrials.gov registration numbers NCT00065715 and NCT01057771, respectively.

8.10 Importance of the Knowledge to be Gained

The primary goal of this project is to determine whether behavioral trainings in mindfulness meditation or moderate intensity exercise can lead to reductions in acute respiratory infection (ARI) illness, such as common cold and influenza. Our preliminary findings suggest substantial benefit of these two interventions in terms of reduced incidence, duration and severity of ARI illness, with corresponding reductions in days of work lost to illness. If the proposed research confirms these findings, there will be major implications for public and private health-related policy, as well as for scientific knowledge regarding health maintenance and disease prevention.

Acute infection from influenza and other respiratory viruses leads to much human suffering and loss of economic productivity. Our own evidence suggests that training in either meditation or exercise may lead to substantial reductions in ARI disease burden and work absenteeism. In addition to testing whether our findings are replicable in a larger sample with refined methodology, the proposed translational comparative-effectiveness research will investigate mechanisms of action and provide initial estimates of cost-effectiveness. If positive findings are confirmed, this line of research could have direct and immediate impact on public and private health-related policies and clinical practice, as well as on scientific understanding of respiratory infection.

9. Data Handling and Record Keeping

9.1 Confidentiality

9.1.1 Protection of Subject Privacy Screening of potential participants and consent and enrolling of participants will be carried out in a private location, or via telephone. Similarly, collection of biological samples (nasal wash, blood draw) will be done with full privacy. The exercise and meditation interventions, however, will be done in group session, hence should not be considered private. All potential participants will be informed of these aspects of the study.

All personal identifiers will be destroyed once data collection and analysis has been completed. For those who formally agree that we may keep their name and contact information for potential future research opportunities, the code list linking individual to study data will be destroyed.

9.1.2 Database Protection All project data will be kept in password-protected security-ensured databases. Project personnel will be required to enter separate personal logins and passwords both for computer workstations specifically designated for the project and for the database user authentication process. Personally identifiable information (name, address, date of birth, adverse effect reports, etc.) required for longitudinal study participant management will be stored in an encrypted database, on a local hard drive physically separate from the database storing research outcome data. Permission to view study participant information will be available only to the Principal Investigator or his designee, as regulated by the user authentication procedure. Data analysts will be restricted to viewing outcome data, and will not be able to access personal identifiers. The database used to perform confidential study participant tracking and reimbursement auditing functions will be accessible only to the PI and to the involved research specialists, in conformity with UW HS-IRB requirements.

Identifying information will be recorded either on a single detachable sheet of paper, kept in a locked filing cabinet, or in a separate password-protected security database, which will not have direct links to process or outcome data. Project databases will generate an unseen unique identifying code for each participant, separate from the study participant number used on data collection instruments, to connect the participant's multiple data elements. Association of that code with the study participant's identifying information will reside in an encrypted database separate from the study's research data, ensuring that no records in the dataset used for analysis will contain any data that could identify the participant. All computerized databases will be protected with passwords, with access restricted to appropriate study personnel.

9.1.3 Confidentiality During AE Reporting AE reports and annual summaries will not include subject-identifiable material. Each report will have only the study identifier associated with that subject.

9.2 Health Information

By signing the consent form research subjects are giving permission for their health information to be used by and shared with the individuals, companies, or institutions described in the consent form. Unless a subject withdraws permission in writing to stop the use of their health information, there is no end date for its use for this research study. Subjects may withdraw their permission at any time by writing to the person whose name is listed in the consent form.

Beginning on the date a subject withdraws their permission, no new information about them will be used. Any information that was shared before they withdrew their permission will continue to be used. If a subject withdraws their permission, they can no longer actively take part in this research study.

10 Study Finances

10.1 Funding Source

This study is financed through an R01 grant from the U.S. National Institute of Health, National Center for Complementary and Alternative Medicine.

10.2 Conflict of Interest

The University of Wisconsin Madison has legal and ethical responsibilities to review and, where appropriate, to reduce, eliminate, or manage potential financial conflicts of interest in research involving human subjects. To ensure that the safety of research participants is adequately protected, the UW-Madison (1) requires reporting and review of significant financial interests that might present a real or potential conflict of interest with the individual's research prior to final IRB approval of the research, and (2) limits the participation of individuals who hold significant financial interests related to a research project involving human subjects in that research. Further, the UW-Madison affirms that significant financial interests should not negatively affect data sharing, publishing, and mentoring of students, fellows, trainees, or other research workers.

PIs, Co-Is, and key personnel on human subjects protocols reviewed by the HS-IRB have an obligation to report all significant financial interests held by themselves or their immediate families in business entities with financial interests that would reasonably appear to be affected by the conduct or outcome of the human subjects research reviewed by a UW-Madison IRB.

PIs, Co-Is, and key personnel report significant financial interests in Annual Outside Activities Reports and/or in protocol-specific Outside Activities Reports in conjunction with IRB applications, as appropriate.

10.3 Subject Compensation

Participants will be compensated $50 for completing the run-in trial, and $250 for complete participation in the main trial, for a maximum total compensation of $300 per participant, plus free training in exercise or meditation (a $550 value). Only those who complete the run-in trial will be eligible for the main study. Those in the main study who complete the first 8 weeks, including receiving influenza vaccination and getting pre- and post-vaccine antibody titers, but dropping out before the next data collection, will be compensated $100. Those completing the next 3 months of observation (including next blood draw) and then dropping out will receive another $75 ($175 total for taking part in the main trial). Those completing the full 8 months of monitoring will receive the full $250 (plus $50 for the run-in trial). Those who are randomized to usual care will have the opportunity to have their tuition paid for the meditation training for the year after they have exited from the study, or may instead choose to receive an additional check for $300. If those originally assigned to usual care non-interventional control choose a meditation class, they will be assisted in joining groups that are not part of this study, so as to avoid any "contamination" effects.

10.4 Costs to Study Subjects

There will be no costs to research subjects for participating in this research study. As part of their study participation they will receive meditation or exercise (see Subject Compensation above) at no cost to them. Additionally, study visits at the CRU and study laboratory samples will be paid for by the research study.

11. Publication Plan

Bruce Barrett, MD/PhD, is the Principal Investigator (PI) of this research. He wrote the grant proposal, will direct the trial, supervise data collection and analysis, write or direct the main results manuscript upon completion of the study, and will be responsible for interpretation of results. All co-investigators will be meaningfully involved with the main results paper, and will in addition work on other papers to result from this study. All co-authors will contribute substantially and approve any final manuscripts. Both the PI and all co-authors will have full access to all of the study data and take full responsibility for the integrity of the data and the accuracy of the data analysis.

Beyond normal scientific review process, the National Center for Complementary and Alternative Medicine did not and will not contribute substantively to the design or conduct of the study, data collection, management, analysis or interpretation of the data, or preparation, review, or approval of manuscripts.

Reference List

1. Barrett B, Hayney MS, Muller D, Rakel DP, Ward A, Obasi C *et al*. Meditation or exercise for preventing acute respiratory infection: A randomized controlled trial. *Annals of Family Medicine* 2012;**10**:339-46.

2. Campbell H. Acute respiratory infection: A global challenge. *Archives of Disease in Childhood* 1995;**73**:281-3.

3. Fendrick AM, Monto AS, Nightengale B, Sarnes M. The economic burden of non-influenza-related viral respiratory tract infection in the United States. *Archives of Internal Medicine.* 2003;**163**:487-94.

4. File TM. The epidemiology of respiratory tract infections. *Seminars in Respiratory Infections.* 2000;**15**:184-94.

5. Varmus H, Klausner R, Zerhouni E, Acharya T, Daar AS, Singer PA. Grand challenges in global health. *Science* 2003;**302**:398-9.

6. Falagas ME, Mourtzoukou EG, Vardakas KZ. Sex differences in the incidence and severity of respiratory tract infections. *Respir.Med.* 2007;**101**:1845-63.

7. Jennings LC, Anderson TP, Beynon KA, Chua A, Laing RT, Werno AM *et al*. Incidence and characteristics of viral community-acquired pneumonia in adults. *Thorax* 2008;**63**:42-8.

8. Mohren DCL, Swaen GMH, Borm PJA, Bast A, Galama JMD. Psychological job demands as a risk factor for common cold in a Dutch working population. *Journal of Psychosomatic Research* 2001;**50 (2001)**:21-7.

9. Cohen S, Doyle WJ, Skoner DP, Fireman P, Gwaltney JM, Newsom JT. State and trait negative affect as predictors of objective and subjective symptoms of respiratory viral infections. *Journal of Personality and Social Psychology* 1995;**68**:159-69.

10. Cohen S, Doyle WJ, Turner R, Alper CM, Skoner DP. Sociability and susceptibility to the common cold. *Psychological Science.* 2003;**14**:389-95.

11. Enarson DA,.Chretien J. Epidemiology of respiratory infectious diseases. *Current Opinion in Pulmonary Medicine.* 1999;**5**:128-35.

12. Graham NMH. The epidemiology of acute respriatory infections in children and adults: A global perspective. *Epidemiologic Reviews* 1990;**12**:149-77.

13. Cohen S, Doyle WJ, Turner RB, Alper CM, Skoner DP. Emotional style and susceptibility to the common cold. *Psychosomatic Medicine.* 2003;**65**:652-7.

14. Cohen S, Doyle WJ, Skoner DP. Psychological stress, cytokine production, and severity of upper respiratory illness. *Psychosomatic Medicine* 1999;**61**:175-80.

15. Doyle WJ, Gentile DA, Cohen S. Emotional style, nasal cytokines, and illness expression after experimental rhinovirus exposure. *Brain Behav.Immun.* 2006;**20**:175-81.

16. Chubak J, McTiernan A, Sorensen B, Wener MH, Yasui Y, Velasquez M *et al*. Moderate-intensity exercise reduces the incidence of colds among postmenopausal women. *Am J Med* 2006;**119**:937-42.

17. Kohut ML, Lee W, Martin A, Arnston B, Russell DW, Ekkekakis P *et al*. The exercise-induced enhancement of influenza immunity is mediated in part by improvements in psychosocial factors in older adults. *Brain, Behavior, & Immunity* 2005;**19**:357-66.

18. Kohut ML, Cooper MM, Nickolaus MS, Russell DR, Cunnick JE. Exercise and psychosocial factors modulate immunity to influenza vaccine in elderly individuals. *Journals of Gerontology Series A-Biological Sciences & Medical Sciences* 2002;**57**:M557-M562.

19. Nieman DC,.Nehlsen-Cannarella SL. The immune response to exercise. *Seminars in Hematology* 1994;**31**:166-79.

20. Pedersen BK,.Hoffman-Goetz L. Exercise and the immune system: regulation, integration, and adaptation. *Physiological Reviews* 2000;**80**:1055-81.

21. Woods JA, Davis JM, Smith JA, Nieman DC. Exercise and cellular innate immune function. *Medicine & Science in Sports & Exercise* 1999;**31**:57-66.

22. Astin JA. Stress reduction through mindfulness meditation. Effects on psychological symptomatology, sense of control, and spiritual experiences. *Psychother.Psychosom.* 1997;**66**:97-106.

23. Bishop SR. What do we really know about mindfulness-based stress reduction? *Psychosom.Med* 2002;**64**:71-83.

24. Carmody J,.Baer RA. Relationships between mindfulness practice and levels of mindfulness, medical and psychological symptoms and well-being in a mindfulness-based stress reduction program. *J.Behav.Med.* 2008;**31**:23-33.

25. Mars TS,.Abbey H. Mindfulness meditation practise as a healthcare intervention: A systematic review. *International Journal of Osteopathic Medicine* 2010;**13**:56-66.

26. Shapiro SL, Astin JA, Bishop SR, Cordova M. Mindfulness-based stress reduction for health care professionals: results from a randomized trial. *International Journal of Stress Management* 2005;**12**:164-76.

27. Tacon AM, McComb J, Caldera Y, Randolph P. Mindfulness meditation, anxiety reduction, and heart disease: a pilot study. *Fam.Community Health* 2003;**26**:25-33.

28. Carlson LE, Speca M, Faris P, Patel KD. One year pre-post intervention follow-up of psychological, immune, endocrine and blood pressure outcomes of mindfulness-based stress reduction (MBSR) in breast and prostate cancer outpatients. *Brain Behav.Immun.* 2007;**21**:1038-49.

29. Creswell JD, Myers HF, Cole SW, Irwin MR. Mindfulness meditation training effects on CD4+ T lymphocytes in HIV-1 infected adults: a small randomized controlled trial. *Brain Behav.Immun.* 2009;**23**:184-8.

30. Davidson RJ, Kabat-Zinn J, Schumacher J, Rosenkranz M, Muller D, Santorelli SF *et al*. Alterations in brain and immune function produced by mindfulness meditation. *Psychosomatic Medicine* 2003;**65**:654-570.

31. Fan Y, Tang YY, Ma Y, Posner MI. Mucosal immunity modulated by integrative meditation in a dose-dependent fashion. *J.Altern.Complement Med.* 2010;**16**:151-5.

32. Pace TW, Negi LT, Adame DD, Cole SP, Sivilli TI, Brown TD *et al*. Effect of compassion meditation on neuroendocrine, innate immune and behavioral responses to psychosocial stress. *Psychoneuroendocrinology* 2009;**34**:87-98.

33. Robinson FP, Mathews HL, Witek-Janusek L. Psycho-endocrine-immune response to mindfulness-based stress reduction in individuals infected with the human immunodeficiency virus: a quasiexperimental study. *Journal of Alternative & Complementary Medicine* 2003;**9**:683-94.

34. Solberg EE, Halvorsen R, Sundgot-Borgen J, Ingjer F, Holen A. Meditation: a modulator of the immune response to physical stress? A brief report. *British Journal of Sports Medicine* 1995;**29**:255-7.

35. Witek-Janusek L, Albuquerque K, Chroniak KR, Chroniak C, Durazo-Arvizu R, Mathews HL. Effect of mindfulness based stress reduction on immune function, quality of life and coping in women newly diagnosed with early stage breast cancer. *Brain Behav.Immun.* 2008;**22**:969-81.

36. Lee WM, Grindle K, Pappas T, Marshall DJ, Moser MJ, Beaty EL *et al*. High-throughput, sensitive, and accurate multiplex PCR-microsphere flow cytometry system for large-scale comprehensive detection of respiratory viruses. *J.Clin.Microbiol.* 2007;**45**:2626-34.

37. Ebell MH,.Alfonso A. A systematic review of clinical prediction rules for the diagnosis of influenza. *Annals of Family Medicine* 2011;**9**:61-77.

38. Gwaltney JM, Jr., Hendley JO, Simon G, Jordan WS, Jr. Rhinovirus infections in an industrial population. II. Characteristics of illness and antibody response. *JAMA* 1967;**202**:494-500.

39. Hall CE, Cooney MK, Fox JP. The Seattle virus watch. IV. Comparative epidemiologic observations of infections with influenza A and B viruses, 1965-1969, in families with young children. *American Journal of Epidemiology.* 1973;**98**:365-80.

40. Rao SS, Hendley JO, Hayden FG, Gwaltney JM. Symptom expression in natural and experimental rhinovirus colds. *American Journal of Rhinology* 1995;**9**:49-52.

41. Doyle WJ, Skoner DP, White M, Hayden F, Kaplan AP, Kaliner MA *et al*. Pattern of nasal secretions during experimental influenza virus infection. *Rhinology.* 1996;**34**:2-8.

42. Eccles R. Understanding the symptoms of the common cold and influenza. *The Lancet Infectious Diseases* 2005;**5**:718-25.

43. Gwaltney JM. Virology and immunology of the common cold. *Rhinology* 1985;**23**:265-71.

44. Dingle JH, Badger GF, Jordan WS. Illness in the home: A study of 25,000 illnesses in a group of Cleveland families. Cleveland: Press of Western Reserve University, 1964.

45. Monto AS. Viral respiratory infections in the community: Epidemiology, agents, and interventions. *American Journal of Medicine* 1995;**99**:24S-7S.

46. Cooney MK, Hall CE, Fox JP. The Seattle virus watch. III. Evaluation of isolation methods and summary of infections detected by virus isolations. *American Journal of Epidemiology.* 1972;**96**:286-305.

47. Williams JV, Harris PA, Tollefson SJ, Halburnt-Rush LL, Pingsterhaus JM, Edwards KM *et al*. Human metapneumovirus and lower respiratory tract disease in otherwise healthy infants and children. *New England Journal of Medicine.* 2004;**350**:443-50.

48. Rainer TH, Chan PK, Ip M, Lee N, Hui DS, Smit D *et al*. The spectrum of severe acute respiratory syndrome-associated coronavirus infection. *Annals of Internal Medicine.* 2004;**140**:614-9.

49. Bertino JS. Cost burden of viral respiratory infections: issues for formulary decision makers. *American Journal of Medicine.* 2002;**112**:Suppl-49S.

50. Monto AS,.Ullman BM. Acute respiratory illness in an American community. *JAMA* 1974;**227**:164-9.

51. Fox JP, Hall CE, Cooney MK, Luce RE, Kronmal RA. The Seattle virus watch. II. Objectives, study population and its observation, data processing and summary of illnesses. *American Journal of Epidemiology.* 1972;**96**:270-85.

52. Gwaltney JM, Hendley JO, Simon G, Jordan WS, Jr. Rhinovirus infections in an industrial population. I. The occurrence of illness. *New England Journal of Medicine* 1966;**275**:1261-8.

53. Gwaltney JM. Viral respiratory infection therapy: historical perspectives and current trials. *American Journal of Medicine.* 2002;**112**:Suppl-41S.

54. Smith MBH,.Feldman W. Over-the-counter cold medications: A critical review of clinical trials between 1950 and 1991. *JAMA* 1993;**269**:2258-63.

55. Turner RB. New considerations in the treatment and prevention of rhinovirus infections. *Pediatric Annals* 2005;**34**:53-7.

56. Carter JM. Hand washing decreases risk of colds and flu. *Journal of the National Medical Association.* 2002;**94**:A11.

57. Harper SA, Fukuda K, Uyeki TM, Cox NJ, Bridges CB. Prevention and control of influenza: recommendations of the Advisory Committee on Immunization Practices (ACIP). *MMWR Recomm.Rep.* 2004;**53**:1-40.

58. Thompson WW, Shay DK, Weintraub E, Brammer L, Cox N, Anderson LJ *et al*. Mortality associated with influenza and respiratory syncytial virus in the United States. *JAMA.* 2003;**289**:179-86.

59. Thompson WW, Shay DK, Weintraub E, Brammer L, Bridges CB, Cox NJ *et al*. Influenza-associated hospitalizations in the United States. *JAMA* 2004;**292**:1333-40.

60. Goodwin K, Viboud C, Simonsen L. Antibody response to influenza vaccination in the elderly: a quantitative review. *Vaccine* 2006;**24**:1159-69.

61. Advisory Committee on Immunization Practices CfDCaP. Prevention and control of influenza. Recommendations of the Advisory Committee on Immunization Practices (ACIP). *MMWR Morb Mortal Wkly Rep* 2006;**55**:1-41.

62. Monto AS. The clinical efficacy of influenza vaccination. *PharmacoEconomics* 1996;**9**:Suppl-22.

63. Morgan R,.King D. Influenza vaccination in the elderly. *Postgraduate Medical Journal* 1996;**72**:339-42.

64. Postma MJ, Baltussen RM, Heijnen ML, de Berg LT, Jager JC. Pharmacoeconomics of influenza vaccination in the elderly: reviewing the available evidence. *Drugs & Aging* 2000;**17**:217-27.

65. Demicheli V, Jefferson T, Rivetti D, Deeks J. Prevention and early treatment of influenza in healthy adults. *Vaccine* 2000;**18**:957-1030.

66. Treanor J, Keitel W, Belshe R, Campbell J, Schiff G, Zangwill K *et al*. Evaluation of a single dose of half strength inactivated influenza vaccine in healthy adults. *Vaccine* 2002;**20**:1099-105.

67. Wilde JA, McMillan JA, Serwint J, Butta J, O'Riordan MA, Steinhoff MC. Effectiveness of influenza vaccine in health care professionals: a randomized trial. *JAMA* 1999;**281**:908-13.

68. Gross PA, Hermogenes AW, Sacks HS, Lau J, Levandowski RA. The efficacy of influenza vaccine in elderly persons. A meta-analysis and review of the literature. *Annals of Internal Medicine* 1995;**123**:518-27.

69. Gluck R,.Wegmann A. Influenza vaccination in the elderly. *Developmental & Comparative Immunology* 1997;**21**:501-7.

70. Deguchi Y, Takasugi Y, Tatara K. Efficacy of influenza vaccine in the elderly in welfare nursing homes: reduction in risks of mortality and morbidity during an influenza A (H3N2) epidemic. *Journal of Medical Microbiology* 2000;**49**:553-6.

71. Keren G, Segev S, Morag A, Zakay-Rones Z, Barzilai A, Rubinstein E. Failure of influenza vaccination in the aged. *Journal of Medical Virology* 1988;**25**:85-9.

72. Kohut ML,.Senchina DS. Reversing age-associated immunosenescence via exercise. *Exercise Immunology Review* 2004;**10**:6-41.

73. Levine M, Beattie BL, McLean DM, Corman D. Characterization of the immune response to trivalent influenza vaccine in elderly men. *Journal of the American Geriatrics Society* 1987;**35**:609-15.

74. Phair J, Kauffman CA, Bjornson A, Adams L, Linnemann C, Jr. Failure to respond to influenza vaccine in the aged: correlation with B-cell number and function. *Journal of Laboratory & Clinical Medicine* 1978;**92**:822-8.

75. Voordouw AC, Sturkenboom MC, Dieleman JP, Stijnen T, Smith DJ, van der LJ *et al*. Annual revaccination against influenza and mortality risk in community-dwelling elderly persons. *JAMA* 2004;**292**:2089-95.

76. Rozanski A,.Kubzansky LD. Psychologic functioning and physical health: A paradigm of flexibility. *Psychosomatic Medicine* 2005;**67**:S47-S53.

77. Adams JH, Aubert RE, Clark VR. The relationship among John Henryism, hostility, perceived stress, social support, and blood pressure in African-American college students. *Ethnicity & Disease* 1999;**9**:359-68.

78. Freeman ZS. Stress and hypertension--a critical review. *Medical Journal of Australia* 1990;**153**:621-5.

79. Pearlin LI, Schieman S, Fazio EM, Meersman SC. Stress, health, and the life course: some conceptual perspectives. *Journal of Health & Social Behavior* 2005;**46**:205-19.

80. Wallace R,.Wallace D. Structured psychosocial stress and therapeutic failure. *Journal of Biological Systems* 2004;**12**:335-69.

81. Pressman SD,.Cohen S. Does positive affect influence health? *Psychol.Bull.* 2005;**131**:925-71.

82. Hammen C. Stress and depression. *Annu.Rev.Clin.Psychol.* 2005;**1**:293-319.

83. Kessler RC. The effects of stressful life events on depression. *Annu.Rev.Psychol.* 1997;**48**:191-214.

84. Lindeberg SI, Ostergren PO, Lindbladh E. Exhaustion is differentiable from depression and anxiety: Evidence provided by the SF-36 vitality scale. *Stress-the International Journal on the Biology of Stress* 2006;**9**:117-23.

85. Rozanski A, Blumenthal JA, Kaplan J. Impact of psychological factors on the pathogenesis of cardiovascular disease and implications for therapy. *Circulation* 1999;**99**:2192-217.

86. Angerer P, Siebert U, Kothny W, Muhlbauer D, Mudra H, von Schacky C. Impact of social support, cynical hostility and anger expression on progression of coronary atherosclerosis. *Journal of the American College of Cardiology* 2000;**36**:1781-8.

87. Kivimaki M, Leino-Arjas P, Luukkonen R, Riihimaki H, Vahtera J, Kirjonen J. Work stress and risk of cardiovascular mortality: prospective cohort study of industrial employees. *British Medical Journal.* 2002;**325**:857.

88. Krantz DS,.McCeney MK. Effects of psychological and social factors on organic disease: a critical assessment of research on coronary heart disease. *Annual Review of Psychology* 2002;**53**:341-69.

89. Kark JD, Goldman S, Epstein L. Iraqi missile attacks on Israel: The association of mortality with a life-threatening stressor. *Journal of the American Medical Association* 1995;**273**:1208-10.

90. Nielsen NR, Kristensen TS, Schnohr P, Gronbaek M. Perceived stress and cause-specific mortality among men and women: results from a prospective cohort study. *Am.J.Epidemiol.* 2008;**168**:481-91.

91. Roberts RE, Kaplan GA, Camacho TC. Psychological distress and mortality: evidence from the Alameda County Study. *Social Science & Medicine.* 1990;**31**:527-36.

92. Rosengren A, Orth-Gomer K, Wedel H, Wilhelmsen L. Stressful life events, social support, and mortality in men born in 1933. *BMJ* 1993;**307**:1102-5.

93. Antoni MH, Cruess DG, Klimas N, Carrico AW, Maher K, Cruess S *et al*. Increases in a marker of immune system reconstitution are predated by decreases in 24-h urinary cortisol output and depressed mood during a 10-week stress management intervention in symptomatic HIV-infected men. *Journal of Psychosomatic Research* 2005;**58**:3-13.

94. Black PH. Stress and the inflammatory response: a review of neurogenic inflammation. *Brain, Behavior, & Immunity* 2002;**16**:622-53.

95. O'Connor MF, Bower JE, Cho HJ, Creswell JD, Dimitrov S, Hamby ME *et al*. To assess, to control, to exclude: effects of biobehavioral factors on circulating inflammatory markers. *Brain Behav.Immun.* 2009;**23**:887-97.

96. Pace TW, Negi LT, Sivilli TI, Issa MJ, Cole SP, Adame DD *et al*. Innate immune, neuroendocrine and behavioral responses to psychosocial stress do not predict subsequent compassion meditation practice time. *Psychoneuroendocrinology* 2010;**35**:310-5.

97. Cohen S, Miller GE, Rabin BS. Psychological stress and antibody response to immunization: a critical review of the human literature. *Psychosom.Med.* 2001;**63**:7-18.

98. Glaser R, Kiecolt-Glaser JK, Malarkey WB, Sheridan JF. The influence of psychological stress on the immune response to vaccines. *Annals of the New York Academy of Sciences* 1998;**840**:655.

99. Cobb JM,.Steptoe A. Psychosocial stress and susceptibility to upper respiratory tract illness in an adult population sample. *Psychosomatic Medicine* 1996;**58**:404-12.

100. Cohen S, Frank E, Doyle WJ, Skoner DP, Rabin BS, Gwaltney JM. Types of stressors that increase susceptibility to the common cold in healthy adults. *Health Psychology* 1998;**17**:214-23.

101. Cohen S, Tyrell DAJ, Smith AP. Psychological stress and susceptibility to the common cold. *NEJM* 1991;**325**:606-12.

102. Cohen S, Line S, Manuck SB, Rabin BS, Heise ER, Kaplan JR. Chronic social stress, social status, and susceptibility to upper respiratory infections in nonhuman primates. *Psychosomatic Medicine* 1997;**59**:213-21.

103. Cohen S, Hamrick N, Rodriguez MS, Feldman PJ, Rabin BS, Manuck SB. Reactivity and vulnerability to stress-associated risk for upper respiratory illness. *Psychosomatic Medicine.* 2002;**64**:302-10.

104. Glaser R,.Kiecolt-Glaser JK. Stress-associated immune modulation: relevance to viral infections and chronic fatigue syndrome. *American Journal of Medicine* 1998;**105**:35S-42S.

105. Pressman SD, Cohen S, Miller GE, Barkin A, Rabin BS, Treanor JJ. Loneliness, social network size, and immune response to influenza vaccination in college freshmen. *Health Psychol.* 2005;**24**:297-306.

106. Smolderen KG, Vingerhoets AJ, Croon MA, Denollet J. Personality, psychological stress, and self-reported influenza symptomatology. *BMC Public Health* 2007;**7**:339.

107. Stone AA, Bovbjerg DH, Neale JM, Napoli A, Valdimarsdottir H, Cox D *et al*. Development of common cold symptoms following experimental rhinovirus infection is related to prior stressful life events. *Behavioral Medicine* 1992;**18**:115-20.

108. Takkouche B, Regueira C, Gestal-Otero JJ. A cohort study of stress and the common cold. *Epidemiology* 2001;**12**:345-9.

109. Pruessner JC, Hellhammer DH, Kirschbaum C. Burnout, perceived stress, and cortisol responses to awakening. *Psychosom.Med.* 1999;**61**:197-204.

110. van Eck M, Berkhof H, Nicolson N, Sulon J. The effects of perceived stress, traits, mood states, and stressful daily events on salivary cortisol. *Psychosomatic Medicine* 1996;**58**:447-58.

111. Ruiz RJ, Fullerton J, Brown CE, Schoolfield J. Relationships of cortisol, perceived stress, genitourinary infections, and fetal fibronectin to gestational age at birth. *Biol.Res.Nurs.* 2001;**3**:39-48.

112. Christ-Crain M,.Muller B. Procalcitonin and pneumonia: is it a useful marker? *Curr.Infect.Dis.Rep.* 2007;**9**:233-40.

113. Holm A, Pedersen SS, Nexoe J, Obel N, Nielsen LP, Koldkjaer O *et al*. Procalcitonin versus C-reactive protein for predicting pneumonia in adults with lower respiratory tract infection in primary care. *Br.J.Gen.Pract.* 2007;**57**:555-60.

114. Shanely RA, Nieman DC, Henson DA, Jin F, Knab AM, Sha W. Inflammation and oxidative stress are lower in physically fit and active adults. *Scand.J.Med Sci Sports* 2011.

115. Ahn S, Kim WY, Kim SH, Hong S, Lim CM, Koh Y *et al*. Role of procalcitonin and C-reactive protein in differentiation of mixed bacterial infection from 2009 H1N1 viral pneumonia. *Influenza.Other Respi.Viruses.* 2011;**5**:398-403.

116. Cunha BA. Procalcitonin vs clinical and chest film findings to diagnose community-acquired pneumonia in patients with acute asthma or acute exacerbations of chronic bronchitis. *Chest* 2011;**140**:1667-8.

117. Cuquemelle E, Soulis F, Villers D, Roche-Campo F, Ara SC, Fartoukh M *et al*. Can procalcitonin help identify associated bacterial infection in patients with severe influenza pneumonia? A multicentre study. *Intensive Care Med* 2011;**37**:796-800.

118. Khan DA, Rahman A, Khan FA. Is procalcitonin better than C-reactive protein for early diagnosis of bacterial pneumonia in children? *J.Clin.Lab Anal.* 2010;**24**:1-5.

119. Kolditz M, Halank M, Schulte-Hubbert B, Hoffken G. Procalcitonin improves the differentiation between infectious and cryptogenic/secondary organizing pneumonia. *J.Infect.* 2012;**64**:122-4.

120. Okimoto N, Hayashi Y, Ishiga M, Nanba F, Kishimoto M, Yagi S *et al*. Procalcitonin and severity of community-acquired pneumonia. *J.Infect.Chemother.* 2009;**15**:426-7.

121. Park JH, Wee JH, Choi SP, Oh SH. The value of procalcitonin level in community-acquired pneumonia in the ED. *Am.J.Emerg.Med* 2011.

122. Almansa R, Sanchez-Garcia M, Herrero A, Calzada S, Roig V, Barbado J *et al*. Host response cytokine signatures in viral and nonviral acute exacerbations of chronic obstructive pulmonary disease. *J.Interferon Cytokine Res.* 2011;**31**:409-13.

123. Hagau N, Slavcovici A, Gonganau DN, Oltean S, Dirzu DS, Brezoszki ES *et al*. Clinical aspects and cytokine response in severe H1N1 influenza A virus infection. *Crit Care* 2010;**14**:R203.

124. Quint JK, Donaldson GC, Goldring JJ, Baghai-Ravary R, Hurst JR, Wedzicha JA. Serum IP-10 as a biomarker of human rhinovirus infection at exacerbation of COPD. *Chest* 2010;**137**:812-22.

125. Roe MF, Bloxham DM, Cowburn AS, O'Donnell DR. Changes in helper lymphocyte chemokine receptor expression and elevation of IP-10 during acute respiratory syncytial virus infection in infants. *Pediatr.Allergy Immunol.* 2011;**22**:229-34.

126. Wark PA, Bucchieri F, Johnston SL, Gibson PG, Hamilton L, Mimica J *et al*. IFN-gamma-induced protein 10 is a novel biomarker of rhinovirus-induced asthma exacerbations. *J.Allergy Clin.Immunol.* 2007;**120**:586-93.

127. Wood LG, Powell H, Grissell TV, Davies B, Shafren DR, Whitehead BF *et al*. Persistence of rhinovirus RNA and IP-10 gene expression after acute asthma. *Respirology* 2011;**16**:291-9.

128. Wood LG, Simpson JL, Wark PA, Powell H, Gibson PG. Characterization of innate immune signalling receptors in virus-induced acute asthma. *Clin.Exp.Allergy* 2011;**41**:640-8.

129. Fondell E, Lagerros YT, Sundberg CJ, Lekander M, Balter O, Rothman KJ *et al*. Physical activity, stress, and self-reported upper respiratory tract infection. *Med.Sci.Sports Exerc.* 2011;**43**:272-9.

130. Kabat-Zinn J, Lipworth L, Burney R, Sellers W. Four-year follow-up of a meditation-based program for the self-regulation of chronic pain: Treatment outcomes and compliance. *The Clinical Journal of Pain* 1987;**2**:159-73.

131. Kabat-Zinn J. Mindfulness meditation: Health benefits of an ancient Buddist practice. In Goleman D, Gurin J, eds. *Mind / body medicine: How to use your mind for better health*, pp 259-75. New York: Consumers Union, 1993.

132. Kabat-Zinn J. Mindfullness-based interventions in context: Past, present, future. *Clin Psychol Sci Prac* 2003;**10**:144-56.

133. Carlson LE,.Garland SN. Impact of mindfulness-based stress reduction (MBSR) on sleep, mood, stress and fatigue symptoms in cancer outpatients. *International Journal of Behavioral Medicine* 2005;**12**:278-85.

134. Kabat-Zinn J, Massion AO, Kristeller J, Peterson LG, Fletcher K, Pbert L *et al*. Effectiveness of a meditation-based stress-reduction program in the treatment of anxiety disorders. *American Journal of Psychiatry* 1992;**149**:936-43.

135. Shapiro SL, Bootzin RR, Figueredo AJ, Lopez AM, Schwartz GE. The efficacy of mindfulness-based stress reduction in the treatment of sleep disturbance in women with breast cancer: an exploratory study. *Journal of Psychosomatic Research* 2003;**54**:85-91.

136. Shapiro SL, Schwartz GE, Bonner G. Effects of mindfulness-based stress reduction on medical and premedical students. *Journal of Behavioral Medicine* 1998;**21**:581-99.

137. Speca M, Carlson LE, Goodey E, Angen M. A randomized, wait-list controlled clinical trial: The effect of a mindfulness meditation-based stress reduction program on mood and symptoms of stress in cancer outpatients. *Psychosomatic Medicine* 2000;**62**:613-22.

138. Williams KA, Kolar MM, Reger BE, Pearson JC. Evaluation of a Wellness-Based Mindfulness Stress Reduction intervention: a controlled trial. *Am J Health Promot.* 2001;**15**:422-32.

139. Ma SH,.Teasdale JD. Mindfulness-based cognitive therapy for depression: replication and exploration of differential relapse prevention effects. *J Consult Clin Psychol* 2004;**72**:31-40.

140. Teasdale JD, Segal ZV, Williams JM, Ridgeway VA, Soulsby JM, Lau MA. Prevention of relapse/recurrence in major depression by mindfulness-based cognitive therapy. *J Consult Clin Psychol* 2000;**68**:615-23.

141. de Vibe M,.Moum T. [Training in mindfulness for patients with stress and chronic illness]. *Tidsskr.Nor Laegeforen.* 2006;**126**:1898-902.

142. Gregg EW, Gerzoff RB, Caspersen CJ, Williamson DF, Narayan KM. Relationship of walking to mortality among US adults with diabetes. *Archives of Internal Medicine.* 2003;**163**:1440-7.

143. Manson JE, Greenland P, LaCroix AZ, Stefanick ML, Mouton CP, Oberman A *et al*. Walking compared with vigorous exercise for the prevention of cardiovascular events in women. *New England Journal of Medicine.* 2002;**347**:716-25.

144. Tanasescu M, Leitzmann MF, Rimm EB, Willett WC, Stampfer MJ, Hu FB. Exercise type and intensity in relation to coronary heart disease in men. *JAMA.* 2002;**288**:1994-2000.

145. Whelton SP, Chin A, Xin X, He J. Effect of aerobic exercise on blood pressure: a meta-analysis of randomized, controlled trials. *Annals of Internal Medicine.* 2002;**136**:493-503.

146. Fleshner M. Exercise and neuroendocrine regulation of antibody production: protective effect of physical activity on stress-induced suppression of the specific antibody response. *International Journal of Sports Medicine* 2000;**21**:Suppl-9.

147. Fleshner M, Campisi J, Johnson JD. Can exercise stress facilitate innate immunity? A functional role for stress-induced extracellular Hsp72. *Exercise Immunology Review* 2003;**9**:6-24.

148. Gleeson M, Pyne DB, Callister R. The missing links in exercise effects on mucosal immunity. *Exercise Immunology Review* 2004;**10**:107-28.

149. Jonsdottir IH. Exercise immunology: neuroendocrine regulation of NK-cells. *International Journal of Sports Medicine* 2000;**21**:Suppl-3.

150. Mackinnon LT. Chronic exercise training effects on immune function. *Medicine & Science in Sports & Exercise* 2000;**32**:Suppl-76.

151. Moseley PL. Exercise, stress, and the immune conversation. *Exercise & Sport Sciences Reviews* 2000;**28**:128-32.

152. Nielsen HB. Lymphocyte responses to maximal exercise: a physiological perspective. *Sports Medicine* 2003;**33**:853-67.

153. Nieman DC, Miller AR, Henson DA, Warren BJ, Gusewitch G, Johnson RL *et al*. Effects of high- vs moderate-intensity exercise on natural killer cell activity. *Med Sci Sports Exerc.* 1993;**25**:1126-34.

154. Nieman DC. Exercise, immunology and nutrition. *World Review of Nutrition & Dietetics* 2001;**90**:89-101.

155. Pershin BB, Geliev AB, Tolstov DV, Kovalchuk LV, Medvedev VY. Reactions of immune system to physical exercises. *Russian Journal of Immunology* 2002;**7**:2-24.

156. Rowbottom DG,.Green KJ. Acute exercise effects on the immune system. *Medicine & Science in Sports & Exercise* 2000;**32**:Suppl-405.

157. Schuler PB, Lloyd LK, Leblanc PA, Clapp TA, Abadie BR, Collins RK. The effect of physical activity and fitness on specific antibody production in college students. *Journal of Sports Medicine & Physical Fitness* 1999;**39**:233-9.

158. Venjatraman JT,.Fernandes G. Exercise, immunity and aging. *Aging-Clinical & Experimental Research* 1997;**9**:42-56.

159. Kostka T, Berthouze SE, Lacour J-R, Bonnefoy M. The symptomatology of upper respiratory tract infections and exercise in elderly people. *Medicine & Science in Sports & Exercise* 2000;**32**:46-51.

160. Klentrou P, Cieslak T, MacNeil M, Vintinner A, Plyley M. Effect of moderate exercise on salivary immunoglobulin A and infection risk in humans. *European Journal of Applied Physiology* 2002;**87**:153-8.

161. Matthews CE, Ockene IS, Freedson PS, Rosal MC, Merriam PA, Hebert JR. Moderate to vigorous physical activity and risk of upper-respiratory tract infection. *Medicine & Science in Sports & Exercise* 2002;**34**:1242-8.

162. Mazzeo RS. The influence of exercise and aging on immune function. *Medicine & Science in Sports & Exercise* 1994;**26**:586-92.

163. Nieman DC. Exercise, upper respiratory tract infection, and the immune system. *Medicine & Science in Sports & Exercise* 1994;**26**:128-39.

164. Nieman DC. Moderate exercise improves immunity and decreases illness rates. *American Journal of Lifestyle Modification* 2012;**In press**.

165. Weidner TG. Literature review: upper respiratory illness and sport and exercise. *Int.J.Sports Med.* 1994;**15**:1-9.

166. Weidner TG, Cranston T, Schurr T, Kaminsky LA. The effect of exercise training on the severity and duration of a viral upper respiratory illness. *Medicine & Science in Sports & Exercise* 1998;**30**:1578-83.

167. Nieman DC, Nehlsen-Cannarella SL, Markoff PA, Balk-Lamberton AJ, Yang H, Chritton DB *et al*. The effects of moderate exercise training on natural killer cells and acute upper respiratory tract infections. *Int.J Sports Med* 1990;**11**:467-73.

168. Nieman DC, Henson DA, Austin MD, Sha W. Upper respiratory tract infection is reduced in physically fit and active adults. *Br.J.Sports Med.* 2010.

169. Weidner T,.Schurr T. Effect of exercise on upper respiratory tract infection in sedentary subjects. *British Journal of Sports Medicine* 2003;**37**:304-6.

170. Jackson GG, Dowling HF, Spiesman IG, Boand AV. Transmission of the common cold to volunteers under controlled conditions. *Arch Intern Med* 1958;**101**:267-78.

171. Jackson GG, Dowling HF, Anderson TO, Riff L, Saporta J, Turck M. Susceptibility and immunity to common upper respiratory viral infections-the common cold. *Annals of Internal Medicine* 1960;**55**:719-38.

172. Jackson GG, Dowling HF, Muldoon RL. Present concepts of the common cold. *Am J Public Health* 1962;**52**:940-5.

173. Barrett B, Brown R, Mundt M, Safdar N, Dye L, Maberry R *et al*. The Wisconsin Upper Respiratory Symptom Survey is responsive, reliable, and valid. *Journal of Clinical Epidemiology* 2005;**58**:609-17.

174. Barrett B, Brown R, Voland R, Maberry R, Turner R. Relations among questionnaire and laboratory measures of rhinovirus infection. *European Respiratory Journal* 2006;**28**:358-63.

175. Barrett B, Brown R, Mundt M. Comparison of anchor-based and distributional approaches in estimating important difference in common cold. *Quality of Life Research* 2008;**17**:75-85.

176. Barrett B, Brown RE, Mundt MP, Thomas GR, Barlow SK, Highstrom AD *et al*. Validation of a short form Wisconsin Upper Respiratory Symptom Survey (WURSS-21). *Health and Quality of Life Outcomes* 2009;**7**.

177. Blalock JE. The immune system as the sixth sense. *Journal of Internal Medicine* 2005;**257**:126-38.

178. Maier SF,.Watkins LR. Cytokines for psychologists: implications of bidirectional immune-to-brain communication for understanding behavior, mood, and cognition. *Psychological Review.* 1998;**105**:83-107.

179. McEwen BS. The neurobiology of stress: from serendipity to clinical relevance. *Brain Research* 2000;**886**:172-89.

180. Zeller JM, McCain NL, McCann JJ, Swanson B, Colletti MA. Methodological issues in psychoneuroimmunology research. *Nursing Research* 1996;**45**:314-8.

181. Cohen S,.Herbert TB. Health psychology: Psychological factors and physical disease from the perspective of human psychoneuroimmunology. *Annual Review of Psychology* 1996;**47**:113-42.

182. Kaplan HB. Social psychology of the immune system: A conceptual framework and review of the literature. *Social Science & Medicine* 1991;**33**:909-23.

183. Korte SM, Koolhaas JM, Wingfield JC, McEwen BS. The Darwinian concept of stress: benefits of allostasis and costs of allostatic load and the trade-offs in health and disease. *Neuroscience & Biobehavioral Reviews* 2005;**29**:3-38.

184. Malarkey WB, Pearl DK, Demers LM, Kiecolt-Glaser JK, Glaser R. Influence of academic stress and season on 24-hour mean concentrations of ACTH, cortisol, and beta-endorphin. *Psychoneuroendocrinology* 1995;**20**:499-508.

185. Petrie KJ, Booth RJ, Pennebaker JW. The immunological effects of thought suppression. *Journal of Personality & Social Psychology.* 1998;**75**:1264-72.

186. Prolo P, Chiappelli F, Fiorucci A, Dovio A, Sartori ML, Angeli A. Psychoneuroimmunology: new avenues of research for the twenty-first century. *Annals of the New York Academy of Sciences* 2002;**966:400-8, 2002 Jun.**:-8.

187. Rosenkranz MA, Jackson DC, Dalton KM, Dolski I, Ryff CD, Singer BH *et al*. Affective style and in vivo immune response: neurobehavioral mechanisms. *Proceedings of the National Academy of Sciences of the United States of America.* 2003;**100**:11148-52.

188. Seeman TE, Singer BH, Rowe JW, Horwitz RI, McEwen BS. Price of adaptation--allostatic load and its health consequences. MacArthur studies of successful aging. *Archives of Internal Medicine* 1997;**157**:2259-68.

189. Barrett B, Locken K, Maberry R, Schwamman J, Bobula J, Brown R *et al*. The Wisconsin Upper Respiratory Symptom Survey: Development of an instrument to measure the common cold. *Journal of Family Practice* 2002;**51**:265-73.

190. Barrett, B., Frey, J. J., Guyatt, G. H., and Brown, R. E. Measuring the minimal important difference: Assessing patient-oriented outcomes in a common cold model. Robert Wood Johnson Generalist Physician Faculty Scholars Program . 2003.

191. Barrett B, Brown R, Mundt M, Dye L, Alt J, Safdar N *et al*. Using benefit harm tradeoffs to estimate sufficiently important difference: the case of the common cold. *Medical Decision Making* 2005;**25**:47-55.

192. Barrett B, Harahan B, Brown D, Zhang Z, Brown R. Sufficiently important difference for common cold: severity reduction. *Ann.Fam.Med* 2007;**5**:216-23.

193. Barrett B, Endrizzi S, Andreoli P, Barlow S, Zhang Z. Clinical significance of common cold treatment: professionals' opinions. *Wisconsin Medical Journal* 2007;**106**:473-80.

194. Brown RL. Using covariance modeling for estimating reliability on scale with ordered polytomous variables. *Educational and Psychological Measurement* 1989;**49**:26-39.

195. Brown RL. Congeneric modeling of reliability using censored variables. *Applied Psychological Measurement* 1989;**13**:201-10.

196. Brown RL. The efficacy of the indirect approach for estimating structural equation models with missing data: A comparison of five methods. *Structural Equation Modeling* 1994;**1**:287-316.

197. Brown RL. Assessing specific mediational effects in complex theoretical models. *Structural Equation Modeling* 1997;**4**:142-56.

198. Coe CL,.Lubach GR. Critical periods of special health relevance for psychoneuroimmunology. *Brain, Behavior, & Immunity* 2003;**17**:3-12.

199. Coe CL. Psychosocial factors and immunity in nonhuman primates: a review. *Psychosom.Med* 1993;**55**:298-308.

200. Coe CL,.Lubach GR. Prenatal origins of individual variation in behavior and immunity. *Neurosci.Biobehav.Rev.* 2005;**29**:39-49.

201. Davidson RJ. Specificity and patterning in biobehavioral systems. Implications for behavior change. *American Psychologist* 1978;**33**:430-6.

202. Davidson RJ. Toward a biology of personality and emotion. *Annals of the New York Academy of Sciences* 2001;**935**:191-207.

203. Davidson RJ. Anxiety and affective style: role of prefrontal cortex and amygdala. *Biological Psychiatry.* 2002;**51**:68-80.

204. Davidson RJ. Affective neuroscience and psychophysiology: toward a synthesis. *Psychophysiology.* 2003;**40**:655-65.

205. Davidson RJ, Coe CC, Dolski I, Donzella B. Individual differences in prefrontal activation asymmetry predict natural killer cell activity at rest and in response to challenge. *Brain Behav.Immun.* 1999;**13**:93-108.

206. Davidson RJ. Empirical explorations of mindfulness: conceptual and methodological conundrums. *Emotion.* 2010;**10**:8-11.

207. Gern JE, Vrtis R, Kelly EAB, Dick EC, Busse WW. Rhinovirus produces nonspecific activation of lymphocytes through a monocyte-dependent mechanism. *Journal of Immunology* 1996;**157**:1605-12.

208. Gern JE. Rhinovirus respiratory infections and asthma. *American Journal of Medicine.* 2002;**112**:Suppl-27S.

209. Gern JE, Brooks GD, Meyer P, Chang A, Shen K, Evans MD *et al*. Bidirectional interactions between viral respiratory illnesses and cytokine responses in the first year of life. *Journal of Allergy & Clinical Immunology* 2006;**117**:72-8.

210. Gern JE, Rosenthal LA, Sorkness RL, Lemanske RF, Jr. Effects of viral respiratory infections on lung development and childhood asthma. *Journal of Allergy & Clinical Immunology* 2005;**115**:668-74.

211. Gern JE. Viral respiratory infection and the link to asthma. *Pediatric Infectious Disease Journal* 2004;**23**:Suppl-86.

212. Gern JE, Martin MS, Anklam KA, Shen K, Roberg KA, Carlson-Dakes KT *et al*. Relationships among specific viral pathogens, virus-induced interleukin-8, and respiratory symptoms in infancy. *Pediatric Allergy & Immunology* 2002;**13**:386-93.

213. Gern JE,.Busse WW. Relationship of viral infections to wheezing illnesses and asthma. *Nature Reviews* 2002;Immunology.:132-8.

214. Hayney MS, Love GD, Buck JM, Ryff CD, Singer B, Muller D. The association between psychosocial factors and vaccine-induced cytokine production. *Vaccine* 2003;**21**:2428-32.

215. Hayney MS, Welter DL, Francois M, Reynolds AM, Love RB. Influenza vaccine antibody responses in lung transplant recipients. *Progress in Transplantation* 2004;**14**:346-51.

216. Hayney MS,.Muller D. Effect of influenza immunization on CYP3A4 activity in vivo. *Journal of Clinical Pharmacology* 2003;**43**:1377-81.

217. Hayney MS, Hammes RJ, Fine JP, Bianco JA. Effect of influenza immunization on CYP3A4 activity. *Vaccine* 2002;**20**:858-61.

218. Hayney MS,.Buck JM. Effect of age and degree of immune activation on cytochrome P450 3A4 activity after influenza immunization. *Pharmacotherapy* 2002;**22**:1235-8.

219. Hayney MS, Love GD, Buck JM, Ryff CD, Singer B, Muller D. The association between psychosocial factors and vaccine-induced cytokine production. *Vaccine* 2003;**21**:2428-32.

220. Hayney MS, Buck JM, Muller D. Production of interferon-gamma and interleukin-10 after inactivated hepatitis A immunization. *Pharmacotherapy* 2003;**23**:431-5.

221. Nieman DC, Henson DA, Gusewitch G, Warren BJ, Dotson RC, Butterworth DE *et al*. Physical activity and immune function in elderly women. *Med Sci Sports Exerc.* 1993;**25**:823-31.

222. Nieman DC, Nehlsen-Cannarella SL, Henson DA, Koch AJ, Butterworth DE, Fagoaga OR *et al*. Immune response to exercise training and/or energy restriction in obese women. *Med Sci Sports Exerc.* 1998;**30**:679-86.

223. Alonso C, Loevinger BL, Muller D, Coe CL. Menstrual cycle influences on pain and emotion in women with fibromyalgia. *Journal of Psychosomatic Research* 2004;**57**:451-8.

224. Barrett B, Muller D, Rakel D, Rabago D, Marchand L, Scheder JC. Placebo, meaning, and health. *Perspect.Biol.Med* 2006;**49**:178-98.

225. Muller D. Fibromyalgia. In Rakel D, ed. *Integrative Medicine*, Philadelphia: Harcourt , 2003.

226. Hayney MS, Love GD, Buck JM, Ryff CD, Singer B, Muller D. The association between psychosocial factors and vaccine-induced cytokine production. *Vaccine* 2003;**21**:2428-32.

227. Rakel D. Integrative Medicine. Philadelphia, London, New York: Saunders, 2003.

228. Rakel D. The salutogenesis-oriented session: creating space and time for healing in primary care. *Explore* 2008;**4**:42-7.

229. Rakel DP, Hoeft TJ, Barrett BP, Chewning BA, Craig BM, Niu M. Practitioner empathy and the duration of the common cold. *Fam.Med.* 2009;**41**:494-501.

230. Rakel DP,.Hedgecock J. Healing the healer: a tool to encourage student reflection towards health. *Med.Teach.* 2008;**30**:633-5.

231. Rakel DP, Guerrera MP, Bayles BP, Desai GJ, Ferrara E. CAM education: promoting a salutogenic focus in health care. *J.Altern.Complement Med.* 2008;**14**:87-93.

232. Rakel DP,.Rindfleisch A. Inflammation: nutritional, botanical, and mind-body influences. *South.Med.J.* 2005;**98**:303-10.

233. Zgierska A, Rabago D, Chawla N, Kushner K, Koehler R, Marlatt A. Mindfulness meditation for substance use disorders: a systematic review. *Subst.Abus.* 2009;**30**:266-94.

234. Zgierska A,.Marcus MT. Mindfulness-based therapies for substance use disorders: part 2. *Subst.Abus.* 2010;**31**:77-8.

235. Zgierska A, Rabago D, Zuelsdroff M, Coe C, Miller M, Fleming M. Mindfulness meditation for alcohol relapse prevention: A feasibility pilot study. *Journal of Addiction Medicine* 2008;**2**:165-73.

236. Pfau PR, Perlman SB, Stanko P, Frick TJ, Gopal DV, Said A *et al*. The role and clinical value of EUS in a multimodality esophageal carcinoma staging program with CT and positron emission tomography. *Gastrointest.Endosc.* 2007;**65**:377-84.

237. Rakel DP, Barrett B, Zhang Z, Hoeft TJ, Chewning B, Marchand L *et al*. Perception of empathy in the therapeutic encounter: Effects on the common cold. *Patient Educ.Couns.* 2011; 85(3):390-297.

238. Xiong C, Yu K, Gao F, Yan Y, Zhang Z. Power and sample size for clinical trials when efficacy is required in multiple endpoints: application to an Alzheimer's treatment trial. *Clinical Trials* 2005;**2**:387-93.

239. Zgierska A, Brown RT, Zuelsdorff M, Brown D, Zhang Z, Fleming MF. Sleep and daytime sleepiness problems among patients with chronic noncancerous pain receiving long-term opioid therapy: a cross-sectional study. *J.Opioid.Manag.* 2007;**3**:317-27.

240. Centers for Disease Control and Prevention, Department of Health and Human Services. Behavioral Risk Factor Surveillance System Survey Questionnaire. Atlanta: Centers for Disease Control and Prevention, 2005.

241. Fletcher GF, Balady GJ, Amsterdam EA, Chaitman B, Eckel R, Fleg J *et al*. Exercise standards for testing and training: a statement for healthcare professionals from the American Heart Association. *Circulation* 2001;**104**:1694-740.

242. Nieman DC. How do the 2008 physical activity guidelines for Americans differ from previous recommendations? *ACSM's Health & Fitness Journal* 2009;**13**:1-3.

243. Physical Activity Guidelines Advisory Committee. Physical Activity Guidelines Advisory Committee Report. 2008. Washington D.C., U.S. Department of Health and Human Services.

244. Barrett B, Brown R, Rakel D, Mundt M, Bone K, Barlow S *et al*. Echinacea for treating the common cold: A randomized controlled trial. *Annals of Internal Medicine* 2010;**153**:769-77.

245. Schafer JL. Analysis of Incomplete Multivariate Data. London: Chapman & Hall, 1997.

246. Hedeker D,.Gibbons RD. Application of random-effects pattern-mixture models for missing data in longitudinal studies. *Psychological Methods* 1997;**2**:64-78.

247. Cayoun BA, Freestun J. Mindfulness-based self-efficacy scale (MSES). http://www.mytherapysession.com/PDFs/MSESSelfEfficacyScale.pdf, 2004.

248. Carlson LE,.Brown KW. Validation of the Mindful Attention Awareness Scale in a cancer population. *J.Psychosom.Res.* 2005;**58**:29-33.

249. Brown KW,.Ryan RM. The benefits of being present: mindfulness and its role in psychological well-being. *J.Pers.Soc.Psychol.* 2003;**84**:822-48.

250. Grossman P, Niemann L, Schmidt S, Walach H. Mindfulness-based stress reduction and health benefits. A meta-analysis. *J Psychosom.Res.* 2004;**57**:35-43.

251. Perri MG, Anton SD, Durning PE, Ketterson TU, Sydeman SJ, Berlant NE *et al*. Adherence to exercise prescriptions: effects of prescribing moderate versus higher levels of intensity and frequency. *Health Psychol* 2002;**21**:452-8.

252. Kohut ML, Martin AE, Senchina DS, Lee W. Glucocorticoids produced during exercise may be necessary for optimal virus-induced IL-2 and cell proliferation whereas both catecholamines and glucocorticoids may be required for adequate immune defense to viral infection. *Brain, Behavior, & Immunity* 2005;**19**:423-35.

253. Borg GV,.Linderholm H. Percieved exertion and pulse rate during graded exercise in various age groups. *Acta Medica Scandinavica* 1970;**472**:194-206.

254. Morgan W, Borg GA. Perception of effort in the prescription of physical activity. In Nelson T, ed. *Mental Health and Emotional Aspects of Sports*, pp 126-9. Chicago: American Medical Association, 1976.

255. Armstrong T,.Bull F. Development of the World Health Organization Global Physical Activity Questionnaire (GPAQ). *Journal of Public Health* 2006;**14**:66-70.

256. Bull FC, Maslin TS, Armstrong T. Global physical activity questionnaire (GPAQ): nine country reliability and validity study. *J.Phys.Act.Health* 2009;**6**:790-804.

257. Kirsch I. How Expectancies Shape Experience. Washington DC: American Psychological Association, 1999.

258. Kaptchuk TJ, Kelley JM, Conboy LA, Davis RB, Kerr CE, Jacobson EE *et al*. Components of placebo effect: randomised controlled trial in patients with irritable bowel syndrome. *BMJ* 2008;**336**:999-1003.

259. Barrett B, Locken K, Maberry R, Schwamman J, Bobula J, Brown R *et al*. The Wisconsin Upper Respiratory Symptom Survey: Development of an instrument to measure the common cold. *Journal of Family Practice* 2002;**51**:265-73.

260. Palmer LA, Rousculp MD, Johnston SS, Mahadevia PJ, Nichol KL. Effect of influenza-like illness and other wintertime respiratory illnesses on worker productivity: The child and household influenza-illness and employee function (CHIEF) study. *Vaccine* 2010;**28**:5049-56.

261. Jartti T, Lee WM, Pappas T, Evans M, Lemanske RF, Jr., Gern JE. Serial viral infections in infants with recurrent respiratory illnesses. *Eur.Respir.J.* 2008;**32**:314-20.

262. Lee WM, Kiesner C, Pappas T, Lee I, Grindle K, Jartti T *et al*. A diverse group of previously unrecognized human rhinoviruses are common causes of respiratory illnesses in infants. *PLoS One* 2007;**2**:e966.

263. Mosser AG, Vrtis R, Burchell L, Lee WM, Dick CR, Weisshaar E *et al*. Quantitative and qualitative analysis of rhinovirus infection in bronchial tissues. *American Journal of Respiratory & Critical Care Medicine* 2005;**171**:645-51.

264. Olenec JP, Kim WK, Lee WM, Vang F, Pappas TE, Salazar LE *et al*. Weekly monitoring of children with asthma for infections and illness during common cold seasons. *J.Allergy Clin.Immunol.* 2010;**125**:1001-6.

265. Lee WM, Grindle K, Pappas T, Marshall DJ, Moser MJ, Beaty EL *et al*. High-throughput, sensitive, and accurate multiplex PCR-microsphere flow cytometry system for large-scale comprehensive detection of respiratory viruses. *J Clin Microbiol* 2007;**45**:2626-34.

266. DeVincenzo JP, Wilkinson T, Vaishnaw A, Cehelsky J, Meyers R, Nochur S *et al*. Viral load drives disease in humans experimentally infected with respiratory syncytial virus. *Am.J.Respir.Crit Care Med.* 2010;**182**:1305-14.

267. Doyle WJ, Casselbrant ML, Li-Korotky HS, Doyle AP, Lo CY, Turner R *et al*. The interleukin 6 -174 C/C genotype predicts greater rhinovirus illness. *J.Infect.Dis.* 2010;**201**:199-206.

268. El Feghaly RE, McGann L, Bonville CA, Branigan PJ, Suryadevera M, Rosenberg HF *et al*. Local production of inflammatory mediators during childhood parainfluenza virus infection. *Pediatr.Infect.Dis.J.* 2010;**29**:e26-e31.

269. Hosoda M, Yamaya M, Suzuki T, Yamada N, Kamanaka M, Sekizawa K *et al*. Effects of rhinovirus infection on histamine and cytokine production by cell lines from human mast cells and basophils. *Journal of Immunology* 2002;**169**:1482-91.

270. Linden M, Greiff L, Andersson M, Svensson C, Akerlund A, Bende M *et al*. Nasal cytokines in common cold and allergic rhinitis. *Clinical & Experimental Allergy* 1995;**25**:166-72.

271. Roseler S, Holtappels G, Wagenmann M, Bachert C. Elevated levels of interleukins IL-1 beta, IL-6 and IL-8 in naturally acquired viral rhinitis. *European Archives of Oto-Rhino-Laryngology.Supplement* 1995;**1**:S61-S63.

272. Becker S, Koren HS, Henke DC. Interleukin-8 expression in normal nasal epithelium and its modulation by infection with respiratory syncytial virus and cytokines tumor necrosis factor, Interleukin-1, and Interleukin-6. *Am.J.Respir.Cell Mol.Biol.* 1993;**8**:20-7.

273. Biagioli MC, Kaul P, Singh I, Turner RB. The role of oxidative stress in rhinovirus induced elaboration of IL-8 by respiratory epithelial cells. *Free Radical Biology & Medicine* 1999;**26**:454-62.

274. Garofalo R, Patel JA, Sim C, Schmalstieg FC, Goldman AS. Production of cytokines by virus-infected human respiratory epithelial cells. *J Allergy Clin Immunol* 1993;**91**:177.

275. Johnston SL, Papi A, Monick MM, Hunninghake GW. Rhinoviruses induce interleukin-8 mRNA and protein production in human monocytes. *Journal of Infectious Disease* 1997;**175**:323.

276. Mastronarde JG, He B, Monick MM, Mukaida N, Matsushima K, Hunninghake GW. Induction of Interleukin (IL)-8 gene expression by respiratory syncytial virus involves activation of nuclear factor (NF)-kB and NF-IL-6. *The Journal of Infectious Diseases* 1996;**174**:262-7.

277. Cervoni JP, Thevenot T, Weil D, Muel E, Barbot O, Sheppard F *et al*. C-Reactive protein predicts short-term mortality in patients with cirrhosis. *J.Hepatol.* 2012.

278. Fan ZX, Hua Q, Li YP, Liu RK, Yang Z. Interleukin-6, but not soluble adhesion molecules, predicts a subsequent mortality from cardiovascular disease in patients with acute ST-segment elevation myocardial infarction. *Cell Biochem.Biophys.* 2011;**61**:443-8.

279. Giovannini S, Onder G, Liperoti R, Russo A, Carter C, Capoluongo E *et al*. Interleukin-6, C-reactive protein, and tumor necrosis factor-alpha as predictors of mortality in frail, community-living elderly individuals. *J.Am.Geriatr.Soc.* 2011;**59**:1679-85.

280. Heffner KL, Waring ME, Roberts MB, Eaton CB, Gramling R. Social isolation, C-reactive protein, and coronary heart disease mortality among community-dwelling adults. *Soc.Sci Med* 2011;**72**:1482-8.

281. Hermida J, Lopez FL, Montes R, Matsushita K, Astor BC, Alonso A. Usefulness of high-sensitivity C-reactive protein to predict mortality in patients with atrial fibrillation (from the Atherosclerosis Risk In Communities [ARIC] Study). *Am.J.Cardiol.* 2012;**109**:95-9.

282. Kengne AP, Batty GD, Hamer M, Stamatakis E, Czernichow S. Association of C-Reactive Protein With Cardiovascular Disease Mortality According to Diabetes Status: Pooled analyses of 25,979 participants from four U.K. prospective cohort studies. *Diabetes Care* 2012;**35**:396-403.

283. Meng FS, Su L, Tang YQ, Wen Q, Liu YS, Liu ZF. Serum procalcitonin at the time of admission to the ICU as a predictor of short-term mortality. *Clin.Biochem.* 2009;**42**:1025-31.

284. Tong HS, Liu YS, Wen Q, Tang YQ, Yuan FF, Su L. Serum procalcitonin predicting mortality in exertional heatstroke. *Emerg.Med J.* 2012;**29**:113-7.

285. Vepsalainen T, Soinio M, Marniemi J, Lehto S, Juutilainen A, Laakso M *et al*. Physical activity, high-sensitivity C-reactive protein, and total and cardiovascular disease mortality in type 2 diabetes. *Diabetes Care* 2011;**34**:1492-6.

286. Abbas AK, Lichtman AH, Pober JS. Cellular and molecular immunology. Philadelphia: W.B. Saunders, 2000.

287. Janeway CA, Travers P, Walport M, Capra JD. Immunobiology: The immune system in health and disease. London & New York: Current Biology Publications, 1999.

288. Naclerio RM, Proud D, Lichtenstein LM, Kagey-Sobotka A, Hendley JO, Sorrentino J *et al*. Kinins are generated during experimental rhinovirus colds. *The Journal of Infectious Diseases* 1988;**157**:133-42.

289. Teran LM, Johnston SL, Schroder JM, Church MK, Holgate ST. Role of nasal interleukin-8 in neutrophil recruitment and activation in children with virus induced asthma. *Am.J.Respir.Cri.Care Me.* 1997;**155**:1362-6.

290. Turner RB. The treatment of rhinovirus infections: Progress and potential. *Antiviral Res* 2001;**49**:1-14.

291. Turner RB, Bauer R, Woelkart K, Hulsey TC, Gangemi JD. An evaluation of Echinacea angustifolia in experimental rhinovirus infections. *New England Journal of Medicine* 2005;**353**:341-8.

292. Umpierre D, Ribeiro PA, Kramer CK, Leitao CB, Zucatti AT, Azevedo MJ *et al*. Physical activity advice only or structured exercise training and association with HbA1c levels in type 2 diabetes: a systematic review and meta-analysis. *JAMA* 2011;**305**:1790-9.

293. Wing RR. Long-term effects of a lifestyle intervention on weight and cardiovascular risk factors in individuals with type 2 diabetes mellitus: four-year results of the Look AHEAD trial. *Arch.Intern.Med.* 2010;**170**:1566-75.

294. Knowler WC, Fowler SE, Hamman RF, Christophi CA, Hoffman HJ, Brenneman AT *et al*. 10-year follow-up of diabetes incidence and weight loss in the Diabetes Prevention Program Outcomes Study. *Lancet* 2009;**374**:1677-86.

295. Rosenzweig S, Reibel DK, Greeson JM, Edman JS, Jasser SA, McMearty KD *et al*. Mindfulness-based stress reduction is associated with improved glycemic control in type 2 diabetes mellitus: a pilot study. *Altern.Ther.Health Med* 2007;**13**:36-8.

296. Khatri D, Mathur KC, Gahlot S, Jain S, Agrawal RP. Effects of yoga and meditation on clinical and biochemical parameters of metabolic syndrome. *Diabetes Res.Clin.Pract.* 2007;**78**:e9-10.

297. Cohen S, Tyrell DAJ, Russell MAH, Jarvis MJ, MPhil, Smith AP. Smoking, alcohol consumption, and susceptibility to the common cold. *American Journal of Public Health* 1993;**83**:1277-83.

298. Carey KB, Carey MP, Maisto SA, Henson JM. Temporal stability of the timeline followback interview for alcohol and drug use with psychiatric outpatients. *Journal of Studies on Alcohol* 2004;**65**:774-81.

299. Duhig AM, Cavallo DA, McKee SA, George TP, Krishnan-Sarin S. Daily patterns of alcohol, cigarette, and marijuana use in adolescent smokers and nonsmokers. *Addictive Behaviors* 2005;**30**:271-83.

300. LaBrie J, Pedersen E, Earleywine M. A group-administered Timeline Followback assessment of alcohol use. *Journal of Studies on Alcohol* 2005;**66**:693-7.

301. Sobell LC, Agrawal S, Sobell MB, Leo GI, Young LJ, Cunningham JA *et al*. Comparison of a quick drinking screen with the timeline followback for individuals with alcohol problems. *Journal of Studies on Alcohol* 2003;**64**:858-61.

302. Sobell LC, Agrawal S, Annis H, Ayala-Velazquez H, Echeverria L, Leo GI *et al*. Cross-cultural evaluation of two drinking assessment instruments: alcohol timeline followback and inventory of drinking situations. *Substance Use & Misuse* 2001;**36**:313-31.

303. Cohen S, Doyle WJ, Turner RB, Alper CM, Skoner DP. Childhood socioeconomic status and host resistance to infectious illness in adulthood. *Psychosomatic Medicine* 2004;**66**:553-8.

304. Kaplan GA, Pamuk ER, Lynch JW, Cohen RD, Balfour JL. Inequality in income and mortality in the United States: Analysis of mortality and potential pathways. *British Journal of Medicine* 1996;**312**:999-1003.

305. Baxter R, Lee J, Fireman B. Evidence of bias in studies of influenza vaccine effectiveness in elderly patients. *J.Infect.Dis.* 2010;**201**:186-9.

306. Fireman B, Lee J, Lewis N, Bembom O, van der LM, Baxter R. Influenza vaccination and mortality: differentiating vaccine effects from bias. *Am.J.Epidemiol.* 2009;**170**:650-6.

307. Hak E, Hoes AW, Nordin J, Nichol KL. Benefits of influenza vaccine in US elderly--appreciating issues of confounding bias and precision. *Int.J.Epidemiol.* 2006;**35**:800-2.

308. Fan VS, Au D, Heagerty P, Deyo RA, McDonell MB, Fihn SD. Validation of case-mix measures derived from self-reports of diagnoses and health. *Journal of Clinical Epidemiology* 2002;**55**:371-80.

309. Chaplin WF, John OP, Goldberg LR. Conceptions of states and traits: dimensional attributes with ideals as prototypes. *J.Pers.Soc.Psychol.* 1988;**54**:541-57.

310. Briggs SR. Assessing the five-factor model of personality description. *J.Pers.* 1992;**60**:253-93.

311. John OP, Hampson SE, Goldberg LR. The basic level in personality-trait hierarchies: studies of trait use and accessibility in different contexts. *J.Pers.Soc.Psychol.* 1991;**60**:348-61.

312. McCrae RR, Costa PT, Jr., Pedroso dL, Simoes A, Ostendorf F, Angleitner A *et al*. Age differences in personality across the adult life span: parallels in five cultures. *Dev.Psychol.* 1999;**35**:466-77.

313. John OP, Naumann LP, Soto CJ. Paradigm shift to the integrative big-five trait taxonomy: History, measurement and conceptual issues. In John OP, Robins RW, Pervin LA, eds. *Handbook of Personality: Theory and Research*, pp 114-58. New York: Guilford Press, 2008.

314. McCrae RR,.Costa PT, Jr. Validation of the five-factor model of personality across instruments and observers. *J.Pers.Soc.Psychol.* 1987;**52**:81-90.

315. Vecchione M, Alessandri G, Barbaranelli C, Caprara G. Higher-order factors of the big five and basic values: empirical and theoretical relations. *Br.J.Psychol.* 2011;**102**:478-98.

316. John, O. P., Donahue, E. M., and Kentle, R. L. The Big Five Inventory--Versions 4a and 54 . 1991. Berkeley, CA, University of California,Berkeley, Institute of Personality and Social Research.

317. Duncan GJ,.Petersen E. The long and short of asking questions about income, wealth, and labor supply. *Social Science Research* 2001;**30**:248-63.

318. Mattke S, Balakrishnan A, Bergamo G, Newberry SJ. A review of methods to measure health-related productivity loss. *Am.J.Manag.Care* 2007;**13**:211-7.

319. Johns G. Attendance dynamics at work: The antecedents and correlates of presenteeism, absenteeism, and productivity loss. *J.Occup.Health Psychol.* 2011;**16**:483-500.

320. Koopman C, Pelletier KR, Murray JF, Sharda CE, Berger ML, Turpin RS *et al*. Stanford presenteeism scale: health status and employee productivity. *J.Occup.Environ.Med* 2002;**44**:14-20.

321. Turpin RS, Ozminkowski RJ, Sharda CE, Collins JJ, Berger ML, Billotti GM *et al*. Reliability and validity of the Stanford Presenteeism Scale. *J.Occup.Environ.Med* 2004;**46**:1123-33.

322. Huang FY, Chung H, Kroenke K, Delucchi KL, Spitzer RL. Using the Patient Health Questionnaire-9 to measure depression among racially and ethnically diverse primary care patients. *J Gen.Intern.Med* 2006;**21**:547-52.

323. Huang FY, Chung H, Kroenke K, Spitzer RL. Racial and ethnic differences in the relationship between depression severity and functional status. *Psychiatr.Serv.* 2006;**57**:498-503.

324. Kroenke K, Spitzer RL, Williams JBW. The PHQ-9: Validity of a brief depression severity measure. *J Gen Intern Med* 2001;**16**:606-13.

325. Lee PW, Schulberg HC, Raue PJ, Kroenke K. Concordance between the PHQ-9 and the HSCL-20 in depressed primary care patients. *J Affect.Disord.* 2007;**99**:139-45.

326. Williams JW, Jr., Noel PH, Cordes JA, Ramirez G, Pignone M. Is this patient clinically depressed? *JAMA* 2002;**287**:1160-70.

327. Williams LS, Brizendine EJ, Plue L, Bakas T, Tu W, Hendrie H *et al*. Performance of the PHQ-9 as a screening tool for depression after stroke. *Stroke* 2005;**36**:635-8.

328. Lowe B, Unutzer J, Callahan CM, Perkins AJ, Kroenke K. Monitoring depression treatment outcomes with the patient health questionnaire-9. *Med Care* 2004;**42**:1194-201.

329. Lowe B, Kroenke K, Herzog W, Grafe K. Measuring depression outcome with a brief self-report instrument: sensitivity to change of the Patient Health Questionnaire (PHQ-9). *J Affect.Disord.* 2004;**81**:61-6.

330. Brazier JE, Harper R, Jones NMB, O'Cathain A, Thomas KJ, Usherwood T *et al*. Validating the SF-36 health survey questionnaire: New outcome measure for primary care. *BMJ* 1992;**305**:160-4.

331. Jenkinson C, Wright L, Coulter A. Criterion validity and reliability of the SF-36 in a population sample. *Quality of Life Research* 1994;**3**:7-12.

332. McHorney CA, Ware JE, Raczek AE. The MOS 36-item short-form health survey (SF-36): II. Psychometric and clinical tests of validity in measuring physical and mental health constructs. *Medical Care* 1998;**31**:247-63.

333. McHorney CA, Ware JE, Lu JFR, Sherbourne CD. The MOS 36-Item short-form health survery (SF-36): III. Tests of data quality, scaling assumptions, and reliability across diverse patient groups. *Medical Care* 1994;**32**:40-66.

334. Ware JE,.Sherbourne CD. The MOS 36-item short-form health survey (SF-36): I. Conceptual framework and item selection. *Medical Care* 1992;**30**:473-83.

335. Backhaus J, Junghanns K, Broocks A, Riemann D, Hohagen F. Test-retest reliability and validity of the Pittsburgh Sleep Quality Index in primary insomnia. *Journal of Psychosomatic Research* 2002;**53**:737-40.

336. Buysse DJ, Reynolds CF, III, Monk TH, Berman SR, Kupfer DJ. The Pittsburgh Sleep Quality Index: a new instrument for psychiatric practice and research. *Psychiatry Research* 1989;**28**:193-213.

337. Fictenberg NL, Putnam SH, Mann NR, Zafonte RD, Millard AE. Insomnia screening in postacute traumatic brain injury: utility and validity of the Pittsburgh Sleep Quality Index. *American Journal of Physical Medicine & Rehabilitation* 2001;**80**:339-45.

338. Watson D, Clark LA, Tellegen A. Development and validation of brief measures of positive and negative affect: the PANAS scales. *Journal of Personality & Social Psychology* 1988;**54**:1063-70.

339. Watson D,.Pennebaker JW. Health complaints, stress, and distress: exploring the central role of negative affectivity. *Psychological Review* 1989;**96**:234-54.

340. Cohen S, Tyrrell DAJ, Smith AP. Negative life events, perceived stress, negative affect, and susceptibility to the common cold. *Journal of Personality and Social Psychology* 1993;**64**:131-40.

341. Cohen S, Kamarck T, Mermelstein R. A global measure of perceived stress. *The Journal of Health and Social Behavior* 1983;**24**:385-96.

342. Cohen S, Williamson G. Perceived stress in a probability sample of the United States. In Spacapam S, Oskamp S, eds. *The social psychology of health: Claremont Symposium on applied social psychology*, Newbury Park, CA: Sage Publications, 1988.

343. Cohen S,.Janicki-Deverts D. Who's stressed? Distributions of psychological stress in the United States in probability samples from 1983, 2006 and 2009. *Journal of Applied Psychology.* 2011;**In press**.

344. Russell D, Cutrona CE, Rose J, Yurko K. Social and emotional loneliness: an examination of Weiss's typology of loneliness. *J.Pers.Soc.Psychol.* 1984;**46**:1313-21.

345. Cutrona C, Russell D, Rose J. Social support and adaptation to stress by the elderly. *Psychol.Aging* 1986;**1**:47-54.

346. Russell D, Peplau LA, Cutrona CE. The revised UCLA Loneliness Scale: concurrent and discriminant validity evidence. *J.Pers.Soc.Psychol.* 1980;**39**:472-80.

347. Brissette I, Cohen S, Seeman TA. Measuring social integration and social networks. In Cohen S, Underwood LG, Gottlieb BH, eds. *Social support measurement and intervention*, pp 53-85. Oxford: Oxford University Press, 2000.

348. Cohen S. Positive events and social supports as buffers of life change stress. *Journal of Applied Social Psychology* 1983;**13**:99-125.

349. Cohen S, Underwood LG, Gottlieb BH. Social support measurement and intervention. Oxford: Oxford University Press, 2000.

350. Goodwin P, Leszcz M, Ennis M, Koopmans J, Vincent L, Guther H *et al*. The effect of group psychosocial support on survival in metastatic breast cancer. *NEJM* 2001;**345**:1719-26.

351. Ryff CD,.Singer B. Interpersonal flourishing: A positive health agenda for the new millenium. *Personality and social psychological review* 2000;**4**:30-44.

352. Singer B,.Ryff CD. Hierarchies of life histories and associated health risk. *Annals New York Academy of Sciences* 1999.

353. Baron RS, Cutrona CE, Hicklin D, Russell DW, Lubaroff DM. Social support and immune function among spouses of cancer patients. *J.Pers.Soc.Psychol.* 1990;**59**:344-52.

354. Cutrona CE,.Russell DW. The provisions of social relationships and adaptation to stress. *Advances in Personal Relationships* 1987;**1**:37-67.

355. Cutrona CE, Cole V, Colangelo N, Assouline SG, Russell DW. Perceived parental social support and academic achievement: an attachment theory perspective. *J.Pers.Soc.Psychol.* 1994;**66**:369-78.

356. Russell DW,.Cutrona CE. Social support, stress, and depressive symptoms among the elderly: test of a process model. *Psychol.Aging* 1991;**6**:190-201.

357. Cohen S, Doyle WJ, Skoner DP, Rabin BS, Gwaltney JM. Social ties and susceptibility to the common cold. *JAMA* 1997;**277**:1940-4.

358. Bandura A. Self-efficacy in Changing Societies. Cambridge: Cambridge University Press, 1995.

359. Bandura A. Self-efficacy: toward a unifying theory of behavioral change. *Psychological Review* 1977;**84**:191-215.

360. Bandura A, Cioffi D, Taylor CB, Brouillard ME. Perceived self-efficacy in coping with cognitive stressors and opioid activation. *Journal of Personality & Social Psychology.* 1988;**55**:479-88.

361. Bandura A, O'Leary A, Taylor CB, Gauthier J, Gossard D. Perceived self-efficacy and pain control: opioid and nonopioid mechanisms. *Journal of Personality & Social Psychology.* 1987;**53**:563-71.

362. Bandura A. Guide for constructing self-efficacy scales. *Self-Efficacy Beliefs of Adolescents*, pp 307-37. Information Age Publishing, 2006.

363. Everett B, Salamonson Y, Davidson PM. Bandura's exercise self-efficacy scale: Validation in an Australian cardiac rehabilitation setting. *International Journal of Nursing Studies* 2009;**46**:824-9.

364. Shin Y, Jang H, Pender NJ. Psychometric evaluation of the exercise self-efficacy scale among Korean adults with chronic diseases. *Research in Nursing & Health* 2001;**24**:68-76.

365. Shin YH, Hur HK, Pender NJ, Jang FJ, Kim MS. Exercise self-efficacy, exercise benefits and barriers, and commitment to a plan for exercise among Korean women with osteoporosis and osteoarthritis. *International Journal of Nursing Studies* 2006;**43**:3-10.

366. Kroll T, Kehn M, Ho PS, Groah S. The SCI Exercise Self-Efficacy Scale (ESES): development and psychometric properties. *Int.J.Behav.Nutr.Phys.Act.* 2007;**4**:34.

367. Baer RA, Smith GT, Hopkins J, Krietemeyer J, Toney L. Using self-report assessment methods to explore facets of mindfulness. *Assessment* 2006;**13**:27-45.

368. Baer RA, Smith GT, Lykins E, Button D, Krietemeyer J, Sauer S *et al*. Construct validity of the five facet mindfulness questionnaire in meditating and nonmeditating samples. *Assessment.* 2008;**15**:329-42.

369. Cardaciotto L, Herbert JD, Forman EM, Moitra E, Farrow V. The assessment of present-moment awareness and acceptance: the Philadelphia Mindfulness Scale. *Assessment* 2008;**15**:204-23.

370. Cayoun BA. Towards a four-stage model of mindfulness-based cognitive behaviour therapy for children: A preliminary trial with generalised anxiety disorder. *Australian Journal of Psychology* 2006;**58**:121.

371. Grossman P. Defining mindfulness by how poorly I think I pay attention during everyday awareness and other intractable problems for psychology's (re)invention of mindfulness: Comment on Brown et al. (2011). *Psychol.Assess.* 2011;**23**:1034-40.

372. Lau MA, Bishop SR, Segal ZV, Buis T, Anderson ND, Carlson L *et al*. The Toronto Mindfulness Scale: Development and validation. *J Clin Psychol* 2006;**62**:1445-67.

373. MacCoon DG, Imel ZE, Rosenkranz MA, Sheftel JG, Weng HY, Sullivan JC *et al*. The validation of an active control intervention for Mindfulness Based Stress Reduction (MBSR). *Behav.Res.Ther.* 2011.

374. Walach H, Buchheld N, Buttenmuller V, Kleinknecht N, Schmidt S. Measuring mindfulness - the Freiburg Mindfulness Inventory (FMI). *Personality and Individual Differences* 2006;**40**:1543-55.

375. Barrett BP, Brown RL, Locken K, Maberry R, Bobula JA, D'Alessio D. Treatment of the common cold with unrefined echinacea: A randomized, double-blind, placebo-controlled trial. *Annals of Internal Medicine* 2002;**137**:939-46.

376. Barrett B, Rakel D, Chewning B, Marchand L, Rabago D, Brown R *et al*. Rationale and methods for a trial assessing placebo, echinacea, and doctor-patient interaction in the common cold. *Explore.(NY)* 2007;**3**:561-72.

377. Berk KN,.Lachenbruch PA. Repeated measures with zeroes. *Statistical Methods in Medical Research* 2002;**11**:303-16.

378. Sumathi, K. and Aruna Rao, K. On estimation and tests for zero inflated regression models. 2009. Karnataka, India, Department of Statistics, Mangalore University. Technical Report.

379. Huber, P. J. The behavior of maximum likelihood estimates under non-standard conditions. 221-233. 1967. Berkeley, University of California Press. Proceedings of the Fifth Berkeley symposium on mathematical statistics and probability.

380. White H. Maximum likelihood estimation of misspecified models. *Econometrica* 1982;**50**:1-25.

381. Altman DG. Practical Statistics for Medical Research. London: Chapman & Hall, 1991.

382. Davis CS. Semi-parametric and non-parametric methods for the analysis of repeated measurements with applications to clinical trials. *Statistics in Medicine* 1991;**1**:1959-80.

383. Dillon WR, Goldstein M. Multivariate analysis: Methods and applications. New York: John Wiley & Sons, 1984.

384. Benjamini Y,.Hochberg Y. On the adaptive control of the false discovery rate in multiple testing with independent statistics. *J.Educ.Behav.Stat.* 2000;**25**:60-83.

385. Bloch DA, Lai TL, Su Z, Tubert-Bitter P. A combined superiority and non-inferiority approach to multiple endpoints in clinical trials. *Stat.Med.* 2007;**26**:1193-207.

386. Bloch DA, Lai TL, Tubert-Bitter P. One-sided tests in clinical trials with multiple endpoints. *Biometrics* 2001;**57**:1039-47.

387. Neuhauser M. How to deal with multiple endpoints in clinical trials. *Fundam.Clin.Pharmacol.* 2006;**20**:515-23.

388. Tang DI, Geller NL, Pocock SJ. On the design and analysis of randomized clinical trials with multiple endpoints. *Biometrics* 1993;**49**:23-30.

389. Kalbfleisch JD, Prentice RL. The statistical analysis of failure time data. Hoboken, N.J.: Wiley, 2002.

390. Jiang H, Fine JP. Kaplan Meier Estimator. In Chow S-C, ed. *Encyclopedia of Biopharmaceutical Statistics*, pp 518-23. 2003.

391. Lee ET, Wang JW. Statistical Methods for Survival Data Analysis. Hoboken, N.J.: Wiley, 2004.

392. Diggle PJ, Liang K-Y, Zeger SL. Analysis of Longitudinal Data. Oxford: Oxford University Press, 1994.

393. Liang KY,.Zeger SL. Longitudinal data analysis using generalized linear models. *Biometrika* 1986;**73**:13-22.

394. Zeger SL,.Liang KY. Longitudinal data analysis for discrete and continuous outcomes. *Biometrics* 1986;**42**:121-30.

395. Lipsitz SR, Laird NM, Harrington D.P. Generalized estimating equations for correlated binary data: Using the odds ratio as a measure of association. *Biometrika* 1991;**78**:153-60.

396. Lipsitz SR, Kim K, Zhao L. Analysis of repeated categorical data using generalized estimating equations. *Statistics in Medicine* 1994;**13**:1149-63.

397. Lipsitz SR, Fitzmaurice GM, Orav EJ, Laird NM. Performance of generalized estimating equations in practical situations. *Biometrics* 1994;**50**:270-8.

398. Hardin JW, Hilbe JM. Generalized Estimating Equations. Boca Raton FL: Chapman & Hall / CRC, 2003.

399. Potthoff RF, Tudor GE, Pieper KS, Hasselblad V. Can one assess whether missing data are missing at random in medical studies? *Statistical Methods in Medical Research* 2006;**15**:213-34.

400. Short LM,.Hennessy M. Using structural equations to estimate effects of behavioral interventions. *Structural Equation Modeling* 1994;**1**:68-81.

401. Judd CM,.Kenny DA. Process analysis: Estimating mediation in treatment evaluations. *Evaluation Review* 1981;**5**:602-19.

402. Yuan Y,.MacKinnon DP. Bayesian mediation analysis. *Psychol.Methods* 2009;**14**:301-22.

403. Efron B, Tibshirani RJ. An Introduction to the Bootstrap. Boca Raton, FL: Chapman & Hall/CRC, 1993.

404. Shalowitz D,.Wendler D. Informed consent for research and authorization under the Health Insurance Portability and Accountability Act Privacy Rule: an integrated approach. *Annals of Internal Medicine* 2006;**144**:685-8.

405. Barrett, B., Bobula, J., Bone, K., Brown, R., Chewning, B., DeMets, D. L., Gern, J. E., Marchand, L., Mundt, M. P., Rabago, D., Rakel, D., and Ryff, C. D. Placebo: Physician or Pill? A randomized trial in a common cold model 1-R01-AT-001428-01. A four year clinical trial sponsored by the National Center for Complementary and Alternative Medicine at the National Institutes of Health Responding to RFA: "The Placebo Effect in Clinical Practice" 2003.

406. Blair SN, Kohl III HW, Barlow CE, Paffenbarger Jr RS, Gibbons LW, Macera CA. Changes in physical fitness and all-cause mortality: A prospective study of health and unhealthy men. *JAMA* 1995;**278**:1093-8.

407. Leon AS, Myers MJ, Connett J. Leisure time physical activity and the 16-year risks of mortality from coronary heart disease and all-causes in the Multiple Risk Factor Intervention Trial (MRFIT). *International Journal of Sports Medicine* 1997;**18**:Suppl-15.

408. Smieja M, Castriciano S, Carruthers S, So G, Chong S, Luinstra K *et al*. Development and evaluation of a flocked nasal midturbinate swab for self-collection in respiratory virus infection diagnostic testing. *J.Clin.Microbiol.* 2010;**48**:3340-2.
