# Supplementary material for: Meditation or exercise for preventing acute respiratory infection (MEPARI-2): A randomized controlled trial
Source: PLoS One. 2018 Jun 22;13(6):e0197778. doi: 10.1371/journal.pone.0197778 (PMC6014660; doi:10.1371/journal.pone.0197778)
Supplement: S2 File — (PDF) [file pone.0197778.s003.pdf]

Appendix A: Study Interventions

Mindfulness Meditation

Four current instructors for the longstanding Mindfulness Based Stress Reduction (MBSR) program at University of Wisconsin Integrative Medicine center are available to teach the 8-week meditation sessions for the proposed project. The format and content of the experimental intervention will mimic the current MBSR class structure.

Mindfulness meditation originates from a Buddhist tradition, but in its current formulation requires no particular religious orientation or belief system. It involves systematic training in the development of a sustained, non-aroused state of attention and clear awareness. Mindfulness is a meditation practice that cultivates paying attention in the present moment, bare of judgment, commentary, and decision. It trains the recognition of automatic reactivity such as habituated patterns of behavior, emotional reactivity, momentary distractions and loss of attention, and thus can attenuate stress reactions and other adverse emotional reactivity. MBSR cultivates awareness, stability, insight and choice. Mindfulness includes working with how one relates to environmental stimuli, one's sensitivity to bodily sensations, breathing, states of mind (thinking and emotions), and habitual patterns of automatic reactivity. MBSR training includes the cultivation of attitudes of self-appreciation, compassion, and empathy.

Participants will meet in groups for 2 ½ hour sessions, once per week for 8-weeks. An additional "half day retreat" session will be available to participants, hence the total duration of contact time is about 25 hours per subject. Audiotapes (or compact discs) vary slightly in length, but are no longer than 45 min. Guided instruction leads participants through their formal meditation practice of the body scan, sitting meditation, and gentle yoga. Participants will be requested to practice for 6 days per week at home. Participants are asked to select a daily practice time that works best for them and to record on a log sheet what they practiced and for how long. Classes will normally involve a review of previously assigned homework, allowing for discussion of difficulties participants may have experienced. The class then progresses to new material, employing hands-on practice/experience with new techniques or skills.

The 8-week schedule is outlined on the pages that follow:

The specific activities for each class are listed below:

<table>
<tr><td colspan="3">Week 1:</td></tr>
<tr>
<td>Check In:
• Review of contract and guidelines
• Collect paperwork
• Introduce instructors
• Introduction to program</td>
<td>Presentation Theme:
• There is more right with you than wrong with you
• Problems can be worked with
• Present moment as only time available to change</td>
<td>Homework:
• Tape 1/Side 1 >= 6 days this week
• Peace (handout 1)*
• Week One (handout 2)
• 9 dots exercise (handout 3)
• Getting Started (handout 4)
• Read upstream/downstream fable (handout 5)
• Read “What is Mindfulness?” (handout 6)
• Eat one meal mindfully this week

* All handouts may not appear in these tables due to space considerations.</td>
</tr>
<tr><td colspan="3">Practices:
1. Centering and sharing-group introductions – 60 minutes
2. Introduction to mindfulness through raisin-eating – 20-30 minutes
3. Breath Awareness – 10-15 mins
4. Group comments – 10-20 mins
5. Introduction to Body Scan – 20-45 mins
6. Group discussion and questions – 10-20 mins</td></tr>
</table>

<table>
<tr><td colspan="3">Week 2:</td></tr>
<tr>
<td>Check In:
• Discuss the homework: the 9 dots exercise and the theme of expanding the field of awareness in problem identification and problem solving. Make connection to class</td>
<td>Presentation Theme:
• Perception and creative responding
• how you see things (or don’t see them) will determine in large measure how you will respond to them
• “It’s not the stressors per se but how you handle them” Normalize body scan experience.</td>
<td>Homework:
• Tape 1/Side 1 ≥ 6 times per week
• Sitting meditation: 10-15 minutes per day
• Practice Log: Complete practice log (to hand in to researcher)
• Mindfulness of routine activities: brushing teeth, washing dishes, taking a shower, taking out garbage, shopping, reading to kids, eating.
• Read “To Heal the Body, Heart, and Mind” by Jack Kornfield (handout 9)</td>
</tr>
<tr><td colspan="3">Practices:
1. Introduction to the attitudes
2. Contemplative Reading about the Body Scan (handout 8)
3. Questions about the past week’s experience of the body scan or other practice
4. Practice and revisit the body scan
5. Introduction to the breath and sitting with breathing as primary object of attention. Introduction to first phrase of metta/loving-kindness practice:</td></tr>
</table>

<table>
<tr><td colspan="3">Week 3:</td></tr>
<tr>
<td>Check In:
• Welcome & brief check-in</td>
<td>Presentation Theme:
• Mindfulness within movement
• Hatha yoga</td>
<td>Homework:
• Alternate Side 1 with Side 2 of Tape 1 ≥ 6 days/week
• Week 3 Assignments (handout 10)
• Sitting meditation with AOB 20 min/day
• Introduce pleasant events and pleasant events calendar for the week, one entry per day (handout 11).
• Yoga positions (handout 12)
• True Home Poem (handout 13)
• Practice Log: Complete practice log (to hand in to researcher)
• Make an effort to “capture” your moments during the day.
• Mindfulness of going on “automatic pilot” and under what circumstances it occurs. What pulls you off center? What do you most not want to look at?</td>
</tr>
<tr><td colspan="3">Practices:
1. Introduction to mindful Yoga (handout 9)
2. Observations and Questions of the Practice
3. Learning about sitting on cushions.
4. Guided sitting meditation with focus on awareness of breathing
5. Close with quiet sitting with metta phrase</td></tr>
</table>

<table>
<tr><td colspan="3">Week 4:</td></tr>
<tr>
<td>Check In:
• What learn/discover/ observe with pleasant experiences exercise?</td>
<td>Presentation Theme:
• Learn how to relate to both pleasant and unpleasant experiences
• Reactivity
• Physical pain</td>
<td>Homework:
• Alternate Side 1 with Side 2 ≥ 6 days per week
• Sitting Meditation 20 min per day with AOB, sensations, body as whole.
• Unpleasant Events Calendar (handout 14)
• Pain Diagram (1-3) and articles (handout 15)
• Women Beside the Well (handout 16)
• Week 4 Assignments (handout 17)
• Pausing, acknowledging, allowing, noticing, being (handout 18)
• Practice Log: Complete practice log (to hand in to researcher)</td>
</tr>
<tr><td colspan="3">Practices:
1. Guided yoga
2. Guided sitting meditation
3. Group discussion and inquiry on 1 & 2
4. Short sitting to close class</td></tr>
</table>

**Week 5:**		
**Check In:**<br>• Welcome	**Presentation Theme:**<br>• Deepen our understanding of being with pleasant and unpleasant experiences, specifically as it relates to emotions	**Homework:**<br>• Tape 2/Side 1 Sitting meditation tape. Alternate with either body scan or yoga from Tape 1.<br>• Practice Log: Complete practice log (to hand in to researcher)<br>• Quotes for working with difficult mind states ("reflections"; handout 21)<br>• Working skillfully with mind states (handout 22)<br>• Week 5 Assignments (handout 23)
**Practices:**<br>1. Self-directed yoga<br>2. Sitting meditation<br>3. Group discussion and inquiry on 1 & 2<br>4. Relating to emotions<br>5. Discussion of practice 4		

**Week 6:**		
**Check In:**<br>• Welcome	**Presentation Theme:**<br>• awareness of thinking<br>• Ignore/avoid/grasp/resist	**Homework:**<br>• Alternate Tape 2/Side 1 with Body Scan and/or Yoga from either tape.<br>• Practice Log: Complete practice log (to hand in to researcher)<br>• Week 6 Assignments (handout 24)<br>• Compassion for the conditioned mind (handout 25)<br>• Yoga Part 2 (handout 12)<br>• Changing Karma? (handout 26)<br>• Free within our Thinking (handout 27)<br>• "I vow that in every step…" (handout 28)<br>• Walking Meditation from "Peace is Every Step" (handout 29)<br>• Thinking hit list (handout 30)
**Practices:**<br>1. New yoga practice CD<br>2. Sitting meditation<br>3. Introduction to walking meditation<br>4. Discussion of experiences, questions/answers, for yoga, sitting, and walking meditation.<br>5. Habitual responses demonstration/exercise<br>6. Discussion of up-coming all day session		

The MBSR class includes a half day retreat session during the 6th week , normally held on the Saturday after the sixth class from 9 am to 1:30pm. A typical schedule follows.

WEEK 6 HALF DAY SESSION

9:00 - 9:20	(20 min)	Welcome & overview of day
9:20 – 10:00	(40 min)	Yoga
10:00 – 10:20	(20 min)	Sitting meditation
10:20 – 10:30	(10 min)	Break
10:30 – 11:00	(30 min)	Silent walking meditation
11:00 – 11:30	(30 min)	Loving kindness forgiveness meditation
11:30 – 12:00	(30 min)	Mountain meditation
12:00 – 12:30	(30 min)	Fast walking and laughing meditation
12:30 – 1:30	(1 hour)	Lunch and Wrap up:

<table>
<tr><td colspan="3">Week 7:</td></tr>
<tr><td>Check In:
• Welcome</td><td>Presentation Theme:
• Making the practice personal</td><td>Homework:
▪ No tape. Practice the formal practice on one's own best one can.
▪ Practice Log: Complete practice log (to hand in to researcher)
▪ Evaluation form (handout 31)
▪ Bringing the practices together (handout 32)
▪ Week 7 Assignments (handout 33)</td></tr>
<tr><td colspan="3">Practices:
1. Walking meditation
2. Sitting meditation with choiceless awareness
3. Discussion of all-day experience
4. Reactivity of mind discussion
5. Metta/loving-kindness practice
6. Revisiting forgiveness practice</td></tr>
</table>

<table>
<tr><td colspan="3">Week 8:</td></tr>
<tr><td>Check In:
• Welcome</td><td>Presentation Theme:
• The eighth week is the rest of your life
• Keeping up the momentum & discipline</td><td>Homework:
▪ Go back to the tapes, using whichever techniques you wish. Keep up the practice and make it your own.
▪ Practice Log: Complete daily practice log until follow-up</td></tr>
<tr><td colspan="3">Practices:
1. Self-directed yoga
2. Walking meditation
3. Sitting meditation
4. Closing reflections
5. Group sharing about closing reflections
6. Continuing mindfulness in everyday life</td></tr>
</table>

Teaching Staff Profiles

Katherine Bonus:

- Established UW MBSR program in 1993 with UW Health Cardiology
- Completed the Professional Training Program with Jon Kabat Zinn and Saki Santorelli through the Mindfulness Based Stress Reduction Clinic, University of Massachusetts Medical Center
- Has taught adult education classes for 20 years

Diana Grove:

- Completed the Professional Training Program with Jon Kabat Zinn and Saki Santorelli through the Mindfulness Based Stress Reduction Clinic, University of Massachusetts Medical Center
- Has taught adult education classes for 20 years

Cindy McCallum

- Completed the Professional Training Program with Jon Kabat Zinn and Saki Santorelli through the Mindfulness Based Stress Reduction Clinic, University of Massachusetts Medical Center
- Completed training with Spirit Rock Meditation Center, Woodacre, CA and Forest Way Insight Meditation Center, Virginia
- Certified Social Worker

Laura Pinger

- Completed the Professional Training Program with Jon Kabat Zinn and Saki Santorelli through the Mindfulness based Stress Reduction Clinic, University of Massachusetts Medical Center
- Internal Family Systems Therapy training
- Certification in yoga teacher training

These are expected mindfulness instructors, but there may be changes. All mindfulness (and exercise) instructors will received appropriate human subjects research training and be named to IRB HSC.

Exercise

The exercise intervention behavioral modification program will be an 8-week individually-tailored program consisting of weekly 2½ hour group sessions and 45 minutes of exercise the other days of the week. Participants will attend 2½ hour sessions for 8 consecutive weeks. The group sessions will be led by exercise specialists experienced in leading groups. At time of writing, we expect that Jude Sullivan and Lisa Millbrandt will lead the exercise training sessions. Mr. Sullivan has a Masters degree in exercise physiology and Ms. Sullivan has a Bachelors degree. Both are licensed athletic trainers. Each has more than 20 years of experience in leading exercise training groups in both research and non-research settings.

Exercise sessions will include approximately 1½ hours addressing cognitive behavioral issues in a conference room and approximately 1 hour of group exercise in the UW Sports Medicine Fitness Center. The cognitive behavioral portion will consist of a check-in period to review the previous week's activities, a brief presentation on exercise, a discussion of behavioral change principles and activities and a wrap-up. The exercise portion will involve a warm-up (5-10 min), aerobic activity (work up to 30-40 min), and cool-down and stretching (10 min). Participants will be asked to exercise 45 minutes each day on their own. These sessions will consist of 5 minute warm-up, 30-35 min aerobic exercise and 5-10 min cool-down and stretching.

The mode of exercise for most participants will be brisk walking and or jogging. These activities are convenient and do not require special equipment. Participants will be exposed to other activities that they can do at home and programs will be developed for participants who have access to exercise equipment. For those unable or unwilling to walk or jog, alternative exercise strategies will be employed. For those with access to appropriate facilities/ equipment, swimming or bicycling (stationary or mobile) may be selected. Alternatively, dance could be chosen as the primary exercise. It is expected that most participants will choose a primary means of achieving moderate intensity exercise, and that walking or jogging will fulfill that role most often. However, the focus of the training will be on achieving and maintaining a regular exercise pattern that suits the lifestyle of the individual, with the goal of continued lifelong practice. Mixing two or more exercise modalities will be perfectly acceptable.

For both the supervised sessions and home exercise, Borg's Rating of Perceived Exertion (RPE) (1) will be used to monitor intensity. RPE provides a good estimate of exercise intensity particularly in individuals who have not had a graded exercise test and those who may be taking medications that affect heart rate response to exercise (2). Participants will be asked to exercise at an RPE of 12-1c on the 6 to 20 scale. This intensity, corresponding to a rating of "somewhat hard" to "hard," is associated with physiologic adaptation to exercise (3).

To monitor adherence to the 8-week program, participants will be asked to keep an exercise log. The log will include date, activity, duration and RPE. Participants will turn their logs in each week. Program staff will review the logs and help participants develop strategies for adhering to the protocol. After the 8-week session, participants will continue to complete exercise logs. These will be mailed in every 2 weeks. In addition participants will be called every two weeks by program staff to assess their frequency and duration of exercise. Also, participants will be asked to complete and objective self-report physical activity questionnaire (International Physical Activity Questionnaire-IPAQ) (3) at baseline, ate the end of the 8-week intervention, and then each month for 6 months.

The specific activities for each class are listed on the following pages:

Week 1:

**Check In:**	**Presentation:**	**Behavior Change:**	**Behavior Challenge:**
• Ice Breaker<br>• Ground Rules	• FIT Principle – core basics of exercise program, pedometer use, RPE scale use<br>• What you need to know - indoor/outdoor venue choices, clothes, shoes	• Getting Started	• Introduce and use tracking sheet

Exercise:
- Warm Up – 5-10 minutes
- Aerobic Activity – 15 minutes; RPE=12-14 or what is tolerated
- Reconvene – 10 minutes; review pedometer, RPE scale use, tracking sheet and other issues
- Aerobic Activity – 15 minutes; RPE=12-14 or what is tolerated
- Cool Down: 10 minutes; stretching

Week 2:

**Check In:**	**Presentation:**	**Behavior Change:**	**Behavior Challenge:**
• Review previous week’s activities	• Why is exercise important – health, fitness, exercise physiology	• Goal Setting	• Set some goals

Exercise:
- Warm Up – 5-10 minutes
- Aerobic Activity – 30 minutes
- Functional Fitness – 10 minutes; balance intro – stepping/standing in free space
- Cool Down: 10 minutes; stretching

Week 3:

**Check In:**	**Presentation:**	**Behavior Change:**	**Behavior Challenge:**
• Review previous week’s activities	• Variety is the spice of life	• Having different modalities/environments	• Experiment with different modalities/environments

Exercise:
- Warm Up – 5 minutes
- Aerobic Activity – 35 minutes
- Functional Fitness – 10 minutes; individual medicine ball intro – Sagittal and Frontal plane
- Cool Down: 10 minutes; stretching

Week 4:

**Check In:**	**Presentation:**	**Behavior Change:**	**Behavior Challenge:**
• Review previous week’s activities	• Injury prevention/dealing with injuries	• Relapse prevention	• Allow yourself to begin again

Exercise:
- Warm Up – 5 minutes
- Aerobic Activity – 40 minutes
- Functional Fitness – 10 minutes; partner cable strength intro
- Cool Down: 5 minutes; stretching

**Week 5:**			
**Check In:**<br>• Review previous week's activities	**Presentation:**<br>Energy expenditure (use of tables)	**Behavior Change:**<br>• Identification of social support	**Behavior Challenge:**<br>• Develop a reliable social network
**Exercise:**<br>• Warm Up – 5 minutes<br>• Aerobic Activity – 40 minutes<br>• Functional Fitness – 10 minutes; balance progression ideas – stepping w/hurdles<br>• Cool Down: 5 minutes; stretching			

**Week 6:**			
**Check In:**<br>• Review previous week's activities	**Presentation:**<br>• Fitting in activity/exercise	**Behavior Change:**<br>• Finding Time/Making Time	**Behavior Challenge:**<br>• Adjust goals to align better with study parameters based on experience to this point
**Exercise:**<br>• Warm Up – 5 minutes<br>• Aerobic Activity – 40 minutes<br>• Functional Fitness – 10 minutes; medicine ball progression ideas – Sagittal, Frontal and Transverse plane<br>• Cool Down: 5 minutes; stretching			

**Week 6: Weekend half day retreat**			
**Group discussion:**<br>• Review progress to date in achieving and maintaining regular exercise<br>30 minutes	**Individual exercise:**<br>Walking / jogging / stationary bicycling / Weight training<br><br>30 minutes	**Presentation:**<br>• Physiological changes that accompany exercise<br><br>25 minutes	**Healthy snack:**<br>Fruits and vegetables<br>Bagels, muffins, cereal<br>Water, milk, juice, tea<br><br>20 minutes
**Presentation:**<br>• Immunity, infectious disease, and exercise<br><br>25 minutes	**Group exercise:**<br>Flexibility, balance and agility exercises<br>**Group discussion:**<br>Working with others (groups or pairs) to enhance exercise<br>30 minutes	**Presentation:**<br>Indoors vs. outdoors activities, buying exercise equipment, choosing health clubs, finding personal trainers.<br><br>20 minutes	**Group discussion:**<br>Building individual and group exercise into a sustainable and fulfilling lifestyle<br><br>30 minutes
**We will plan to begin at 8am and serve a healthy lunch around 12:30 after the morning's activities**			

**Week 7:**			
**Check In:**<br>• Review previous week's activities	**Presentation:**<br>• Turn up the volume	**Behavior Change:**<br>• Expand the program	**Behavior Challenge:**<br>• Knowing when it is OK to push
**Exercise:**<br>• Warm Up – 5 minutes<br>• Aerobic Activity – 40 minutes<br>• Functional Fitness – 10 minutes; partner cable progression ideas – combination balance and multi-planar movement<br>• Cool Down: 5 minutes; stretching			

**Week 8:**			
**Check In:**<br>• Review previous week's activities	**Presentation:**<br>• Wrap up	**Behavior Change:**<br>• Evaluate progress	**Behavior Challenge:**<br>• Readjust goals based on experience to this point
**Exercise:**<br>• Warm Up – 5 minutes<br>• Aerobic/Functional Fitness Circuit – 50 minutes<br>• Cool Down: 5 minutes; stretching			

References:

1. Borg GV, Linderholm H. Perceived exertion and pulse rate during graded exercise in various age groups. Acta Medica Scandinavica 1970; 472 (suppl): 194-206.

2. Morgan W, Borg GA. Perception of effort in the prescription of physical activity. Nelson T, ed. Mental Health and Emotional Aspects of Sports. Chicago: American Medical Association, 1976: 126-129.

3. Pollock M, Gaesser GA, Butcher JD, Despres JP, Dishman RK, Franklin BA, Garber CE. The recommended quantity and quality of exercise for developing and maintaining cardiorespiratory and muscular fitness, and flexibility in healthy adults. Med Sci Sports Exerc 1998; 30: 975-991.

Appendix B: Questionnaire Instruments

(uploaded individually into ARROW application)

Wisconsin Upper Respiratory Symptom Survey (WURSS-24)
Medication use during ARI
Alcohol and Tobacco Use Report Form (TimeLine Followback)
Demographics
Seattle Index of Co-Morbidity (SIC)
Big Five Inventory (BFI)
Health Care Utilization (HCU) – participant
Health Care Utilization (HCU) – study personnel
Stanford Presenteeism Scale General (StPS-G)
Stanford Presenteeism Scale Illness (StPS-I)
PHQ-9 (Depression screen)
General physical and mental health (SF-12)
Pittsburgh Sleep Quality Index (PSQI)
Positive and Negative Affect Schedule (PANAS)
Perceived Stress Scale (Cohen PSS-10)
Social Provisions Scale (SPS)
Social Network Index (SNI)
Being Loved (BL)
Exercise Self-Efficacy Scale (ESES)
Mindfulness-Based Self Efficacy Scale (MSES)
Mindfulness Attention Awareness Scale (MAAS)
Global Physical Activity Questionnaire (GPAQ)
Expectancy Ratings (Pre)
Expectancy Ratings (Post)
Meditation Log
Exercise Log
Jackson Scale

BIG FIVE INVENTORY

Computing Simple BFI Scale Scores
1=Disagree Strongly; 2=Disagree A Little; 3=Neither Agree or Disagree; 4=Agree a Little; 5=Agree Strongly

Reverse score the items labeled "R" and compute scale scores as the mean of the following items:

Extraversion (8 items): 1, 6R, 11, 16, 21R, 26, 31R, 36
Agreeableness (9 items): 2R, 7, 12R, 17, 22, 27R, 32, 37R, 42
Conscientiousness (9 items): 3, 8R, 13, 18R, 23R, 28, 33, 38, 43R
Neurotiscism (8 items): 4, 9R, 14, 19, 24R, 29, 34R, 39
Openness (10 items): 5, 10, 15, 20, 25, 30, 35R, 40, 41R, 44

Computing the content-balanced acquiesence index and Ipsatizing the BFI items
(Ipsatizing looks at the degree to which a participant answers pre-paired "opposite" characteristic questions in opposite directions.)
SPSS sytnax: compute within person response means [of raw score] and standard deviations for 3,8,9,13,18,19,23,24,28,29,34,39 as a Z-score.
Conscientiousness: 3 and 43, 8 and 13; 18 and 33; 23 and 28

Neurotiscism: 9 and 19; 24 and 29; 34 and 39

Instrument is administered at Run-in and Exit

From the MEPARI Protocol Manual 07-18-12 (p. 13, 16)
6.4.5 Big Five Inventory (BFI) Research on personality and health has been underway for some time, leading to various conceptual structures of state and trait psychological domains. The Big Five taxonomy has helped clarify and organize the links between personality, health behaviors, illness and mortality across the lifespan. Of the five dimensions measured (openness, conscientiousness, extraversion, agreeableness, and neuroticism), we will use baseline “conscientiousness” and “neuroticism” scores on the Big Five Inventory to gauge propensity for self-report bias on instrument completion activities, and to control for between person differences in multivariate efficacy models.

Covariate to control for between-person variability

John OP, Naumann LP, Soto CJ. Paradigm shift to the integrative big-five trait taxonomy: History, measurement and conceptual issues. In John OP, Robins RW, Pervin LA, eds. *Handbook of Personality: Theory and Research* , pp 114-58. New York: Guilford Press, 2008.

BIOMARKERS

Interleukin 6 secondary outcome, potential mediator
Interleukin 8 secondary outcome, potential mediator
IFN-induced Protein 10 secondary outcome, potential mediator
C-reactive Protein secondary outcome, potential mediator
Procalcitonin secondary outcome, potential mediator
Neutrophil potential mediator
HgA1C secondary outcome
Viral Identification secondary outcome, potential mediator

Outcomes: Blood and nasal wash samples will be obtained at baseline, one month after the end of the 8-week interventions, and once again three months later. Blood and nasal wash samples will be obtained approximately 24-72 hours into each ARI episode. Nasal wash samples will be tested with multiplex PCR (polymerase chain reaction) to identify etiological agents. Serum and nasal wash will be analyzed for interleukin-6, interleukin-8, C-reactive protein, procalcitonin, and interferon-gamma-induced protein 10. These inflammatory biomarkers will serve as objective indicators of disease severity to compare with illness severity self-reported on the WURSS-24 ... Inflammatory biomarkers ... will be analyzed as potential mediators of causal pathways leading from behavioral training interventions to ARI illness outcomes.

6.3.4 Viral identification will be done in Dr. Gern's lab, where high-throughput PCR-based multiplex methods have been developed and authenticated, and are able to identify nearly all of the pathogens associated with ARI illness [36;261-264] Trial will assess 2 samples, one done by self-swab at home, and the other by nasal wash at lab. We will also improve sample processing and include newly developed viral types. Dr. Gern's published data report that up to 91.4% of nasal washes from community-acquired ARI can yield positive viral IDs.[265]

6.3.5 Pro-inflammatory cytokines Laboratory-assessed objective measures will primarily serve to corroborate self-reports of disease severity. C-reactive protein (CRP) and procalcitonin (PCT) are well-established indicators of disease severity during respiratory infection, and can be measured in serum as well as in nasal wash.[113;115;118-120] Concentrations of interleukin-6 (IL-6)[266-271] and interleukin-8 (IL-8)[272-276] in nasal wash have been shown to correlate with illness severity. More recently, interferon-gamma-induced protein 10 (IP-10) has been shown to be measurably increased in both serum and nasal wash during times of acute viral ARI.[122-128] Inflammatory cytokines will be measured by ELISA methods in laboratories directed by Dr. Coe and Dr. Hayney.

6.3.6 Inflammatory tendency The same array of pro-inflammatory cytokines will also be analyzed as indicators of low level inflammation or pro-inflammatory tendency and as potential mediators of effects of behavioral interventions on ARI illness incidence, duration, and severity. The importance of CRP, PCT and IL-6 has been underscored by the ability of these pro-inflammatory biomarkers to predict mortality.[277-285] As potential mediators, pro-inflammatory cytokines (CRP, PCT, IL-6, IL-8, IP-10) will be assessed as change from baseline to one month after the 8 week behavioral interventions finish. Repeating these assays 3 months later will assess whether potential pro-inflammatory changes resulting from interventions will be sustained.

6.3.7 Polymorphonuclear neutrophil count in nasal mucus is a relatively well-established indicator of inflammation of the nasal epithelium.[286-290] Neutrophil counts correlate to symptom severity, viral titer and cytokine levels.[174;291] ... Neutrophil counts will be done on nasal wash collected during ARI episodes.

6.3.8 Glycosylated hemoglobin (HgA1C) Regular exercise is known to reduce hemoglobin A1C, a widely accepted indicator of average blood glucose levels.[142;292-294] There are at least two preliminary reports suggesting that mindfulness meditation might reduce HgA1C.[295;296] To explore these possibilities, we will assess HgA1C at baseline, 1 month after interventions, and again 3 months later.

For IL-6, a value of 0.01 indicates a level below detectable concentration, and for IP-10, a value of 1 indicates a level below detectable concentration.

BLOOD PRESSURE

Blood pressure is taken at Baseline, December, March and Exit visits.

American Heart Association …

Category	systolic, mmHg	diastolic, mmHg
Hypotension	<90	<60
Desired	90-119	60-79
Prehypertension	120-139	80-89
Stage 1 Hypertension	140-159	90-99
Stage 2 Hypertension	160-179	100-109
Hypertensive Crisis	$\geq$ 180	> 110

From the MEPARI Protocol Manual 07-18-12 (p. 15, p. 20)

6.4.24 Blood pressure Blood pressure is a well-recognized health indicator. There is some reason to believe that stress reduction or regular exercise might reduce blood pressure. In this study, blood pressure will be assessed at baseline and at both standardized follow-up periods using standard calibrated sphygmomanometers. Blood pressure will be analyzed as a secondary outcome using methods described in Section 7.

7.2.6 Secondary efficacy analyses Influence of interventions on secondary outcomes will be assessed using ANOVA-based multivariate regression models using SAS software.381-383 Adjustment for multiple comparisons will be incorporated, and interpretation will be cautious.238;384-388 In general, we will want to see relationships with p<0.01 in order to justify tentative null hypothesis rejection. Pre-planned secondary efficacy analyses will include effects of interventions on: ... 9) blood pressure ... 7.2.6.9 Those in the intervention groups will have lower blood pressure compared to control.

Secondary outcome

Expectancy Exit

Participant ID ______________________

1. Do you believe that the [exercise|meditation] training you received helped protect you from colds and flu this year? Although you did not receive meditation or exercise training, do you believe that participating in this study influenced your cold and flu episodes this year?

☐ Yes ☐ No

2. Compared to cold and flu episodes you have had in past years, do you think that your cold and flu episodes this year were:

☐ much worse
☐ somewhat worse
☐ slightly worse
☐ the same
☐ slightly better
☐ somewhat better
☐ much better
☐ very much better

Expectancy Pre-randomization

Participant ID ______________________________

1. Do you believe that regular exercise can help protect you from colds and flu?
   1 ☐ Yes 0 ☐ No

2. If you receive exercise training as part of this study, compared to cold and flu episodes you have had in past years, do you expect that your cold and flu episodes this year will be:
   1 ☐ much worse
   2 ☐ somewhat worse
   3 ☐ slightly worse
   4 ☐ the same
   5 ☐ slightly better
   6 ☐ somewhat better
   7 ☐ much better
   8 ☐ very much better

3. Do you believe that mindfulness meditation can help protect you from colds and flu?
   1 ☐ Yes 0 ☐ No

4. If you receive meditation training as part of this study, compared to cold and flu episodes you have had in past years, do you expect that your cold and flu episodes this year will be:
   1 ☐ much worse
   2 ☐ somewhat worse
   3 ☐ slightly worse
   4 ☐ the same
   5 ☐ slightly better
   6 ☐ somewhat better
   7 ☐ much better
   8 ☐ very much better

Expectancy Post-randomization

Participant ID ______________________________

Given that you will [not] receive [exercise|meditation] training, compared to cold or flu illness episodes you have had in past years, do you expect that your cold and flu episodes this year will be:

1 ☐ much worse
2 ☐ somewhat worse
3 ☐ slightly worse
4 ☐ the same
5 ☐ slightly better
6 ☐ somewhat better
7 ☐ much better
8 ☐ very much better

Expectancy Post-intervention

Participant ID ______________________________

Given that you [did not] receive[d] [exercise|meditation] training, compared to cold or flu illness episodes you have had in past years, do you expect that your cold and flu episodes this year will be:

1 ☐ much worse
2 ☐ somewhat worse
3 ☐ slightly worse
4 ☐ the same
5 ☐ slightly better
6 ☐ somewhat better
7 ☐ much better
8 ☐ very much better

Expectancy Exit

Participant ID ______________________________

1. Do you believe that the [exercise|meditation] training you received helped protect you from colds and flu this year? Although you did not receive meditation or exercise training, do you believe that participating in this study influenced your cold and flu episodes this year?

1 ☐ Yes 0 ☐ No

2. Compared to cold and flu episodes you have had in past years, do you think that your cold and flu episodes this year were:

1 ☐ much worse
2 ☐ somewhat worse
3 ☐ slightly worse
4 ☐ the same
5 ☐ slightly better
6 ☐ somewhat better
7 ☐ much better
8 ☐ very much better

Expectancy Pre-randomization

Participant ID ______________________

1. Do you believe that regular exercise can help protect you from colds and flu?
☐ Yes ☐ No

2. If you receive exercise training as part of this study, compared to cold and flu episodes you have had in past years, do you expect that your cold and flu episodes this year will be:
☐ much worse
☐ somewhat worse
☐ slightly worse
☐ the same
☐ slightly better
☐ somewhat better
☐ much better
☐ very much better

3. Do you believe that mindfulness meditation can help protect you from colds and flu?
☐ Yes ☐ No

4. If you receive meditation training as part of this study, compared to cold and flu episodes you have had in past years, do you expect that your cold and flu episodes this year will be:
☐ much worse
☐ somewhat worse
☐ slightly worse
☐ the same
☐ slightly better
☐ somewhat better
☐ much better
☐ very much better

Expectancy Post-randomization

Participant ID ______________________

Given that you will [not] receive [exercise|meditation] training, compared to cold or flu illness episodes you have had in past years, do you expect that your cold and flu episodes this year will be:
☐ much worse
☐ somewhat worse
☐ slightly worse
☐ the same
☐ slightly better
☐ somewhat better
☐ much better
☐ very much better

Expectancy Post-intervention

Participant ID ________________________

Given that you [did not] receive[d] [exercise|meditation] training, compared to cold or flu illness episodes you have had in past years, do you expect that your cold and flu episodes this year will be:

- ☐ much worse
- ☐ somewhat worse
- ☐ slightly worse
- ☐ the same
- ☐ slightly better
- ☐ somewhat better
- ☐ much better
- ☐ very much better

Expectancy Exit

Participant ID ________________________

1. Do you believe that the [exercise|meditation] training you received helped protect you from colds and flu this year? Although you did not receive meditation or exercise training, do you believe that participating in this study influenced your cold and flu episodes this year?

☐ Yes ☐ No

2. Compared to cold and flu episodes you have had in past years, do you think that your cold and flu episodes this year were:

- ☐ much worse
- ☐ somewhat worse
- ☐ slightly worse
- ☐ the same
- ☐ slightly better
- ☐ somewhat better
- ☐ much better
- ☐ very much better

Expectancy Post-intervention

Participant ID ______________________________

Given that you [did not] receive[d] [exercise|meditation] training, compared to cold or flu illness episodes you have had in past years, do you expect that your cold and flu episodes this year will be:

- ☐ much worse
- ☐ somewhat worse
- ☐ slightly worse
- ☐ the same
- ☐ slightly better
- ☐ somewhat better
- ☐ much better
- ☐ very much better

Expectancy Exit

Participant ID ______________________________

1. Do you believe that the [exercise|meditation] training you received helped protect you from colds and flu this year? Although you did not receive meditation or exercise training, do you believe that participating in this study influenced your cold and flu episodes this year?

☐ Yes ☐ No

2. Compared to cold and flu episodes you have had in past years, do you think that your cold and flu episodes this year were:

- ☐ much worse
- ☐ somewhat worse
- ☐ slightly worse
- ☐ the same
- ☐ slightly better
- ☐ somewhat better
- ☐ much better
- ☐ very much better

Expectancy Post-intervention

Participant ID ______________________________

Given that you [did not] receive[d] [exercise|meditation] training, compared to cold or flu illness episodes you have had in past years, do you expect that your cold and flu episodes this year will be:

- ☐ much worse
- ☐ somewhat worse
- ☐ slightly worse
- ☐ the same
- ☐ slightly better
- ☐ somewhat better
- ☐ much better
- ☐ very much better

Expectancy Post-randomization

Participant ID ______________________________

Given that you will [not] receive [exercise|meditation] training, compared to cold or flu illness episodes you have had in past years, do you expect that your cold and flu episodes this year will be:

- ☐ much worse
- ☐ somewhat worse
- ☐ slightly worse
- ☐ the same
- ☐ slightly better
- ☐ somewhat better
- ☐ much better
- ☐ very much better

Expectancy Pre-randomization

Participant ID ______________________

1. Do you believe that regular exercise can help protect you from colds and flu?
   ☐ Yes ☐ No

2. If you receive exercise training as part of this study, compared to cold and flu episodes you have had in past years, do you expect that your cold and flu episodes this year will be:
   ☐ much worse
   ☐ somewhat worse
   ☐ slightly worse
   ☐ the same
   ☐ slightly better
   ☐ somewhat better
   ☐ much better
   ☐ very much better

3. Do you believe that mindfulness meditation can help protect you from colds and flu?
   ☐ Yes ☐ No

4. If you receive meditation training as part of this study, compared to cold and flu episodes you have had in past years, do you expect that your cold and flu episodes this year will be:
   ☐ much worse
   ☐ somewhat worse
   ☐ slightly worse
   ☐ the same
   ☐ slightly better
   ☐ somewhat better
   ☐ much better
   ☐ very much better

Expectancy Post-randomization

Participant ID ______________________

Given that you will [not] receive [exercise|meditation] training, compared to cold or flu illness episodes you have had in past years, do you expect that your cold and flu episodes this year will be:

☐ much worse
☐ somewhat worse
☐ slightly worse
☐ the same
☐ slightly better
☐ somewhat better
☐ much better
☐ very much better

Expectancy Pre-randomization

Participant ID ______________________

1. Do you believe that regular exercise can help protect you from colds and flu?

☐ Yes ☐ No

2. If you receive exercise training as part of this study, compared to cold and flu episodes you have had in past years, do you expect that your cold and flu episodes this year will be:

☐ much worse
☐ somewhat worse
☐ slightly worse
☐ the same
☐ slightly better
☐ somewhat better
☐ much better
☐ very much better

3. Do you believe that mindfulness meditation can help protect you from colds and flu?

☐ Yes ☐ No

4. If you receive meditation training as part of this study, compared to cold and flu episodes you have had in past years, do you expect that your cold and flu episodes this year will be:

☐ much worse
☐ somewhat worse
☐ slightly worse
☐ the same
☐ slightly better
☐ somewhat better
☐ much better
☐ very much better

FEELING LOVED

Please answer **all four** of following questions.

1 **Do you feel loved?** O Yes O No

Please indicate how loved you feel, on average, on the scale below.

Simply put an "X" on the scale at the point that best captures how loved you feel.

2 **How loved do you feel?**

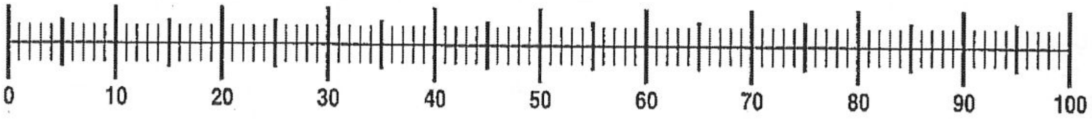

Not at all — *Very, very much*

Do not write in this area

0	0	0
1	1	1
	2	2
	3	3
	4	4
	5	5
	6	6
	7	7
	8	8
	9	9

3 **Do you love yourself?** O Yes O No

Please indicate how much you love yourself, on average, on the scale below.

Again, simply put an "X" on the scale at the point that best captures how much you love yourself.

4 **How much do you love yourself?**

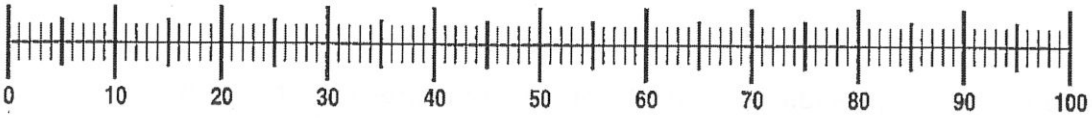

Not at all — *Very, very much*

Do not write in this area

0	0	0
1	1	1
	2	2
	3	3
	4	4
	5	5
	6	6
	7	7
	8	8
	9	9

Flow Sheet - Baseline

Participant ID ______________________

Visit Date ______________________

Height (inches) ______________________

Weight (pounds) ______________________

Vital Signs

Temperature (°C) ______________________

Pulse (per min) ______________________

Respiration (per min) ______________________

Blood Pressure (mm/Hg) ______________________

	1	0
Blood Draw	☐ Yes	☐ No
Nasal wash/Neti Pot used/nasal sprays 24 hours prior to visit?	☐ Yes	☐ No
Cold Symptoms?	☐ Yes	☐ No

Cold Visit Procedures

Nasal Swab 1 ☐ Yes 0 ☐ No

Nasal Swab Date obtained ______________________

Nasal Lavage? 1 ☐ Yes 0 ☐ No

Flow Sheet - Baseline

Participant ID ______________________

Visit Date ______________________

Height (inches) ______________________

Weight (pounds) ______________________

Vital Signs

Temperature (°C) ______________________

Pulse (per min) ______________________

Respiration (per min) ______________________

Blood Pressure (mm/Hg) ______________________

Blood Draw ☐ Yes ☐ No

Nasal wash/Neti Pot used/nasal sprays 24 hours prior to visit? ☐ Yes ☐ No

Cold Symptoms? ☐ Yes ☐ No

Cold Visit Procedures

Nasal Swab ☐ Yes ☐ No

Nasal Swab Date obtained ______________________

Nasal Lavage? ☐ Yes ☐ No

Global Physical Activity Questionnaire

Directions

The following asks you about the time you spend doing different types of physical activity in a typical week.

Please answer these questions even if you do not consider yourself to be a physically active person.

In answering the following questions 'vigorous-intensity activities' are activities that require hard physical effort and cause large increases in breathing or heart rate, 'moderate-intensity activities' are activities that require moderate physical effort and cause small increases in breathing or heart rate.

Activity at work

Think first about the time you spend **doing work**. Think of work as the things that you have to do such as paid or unpaid work, study/training, household chores, yard work, etc.

1 Does your work involve **vigorous-intensity activity** that causes large increases in breathing or heart rate like carrying or lifting heavy loads, digging or construction work for at least 10 minutes continuously?

1 ○ Yes 2 0 * ○ No (if no, skip to question 2).

1a In a typical week, how many **days** do you do **vigorous-intensity** activities as part of your work?

Number of days

0	1	2	3	4	5	6	7
○	○	○	○	○	○	○	○
0	1	2	3	4	5	6	7

1b How much **time** do you spend doing **vigorous-intensity** activities at work on a typical day?

Hours : Minutes [___]___] : [___]___]

Hours **minutes**

HOUR		MIN	
0	0	0	0
1	1	1	1
	2	2	2
	3	3	3
	4	4	4
	5	5	5
	6	6	6
	7	7	7
	8	8	8
	9	9	9

2 Does your work involve **moderate-intensity activity** that causes small increases in breathing or heart rate such as brisk walking [or carrying light loads] for at least 10 minutes continuously?

1 ○ Yes 2 0 * ○ No (if no, skip to question 3).

2a In a typical week, how many **days** do you do **moderate-intensity** activities as part of your work?

Number of days

0	1	2	3	4	5	6	7
○	○	○	○	○	○	○	○
0	1	2	3	4	5	6	7

2b How much **time** do you spend doing **moderate-intensity** activities at work on a typical day?

Hours : Minutes [___]___] : [___]___]

Hours **minutes**

HOUR		MIN	
0	0	0	0
1	1	1	1
	2	2	2
	3	3	3
	4	4	4
	5	5	5
	6	6	6
	7	7	7
	8	8	8
	9	9	9

Travel to and from places

The next questions exclude the physical activities at work that you have already mentioned. Think about the usual way you travel to and from places; for example, going to work, for shopping, or to a place of worship.

3 Do you **walk or use a bicycle** for at least 10 minutes continuously to get to and from places?

1 ○ Yes 2 0 * ○ No (if no, skip to question 4).

PLEASE DO NOT WRITE IN THIS AREA

3a In a typical week, how many **days** do you **walk or bicycle** for at least 10 minutes continuously to get to and from places?

Number of days

0	1	2	3	4	5	6	7
○	○	○	○	○	○	○	○

0 1 2 3 4 5 6 7

3b How much **time** do you spend **walking or bicycling** for travel on a typical day?

Hours : Minutes [__]__] : [__]__]
Hours **minutes**

HOUR		MIN	
0	0	0	0
1	1	1	1
	2	2	2
	3	3	3
	4	4	4
	5	5	5
	6	6	6
	7	7	7
	8	8	8
	9	9	9

Recreational activities

The next questions exclude the work and travel activities already mentioned. Consider the sports, fitness and recreational activities (leisure) you do.

4 Do you do any **vigorous-intensity** sports, fitness or recreational (leisure) activities that cause large increases in breathing or heart rate like [running, soccer, basketball, aerobic exercise] for at least 10 minutes continuously?

1 ○ Yes 2 0 ○ No (if no, skip to question 5).

4a In a typical week, how many **days** do you do **vigorous-intensity** sports, fitness or recreational (leisure) activities?

Number of days

0	1	2	3	4	5	6	7
○	○	○	○	○	○	○	○

0 1 2 3 4 5 6 7

4b How much **time** do you spend doing **vigorous-intensity** sports, fitness or recreational (leisure) activities on a typical day?

Hours : Minutes [__]__] : [__]__]
Hours **minutes**

HOUR		MIN	
0	0	0	0
1	1	1	1
	2	2	2
	3	3	3
	4	4	4
	5	5	5
	6	6	6
	7	7	7
	8	8	8
	9	9	9

5 Do you do any **moderate-intensity** sports, fitness or recreational (leisure) activities that cause a small large increase in breathing or heart rate such as brisk walking, cycling, swimming, or volleyball, for at least 10 minutes continuously?

1 ○ Yes 2 0 ○ No (if no, skip to question 6).

5a In a typical week, how many **days** do you do **moderate-intensity** sports, fitness or recreational (leisure) activities?

Number of days

0	1	2	3	4	5	6	7
○	○	○	○	○	○	○	○

0 1 2 3 4 5 6 7

5b How much **time** do you spend doing **moderate-intensity** sports, fitness or recreational (leisure) activities on a typical day?

Hours : Minutes [__]__] : [__]__]
Hours **minutes**

HOUR		MIN	
0	0	0	0
1	1	1	1
	2	2	2
	3	3	3
	4	4	4
	5	5	5
	6	6	6
	7	7	7
	8	8	8
	9	9	9

Sedentary behavior

The following is about **sitting or reclining** at work, at home, getting to and from places, or with friends including time spent sitting at a desk, working on a computer, sitting with friends, travelling in a car or bus, reading, doing crafts (knitting, crocheting, needlepoint, etc.), playing cards or watching TV. **Do not include time spent sleeping.**

6 How much time do you usually spend sitting or reclining on a typical day?

Hours : Minutes [__]__] : [__]__]
Hours **minutes**

HOUR		MIN	
0	0	0	0
1	1	1	1
	2	2	2
	3	3	3
	4	4	4
	5	5	5
	6	6	6
	7	7	7
	8	8	8
	9	9	9

PLEASE DO NOT WRITE IN THIS AREA

Health Care Utilization

Participant ID ______________________

Start Date ______________________

End Date ______________________

Submitted ______________________

COLDS AND/OR FLU

Have you had cold or flu symptoms in the last week such as a runny or stuffy nose, sneezing, coughing, sore throat or body aches?

1 ☐ Yes 0 ☐ No

**CALL THE STUDY TEAM TO REPORT YOUR COLD!! 608-263-2653

VISITS TO MEDICAL PROVIDER

Since we last asked you, have you been seen by a doctor, nurse practitioner, or physician assistant?

1 ☐ Yes 0 ☐ No

What was the DATE of your first visit? ______________________

Where were you seen for the first visit?

2 ☐ Primary care (Family Medicine, Internal Medicine, Pediatrics, Obstetrics/Gynecology)
3 ☐ Specialty clinic (i.e. Ears Nose Throat, Gastroenterology, Oncology, Surgery, etc)
4 ☐ Urgent Care
5 ☐ Hospital/ER
6 ☐ Other

Other (where you were seen for the first visit) ______________________

What was the REASON for the first visit? ______________________

If you had a second visit, what was the date of your second visit? ______________________

Where were you seen for the second visit?

2 ☐ Primary care (Family Medicine, Internal Medicine, Pediatrics, Obstetrics/Gynecology)
3 ☐ Specialty clinic (i.e. Ears Nose Throat, Gastroenterology, Oncology, Surgery, etc)
4 ☐ Urgent Care
5 ☐ Hospital/ER
6 ☐ Other

Other (where you were seen for the second visit) ______________________

What was the REASON for the second visit? ______________________

If you had a third visit, what was the date of your third visit? ______________________

Where were you seen for the third visit?

2 ☐ Primary care (Family Medicine, Internal Medicine, Pediatrics, Obstetrics/Gynecology)
3 ☐ Specialty clinic (i.e. Ears Nose Throat, Gastroenterology, Oncology, Surgery, etc)
4 ☐ Urgent Care
5 ☐ Hospital/ER
6 ☐ Other

Other (where you were seen for the third visit) ____________________

What was the REASON for the third visit? ____________________

If you had a fourth visit, what was the date of your fourth visit? ____________________

Where were you seen for the fourth visit?

2 ☐ Primary care (Family Medicine, Internal Medicine, Pediatrics, Obstetrics/Gynecology)
3 ☐ Specialty clinic (i.e. Ears Nose Throat, Gastroenterology, Oncology, Surgery, etc)
4 ☐ Urgent Care
5 ☐ Hospital/ER
6 ☐ Other

Other (where you were seen for the fourth visit) ____________________

What was the REASON for the fourth visit? ____________________

If you had a fifth visit, what was the date of your fifth visit? ____________________

Where were you seen for the fifth visit?

2 ☐ Primary care (Family Medicine, Internal Medicine, Pediatrics, Obstetrics/Gynecology)
3 ☐ Specialty clinic (i.e. Ears Nose Throat, Gastroenterology, Oncology, Surgery, etc)
4 ☐ Urgent Care
5 ☐ Hospital/ER
6 ☐ Other

Other (where you were seen for the fifth visit) ____________________

What was the REASON for the fifth visit? ____________________

HOSPITALIZATIONS

Since we last asked you, have you stayed overnight in the hospital for any reason?

1 ☐ Yes 0 ☐ No

What was the reason(s) for your hospital stay? ____________________

Date admitted to the hospital ____________________

How many nights did you spend in the hospital? ____________________

DAYS MISSED OF WORK OR SCHOOL

Since we last asked you, has your work situation changed?

1 ☐ Yes 0 ☐ No

Please explain how your employment situation has changed ____________________

What is your CURRENT employment status? (check all that apply) Please note we want to include all hours you work per week and any work you may have done for pay or profit. This can include activities without pay you have done for 15 hours or more per week in a family-owned business operated by someone in your household.

1 ☐ Full time (35 + hours/ week)
2 ☐ Part-time (1-34 hours/ week)
3 ☐ Not employed
4 ☐ Student
5 ☐ Retired

Since we last asked you, have you missed any days of work or school? (if unemployed or retired check NO)

1 ☐ Yes 0 ☐ No

What was the REASON for missed work or school? ______________________________

What's the first date of work or school that you missed? ______________________________

How many hours of work or school did you miss on that first date? ______________________________

What's the second date of work or school that you missed? ______________________________

How many hours of work or school did you miss on that second date? ______________________________

What's the third date of work or school that you missed? ______________________________

How many hours of work or school did you miss on that third date? ______________________________

What's the fourth date of work or school that you missed? ______________________________

How many hours of work or school did you miss on that fourth date? ______________________________

What's the fifth date of work or school that you missed? ______________________________

How many hours of work or school did you miss on that fifth date? ______________________________

What's the sixth date of work or school that you missed? ______________________________

How many hours of work or school did you miss on that sixth date? ______________________________

What's the seventh date of work or school that you missed? ______________________________

How many hours of work or school did you miss on that seventh date? ______________________________

TO BE COMPLETED BY BLINDED STUDY PERSONNEL ONLY

ARI Verified By

1 ☐ Jackson
2 ☐ Nasal Wash
3 ☐ RIDL

Was first visit to medical provider related to ARI?

1 ☐ Yes 0 ☐ No

Was second visit to medical provider related to ARI?

1 ☐ Yes 0 ☐ No

Was third visit to medical provider related to ARI?	1 ☐ Yes	0 ☐ No
Was fourth visit to medical provider related to ARI?	1 ☐ Yes	0 ☐ No
Was fifth visit to medical provider related to ARI?	1 ☐ Yes	0 ☐ No
Was the hospitalization related to ARI?	1 ☐ Yes	0 ☐ No
Was first day of missed work related to ARI?	1 ☐ Yes	0 ☐ No
Was second day of missed work related to ARI?	1 ☐ Yes	0 ☐ No
Was third day of missed work related to ARI?	1 ☐ Yes	0 ☐ No
Was fourth day of missed work related to ARI?	1 ☐ Yes	0 ☐ No
Was fifth day of missed work related to ARI?	1 ☐ Yes	0 ☐ No
Was sixth day of missed work related to ARI?	1 ☐ Yes	0 ☐ No
Was seventh day of missed work related to ARI?	1 ☐ Yes	0 ☐ No
ARI Verified Notes	______________________	

Health Care Utilization

Participant ID ________________________

Start Date ________________________

End Date ________________________

Submitted ________________________

COLDS AND/OR FLU

Have you had cold or flu symptoms in the last week such as a runny or stuffy nose, sneezing, coughing, sore throat or body aches?
☐ Yes ☐ No

**CALL THE STUDY TEAM TO REPORT YOUR COLD!! 608-263-2653

VISITS TO MEDICAL PROVIDER

Since we last asked you, have you been seen by a doctor, nurse practitioner, or physician assistant?
☐ Yes ☐ No

What was the DATE of your first visit? ________________________

Where were you seen for the first visit?
- ☐ Primary care (Family Medicine, Internal Medicine, Pediatrics, Obstetrics/Gynecology)
- ☐ Specialty clinic (i.e. Ears Nose Throat, Gastroenterology, Oncology, Surgery, etc)
- ☐ Urgent Care
- ☐ Hospital/ER
- ☐ Other

Other (where you were seen for the first visit) ________________________

What was the REASON for the first visit? ________________________

If you had a second visit, what was the date of your second visit? ________________________

Where were you seen for the second visit?
- ☐ Primary care (Family Medicine, Internal Medicine, Pediatrics, Obstetrics/Gynecology)
- ☐ Specialty clinic (i.e. Ears Nose Throat, Gastroenterology, Oncology, Surgery, etc)
- ☐ Urgent Care
- ☐ Hospital/ER
- ☐ Other

Other (where you were seen for the second visit) ________________________

What was the REASON for the second visit? ________________________

If you had a third visit, what was the date of your third visit? ________________________

Where were you seen for the third visit?

- ☐ Primary care (Family Medicine, Internal Medicine, Pediatrics, Obstetrics/Gynecology)
- ☐ Specialty clinic (i.e. Ears Nose Throat, Gastroenterology, Oncology, Surgery, etc)
- ☐ Urgent Care
- ☐ Hospital/ER
- ☐ Other

Other (where you were seen for the third visit) ______________________________

What was the REASON for the third visit? ______________________________

If you had a fourth visit, what was the date of your fourth visit? ______________________________

Where were you seen for the fourth visit?

- ☐ Primary care (Family Medicine, Internal Medicine, Pediatrics, Obstetrics/Gynecology)
- ☐ Specialty clinic (i.e. Ears Nose Throat, Gastroenterology, Oncology, Surgery, etc)
- ☐ Urgent Care
- ☐ Hospital/ER
- ☐ Other

Other (where you were seen for the fourth visit) ______________________________

What was the REASON for the fourth visit? ______________________________

If you had a fifth visit, what was the date of your fifth visit? ______________________________

Where were you seen for the fifth visit?

- ☐ Primary care (Family Medicine, Internal Medicine, Pediatrics, Obstetrics/Gynecology)
- ☐ Specialty clinic (i.e. Ears Nose Throat, Gastroenterology, Oncology, Surgery, etc)
- ☐ Urgent Care
- ☐ Hospital/ER
- ☐ Other

Other (where you were seen for the fifth visit) ______________________________

What was the REASON for the fifth visit? ______________________________

HOSPITALIZATIONS

Since we last asked you, have you stayed overnight in the hospital for any reason? ☐ Yes ☐ No

What was the reason(s) for your hospital stay? ______________________________

Date admitted to the hospital ______________________________

How many nights did you spend in the hospital? ______________________________

DAYS MISSED OF WORK OR SCHOOL

Since we last asked you, has your work situation changed? ☐ Yes ☐ No

Please explain how your employment situation has changed ______________________________

What is your CURRENT employment status? (check all that apply) Please note we want to include all hours you work per week and any work you may have done for pay or profit. This can include activities without pay you have done for 15 hours or more per week in a family-owned business operated by someone in your household.

☐ Full time (35 + hours/ week)
☐ Part-time (1-34 hours/ week)
☐ Not employed
☐ Student
☐ Retired

Since we last asked you, have you missed any days of work or school? (if unemployed or retired check NO)

☐ Yes ☐ No

What was the REASON for missed work or school? ________________________________

What's the first date of work or school that you missed? ________________________________

How many hours of work or school did you miss on that first date? ________________________________

What's the second date of work or school that you missed? ________________________________

How many hours of work or school did you miss on that second date? ________________________________

What's the third date of work or school that you missed? ________________________________

How many hours of work or school did you miss on that third date? ________________________________

What's the fourth date of work or school that you missed? ________________________________

How many hours of work or school did you miss on that fourth date? ________________________________

What's the fifth date of work or school that you missed? ________________________________

How many hours of work or school did you miss on that fifth date? ________________________________

What's the sixth date of work or school that you missed? ________________________________

How many hours of work or school did you miss on that sixth date? ________________________________

What's the seventh date of work or school that you missed? ________________________________

How many hours of work or school did you miss on that seventh date? ________________________________

TO BE COMPLETED BY BLINDED STUDY PERSONNEL ONLY

ARI Verified By

☐ Jackson
☐ Nasal Wash
☐ RIDL

Was first visit to medical provider related to ARI?

☐ Yes ☐ No

Was second visit to medical provider related to ARI?

☐ Yes ☐ No

Was third visit to medical provider related to ARI?	☐ Yes	☐ No
Was fourth visit to medical provider related to ARI?	☐ Yes	☐ No
Was fifth visit to medical provider related to ARI?	☐ Yes	☐ No
Was the hospitalization related to ARI?	☐ Yes	☐ No
Was first day of missed work related to ARI?	☐ Yes	☐ No
Was second day of missed work related to ARI?	☐ Yes	☐ No
Was third day of missed work related to ARI?	☐ Yes	☐ No
Was fourth day of missed work related to ARI?	☐ Yes	☐ No
Was fifth day of missed work related to ARI?	☐ Yes	☐ No
Was sixth day of missed work related to ARI?	☐ Yes	☐ No
Was seventh day of missed work related to ARI?	☐ Yes	☐ No
ARI Verified Notes	______________________________	

ATTENDANCE AT INTERVENTION SESSIONS

Attendance is collected at all intervention sessions. The instructors send the attendance records to the MEPARI staff, where the data is manually entered into REDCap and verified.

7.2.7 Subgroup analyses ...Pre-planned secondary efficacy analyses will be conducted on the following sub-groups: 1) those who attend at least 7 of the 8 weekly training sessions ... One-sided testing will be based on the underlying hypotheses that: A) behavioral trainings are more likely to work for those who attend classes.

Appendix I: ... ARI episodes, missed work, etc [data] Per protocol (PP): Only people who attend at least 5 of the 9 exercise or mindfulness sessions will be included. For cohort 1 (2012-13) the following date will serve as cut-off for PP analysis: Oct. 22.

BIG FIVE INVENTORY

Computing Simple BFI Scale Scores
1=Disagree Strongly; 2=Disagree A Little; 3=Neither Agree or Disagree; 4=Agree a Little; 5=Agree Strongly

Reverse score the items labeled "R" and compute scale scores as the mean of the following items:

Extraversion (8 items): 1, 6R, 11, 16, 21R, 26, 31R, 36
Agreeableness (9 items): 2R, 7, 12R, 17, 22, 27R, 32, 37R, 42
Conscientiousness (9 items): 3, 8R, 13, 18R, 23R, 28, 33, 38, 43R
Neurotiscism (8 items): 4, 9R, 14, 19, 24R, 29, 34R, 39
Openness (10 items): 5, 10, 15, 20, 25, 30, 35R, 40, 41R, 44

Computing the content-balanced acquiesence index and Ipsatizing the BFI items
(Ipsatizing looks at the degree to which a participant answers pre-paired "opposite" characteristic questions in opposite directions.)
SPSS sytnax: compute within person response means [of raw score] and standard deviations for 3,8,9,13,18,19,23,24,28,29,34,39 as a Z-score.
Conscientiousness: 3 and 43, 8 and 13; 18 and 33; 23 and 28

Neurotiscism: 9 and 19; 24 and 29; 34 and 39

Instrument is administered at Run-in and Exit

From the MEPARI Protocol Manual 07-18-12 (p. 13, 16)
6.4.5 Big Five Inventory (BFI) Research on personality and health has been underway for some time, leading to various conceptual structures of state and trait psychological domains. The Big Five taxonomy has helped clarify and organize the links between personality, health behaviors, illness and mortality across the lifespan. Of the five dimensions measured (openness, conscientiousness, extraversion, agreeableness, and neuroticism), we will use baseline “conscientiousness” and “neuroticism” scores on the Big Five Inventory to gauge propensity for self-report bias on instrument completion activities, and to control for between person differences in multivariate efficacy models.

Covariate to control for between-person variability

John OP, Naumann LP, Soto CJ. Paradigm shift to the integrative big-five trait taxonomy: History, measurement and conceptual issues. In John OP, Robins RW, Pervin LA, eds. *Handbook of Personality: Theory and Research* , pp 114-58. New York: Guilford Press, 2008.

BIOMARKERS

Interleukin 6 secondary outcome, potential mediator
Interleukin 8 secondary outcome, potential mediator
IFN-induced Protein 10 secondary outcome, potential mediator
C-reactive Protein secondary outcome, potential mediator
Procalcitonin secondary outcome, potential mediator
Neutrophil potential mediator
HgA1C secondary outcome
Viral Identification secondary outcome, potential mediator

Outcomes: Blood and nasal wash samples will be obtained at baseline, one month after the end of the 8-week interventions, and once again three months later. Blood and nasal wash samples will be obtained approximately 24-72 hours into each ARI episode. Nasal wash samples will be tested with multiplex PCR (polymerase chain reaction) to identify etiological agents. Serum and nasal wash will be analyzed for interleukin-6, interleukin-8, C-reactive protein, procalcitonin, and interferon-gamma-induced protein 10. These inflammatory biomarkers will serve as objective indicators of disease severity to compare with illness severity self-reported on the WURSS-24 ... Inflammatory biomarkers ... will be analyzed as potential mediators of causal pathways leading from behavioral training interventions to ARI illness outcomes.

6.3.4 Viral identification will be done in Dr. Gern's lab, where high-throughput PCR-based multiplex methods have been developed and authenticated, and are able to identify nearly all of the pathogens associated with ARI illness [36;261-264] Trial will assess 2 samples, one done by self-swab at home, and the other by nasal wash at lab. We will also improve sample processing and include newly developed viral types. Dr. Gern's published data report that up to 91.4% of nasal washes from community-acquired ARI can yield positive viral IDs.[265]

6.3.5 Pro-inflammatory cytokines Laboratory-assessed objective measures will primarily serve to corroborate self-reports of disease severity. C-reactive protein (CRP) and procalcitonin (PCT) are well-established indicators of disease severity during respiratory infection, and can be measured in serum as well as in nasal wash.[113;115;118-120] Concentrations of interleukin-6 (IL-6)[266-271] and interleukin-8 (IL-8)[272-276] in nasal wash have been shown to correlate with illness severity. More recently, interferon-gamma-induced protein 10 (IP-10) has been shown to be measurably increased in both serum and nasal wash during times of acute viral ARI.[122-128] Inflammatory cytokines will be measured by ELISA methods in laboratories directed by Dr. Coe and Dr. Hayney.

6.3.6 Inflammatory tendency The same array of pro-inflammatory cytokines will also be analyzed as indicators of low level inflammation or pro-inflammatory tendency and as potential mediators of effects of behavioral interventions on ARI illness incidence, duration, and severity. The importance of CRP, PCT and IL-6 has been underscored by the ability of these pro-inflammatory biomarkers to predict mortality.[277-285] As potential mediators, pro-inflammatory cytokines (CRP, PCT, IL-6, IL-8, IP-10) will be assessed as change from baseline to one month after the 8 week behavioral interventions finish. Repeating these assays 3 months later will assess whether potential pro-inflammatory changes resulting from interventions will be sustained.

6.3.7 Polymorphonuclear neutrophil count in nasal mucus is a relatively well-established indicator of inflammation of the nasal epithelium.[286-290] Neutrophil counts correlate to symptom severity, viral titer and cytokine levels.[174;291] ... Neutrophil counts will be done on nasal wash collected during ARI episodes.

6.3.8 Glycosylated hemoglobin (HgA1C) Regular exercise is known to reduce hemoglobin A1C, a widely accepted indicator of average blood glucose levels.[142;292-294] There are at least two preliminary reports suggesting that mindfulness meditation might reduce HgA1C.[295;296] To explore these possibilities, we will assess HgA1C at baseline, 1 month after interventions, and again 3 months later.

For IL-6, a value of 0.01 indicates a level below detectable concentration, and for IP-10, a value of 1 indicates a level below detectable concentration.

BLOOD PRESSURE

Blood pressure is taken at Baseline, December, March and Exit visits.

American Heart Association …

Category	systolic, mmHg	diastolic, mmHg
Hypotension	<90	<60
Desired	90-119	60-79
Prehypertension	120-139	80-89
Stage 1 Hypertension	140-159	90-99
Stage 2 Hypertension	160-179	100-109
Hypertensive Crisis	≥ 180	> 110

From the MEPARI Protocol Manual 07-18-12 (p. 15, p. 20)

6.4.24 Blood pressure Blood pressure is a well-recognized health indicator. There is some reason to believe that stress reduction or regular exercise might reduce blood pressure. In this study, blood pressure will be assessed at baseline and at both standardized follow-up periods using standard calibrated sphygmomanometers. Blood pressure will be analyzed as a secondary outcome using methods described in Section 7.

7.2.6 Secondary efficacy analyses Influence of interventions on secondary outcomes will be assessed using ANOVA-based multivariate regression models using SAS software.381-383 Adjustment for multiple comparisons will be incorporated, and interpretation will be cautious.238;384-388 In general, we will want to see relationships with p<0.01 in order to justify tentative null hypothesis rejection. Pre-planned secondary efficacy analyses will include effects of interventions on: ... 9) blood pressure ... 7.2.6.9 Those in the intervention groups will have lower blood pressure compared to control.

Secondary outcome

BODY MASS INDEX

Height (in decimalized inches) is captured at baseline visit; weight (in decimalized pounds) is captured at Baseline, December, March and Exit visits.

Data is entered from CRU flowsheets into corresponding REDCap "event".

Excel formula for calculating BMI: 703*weight/height^2

From the MEPARI Protocol Manual 07-18-12 (p. 12)

6.4.2 Body Mass Index (BMI) Body habitus is associated with many disease processes, and may be related to immune function and susceptibility to respiratory infection. Height will be assessed at baseline only. Weight will be measured at baseline, 1 and 4 months post-intervention, and at exit. Baseline BMI will be calculated and used as a covariate in statistical models. BMI will also be considered a secondary outcome of potential importance.

Secondary outcome, potential mediator

DEMOGRAPHICS

1 1 - Date of birth: Mo/Da/Year
2 Gender: Male = 1; Female = 2
3 Ethnicity (Hispanic/Latino origin): No = 0; Yes = 1
4 Race: 1= Black/African American; 2= Native Hawaiian or other Pacific Islander; 3= White/Caucasian; 4 = American Indian or Alaska Native; 5 = Asian; 6 = Other
5 Smoking history: 0 = Never Smoked; 1 = Past Smoker; 2 = Current smoker; 3 = 5 or fewer cigs; 4 = More than 5 cigs
6 Education: 1=Some high school; 2=High school grad/GED; 3=Some college/tech school; 4=College grad (bachelor's); 5=College post grad (master's, doctoral)
7 Household income: 1 = <$15K; 2 = $15-25K; 3 = $25-50K; 4 = $50-75K; 5 = $75-100K; 6 = Over $100K
8 Not applicable OR Salary (up to 4 jobs - 8a, 8b, 8c, 8d): Wages per hour/week/month/year; I work __ hours per week.*

Scanned document collected at run-in and an abbreviated version (Education/Household Income/Salary) at exit.

From the MEPARI Protocol Manual 07-18-12 (p. 13)
6.4.3 Demographic indicators Socioeconomic status is related to health and disease, including incidence and severity of respiratory infection. Demographic indicators to be assessed will include age, sex, years of education completed, household income, and number of children under the age of 18 living in the home. Age, sex and education will be used as covariates in multivariate efficacy analyses.

*Salary information will be averaged to garner an average hourly salary across all jobs and will be used in conjuction with missed days of work reported on weekly HCU forms.

Number of children under age 18 living in the home is garnered from the SNI (see separate instrument instructions).

Covariate to control for between-person variability

EXERCISE SELF-EFFICACY

This is an 18-item scale scored on a scale of 0-10, with participants indicating their confidence in their ability to perform exercise on a regular basis. A score of 0 indicates their level of confidence as "cannot do at all", while a score of 10 indicates their level of confidence is "certain can do". Items are summed for each participant. Range of scores is 0-180.

Higher scores indicate higher levels of self-efficacy.

Instrument is administered at Run-in Homework, December, February and April

When cleaning data, scanned "A" entries should be converted to "10".

From the MEPARI Protocol Manual 07-18-12 (p. 15)

6.4.18 Exercise Self Efficacy (ESES) Self-efficacy has been defined as "the belief in one's capabilities to organize and execute the courses of action required to manage prospective situations." The ESES scale was developed based on work by Bandura and colleagues, and has been validated by Shin, Kroll, and Everett. For our study, the ESES will be used to verify results of the exercise intervention, and to help explain potential mediational effects of exercise.

Secondary outcome, potential mediator

Everett B, Salamonson Y, Davidson PM. Bandura's exercise self-efficacy scale: Validation in an Australian cardiac rehabilitation setting. *International Journal of Nursing Studies* 2009;**46**:824-9.

(Bandura advocated for using a scale of 0-100).

EXERCISE LOG

Minutes of daily practice data for 7 days - Monday through Sunday - entered into REDCap. Categories as denoted in REDCap headings are moderate = 1 or vigorous = 2.

Practice minutes will be summed within each of the 2 categories.

Unless participants randomized to the exercise arm of the study request a weekly phone call from study personnel for assistance in completing their weekly practice logs, they are sent weekly email reminders to complete the electronic survey.

From the MEPARI Protocol Manual 07-18-12 (p. 15)

6.4.22 Mindfulness practice and exercise daily tracking log After the 8-week intervention, participants assigned to the meditation group will be asked to continue meditation at ≥150 minutes/week, in sessions of at least 10 minutes each. Similarly, those assigned to the exercise group will be asked to continue moderate intensity exercise at ≥150 minutes/week, in sessions of at least 10 minutes each. Using modified versions of practice logs developed at the University of Wisconsin by Dr. Davidson (meditation) and at Appalachian State by Dr. Niemann (exercise), study participants will record their practice once daily on a paper log and will enter their practice minutes once weekly through an on-line web-based data collection portal.

7.2.7 Subgroup analyses ...Pre-planned secondary efficacy analyses will be conducted on the following sub-groups: ... 2) those who continue to exercise or meditate for an average of at least 60 minutes per week after the trainings end... One-sided testing will be based on the underlying hypotheses that: A) behavioral trainings are more likely to work for those who attend classes, and B) the interventions will be more effective for more highly stressed people.

Potential mediator

EXPECTANCY

Run-in "Thinking Ahead"
Question order is being alternated; even numbers have exercise expectancy questions asked first; odd numbers have meditation.
Q1 and Q3: No = 0; Yes = 1
Q2 and Q4: 1 = much worse; 2 = somewhat worse; 3 = slightly worse; 4 = the same; 5 = slightly better; 6 = somewhat better; 7 = much better; 8 = very much better

Baseline Post-randomization AND Follow-up 1 Post-intervention "Thinking Ahead"
1 = much worse; 2 = somewhat worse; 3 = slightly worse; 4 = the same; 5 = slightly better; 6 = somewhat better; 7 = much better; 8 = very much better

Exit "Thinking Back"
Q1: No = 0; Yes = 1
Q2: 1 = much worse; 2 = somewhat worse; 3 = slightly worse; 4 = the same; 5 = slightly better; 6 = somewhat better; 7 = much better; 8 = very much better

When running report from REDCap, using the "Raw" data gives the variables as numbered above.

From the MEPARI Protocol Manual 07-18-12 (p. 15)
6.4.23 Expectancy In order to assess and potentially control for intervention-related expectancy, we will ask participants about their attitudes towards meditation and exercise before and after randomization, after the 8-week behavioral trainings, and at exit.

GPAQ

Equation: Total Physical Activity = [(1a * 1b * 8) + (2a * 2b * 4) + (3a * 3b * 4) + (4a * 4b * 8) + (5a * 5b * 4)]

High:
IF:(1a + 4a) >= 3 days AND Total physical activity MET minutes per week is >= 1500
OR
IF: (1a + 2a + 3a + 4a + 5a) >= 7 days AND total physical activity MET minutes per week is >= 3000
Moderate:
IF: (1a + 4a) >= 3 days AND ((1a * 1b) + (4a * 4b)) >= 60 minutes
OR
• IF: (P5 + P8 + P14) >= 5 days AND ((P5 * P6) + (P8 * P9) + (P14 * P15)>= 150 minutes
OR
• IF: (P2 + P5 + P8 + P11 + P14)>= 5 days AND Total physical activity MET minutes per week >= 600
Low F: the value does not reach the criteria for either high or moderate levels of physical activity

Data cleaning notes:
Convert 4b to minutes (i.e. 1 hour 45 minutes = 105 minutes)
Q6 should NOT be time-formatted and must convert 30 minutes to 50, 45 minutes to 75, etc., for algorithm to work (i.e., 3 hours 30 minutes = 350; 2 hours 45 minutes = 275)

Instrument is administered at Baseline, November, January, March, Exit

From the MEPARI Protocol Manual 07-18-12 (p. 15)
6.4.21 Global Physical Activity Questionnaire (GPAQ) The GPAQ was developed and validated through the World Health Organization, and displays excellent reliability and responsiveness characteristics.[255;256] GPAQ scores will be used to assess degree-of-change resulting from exercise training, and for dose-dependency and mediation analyses.

Potential mediator

Bull FC, Maslin TS, Armstrong T. Global physical activity questionnaire (GPAQ): nine country reliability and validity study. *J.Phys.Act.Health* 2009;**6**:790-804.

HEALTHCARE UTILIZATION/WEEKLY CHECK-IN

Unless participants request a weekly phone call from study personnel for assistance in completing a WEEKLY CHECK-IN survey, they are sent weekly email reminders to complete the electronic survey. Once the weekly check-in survey is accessed, the first question answered is whether or not participants have had cold symptoms in the previous week (Monday-Sunday), and if they answer affirmatively, they are told to call the study phone number, if they have not already done so. The survey also asks questions about their visits to healthcare providers (including type of provider visit - i.e., primary care, specialty clinic, urgent care, emergency room, other), overnight hospitalizations, and the reason for the visit/hospitalization. UWHC rates tied to the visit type will be used to calculate healthcare usage costs.

Specific details of the anticipated usage of missed-work data is found in the scoring instructions for the DEMOGRAPHIC questionnaire, but the gist is that it will be tied to the hourly wage the participant reports earning on the demographic questionnaire.

Determination of whether healthcare utilization or missed work are related to an ARI will be done by confirming via Jackson score. In instances where a participant reports a healthcare utilization and/or missed work, and has not called to report an ARI, but answers "yes" to the cold symptom question on the weekly check-in survey or offers an explanation of the reason for visiting a healthcare provider and/or missed work that is unclear on its potential connection to an ARI, blinded study personnel will contact the participant to ask for more information and make a

ARI-related medications will be captured on the final page of the RIDL booklets. The majority of this medication data will need to be hand-entered. Costs will be calculated using drugs.com or another similar online pricing guide.

From the MEPARI Protocol Manual 07-18-12 (p. 13)
6.4.6 Health care utilization and **antibiotics prescribed** Evaluation and treatment of ARI illness is very costly and often associated with unnecessary prescriptions, especially antibiotics. For this study, we plan to document total number of health care visits, ARI-related health care visits, and ARI-related prescriptions, including antibiotics. Each weekly communication will include the question, “Have you seen a doctor or visited a clinic, hospital or urgent care center?” Persons answering “Yes” will be asked the reason for the visit. Those answers will then be classified by study personnel as either “Related,” or “Unrelated” to ARI illness, including upper respiratory infection, influenza, pharyngitis, acute sinusitis, bronchitis, and pneumonia. All questionable cases will be verified by inspection of medical records (with case-specific participant permission). Prescriptions for antibiotics, prescription cough medicines, influenza antivirals and other ARI medications will be documented, as will self-reported use of nonprescription medications such as analgesics, antihistamines, decongestants, cough suppressants, and expectorants.

Secondary outcome

FEELING LOVED

The dichotomous A and B questions noted below are scored 0=no and 1=yes. The virtual analogue scale (VAS) values are entered as whole numbers ranging from 0-100.

Instrument is administered at Run-in Homework, December, February and April

From the MEPARI Protocol Manual 07-18-12 (p. 15)
6.4.17 Feeling Loved (FL) In addition to the validated perceived social support measures described above, we will use two novel questions with Yes/No response options: A) Do you feel loved? B) Do you love yourself? and two questions with visual analogue (VAS) response scales: How loved do you feel? How much do you love yourself? The ends of each 100mm VAS scale will be bounded by "not at all" and "very, very much."

Potential mediator

JACKSON

Sum the score of the symptoms. Range possibility of 0-24 (symptoms would only be assessed for severity if they are

Date of completion of Jackson screening may be utilized to determine start date of ARI episode in the event that RIDL data is incomplete.

When affirmative response is made to Question 1 on Weekly Check-in indicating presence of cold symptoms in the past week, confirming of a Jackson score is first step to ensure ARI data is being captured.

Jackson score is used to validate ARI-relatedness of healthcare utilization and missed work.

Administered when participant calls to report suspected ARI.

From the MEPARI Protocol Manual 07-18-12 (p. 12)
6.3.1 Definition of ARI illness The beginning of each ARI illness episode will be defined by: 1) answering "Yes" to either: "Do you think you have a cold" or "Do you think you are coming down with a cold?" AND 2) reporting at least 1 of 4 cold symptoms or synonyms: nasal discharge (runny nose); nasal obstruction (plugged or congested); sneezing; or sore (scratchy) throat, AND 3) scoring at least 2 points on the Jackson scale. The Jackson score is calculated by summing 8 symptom scores (sneezing, headache, malaise, chilliness, nasal discharge, nasal obstruction, sore throat and cough) rated , 0=absent, 1=mild, 2=moderate, and 3=severe.[170-172] In order for these symptoms to be classified as an ARI illness episode (and analyzed as such), at least 2 days in a row must meet these criteria. From the first day of ARI illness and forward each participant will fill out a daily WURSS-24 until they answer "No" to the question "Do you think that you are still sick with this respiratory infection?" for 2 days in a row. The last day the participant answers "Yes" will be the last day classified as ARI illness and included in the calculation of severity-weighted days of ARI illness.

Integral to determining ARI presence; however, not a primary outcome.

Jackson GG, Dowling HF, Spiesman IG, Boand AV. Transmission of the common cold to volunteers under controlled conditions. *Arch Intern Med* 1958;**101**:267-78.

MAAS

Descriptor taken from Brown 2003, p. 825
Respondents indicate how frequently they have the experience described in each statement using a 6-point Likert scale from 1 (almost always) to 6 (almost never), where high scores reflect more mindfulness.

Descriptor taken from L.E. Carlson and K.W. Brown, Validation of the Mindful Attention Awareness Scale in a cancer population, J Psychosom Res 58 (2005), pp. 29–33.
This 15-item scale measures the frequency of mindful states in day-to-day life, using both general and situation-specific statements. Individual scores are summed and then divided by 15 to determine the mean MAAS score, which can range from 1 to 6. Higher scores indicate greater mindfulness.

MEPARI 2 scoring follows Carlson instructions; mean is decimalized to the tenth.

Instrument is administered at Baseline, November, January, March and Exit.

From the MEPARI Protocol Manual 07-18-12 (p. 15)
6.4.20 Mindfulness Attention Awareness Scale (MAAS) For our study, we will use the 15-item MAAS[248;249] to assess effects of MBSR training, and to help understand/explain potential mediating influences of mindfulness on our major outcomes. The MSES and MAAS instruments will also serve as an intervention check, in that scores are expected to change more among those randomized to meditation than in the exercise or control groups.

Potential mediator

Carlson LE,.Brown KW. Validation of the Mindful Attention Awareness Scale in a cancer population. *J.Psychosom.Res.* 2005;**58**:29-33.

Brown KW,.Ryan RM. The benefits of being present: mindfulness and its role in psychological well-being. *J.Pers.Soc.Psychol.* 2003;84:822-48.

MEDITATION LOG

Minutes of daily practice data for 7 days - Monday through Sunday - entered into REDCap. Categories as denoted in REDCap headings are formal = 1 or informal = 2.

Practice minutes will be summed within each of the 2 categories.

Unless participants randomized to the Meditation arm of the study request a weekly phone call from study personnel for assistance in completing their weekly practice logs, they are sent weekly email reminders to complete the electronic

From the MEPARI Protocol Manual 07-18-12 (p. 15)
6.4.22 Mindfulness practice and exercise daily tracking log After the 8-week intervention, participants assigned to the meditation group will be asked to continue meditation at ≥150 minutes/week, in sessions of at least 10 minutes each. Similarly, those assigned to the exercise group will be asked to continue moderate intensity exercise at ≥150 minutes/week, in sessions of at least 10 minutes each. Using modified versions of practice logs developed at the University of Wisconsin by Dr. Davidson (meditation) and at Appalachian State by Dr. Niemann (exercise), study participants will record their practice once daily on a paper log and will enter their practice minutes once weekly

7.2.7 Subgroup analyses ...Pre-planned secondary efficacy analyses will be conducted on the following sub-groups: ... 2) those who continue to exercise or meditate for an average of at least 60 minutes per week after the trainings end... One-sided testing will be based on the underlying hypotheses that: A) behavioral trainings are more likely to work for those who attend classes, and B) the interventions will be more effective for more highly stressed people.

Potential mediator

MINDFULNESS SELF-EFFICACY SCALE

Responses are ranked as 0=Not at all; 1=A little; 2=Moderately; 3=A lot; 4=Completely

The MSES comprises seven generic subscales of self-efficacy:
1. Behaviour (items 1, 8, 15, 22, 29)
2. Cognition (items 2, 9, 16, 23, 30)
3. Interoception (items 3, 10, 17, 24, 31)
4. Affect (items 4, 11, 18, 25, 32)
5. Interpersonal (items 5, 12, 19, 26, 33)
6. Avoidance (items 6, 13, 20, 27, 34)
7. Mindfulness (items 7, 14, 21, 28, 35)

Before scale and global scores of self-efficacy can be calculated, 18 items must be scored in reverse.
These are:
4 5 6 8 11 14 16 17 22 23 25 26 27 28 29 30 34 35
LOOKUP(#REF!,{0,1,2,3,4},{4,3,2,1,0})
When the score for each of the 18 items listed above has been reversed, you can sum the scores for each of the 7 dimensions. The scale scores provide an estimate of Dimensional Self Efficacy (DSE) for each dimension. To calculate the Global Self Efficacy (GSE) score, sum all DSE scores.

The current lack of psychometric data for the MSES renders the following ranges very tentative. They are currently only a rough clinical guide and scores must be interpreted with caution.
0-34 Poor sense of self-efficacy
35-69 Weak sense of self-efficacy
70-104 Moderate sense of self-efficacy
105-140 Good sense of self-efficacy

The MSES was constructed to measure the change in levels of self-efficacy before, during, and following mindfulness-based therapy programs. Participants in these programs will relate more easily to some of the items presented, especially items 3 and 10 in Interoception subscale. This is because body scan tasks of such programs tend to increase interoceptive awareness and acceptance, whereas a number of non-experiential therapies do not. As a result, a person undergoing counseling or traditional cognitive therapy is likely to interpret a high score on item 3 as undesirable. The context in which this scale is being used is therefore a factor worth taking into account.

Instrument is administered at Run-in Homework, December, February and April

From the MEPARI Protocol Manual 07-18-12 (p. 15)
6.4.19 Mindfulness-based Self-Efficacy Scale (MSES) Research aimed at defining and assessing the concept of "mindfulness" is well underway, with several questionnaire instruments available.[367-374] The MSES is one of the more recent questionnaires, developed by Cayoun and Freestun to assess effects of MBSR training on perceived self-efficacy.[247] The MSES assesses 7 domains related to mindfulness self-efficacy, including behavior, cognition, interoception, affect, interpersonal, avoidance and mindfulness. The MSES will provide a nice counterpart to the ESES

Secondary outcome, potential mediator

http://www.mytherapysession.com/PDFs/MSESSelfEfficacyScale.pdf
This citation link for the 36-item MSES is broken. Current instrument availability is of the MSES-R, a revised 22-item instrument.

POSITIVE AFFECT/NEGATIVE AFFECT SCALE

The Positive Affect Negative Affect Schedule (PANAS) is a psychometric scale developed to measure the largely independent constructs of positive and negative affect, both as states and traits. Participants respond to 20 words by ascribing the degree to which they have felt them in the past few weeks. The options are 1 - very slightly or not at all; 2 - a little; 3 - moderately; 4 - quite a bit; 5 - extremely. Score for each affect direction (positive or negative) range between 10 and 50, with higher scores indicating stronger affect tendency.

Items 1, 3, 5, 9, 10, 12, 14, 16, 17 and 19 with high scores are indicative of a positive affect.
Items 2, 4, 6, 7, 8, 11, 13, 15, 18 and 20 with high scores are indicative of a negative affect.

Sum the scores for each of the numbers corresponding to their respective affect.

Instrument is administered at Run-in Homework, December, February and April

From the MEPARI Protocol Manual 07-18-12 (p. 14)

6.4.13 Positive and Negative Affect Schedule (PANAS) The widely used PANAS scale reliably assesses both positive and negative affect (emotion).[338] Self-reported positive and negative emotion have long been known to be independent predictors of psychological and physical health.[339] In the ARI setting, positive and negative emotion predict not only symptom expression, but actual infection as indicated by viral shedding.[9;13;340] In an RCT setting, PANAS scores improved after MBSR training, ($p < 0.05$) as compared to controls.[30]

Potential mediator

Watson D, Clark LA, Tellegen A. Development and validation of brief measures of positive and negative affect: the PANAS scales. *Journal of Personality & Social Psychology* 1988;**54**:1063-70.

PHQ-9

Participants indicate which statement best describes how much they've been bothered by the given 9 problems over the previous two weeks using a 4-point scale. Choices are: 0 (not at all), 1 (several days), 2 (more than half the days) and 3 (nearly every day); thus, summed scores can range from 0-27 with higher numbers indicative of depression.

Question 2 assesses functional health. Changes in scores after the intervention begins can show impact on patient's

Make sure scanned scoring reflects 0-3, not 1-4

Instrument is administered at Run-in, December, February and April

From the MEPARI Protocol Manual 07-18-12 (p. 14)
6.4.10 Depression screen (PHQ-9) The PHQ-9 is a widely used and well-validated depression screen,[322-327] and also demonstrates good responsiveness.[328;329] In our study, prospective participants with PHQ-9 scores of ≥15 will be excluded (and referred to appropriate clinical care). PHQ-9 scores will be assessed as secondary outcomes.

Secondary outcome

Kroenke K, Spitzer RL, Williams JBW. The PHQ-9: Validity of a brief depression severity measure. *J Gen Intern Med* 2001;**16**:606-13.

PITTSBURG SLEEP QUALITY INDEX

Separate detailed scoring rubric exists.

Range of 0-21

The PSQI generates seven scores that correspond to these domains: Subjective Sleep Quality, Sleep Latency, Sleep Duration, Habitual Sleep Efficiency, Sleep Disturbances, Use of Sleep Medications, and Daytime Dysfunction. Each component score ranges from 0 (no difficulty) to 3 (severe difficulty). The component scores are summed to produce a global score (range of 0–21). A PSQI global score >5 is considered to be suggestive of significant sleep disturbance. Any missing data renders entire score null.

Be sure to check that values for scanned data for Questions 5a-5j range from 0-3, not 1-4 (need to convert otherwise.)

Responses to Questions 1 and 3 should be in military time (these values provide the time the participant spends in bed). Once the in-bed time has been calculated, it needs to be decimalized to be used in calculating a percentage of sleep efficiency. The sleep efficiency percentage algorithm uses a decimalized conversion of the response to Question 4. The % efficiency may be inverted positively (>100%) if estimates of self-reported hours of sleep per night are greater than what is derived from calculating the hours of sleep using the reported going to bed time and getting up time.

Instrument is administered at Baseline, November, January, March, Exit

From the MEPARI Protocol Manual 07-18-12 (p. 14)
Sleep quality may improve following interventions, and hence can be interpreted as a potentially important secondary outcome.

Secondary outcome, potential mediator

Buysse DJ, Reynolds CF, III, Monk TH, Berman SR, Kupfer DJ. The Pittsburgh Sleep Quality Index: a new instrument for psychiatric practice and research. *Psychiatry Research* 1989;**28**:193-213.

PSS-10

Gleaned from Cohen 1988
Participants indicate which statement best describes the frequency of their feelings and thoughts during the previous month using a 5-point scale. Choices are: 0 (never), 1 (almost never), 2 (sometimes), 3 (fairly often) and 4 (very often). Of the 10 items, 4 are worded in a positive direction and 6 are worded in a negative direction. After reversing the scoring for the positively worded items, item scores are summed to yield an overall perceived stress score with high scores representing greater perceived stress. Scores can range from 0-40.

Reverse scores for Items 4, 5, 7 and 8.
Excel algorithm for reversal: IF(*cell* =4,0,IF(*cell* =3,1,IF(*cell* =2,2,IF(*cell* =1,3,IF(*cell* =0,4)))))

Instructions for algorithm - replace the word "cell" with the actual cell correlates (A1, A2, A3, etc.)

Instrument is administered at Baseline, November, January, March, Exit

From the MEPARI Protocol Manual 07-18-12 (p. 14)
6.4.14 Perceived Stress Scale (PSS-10) The PSS-10 has been validated in multiple studies.[100-103;341-343] PSS scores predict rates of viral infection among volunteers inoculated with rhinovirus, and correlate with physiologic and self-report indicators of ARI illness, including nasal IL-6 level.[14;99-102] Because stress reduction is one of the hypothesized mechanisms of action, we have expanded our study population to include working-age participants, who we presume
7.2.7 Subgroup analyses ... Pre-planned secondary efficacy analyses will be conducted on the following sub-groups: ... 3) those who at baseline have perceived stress scores at least 14 points (PSS-10). One-sided testing will be based on the underlying hypotheses that: A) behavioral trainings are more likely to work for those who attend classes, and B) the interventions will be more effective for more highly stressed people.

Secondary outcome, potential mediator

http://www.ncsu.edu/assessment/resources/perceived_stress_scale.pdf
Norm Table from L. Harris Poll
Male Mean 12.1
Female Mean 13.7
Age
18-29 14.2
30-44 13.0
45-54 12.6
55-64 11.9
65 & older 12.0
Race
White 12.8
Hispanic 14.0
Black 14.7
Other minority 14.1

Cohen, S., & Williamson, G. (1988). Perceived stress in a probability sample of the United States. In S. Spacapam & S. Oskamp (Eds.), *The social psychology of health: Claremont Symposium on applied social psychology.* Newbury Park, CA:

SFS-12

Statistician generated scoring as part of instrument package purchase.

National mean norms for similarly aged adults using the SF-12…
Ages 30-34: Physical - 53.27; Mental - 48.90
Ages 35-44: Physical - 52.00; Mental - 48.79
Ages 45-54: Physical - 49.35; Mental - 49.90
Ages 55-64: Physical - 46.90; Mental - 50.84
Ages 65-69: Physical - 43.93; Mental - 51.57

Instrument is administered at Baseline, November, January, March, Exit

From the MEPARI Protocol Manual 07-18-12 (p. 14)
6.4.11 Health-related quality of life (SF-12) Also known as the Medical Outcomes Study Short Form, this 12-item questionnaire is commonly used to measure overall health, including physical (SF12-P) and mental health (SF12-M) subscales. It has been extensively assessed for reliability, responsiveness and criterion validity.[330-334] In our study, it will be used to assess potential changes in general physical and mental health due to interventions, and as a covariate to control for baseline between-person differences in multivariate efficacy analyses.

Secondary outcome, covariate to control for between-person variability.

Citation (separate book on Shari's shelf)
Ware, J.E. Jr., Kosinski, M., Turner-Bowker, D.M., Gandek, B. *User's Manual for the SF-12v2 Health Survey with a Supplement Documenting SF-12 Health Survey* Lincoln, RI: QualityMetric Incorporated, 2002.

SEATTLE INDEX OF COMORBIDITY

Respondents answer yes = 1 or no = 0 on nine health issues and self-report their smoking status.
SIC weighting formula = Age (in 5-year intervals) + Prior MI + 2*(Cancer) + Lung Disease + 2*(CHF) + 2*(Diabetes) + Pneumonia + 2*(Stroke) + 2*(Past Smoker) + 4*(Current smoker). 1 point was assigned for each 5-year interval above the age of 55.
age 30–54 = 0 points
55–59 = 1 point
60–64 = 2 points
65-69 = 3 points

Positive responses for individual items are also reported.

From Parimon T, Chien JW, Bryson CL, McDonell MB, Udris EM, Au DH. Inhaled corticosteroids and risk of lung cancer among patients with chronic obstructive pulmonary disease. Am J Respir Crit Care Med 2007;175, p. 713

The SIC is a weighted score derived from self-report of conditions and incorporates history of previous myocardial infarction, cancer, chronic lung disease, chronic heart failure, pneumonia, cerebral vascular accidents, and smoking status. Smoking status is obtained by self-report and categorized as never, past, and current smoker. MEPARI 2 instrument has 2 added items: allergies and asthma.

Instrument is administered at Run-in and Exit

From the MEPARI Protocol Manual 07-18-12 (p. 13)

6.4.4 Seattle Index of Comorbidity (SIC) People with diabetes, cardiovascular disease and pulmonary disease are known to have increased risks when infected with influenza or other respiratory viruses.[305-307] The SIC is a simple 8-item measure shown to predict hospitalization and mortality.[308] We will add items on allergy and asthma, as these illnesses are known to be related to severity of ARI. The modified SIC will be assessed at baseline and exit, and used as a covariate to control for possible influences of chronic disease on ARI outcomes. Relationships of individual items to outcomes and to other co-variates will be explored.

Covariate to control for between-person variability

Fan VS, Au D, Heagerty P, Deyo RA, McDonell MB, Fihn SD. Validation of case-mix measures derived from self-reports of diagnoses and health. *Journal of Clinical Epidemiology* 2002;**55**:371-80.

SOCIAL NETWORK INDEX

Role - there are 13 possible points (Roommate, Spouse, Parent, Child, Child-in-law, Close Relative, Close Friend, Religious Member, Group Member, Employee, Volunteer, Student, Neighbor)
High Contact - there are 14 possible points (Roommate, Spouse, Parent, Child, Child-in-law, Close Relative, Close Friend, Religious Member, Group Member, Employee Supervisor, Employee Peer, Volunteer, Student, Neighbor)
Potential Contact - there are 73 possible points (Roommate=1, Spouse=1, Parent=4, Child=2, Child-in-law=2, Close Relative=7, Close Friend=7, Religious Member=7, Group Member=7, Employee Supervisor=7, Employee Peer=7, Volunteer=7, Student=7, Neighbor=7)

Scoring details
Number of roles: for Question 1, if not "1", score 1 point; for Question 2, if "2", score 1 point; for Questions 3, 6, 7, 8, 9, 10, 11, 12, 13, one point is assigned for each type of relationship for which respondents answer other than "no" or "0"; for question 4, if not 2, score 1 point; for Question 5, if 1, recode to -99, if not 1, 3 or 4, score 1 point.
High contact role: for Question 2, if "2", score 1 point; for Questions 1, 3b, 6a, 7a, 8b, 9b, 10a, 10b, 11a, 12a, 13, one point is assigned if the participant answers anything but "no" or "none"; for Question 4a, if not "2", give 1 point; for Question 5a, if "1", recode to "-99", and if not "3", give 1 point.
9a, 10a, 10b, 11, 12a, 13, no recode; if Question 4 is 1=2, 2=0, 3 or 4=1; if Question 5 is 1, recode to -99, 2=2,3=0, 4 or 5=1

Reminder that in the MEPARI 2 data set, -99 indicates question was not applicable and -88 indicates missing data. This demands if using Excel to calculate the score, when tallying the total, to the sum, you must add the total of the -99 and -88 entries (i.e., if you had 3 answers with -99, you would add 297 to the total; if you had 3 answers of -99 and one with -88, you would add 385 to the total).

Items 3a and 3c are included as separate columns in dataset. Item 3a asks how many children live with participant >3 days/week on average. Answer values are None=0; 1=1; 2=2; 3 or more=3. Item 3c ascertains how many children ages 17 and under participant has contact in an average week. Answer values are: None=0; 1 to 5=1; 6 to 19=2; 20 or

Instrument is administered at Run-in Homework, December, February and April

From the MEPARI Protocol Manual 07-18-12 (p. 15)
6.4.16 Social Network Index (SNI) will serve to quantify social network size in order to help characterize social support. Cohen's research using the SNI suggests that the number of social contacts is predictive of susceptibility to ARI.[10;357] The SNI will also serve as an index of interpersonal contacts that could serve to transmit ARI virus. The SNI will be modified to document the number and ages of the children with whom participants have contact.

Demographic data regarding # of children under age 18 living more than half-time with participant is captured on SNI.

6.4.3 Demographic indicators Socioeconomic status is related to health and disease, including incidence and severity of respiratory infection.[102;303;304] Demographic indicators to be assessed will include age, sex, years of education completed, household income, and number of children under the age of 18 living in the home. Age, sex and education will be used as covariates in multivariate efficacy analyses

Potential mediator

Cohen S, Doyle WJ, Skoner DP, Rabin BS, Gwaltney JM. Social ties and susceptibility to the common cold. *JAMA* 1997;**277**:1940-4.

SOCIAL PROVISIONS SCALE

The instrument contains 24 items, four for each of the following: Attachment, Social Integration, Reassurance of Worth, Reliable Alliance, Guidance, and Opportunity for Nurturance. Half of the items describe the presence of a type of support and the others describe the absence of a type of support.

The respondent indicates on a 4-point scale the extent to which each statement describes her current social network. Responses range from 1 (strongly disagree) to 4 (strongly agree). After reversal of negatively worded items (indicated by an "R" below) a total score may be computed by summing all items. Subscale scores may be computed by summing items as follows:

• Attachment: Items 2R, 11, 17, and 21R
• Social Integration: Items 5, 8, 14R, and 22R
• Reassurance of Worth: 6R, 9R, 13, and 20
• Reliable Alliance: Items 1, 10R, 18R, and 23
• Guidance: Items 3R, 12, 16, and 19R
• Opportunity for Nurturance: 4, 7, 15R, and 24R

Scores Interpretation
A high score indicates a greater degree of perceived support.

http://www.iprc.unc.edu/longscan/pages/measures/Ages5to11/Social%20Provisions%20Scale.pdf

Instrument is administered at Baseline, November, January, March, Exit

From the MEPARI Protocol Manual 07-18-12 (p. 15)
6.4.15 Social Provisions Scale (SPS) [344-346] assesses perceived social support, which has been linked with a host of health and illness indicators.[347-352] The SPS is a 24-item index assessing 6 domains of social health: attachment, social integration, reassurance of worth, reliable alliance, guidance, and opportunity for nurturance. The SPS, developed by Russell and Cutrona,[344-346] predicts both immunological[353;354] and psychosocial outcomes.[355;356]

Potential mediator

Cutrona C, Russell D, Rose J. Social support and adaptation to stress by the elderly. *Psychology And Aging* . March 1986;1(1):47-54. Available from: PsycARTICLES, Ipswich, MA.

STANFORD PRESENTEEISM SCALE

Items receive a value of 1=strongly disagree; 2=somewhat disagree; 3=uncertain whether I disagree or agree; 4=somewhat agree; 5=strongly agree

Higher scores are indicative of a high level of presenteeism. Items 1, 3 and 4 are reverse coded.

http://www.drpelletier.com/chip/pdf/CHIP-stanford_presenteeism_scale.pdf

If participant does not answer Questions 1-6 and indicates not applicable, all answers should be filled in -99.
If participant answers Questions 1-6 AND answers not applicable below, disregard not applicable answer.

Instrument is administered at Run-in Homework, December, February and April.
A modified version is administered during an ARI.

From the MEPARI Protocol Manual 07-18-12 (p. 14)
6.4.9 Absenteeism/Presenteeism At enrollment we will assess employment, including type of work, hours per week worked, and compensation, assessed as hourly wage. Each week we will ask about any missed work, ascertain number of hours missed, and assess and classify reasons for missing work as either ARI-related or not ARI-related. The person making the classification will be blinded to allocation. Beyond missed work (absenteeism), illnesses such as ARI can decrease energy and focus at work,[260] leading to lost work productivity[318] from reduced "presenteeism."[319] To refine economic impact analysis, we will assess self-reported ability to perform work using the Stanford Presenteeism Scale (StPS).[320;321] The standard 1-month recall version will be administered at baseline, then 1, 3 and 5 months after interventions. A modified version with illness-specific recall will be used at end of each ARI episode, at the end of the RIDL questionnaire booklet (see 6.3.3 above).

Secondary outcome

Turpin RS, Ozminkowski RJ, Sharda CE, Collins JJ, Berger ML, Billotti GM *et al* . Reliability and validity of the Stanford Presenteeism Scale. *J.Occup.Environ.Med* 2004;**46**:1123-33.

TIMELINE FOLLOWBACK

The TLFB is a continuous measure of alcohol consumption based on recall of the prior two weeks, with 1 drink = 12 oz of beer, 5 oz of wine, and 1.5 oz of spirits. On the basis of the frequency and intensity of drinking, the TLFB measures the following: (1) days of heavy drinking in past 2 weeks, (2) number of drinking days in past 2 weeks, and (3) number of drinks per week. For MEPARI, tobacco usage is also calculated.

Instrument is administered at Baseline, November, January, March, Exit

From the MEPARI Protocol Manual 07-18-12 (p. 13)

6.4.1 Alcohol and tobacco use Tobacco use is associated with depressed immune function and increased rate and severity of acute respiratory infection (ARI).[297] Overuse of alcohol also appears to be associated with immune system depression and increased levels of ARI illness.[297] To assess and monitor both tobacco and alcohol use, we will use the validated and widely used Timeline Followback method (TLFB).[298-302] We will consider tobacco use and alcohol overuse as secondary outcomes of potential importance. Baseline tobacco use will be used as a covariate in multivariate

TLFB Alcohol - Secondary outcome
TLFB Smoking - Secondary outcome, covariate to control for between-person differences

Sobell LC, Agrawal S, Sobell MB, Leo GI, Young LJ, Cunningham JA *et al* . Comparison of a quick drinking screen with the timeline followback for individuals with alcohol problems. *Journal of Studies on Alcohol* 2003;**64**:858-61.

WURSS

Duration Formulas embedded in these cells in this document

Start Date and time	End Date and time	Calculate Duration	Convert to Hrs	Decimalized Days
4/2/2010 17:00	4/16/2010 22:45	**14 days, 05 hours, 45 minutes**	341.75	14.2
12/2/2010 9:00	12/6/2010 7:30	**3 days, 22 hours, 30 minutes**	94.5	3.9

Severity
Sum the scores for Items 2-22. Items 1 and 24 are calculated separately.

From the MEPARI Protocol Manual 07-18-12 (p. 12)
The Wisconsin Upper Respiratory Symptom Survey (WURSS) is a validated questionnaire evaluating ARI-related symptom severity and quality of life impact.[259] Since WURSS was first developed by Dr. Barrett and colleagues in 2002,[189] more than 125 institutions in 37 countries have used the WURSS in their research. Initially, a 44-item version (WURSS-44) was assessed for reliability, responsiveness and importance to patients.[173] Psychometric analyses guided item reduction to yield the WURSS-21 which has been independently validated.[176] The WURSS-24 includes all WURSS-21 questions and 3 additional items assessing fever, headache and body aches - symptoms characteristic of influenza-like illness.[260]

Primary outcome

Barrett B, Brown RE, Mundt MP, Thomas GR, Barlow SK, Highstrom AD *et al* . Validation of a short form Wisconsin Upper Respiratory Symptom Survey (WURSS-21). *Health and Quality of Life Outcomes* 2009;**7**.

ATTENDANCE AT INTERVENTION SESSIONS

Attendance is collected at all intervention sessions. The instructors send the attendance records to the MEPARI staff, where the data is manually entered into REDCap and verified. Attendance data scoring is reflected in the REDCap Participant data example at the start of Appendix C (and the MEPARI 2 instrument manual).

7.2.7 Subgroup analyses ...Pre-planned secondary efficacy analyses will be conducted on the following sub-groups: 1) those who attend at least 7 of the 8 weekly training sessions ... One-sided testing will be based on the underlying hypotheses that: A) behavioral trainings are more likely to work for those who attend classes.

Appendix I: ... ARI episodes, missed work, etc [data] Per protocol (PP): Only people who attend at least 5 of the 9 exercise or mindfulness sessions will be included. For cohort 1 (2012-13) the following date will serve as cut-off for PP analysis: Oct. 22.

BIG FIVE INVENTORY

Computing Simple BFI Scale Scores
1=Disagree Strongly; 2=Disagree A Little; 3=Neither Agree or Disagree; 4=Agree a Little; 5=Agree Strongly

Reverse score the items labeled "R" and compute scale scores as the mean of the following items:

Extraversion (8 items): 1, 6R, 11, 16, 21R, 26, 31R, 36
Agreeableness (9 items): 2R, 7, 12R, 17, 22, 27R, 32, 37R, 42
Conscientiousness (9 items): 3, 8R, 13, 18R, 23R, 28, 33, 38, 43R
Neurotiscism (8 items): 4, 9R, 14, 19, 24R, 29, 34R, 39
Openness (10 items): 5, 10, 15, 20, 25, 30, 35R, 40, 41R, 44

Computing the content-balanced acquiesence index and Ipsatizing the BFI items
(Ipsatizing looks at the degree to which a participant answers pre-paired "opposite" characteristic questions in opposite directions.)
SPSS sytnax: compute within person response means [of raw score] and standard deviations for 3,8,9,13,18,19,23,24,28,29,34,39 as a Z-score.
Conscientiousness: 3 and 43, 8 and 13; 18 and 33; 23 and 28

Neurotiscism: 9 and 19; 24 and 29; 34 and 39

Instrument is administered at Run-in and Exit

From the MEPARI Protocol Manual 07-18-12 (p. 13, 16)
6.4.5 Big Five Inventory (BFI) Research on personality and health has been underway for some time, leading to various conceptual structures of state and trait psychological domains. The Big Five taxonomy has helped clarify and organize the links between personality, health behaviors, illness and mortality across the lifespan. Of the five dimensions measured (openness, conscientiousness, extraversion, agreeableness, and neuroticism), we will use baseline “conscientiousness” and “neuroticism” scores on the Big Five Inventory to gauge propensity for self-report bias on instrument completion activities, and to control for between person differences in multivariate efficacy models.

Covariate to control for between-person variability

John OP, Naumann LP, Soto CJ. Paradigm shift to the integrative big-five trait taxonomy: History, measurement and conceptual issues. In John OP, Robins RW, Pervin LA, eds. *Handbook of Personality: Theory and Research* , pp 114-58. New York: Guilford Press, 2008.

BIOMARKERS

Interleukin 6 secondary outcome, potential mediator
Interleukin 8 secondary outcome, potential mediator
IFN-induced Protein 10 secondary outcome, potential mediator
C-reactive Protein secondary outcome, potential mediator
Procalcitonin secondary outcome, potential mediator
Neutrophil potential mediator
HgA1C secondary outcome
Viral Identification secondary outcome, potential mediator

Outcomes: Blood and nasal wash samples will be obtained at baseline, one month after the end of the 8-week interventions, and once again three months later. Blood and nasal wash samples will be obtained approximately 24-72 hours into each ARI episode. Nasal wash samples will be tested with multiplex PCR (polymerase chain reaction) to identify etiological agents. Serum and nasal wash will be analyzed for interleukin-6, interleukin-8, C-reactive protein, procalcitonin, and interferon-gamma-induced protein 10. These inflammatory biomarkers will serve as objective indicators of disease severity to compare with illness severity self-reported on the WURSS-24 ... Inflammatory biomarkers ... will be analyzed as potential mediators of causal pathways leading from behavioral training interventions to ARI illness outcomes.

6.3.4 Viral identification will be done in Dr. Gern's lab, where high-throughput PCR-based multiplex methods have been developed and authenticated, and are able to identify nearly all of the pathogens associated with ARI illness [36;261-264] Trial will assess 2 samples, one done by self-swab at home, and the other by nasal wash at lab. We will also improve sample processing and include newly developed viral types. Dr. Gern's published data report that up to 91.4% of nasal washes from community-acquired ARI can yield positive viral IDs.[265]

6.3.5 Pro-inflammatory cytokines Laboratory-assessed objective measures will primarily serve to corroborate self-reports of disease severity. C-reactive protein (CRP) and procalcitonin (PCT) are well-established indicators of disease severity during respiratory infection, and can be measured in serum as well as in nasal wash.[113;115;118-120] Concentrations of interleukin-6 (IL-6)[266-271] and interleukin-8 (IL-8)[272-276] in nasal wash have been shown to correlate with illness severity. More recently, interferon-gamma-induced protein 10 (IP-10) has been shown to be measurably increased in both serum and nasal wash during times of acute viral ARI.[122-128] Inflammatory cytokines will be measured by ELISA methods in laboratories directed by Dr. Coe and Dr. Hayney.

6.3.6 Inflammatory tendency The same array of pro-inflammatory cytokines will also be analyzed as indicators of low level inflammation or pro-inflammatory tendency and as potential mediators of effects of behavioral interventions on ARI illness incidence, duration, and severity. The importance of CRP, PCT and IL-6 has been underscored by the ability of these pro-inflammatory biomarkers to predict mortality.[277-285] As potential mediators, pro-inflammatory cytokines (CRP, PCT, IL-6, IL-8, IP-10) will be assessed as change from baseline to one month after the 8 week behavioral interventions finish. Repeating these assays 3 months later will assess whether potential pro-inflammatory changes resulting from interventions will be sustained.

6.3.7 Polymorphonuclear neutrophil count in nasal mucus is a relatively well-established indicator of inflammation of the nasal epithelium.[286-290] Neutrophil counts correlate to symptom severity, viral titer and cytokine levels.[174;291] ... Neutrophil counts will be done on nasal wash collected during ARI episodes.

6.3.8 Glycosylated hemoglobin (HgA1C) Regular exercise is known to reduce hemoglobin A1C, a widely accepted indicator of average blood glucose levels.[142;292-294] There are at least two preliminary reports suggesting that mindfulness meditation might reduce HgA1C.[295;296] To explore these possibilities, we will assess HgA1C at baseline, 1 month after interventions, and again 3 months later.

For IL-6, a value of 0.01 indicates a level below detectable concentration, and for IP-10, a value of 1 indicates a level below detectable concentration.

BLOOD PRESSURE

Blood pressure is taken at Baseline, December, March and Exit visits.

American Heart Association …

Category	systolic, mmHg	diastolic, mmHg
Hypotension	<90	<60
Desired	90-119	60-79
Prehypertension	120-139	80-89
Stage 1 Hypertension	140-159	90-99
Stage 2 Hypertension	160-179	100-109
Hypertensive Crisis	$\geq$ 180	> 110

From the MEPARI Protocol Manual 07-18-12 (p. 15, p. 20)

6.4.24 Blood pressure Blood pressure is a well-recognized health indicator. There is some reason to believe that stress reduction or regular exercise might reduce blood pressure. In this study, blood pressure will be assessed at baseline and at both standardized follow-up periods using standard calibrated sphygmomanometers. Blood pressure will be analyzed as a secondary outcome using methods described in Section 7.

7.2.6 Secondary efficacy analyses Influence of interventions on secondary outcomes will be assessed using ANOVA-based multivariate regression models using SAS software.381-383 Adjustment for multiple comparisons will be incorporated, and interpretation will be cautious.238;384-388 In general, we will want to see relationships with p<0.01 in order to justify tentative null hypothesis rejection. Pre-planned secondary efficacy analyses will include effects of interventions on: ... 9) blood pressure ... 7.2.6.9 Those in the intervention groups will have lower blood pressure compared to control.

Secondary outcome

BODY MASS INDEX

Height (in decimalized inches) is captured at baseline visit; weight (in decimalized pounds) is captured at Baseline, December, March and Exit visits.

Data is entered from CRU flowsheets into corresponding REDCap "event".

Excel formula for calculating BMI: 703*weight/height^2

From the MEPARI Protocol Manual 07-18-12 (p. 12)
6.4.2 Body Mass Index (BMI) Body habitus is associated with many disease processes, and may be related to immune function and susceptibility to respiratory infection. Height will be assessed at baseline only. Weight will be measured at baseline, 1 and 4 months post-intervention, and at exit. Baseline BMI will be calculated and used as a covariate in statistical models. BMI will also be considered a secondary outcome of potential importance.

Secondary outcome, potential mediator

DEMOGRAPHICS

1 1 - Date of birth: Mo/Da/Year
2 Gender: Male = 1; Female = 2
3 Ethnicity (Hispanic/Latino origin): No = 0; Yes = 1
4 Race: 1= Black/African American; 2= Native Hawaiian or other Pacific Islander;
3= White/Caucasian; 4 = American Indian or Alaska Native; 5 = Asian; 6 = Other
5 Smoking history: 0 = Never Smoked; 1 = Past Smoker; 2 = Current smoker; 3 = 5 or fewer cigs;
4 = More than 5 cigs
6 Education: 1=Some high school; 2=High school grad/GED; 3=Some college/tech school; 4=College grad (bachelor's);
5=College post grad (master's, doctoral)
7 Household income: 1 = <$15K; 2 = $15-25K; 3 = $25-50K; 4 = $50-75K; 5 = $75-100K; 6 = Over $100K
8 Not applicable OR Salary (up to 4 jobs - 8a, 8b, 8c, 8d): Wages per hour/week/month/year; I work __ hours per week.*

Scanned document collected at run-in and an abbreviated version (Education/Household Income/Salary) at exit.

From the MEPARI Protocol Manual 07-18-12 (p. 13)
6.4.3 Demographic indicators Socioeconomic status is related to health and disease, including incidence and severity of respiratory infection. Demographic indicators to be assessed will include age, sex, years of education completed, household income, and number of children under the age of 18 living in the home. Age, sex and education will be used as covariates in multivariate efficacy analyses.

*Salary information will be averaged to garner an average hourly salary across all jobs and will be used in conjuction with missed days of work reported on weekly HCU forms.

Number of children under age 18 living in the home is garnered from the SNI (see separate instrument instructions).

Covariate to control for between-person variability

EXERCISE SELF-EFFICACY

This is an 18-item scale scored on a scale of 0-10, with participants indicating their confidence in their ability to perform exercise on a regular basis. A score of 0 indicates their level of confidence as "cannot do at all", while a score of 10 indicates their level of confidence is "certain can do". Items are summed for each participant. Range of scores is 0-180.

Higher scores indicate higher levels of self-efficacy.

Instrument is administered at Run-in Homework, December, February and April

When cleaning data, scanned "A" entries should be converted to "10".

From the MEPARI Protocol Manual 07-18-12 (p. 15)
6.4.18 Exercise Self Efficacy (ESES) Self-efficacy has been defined as "the belief in one's capabilities to organize and execute the courses of action required to manage prospective situations." The ESES scale was developed based on work by Bandura and colleagues, and has been validated by Shin, Kroll, and Everett. For our study, the ESES will be used to verify results of the exercise intervention, and to help explain potential mediational effects of exercise.

Secondary outcome, potential mediator

Everett B, Salamonson Y, Davidson PM. Bandura's exercise self-efficacy scale: Validation in an Australian cardiac rehabilitation setting. *International Journal of Nursing Studies* 2009;**46**:824-9.

(Bandura advocated for using a scale of 0-100).

EXERCISE LOG

Minutes of daily practice data for 7 days - Monday through Sunday - entered into REDCap. Categories as denoted in REDCap headings are moderate = 1 or vigorous = 2.

Practice minutes will be summed within each of the 2 categories.

Unless participants randomized to the exercise arm of the study request a weekly phone call from study personnel for assistance in completing their weekly practice logs, they are sent weekly email reminders to complete the electronic survey.

From the MEPARI Protocol Manual 07-18-12 (p. 15)

6.4.22 Mindfulness practice and exercise daily tracking log After the 8-week intervention, participants assigned to the meditation group will be asked to continue meditation at ≥150 minutes/week, in sessions of at least 10 minutes each. Similarly, those assigned to the exercise group will be asked to continue moderate intensity exercise at ≥150 minutes/week, in sessions of at least 10 minutes each. Using modified versions of practice logs developed at the University of Wisconsin by Dr. Davidson (meditation) and at Appalachian State by Dr. Niemann (exercise), study participants will record their practice once daily on a paper log and will enter their practice minutes once weekly through an on-line web-based data collection portal.

7.2.7 Subgroup analyses ...Pre-planned secondary efficacy analyses will be conducted on the following sub-groups: ... 2) those who continue to exercise or meditate for an average of at least 60 minutes per week after the trainings end... One-sided testing will be based on the underlying hypotheses that: A) behavioral trainings are more likely to work for those who attend classes, and B) the interventions will be more effective for more highly stressed people.

Potential mediator

EXPECTANCY

Run-in "Thinking Ahead"
Question order is being alternated; even numbers have exercise expectancy questions asked first; odd numbers have meditation.
Q1 and Q3: No = 0; Yes = 1
Q2 and Q4: 1 = much worse; 2 = somewhat worse; 3 = slightly worse; 4 = the same; 5 = slightly better; 6 = somewhat better; 7 = much better; 8 = very much better

Baseline Post-randomization AND Follow-up 1 Post-intervention "Thinking Ahead"
1 = much worse; 2 = somewhat worse; 3 = slightly worse; 4 = the same; 5 = slightly better; 6 = somewhat better; 7 = much better; 8 = very much better

Exit "Thinking Back"
Q1: No = 0; Yes = 1
Q2: 1 = much worse; 2 = somewhat worse; 3 = slightly worse; 4 = the same; 5 = slightly better; 6 = somewhat better; 7 = much better; 8 = very much better

When running report from REDCap, using the "Raw" data gives the variables as numbered above.

From the MEPARI Protocol Manual 07-18-12 (p. 15)
6.4.23 Expectancy In order to assess and potentially control for intervention-related expectancy, we will ask participants about their attitudes towards meditation and exercise before and after randomization, after the 8-week behavioral trainings, and at exit.

GPAQ

Equation: Total Physical Activity = [(1a * 1b * 8) + (2a * 2b * 4) + (3a * 3b * 4) + (4a * 4b * 8) + (5a * 5b * 4)]

High:
IF:(1a + 4a) >= 3 days AND Total physical activity MET minutes per week is >= 1500
OR
IF: (1a + 2a + 3a + 4a + 5a) >= 7 days AND total physical activity MET minutes per week is >= 3000
Moderate:
IF: (1a + 4a) >= 3 days AND ((1a * 1b) + (4a * 4b)) >= 60 minutes
OR
• IF: (P5 + P8 + P14) >= 5 days AND ((P5 * P6) + (P8 * P9) + (P14 * P15)>= 150 minutes
OR
• IF: (P2 + P5 + P8 + P11 + P14)>= 5 days AND Total physical activity MET minutes per week >= 600
Low F: the value does not reach the criteria for either high or moderate levels of physical activity

Data cleaning notes:
Convert 4b to minutes (i.e. 1 hour 45 minutes = 105 minutes)
Q6 should NOT be time-formatted and must convert 30 minutes to 50, 45 minutes to 75, etc., for algorithm to work (i.e., 3 hours 30 minutes = 350; 2 hours 45 minutes = 275)

Instrument is administered at Baseline, November, January, March, Exit

From the MEPARI Protocol Manual 07-18-12 (p. 15)
6.4.21 Global Physical Activity Questionnaire (GPAQ) The GPAQ was developed and validated through the World Health Organization, and displays excellent reliability and responsiveness characteristics.[255;256] GPAQ scores will be used to assess degree-of-change resulting from exercise training, and for dose-dependency and mediation analyses.

Potential mediator

Bull FC, Maslin TS, Armstrong T. Global physical activity questionnaire (GPAQ): nine country reliability and validity study. *J.Phys.Act.Health* 2009;**6**:790-804.

HEALTHCARE UTILIZATION/WEEKLY CHECK-IN

Unless participants request a weekly phone call from study personnel for assistance in completing a WEEKLY CHECK-IN survey, they are sent weekly email reminders to complete the electronic survey. Once the weekly check-in survey is accessed, the first question answered is whether or not participants have had cold symptoms in the previous week (Monday-Sunday), and if they answer affirmatively, they are told to call the study phone number, if they have not already done so. The survey also asks questions about their visits to healthcare providers (including type of provider visit - i.e., primary care, specialty clinic, urgent care, emergency room, other), overnight hospitalizations, and the reason for the visit/hospitalization. UWHC rates tied to the visit type will be used to calculate healthcare usage costs.

Specific details of the anticipated usage of missed-work data is found in the scoring instructions for the DEMOGRAPHIC questionnaire, but the gist is that it will be tied to the hourly wage the participant reports earning on the demographic questionnaire.

Determination of whether healthcare utilization or missed work are related to an ARI will be done by confirming via Jackson score. In instances where a participant reports a healthcare utilization and/or missed work, and has not called to report an ARI, but answers "yes" to the cold symptom question on the weekly check-in survey or offers an explanation of the reason for visiting a healthcare provider and/or missed work that is unclear on its potential connection to an ARI, blinded study personnel will contact the participant to ask for more information and make a

ARI-related medications will be captured on the final page of the RIDL booklets. The majority of this medication data will need to be hand-entered. Costs will be calculated using drugs.com or another similar online pricing guide.

From the MEPARI Protocol Manual 07-18-12 (p. 13)
6.4.6 Health care utilization and **antibiotics prescribed** Evaluation and treatment of ARI illness is very costly and often associated with unnecessary prescriptions, especially antibiotics. For this study, we plan to document total number of health care visits, ARI-related health care visits, and ARI-related prescriptions, including antibiotics. Each weekly communication will include the question, “Have you seen a doctor or visited a clinic, hospital or urgent care center?” Persons answering “Yes” will be asked the reason for the visit. Those answers will then be classified by study personnel as either “Related,” or “Unrelated” to ARI illness, including upper respiratory infection, influenza, pharyngitis, acute sinusitis, bronchitis, and pneumonia. All questionable cases will be verified by inspection of medical records (with case-specific participant permission). Prescriptions for antibiotics, prescription cough medicines, influenza antivirals and other ARI medications will be documented, as will self-reported use of nonprescription medications such as analgesics, antihistamines, decongestants, cough suppressants, and expectorants.

Secondary outcome

FEELING LOVED

The dichotomous A and B questions noted below are scored 0=no and 1=yes. The virtual analogue scale (VAS) values are entered as whole numbers ranging from 0-100.

Instrument is administered at Run-in Homework, December, February and April

From the MEPARI Protocol Manual 07-18-12 (p. 15)
6.4.17 Feeling Loved (FL) In addition to the validated perceived social support measures described above, we will use two novel questions with Yes/No response options: A) Do you feel loved? B) Do you love yourself? and two questions with visual analogue (VAS) response scales: How loved do you feel? How much do you love yourself? The ends of each 100mm VAS scale will be bounded by "not at all" and "very, very much."

Potential mediator

JACKSON

Sum the score of the symptoms. Range possibility of 0-24 (symptoms would only be assessed for severity if they are

Date of completion of Jackson screening may be utilized to determine start date of ARI episode in the event that RIDL data is incomplete.

When affirmative response is made to Question 1 on Weekly Check-in indicating presence of cold symptoms in the past week, confirming of a Jackson score is first step to ensure ARI data is being captured.

Jackson score is used to validate ARI-relatedness of healthcare utilization and missed work.

Administered when participant calls to report suspected ARI.

From the MEPARI Protocol Manual 07-18-12 (p. 12)
6.3.1 Definition of ARI illness The beginning of each ARI illness episode will be defined by: 1) answering "Yes" to either: "Do you think you have a cold" or "Do you think you are coming down with a cold?" AND 2) reporting at least 1 of 4 cold symptoms or synonyms: nasal discharge (runny nose); nasal obstruction (plugged or congested); sneezing; or sore (scratchy) throat, AND 3) scoring at least 2 points on the Jackson scale. The Jackson score is calculated by summing 8 symptom scores (sneezing, headache, malaise, chilliness, nasal discharge, nasal obstruction, sore throat and cough) rated , 0=absent, 1=mild, 2=moderate, and 3=severe.[170-172] In order for these symptoms to be classified as an ARI illness episode (and analyzed as such), at least 2 days in a row must meet these criteria. From the first day of ARI illness and forward each participant will fill out a daily WURSS-24 until they answer "No" to the question "Do you think that you are still sick with this respiratory infection?" for 2 days in a row. The last day the participant answers "Yes" will be the last day classified as ARI illness and included in the calculation of severity-weighted days of ARI illness.

Integral to determining ARI presence; however, not a primary outcome.

Jackson GG, Dowling HF, Spiesman IG, Boand AV. Transmission of the common cold to volunteers under controlled conditions. *Arch Intern Med* 1958;**101**:267-78.

MAAS

Descriptor taken from Brown 2003, p. 825
Respondents indicate how frequently they have the experience described in each statement using a 6-point Likert scale from 1 (almost always) to 6 (almost never), where high scores reflect more mindfulness.

Descriptor taken from L.E. Carlson and K.W. Brown, Validation of the Mindful Attention Awareness Scale in a cancer population, J Psychosom Res 58 (2005), pp. 29–33.
This 15-item scale measures the frequency of mindful states in day-to-day life, using both general and situation-specific statements. Individual scores are summed and then divided by 15 to determine the mean MAAS score, which can range from 1 to 6. Higher scores indicate greater mindfulness.

MEPARI 2 scoring follows Carlson instructions; mean is decimalized to the tenth.

Instrument is administered at Baseline, November, January, March and Exit.

From the MEPARI Protocol Manual 07-18-12 (p. 15)
6.4.20 Mindfulness Attention Awareness Scale (MAAS) For our study, we will use the 15-item MAAS[248;249] to assess effects of MBSR training, and to help understand/explain potential mediating influences of mindfulness on our major outcomes. The MSES and MAAS instruments will also serve as an intervention check, in that scores are expected to change more among those randomized to meditation than in the exercise or control groups.

Potential mediator

Carlson LE,.Brown KW. Validation of the Mindful Attention Awareness Scale in a cancer population. *J.Psychosom.Res.* 2005;**58**:29-33.

Brown KW,.Ryan RM. The benefits of being present: mindfulness and its role in psychological well-being. *J.Pers.Soc.Psychol.* 2003;84:822-48.

MEDITATION LOG

Minutes of daily practice data for 7 days - Monday through Sunday - entered into REDCap. Categories as denoted in REDCap headings are formal = 1 or informal = 2.

Practice minutes will be summed within each of the 2 categories.

Unless participants randomized to the Meditation arm of the study request a weekly phone call from study personnel for assistance in completing their weekly practice logs, they are sent weekly email reminders to complete the electronic

From the MEPARI Protocol Manual 07-18-12 (p. 15)
6.4.22 Mindfulness practice and exercise daily tracking log After the 8-week intervention, participants assigned to the meditation group will be asked to continue meditation at ≥150 minutes/week, in sessions of at least 10 minutes each. Similarly, those assigned to the exercise group will be asked to continue moderate intensity exercise at ≥150 minutes/week, in sessions of at least 10 minutes each. Using modified versions of practice logs developed at the University of Wisconsin by Dr. Davidson (meditation) and at Appalachian State by Dr. Niemann (exercise), study participants will record their practice once daily on a paper log and will enter their practice minutes once weekly

7.2.7 Subgroup analyses ...Pre-planned secondary efficacy analyses will be conducted on the following sub-groups: ... 2) those who continue to exercise or meditate for an average of at least 60 minutes per week after the trainings end... One-sided testing will be based on the underlying hypotheses that: A) behavioral trainings are more likely to work for those who attend classes, and B) the interventions will be more effective for more highly stressed people.

Potential mediator

MINDFULNESS SELF-EFFICACY SCALE

Responses are ranked as 0=Not at all; 1=A little; 2=Moderately; 3=A lot; 4=Completely

The MSES comprises seven generic subscales of self-efficacy:
1. Behaviour (items 1, 8, 15, 22, 29)
2. Cognition (items 2, 9, 16, 23, 30)
3. Interoception (items 3, 10, 17, 24, 31)
4. Affect (items 4, 11, 18, 25, 32)
5. Interpersonal (items 5, 12, 19, 26, 33)
6. Avoidance (items 6, 13, 20, 27, 34)
7. Mindfulness (items 7, 14, 21, 28, 35)

Before scale and global scores of self-efficacy can be calculated, 18 items must be scored in reverse.
These are:
4 5 6 8 11 14 16 17 22 23 25 26 27 28 29 30 34 35
LOOKUP(#REF!,{0,1,2,3,4},{4,3,2,1,0})
When the score for each of the 18 items listed above has been reversed, you can sum the scores for each of the 7 dimensions. The scale scores provide an estimate of Dimensional Self Efficacy (DSE) for each dimension. To calculate the Global Self Efficacy (GSE) score, sum all DSE scores.

The current lack of psychometric data for the MSES renders the following ranges very tentative. They are currently only a rough clinical guide and scores must be interpreted with caution.
0-34 Poor sense of self-efficacy
35-69 Weak sense of self-efficacy
70-104 Moderate sense of self-efficacy
105-140 Good sense of self-efficacy

The MSES was constructed to measure the change in levels of self-efficacy before, during, and following mindfulness-based therapy programs. Participants in these programs will relate more easily to some of the items presented, especially items 3 and 10 in Interoception subscale. This is because body scan tasks of such programs tend to increase interoceptive awareness and acceptance, whereas a number of non-experiential therapies do not. As a result, a person undergoing counseling or traditional cognitive therapy is likely to interpret a high score on item 3 as undesirable. The context in which this scale is being used is therefore a factor worth taking into account.

Instrument is administered at Run-in Homework, December, February and April

From the MEPARI Protocol Manual 07-18-12 (p. 15)
6.4.19 Mindfulness-based Self-Efficacy Scale (MSES) Research aimed at defining and assessing the concept of "mindfulness" is well underway, with several questionnaire instruments available.[367-374] The MSES is one of the more recent questionnaires, developed by Cayoun and Freestun to assess effects of MBSR training on perceived self-efficacy.[247] The MSES assesses 7 domains related to mindfulness self-efficacy, including behavior, cognition, interoception, affect, interpersonal, avoidance and mindfulness. The MSES will provide a nice counterpart to the ESES

Secondary outcome, potential mediator

http://www.mytherapysession.com/PDFs/MSESSelfEfficacyScale.pdf
This citation link for the 36-item MSES is broken. Current instrument availability is of the MSES-R, a revised 22-item instrument.

POSITIVE AFFECT/NEGATIVE AFFECT SCALE

The Positive Affect Negative Affect Schedule (PANAS) is a psychometric scale developed to measure the largely independent constructs of positive and negative affect, both as states and traits. Participants respond to 20 words by ascribing the degree to which they have felt them in the past few weeks. The options are 1 - very slightly or not at all; 2 - a little; 3 - moderately; 4 - quite a bit; 5 - extremely. Score for each affect direction (positive or negative) range between 10 and 50, with higher scores indicating stronger affect tendency.

Items 1, 3, 5, 9, 10, 12, 14, 16, 17 and 19 with high scores are indicative of a positive affect.
Items 2, 4, 6, 7, 8, 11, 13, 15, 18 and 20 with high scores are indicative of a negative affect.

Sum the scores for each of the numbers corresponding to their respective affect.

Instrument is administered at Run-in Homework, December, February and April

From the MEPARI Protocol Manual 07-18-12 (p. 14)

6.4.13 Positive and Negative Affect Schedule (PANAS) The widely used PANAS scale reliably assesses both positive and negative affect (emotion).[338] Self-reported positive and negative emotion have long been known to be independent predictors of psychological and physical health.[339] In the ARI setting, positive and negative emotion predict not only symptom expression, but actual infection as indicated by viral shedding.[9;13;340] In an RCT setting, PANAS scores improved after MBSR training, ($p < 0.05$) as compared to controls.[30]

Potential mediator

Watson D, Clark LA, Tellegen A. Development and validation of brief measures of positive and negative affect: the PANAS scales. *Journal of Personality & Social Psychology* 1988;**54**:1063-70.

PHQ-9

Participants indicate which statement best describes how much they've been bothered by the given 9 problems over the previous two weeks using a 4-point scale. Choices are: 0 (not at all), 1 (several days), 2 (more than half the days) and 3 (nearly every day); thus, summed scores can range from 0-27 with higher numbers indicative of depression.

Question 2 assesses functional health. Changes in scores after the intervention begins can show impact on patient's

Make sure scanned scoring reflects 0-3, not 1-4

Instrument is administered at Run-in, December, February and April

From the MEPARI Protocol Manual 07-18-12 (p. 14)
6.4.10 Depression screen (PHQ-9) The PHQ-9 is a widely used and well-validated depression screen,[322-327] and also demonstrates good responsiveness.[328;329] In our study, prospective participants with PHQ-9 scores of ≥15 will be excluded (and referred to appropriate clinical care). PHQ-9 scores will be assessed as secondary outcomes.

Secondary outcome

Kroenke K, Spitzer RL, Williams JBW. The PHQ-9: Validity of a brief depression severity measure. *J Gen Intern Med* 2001;**16**:606-13.

PITTSBURGH SLEEP QUALITY INDEX

Separate detailed scoring rubric exists.

Range of 0-21

The PSQI generates seven scores that correspond to these domains: Subjective Sleep Quality, Sleep Latency, Sleep Duration, Habitual Sleep Efficiency, Sleep Disturbances, Use of Sleep Medications, and Daytime Dysfunction. Each component score ranges from 0 (no difficulty) to 3 (severe difficulty). The component scores are summed to produce a global score (range of 0–21). A PSQI global score >5 is considered to be suggestive of significant sleep disturbance. Any missing data renders entire score null.

Be sure to check that values for scanned data for Questions 5a-5j range from 0-3, not 1-4 (need to convert otherwise.)

Responses to Questions 1 and 3 should be in military time (these values provide the time the participant spends in bed). Once the in-bed time has been calculated, it needs to be decimalized to be used in calculating a percentage of sleep efficiency. The sleep efficiency percentage algorithm uses a decimalized conversion of the response to Question 4. The % efficiency may be inverted positively (>100%) if estimates of self-reported hours of sleep per night are greater than what is derived from calculating the hours of sleep using the reported going to bed time and getting up time.

Instrument is administered at Baseline, November, January, March, Exit

From the MEPARI Protocol Manual 07-18-12 (p. 14)
Sleep quality may improve following interventions, and hence can be interpreted as a potentially important secondary outcome.

Secondary outcome, potential mediator

Buysse DJ, Reynolds CF, III, Monk TH, Berman SR, Kupfer DJ. The Pittsburgh Sleep Quality Index: a new instrument for psychiatric practice and research. *Psychiatry Research* 1989;**28**:193-213.

PSS-10

Gleaned from Cohen 1988
Participants indicate which statement best describes the frequency of their feelings and thoughts during the previous month using a 5-point scale. Choices are: 0 (never), 1 (almost never), 2 (sometimes), 3 (fairly often) and 4 (very often). Of the 10 items, 4 are worded in a positive direction and 6 are worded in a negative direction. After reversing the scoring for the positively worded items, item scores are summed to yield an overall perceived stress score with high scores representing greater perceived stress. Scores can range from 0-40.

Reverse scores for Items 4, 5, 7 and 8.
Excel algorithm for reversal: IF(*cell* =4,0,IF(*cell* =3,1,IF(*cell* =2,2,IF(*cell* =1,3,IF(*cell* =0,4)))))

Instructions for algorithm - replace the word "cell" with the actual cell correlates (A1, A2, A3, etc.)

Instrument is administered at Baseline, November, January, March, Exit

From the MEPARI Protocol Manual 07-18-12 (p. 14)
6.4.14 Perceived Stress Scale (PSS-10) The PSS-10 has been validated in multiple studies.[100-103;341-343] PSS scores predict rates of viral infection among volunteers inoculated with rhinovirus, and correlate with physiologic and self-report indicators of ARI illness, including nasal IL-6 level.[14;99-102] Because stress reduction is one of the hypothesized mechanisms of action, we have expanded our study population to include working-age participants, who we presume
7.2.7 Subgroup analyses ... Pre-planned secondary efficacy analyses will be conducted on the following sub-groups: ... 3) those who at baseline have perceived stress scores at least 14 points (PSS-10). One-sided testing will be based on the underlying hypotheses that: A) behavioral trainings are more likely to work for those who attend classes, and B) the interventions will be more effective for more highly stressed people.

Secondary outcome, potential mediator

http://www.ncsu.edu/assessment/resources/perceived_stress_scale.pdf
Norm Table from L. Harris Poll
Male Mean 12.1
Female Mean 13.7
Age
18-29 14.2
30-44 13.0
45-54 12.6
55-64 11.9
65 & older 12.0
Race
White 12.8
Hispanic 14.0
Black 14.7
Other minority 14.1

Cohen, S., & Williamson, G. (1988). Perceived stress in a probability sample of the United States. In S. Spacapam & S. Oskamp (Eds.), *The social psychology of health: Claremont Symposium on applied social psychology.* Newbury Park, CA:

SFS-12

Statistician generated scoring as part of instrument package purchase.

National mean norms for similarly aged adults using the SF-12…
Ages 30-34: Physical - 53.27; Mental - 48.90
Ages 35-44: Physical - 52.00; Mental - 48.79
Ages 45-54: Physical - 49.35; Mental - 49.90
Ages 55-64: Physical - 46.90; Mental - 50.84
Ages 65-69: Physical - 43.93; Mental - 51.57

Instrument is administered at Baseline, November, January, March, Exit

From the MEPARI Protocol Manual 07-18-12 (p. 14)
6.4.11 Health-related quality of life (SF-12) Also known as the Medical Outcomes Study Short Form, this 12-item questionnaire is commonly used to measure overall health, including physical (SF12-P) and mental health (SF12-M) subscales. It has been extensively assessed for reliability, responsiveness and criterion validity.[330-334] In our study, it will be used to assess potential changes in general physical and mental health due to interventions, and as a covariate to control for baseline between-person differences in multivariate efficacy analyses.

Secondary outcome, covariate to control for between-person variability.

Citation (separate book on Shari's shelf)
Ware, J.E. Jr., Kosinski, M., Turner-Bowker, D.M., Gandek, B. *User's Manual for the SF-12v2 Health Survey with a Supplement Documenting SF-12 Health Survey* Lincoln, RI: QualityMetric Incorporated, 2002.

SEATTLE INDEX OF COMORBIDITY

Respondents answer yes = 1 or no = 0 on nine health issues and self-report their smoking status.
SIC weighting formula = Age (in 5-year intervals) + Prior MI + 2*(Cancer) + Lung Disease + 2*(CHF) + 2*(Diabetes) + Pneumonia + 2*(Stroke) + 2*(Past Smoker) + 4*(Current smoker). 1 point was assigned for each 5-year interval above the age of 55.
age 30–54 = 0 points
55–59 = 1 point
60–64 = 2 points
65-69 = 3 points

Positive responses for individual items are also reported.

From Parimon T, Chien JW, Bryson CL, McDonell MB, Udris EM, Au DH. Inhaled corticosteroids and risk of lung cancer among patients with chronic obstructive pulmonary disease. Am J Respir Crit Care Med 2007;175, p. 713

The SIC is a weighted score derived from self-report of conditions and incorporates history of previous myocardial infarction, cancer, chronic lung disease, chronic heart failure, pneumonia, cerebral vascular accidents, and smoking status. Smoking status is obtained by self-report and categorized as never, past, and current smoker. MEPARI 2 instrument has 2 added items: allergies and asthma.

Instrument is administered at Run-in and Exit

From the MEPARI Protocol Manual 07-18-12 (p. 13)

6.4.4 Seattle Index of Comorbidity (SIC) People with diabetes, cardiovascular disease and pulmonary disease are known to have increased risks when infected with influenza or other respiratory viruses.[305-307] The SIC is a simple 8-item measure shown to predict hospitalization and mortality.[308] We will add items on allergy and asthma, as these illnesses are known to be related to severity of ARI. The modified SIC will be assessed at baseline and exit, and used as a covariate to control for possible influences of chronic disease on ARI outcomes. Relationships of individual items to outcomes and to other co-variates will be explored.

Covariate to control for between-person variability

Fan VS, Au D, Heagerty P, Deyo RA, McDonell MB, Fihn SD. Validation of case-mix measures derived from self-reports of diagnoses and health. *Journal of Clinical Epidemiology* 2002;**55**:371-80.

SOCIAL NETWORK INDEX

Role - there are 13 possible points (Roommate, Spouse, Parent, Child, Child-in-law, Close Relative, Close Friend, Religious Member, Group Member, Employee, Volunteer, Student, Neighbor)
High Contact - there are 14 possible points (Roommate, Spouse, Parent, Child, Child-in-law, Close Relative, Close Friend, Religious Member, Group Member, Employee Supervisor, Employee Peer, Volunteer, Student, Neighbor)
Potential Contact - there are 73 possible points (Roommate=1, Spouse=1, Parent=4, Child=2, Child-in-law=2, Close Relative=7, Close Friend=7, Religious Member=7, Group Member=7, Employee Supervisor=7, Employee Peer=7, Volunteer=7, Student=7, Neighbor=7)

Scoring details
Number of roles: for Question 1, if not "1", score 1 point; for Question 2, if "2", score 1 point; for Questions 3, 6, 7, 8, 9, 10, 11, 12, 13, one point is assigned for each type of relationship for which respondents answer other than "no" or "0"; for question 4, if not 2, score 1 point; for Question 5, if 1, recode to -99, if not 1, 3 or 4, score 1 point.
High contact role: for Question 2, if "2", score 1 point; for Questions 1, 3b, 6a, 7a, 8b, 9b, 10a, 10b, 11a, 12a, 13, one point is assigned if the participant answers anything but "no" or "none"; for Question 4a, if not "2", give 1 point; for Question 5a, if "1", recode to "-99", and if not "3", give 1 point.
9a, 10a, 10b, 11, 12a, 13, no recode; if Question 4 is 1=2, 2=0, 3 or 4=1; if Question 5 is 1, recode to -99, 2=2,3=0, 4 or 5=1

Reminder that in the MEPARI 2 data set, -99 indicates question was not applicable and -88 indicates missing data. This demands if using Excel to calculate the score, when tallying the total, to the sum, you must add the total of the -99 and -88 entries (i.e., if you had 3 answers with -99, you would add 297 to the total; if you had 3 answers of -99 and one with -88, you would add 385 to the total).

Items 3a and 3c are included as separate columns in dataset. Item 3a asks how many children live with participant >3 days/week on average. Answer values are None=0; 1=1; 2=2; 3 or more=3. Item 3c ascertains how many children ages 17 and under participant has contact in an average week. Answer values are: None=0; 1 to 5=1; 6 to 19=2; 20 or

Instrument is administered at Run-in Homework, December, February and April

From the MEPARI Protocol Manual 07-18-12 (p. 15)
6.4.16 Social Network Index (SNI) will serve to quantify social network size in order to help characterize social support. Cohen's research using the SNI suggests that the number of social contacts is predictive of susceptibility to ARI.[10;357] The SNI will also serve as an index of interpersonal contacts that could serve to transmit ARI virus. The SNI will be modified to document the number and ages of the children with whom participants have contact.

Demographic data regarding # of children under age 18 living more than half-time with participant is captured on SNI.

6.4.3 Demographic indicators Socioeconomic status is related to health and disease, including incidence and severity of respiratory infection.[102;303;304] Demographic indicators to be assessed will include age, sex, years of education completed, household income, and number of children under the age of 18 living in the home. Age, sex and education will be used as covariates in multivariate efficacy analyses

Potential mediator

Cohen S, Doyle WJ, Skoner DP, Rabin BS, Gwaltney JM. Social ties and susceptibility to the common cold. *JAMA* 1997;**277**:1940-4.

SOCIAL PROVISIONS SCALE

The instrument contains 24 items, four for each of the following: Attachment, Social Integration, Reassurance of Worth, Reliable Alliance, Guidance, and Opportunity for Nurturance. Half of the items describe the presence of a type of support and the others describe the absence of a type of support.

The respondent indicates on a 4-point scale the extent to which each statement describes her current social network. Responses range from 1 (strongly disagree) to 4 (strongly agree). After reversal of negatively worded items (indicated by an "R" below) a total score may be computed by summing all items. Subscale scores may be computed by summing items as follows:

- Attachment: Items 2R, 11, 17, and 21R
- Social Integration: Items 5, 8, 14R, and 22R
- Reassurance of Worth: 6R, 9R, 13, and 20
- Reliable Alliance: Items 1, 10R, 18R, and 23
- Guidance: Items 3R, 12, 16, and 19R
- Opportunity for Nurturance: 4, 7, 15R, and 24R

Scores Interpretation
A high score indicates a greater degree of perceived support.

http://www.iprc.unc.edu/longscan/pages/measures/Ages5to11/Social%20Provisions%20Scale.pdf

Instrument is administered at Baseline, November, January, March, Exit

From the MEPARI Protocol Manual 07-18-12 (p. 15)
6.4.15 Social Provisions Scale (SPS) [344-346] assesses perceived social support, which has been linked with a host of health and illness indicators.[347-352] The SPS is a 24-item index assessing 6 domains of social health: attachment, social integration, reassurance of worth, reliable alliance, guidance, and opportunity for nurturance. The SPS, developed by Russell and Cutrona,[344-346] predicts both immunological[353;354] and psychosocial outcomes.[355;356]

Potential mediator

Cutrona C, Russell D, Rose J. Social support and adaptation to stress by the elderly. *Psychology And Aging* . March 1986;1(1):47-54. Available from: PsycARTICLES, Ipswich, MA.

STANFORD PRESENTEEISM SCALE

Items receive a value of 1=strongly disagree; 2=somewhat disagree; 3=uncertain whether I disagree or agree; 4=somewhat agree; 5=strongly agree

Higher scores are indicative of a high level of presenteeism. Items 1, 3 and 4 are reverse coded.

http://www.drpelletier.com/chip/pdf/CHIP-stanford_presenteeism_scale.pdf

If participant does not answer Questions 1-6 and indicates not applicable, all answers should be filled in -99.
If participant answers Questions 1-6 AND answers not applicable below, disregard not applicable answer.

Instrument is administered at Run-in Homework, December, February and April.
A modified version is administered during an ARI.

From the MEPARI Protocol Manual 07-18-12 (p. 14)
6.4.9 Absenteeism/Presenteeism At enrollment we will assess employment, including type of work, hours per week worked, and compensation, assessed as hourly wage. Each week we will ask about any missed work, ascertain number of hours missed, and assess and classify reasons for missing work as either ARI-related or not ARI-related. The person making the classification will be blinded to allocation. Beyond missed work (absenteeism), illnesses such as ARI can decrease energy and focus at work,[260] leading to lost work productivity[318] from reduced "presenteeism."[319] To refine economic impact analysis, we will assess self-reported ability to perform work using the Stanford Presenteeism Scale (StPS).[320;321] The standard 1-month recall version will be administered at baseline, then 1, 3 and 5 months after interventions. A modified version with illness-specific recall will be used at end of each ARI episode, at the end of the RIDL questionnaire booklet (see 6.3.3 above).

Secondary outcome

Turpin RS, Ozminkowski RJ, Sharda CE, Collins JJ, Berger ML, Billotti GM *et al* . Reliability and validity of the Stanford Presenteeism Scale. *J.Occup.Environ.Med* 2004;**46**:1123-33.

TIMELINE FOLLOWBACK

The TLFB is a continuous measure of alcohol consumption based on recall of the prior two weeks, with 1 drink = 12 oz of beer, 5 oz of wine, and 1.5 oz of spirits. On the basis of the frequency and intensity of drinking, the TLFB measures the following: (1) days of heavy drinking in past 2 weeks, (2) number of drinking days in past 2 weeks, and (3) number of drinks per week. For MEPARI, tobacco usage is also calculated.

Instrument is administered at Baseline, November, January, March, Exit

From the MEPARI Protocol Manual 07-18-12 (p. 13)
6.4.1 Alcohol and tobacco use Tobacco use is associated with depressed immune function and increased rate and severity of acute respiratory infection (ARI).[297] Overuse of alcohol also appears to be associated with immune system depression and increased levels of ARI illness.[297] To assess and monitor both tobacco and alcohol use, we will use the validated and widely used Timeline Followback method (TLFB).[298-302] We will consider tobacco use and alcohol overuse as secondary outcomes of potential importance. Baseline tobacco use will be used as a covariate in multivariate

TLFB Alcohol - Secondary outcome
TLFB Smoking - Secondary outcome, covariate to control for between-person differences

Sobell LC, Agrawal S, Sobell MB, Leo GI, Young LJ, Cunningham JA *et al* . Comparison of a quick drinking screen with the timeline followback for individuals with alcohol problems. *Journal of Studies on Alcohol* 2003;**64**:858-61.

WURSS

Duration Formulas embedded in these cells in this document

Start Date and time	End Date and time	Calculate Duration	Convert to Hrs	Decimalized Days
4/2/2010 17:00	4/16/2010 22:45	**14 days, 05 hours, 45 minutes**	341.75	14.2
12/2/2010 9:00	12/6/2010 7:30	**3 days, 22 hours, 30 minutes**	94.5	3.9

Severity
Sum the scores for Items 2-22. Items 1 and 24 are calculated separately.

From the MEPARI Protocol Manual 07-18-12 (p. 12)
The Wisconsin Upper Respiratory Symptom Survey (WURSS) is a validated questionnaire evaluating ARI-related symptom severity and quality of life impact.[259] Since WURSS was first developed by Dr. Barrett and colleagues in 2002,[189] more than 125 institutions in 37 countries have used the WURSS in their research. Initially, a 44-item version (WURSS-44) was assessed for reliability, responsiveness and importance to patients.[173] Psychometric analyses guided item reduction to yield the WURSS-21 which has been independently validated.[176] The WURSS-24 includes all WURSS-21 questions and 3 additional items assessing fever, headache and body aches - symptoms characteristic of influenza-like illness.[260]

Primary outcome

Barrett B, Brown RE, Mundt MP, Thomas GR, Barlow SK, Highstrom AD *et al* . Validation of a short form Wisconsin Upper Respiratory Symptom Survey (WURSS-21). *Health and Quality of Life Outcomes* 2009;**7**.

BIOMARKERS

Interleukin 6 secondary outcome, potential mediator
Interleukin 8 secondary outcome, potential mediator
IFN-induced Protein 10 secondary outcome, potential mediator
C-reactive Protein secondary outcome, potential mediator
Procalcitonin secondary outcome, potential mediator
Neutrophil potential mediator
HgA1C secondary outcome
Viral Identification secondary outcome, potential mediator

Outcomes: Blood and nasal wash samples will be obtained at baseline, one month after the end of the 8-week interventions, and once again three months later. Blood and nasal wash samples will be obtained approximately 24-72 hours into each ARI episode. Nasal wash samples will be tested with multiplex PCR (polymerase chain reaction) to identify etiological agents. Serum and nasal wash will be analyzed for interleukin-6, interleukin-8, C-reactive protein, procalcitonin, and interferon-gamma-induced protein 10. These inflammatory biomarkers will serve as objective indicators of disease severity to compare with illness severity self-reported on the WURSS-24 ... Inflammatory biomarkers ... will be analyzed as potential mediators of causal pathways leading from behavioral training interventions to ARI illness outcomes.

6.3.4 Viral identification will be done in Dr. Gern's lab, where high-throughput PCR-based multiplex methods have been developed and authenticated, and are able to identify nearly all of the pathogens associated with ARI illness [36;261-264] Trial will assess 2 samples, one done by self-swab at home, and the other by nasal wash at lab. We will also improve sample processing and include newly developed viral types. Dr. Gern's published data report that up to 91.4% of nasal washes from community-acquired ARI can yield positive viral IDs.[265]

6.3.5 Pro-inflammatory cytokines Laboratory-assessed objective measures will primarily serve to corroborate self-reports of disease severity. C-reactive protein (CRP) and procalcitonin (PCT) are well-established indicators of disease severity during respiratory infection, and can be measured in serum as well as in nasal wash.[113;115;118-120] Concentrations of interleukin-6 (IL-6)[266-271] and interleukin-8 (IL-8)[272-276] in nasal wash have been shown to correlate with illness severity. More recently, interferon-gamma-induced protein 10 (IP-10) has been shown to be measurably increased in both serum and nasal wash during times of acute viral ARI.[122-128] Inflammatory cytokines will be measured by ELISA methods in laboratories directed by Dr. Coe and Dr. Hayney.

6.3.6 Inflammatory tendency The same array of pro-inflammatory cytokines will also be analyzed as indicators of low level inflammation or pro-inflammatory tendency and as potential mediators of effects of behavioral interventions on ARI illness incidence, duration, and severity. The importance of CRP, PCT and IL-6 has been underscored by the ability of these pro-inflammatory biomarkers to predict mortality.[277-285] As potential mediators, pro-inflammatory cytokines (CRP, PCT, IL-6, IL-8, IP-10) will be assessed as change from baseline to one month after the 8 week behavioral interventions finish. Repeating these assays 3 months later will assess whether potential pro-inflammatory changes resulting from interventions will be sustained.

6.3.7 Polymorphonuclear neutrophil count in nasal mucus is a relatively well-established indicator of inflammation of the nasal epithelium.[286-290] Neutrophil counts correlate to symptom severity, viral titer and cytokine levels.[174;291] ... Neutrophil counts will be done on nasal wash collected during ARI episodes.

6.3.8 Glycosylated hemoglobin (HgA1C) Regular exercise is known to reduce hemoglobin A1C, a widely accepted indicator of average blood glucose levels.[142;292-294] There are at least two preliminary reports suggesting that mindfulness meditation might reduce HgA1C.[295;296] To explore these possibilities, we will assess HgA1C at baseline, 1 month after interventions, and again 3 months later.

For IL-6, a value of 0.01 indicates a level below detectable concentration, and for IP-10, a value of 1 indicates a level below detectable concentration.

ATTENDANCE AT INTERVENTION SESSIONS

Attendance is collected at all intervention sessions. The instructors send the attendance records to the MEPARI staff, where the data is manually entered into REDCap and verified. Attendance data scoring is reflected in the REDCap Participant data example at the start of Appendix C (and the MEPARI 2 instrument manual).

7.2.7 Subgroup analyses ...Pre-planned secondary efficacy analyses will be conducted on the following sub-groups: 1) those who attend at least 7 of the 8 weekly training sessions ... One-sided testing will be based on the underlying hypotheses that: A) behavioral trainings are more likely to work for those who attend classes.

Appendix I: ... ARI episodes, missed work, etc [data] Per protocol (PP): Only people who attend at least 5 of the 9 exercise or mindfulness sessions will be included. For cohort 1 (2012-13) the following date will serve as cut-off for PP analysis: Oct. 22.

BIOMARKERS

Interleukin 6 secondary outcome, potential mediator
Interleukin 8 secondary outcome, potential mediator
IFN-induced Protein 10 secondary outcome, potential mediator
C-reactive Protein secondary outcome, potential mediator
Procalcitonin secondary outcome, potential mediator
Neutrophil potential mediator
HgA1C secondary outcome
Viral Identification secondary outcome, potential mediator

Outcomes: Blood and nasal wash samples will be obtained at baseline, one month after the end of the 8-week interventions, and once again three months later. Blood and nasal wash samples will be obtained approximately 24-72 hours into each ARI episode. Nasal wash samples will be tested with multiplex PCR (polymerase chain reaction) to identify etiological agents. Serum and nasal wash will be analyzed for interleukin-6, interleukin-8, C-reactive protein, procalcitonin, and interferon-gamma-induced protein 10. These inflammatory biomarkers will serve as objective indicators of disease severity to compare with illness severity self-reported on the WURSS-24 ... Inflammatory biomarkers ... will be analyzed as potential mediators of causal pathways leading from behavioral training interventions to ARI illness outcomes.

6.3.4 Viral identification will be done in Dr. Gern's lab, where high-throughput PCR-based multiplex methods have been developed and authenticated, and are able to identify nearly all of the pathogens associated with ARI illness [36;261-264] Trial will assess 2 samples, one done by self-swab at home, and the other by nasal wash at lab. We will also improve sample processing and include newly developed viral types. Dr. Gern's published data report that up to 91.4% of nasal washes from community-acquired ARI can yield positive viral IDs [265]

6.3.5 Pro-inflammatory cytokines Laboratory-assessed objective measures will primarily serve to corroborate self-reports of disease severity. C-reactive protein (CRP) and procalcitonin (PCT) are well-established indicators of disease severity during respiratory infection, and can be measured in serum as well as in nasal wash.[113;115;118-120] Concentrations of interleukin-6 (IL-6)[266-271] and interleukin-8 (IL-8)[272-276] in nasal wash have been shown to correlate with illness severity. More recently, interferon-gamma-induced protein 10 (IP-10) has been shown to be measurably increased in both serum and nasal wash during times of acute viral ARI.[122-128] Inflammatory cytokines will be measured by ELISA methods in laboratories directed by Dr. Coe and Dr. Hayney.

6.3.6 Inflammatory tendency The same array of pro-inflammatory cytokines will also be analyzed as indicators of low level inflammation or pro-inflammatory tendency and as potential mediators of effects of behavioral interventions on ARI illness incidence, duration, and severity. The importance of CRP, PCT and IL-6 has been underscored by the ability of these pro-inflammatory biomarkers to predict mortality.[277-285] As potential mediators, pro-inflammatory cytokines (CRP, PCT, IL-6, IL-8, IP-10) will be assessed as change from baseline to one month after the 8 week behavioral interventions finish. Repeating these assays 3 months later will assess whether potential pro-inflammatory changes resulting from interventions will be sustained.

6.3.7 Polymorphonuclear neutrophil count in nasal mucus is a relatively well-established indicator of inflammation of the nasal epithelium.[286-290] Neutrophil counts correlate to symptom severity, viral titer and cytokine levels.[174;291] ... Neutrophil counts will be done on nasal wash collected during ARI episodes.

6.3.8 Glycosylated hemoglobin (HgA1C) Regular exercise is known to reduce hemoglobin A1C, a widely accepted indicator of average blood glucose levels.[142;292-294] There are at least two preliminary reports suggesting that mindfulness meditation might reduce HgA1C.[295;296] To explore these possibilities, we will assess HgA1C at baseline, 1 month after interventions, and again 3 months later.

For IL-6, a value of 0.01 indicates a level below detectable concentration, and for IP-10, a value of 1 indicates a level below detectable concentration.

Jackson Scale

Participant ID ____________________

Date ____________________

1. DO YOU THINK YOU ARE COMING DOWN WITH A COLD and/or HAVE A COLD?
   1 ☐ Yes 0 ☐ No

2. DO YOU HAVE ANY OF THE FOLLOWING?
   1 ☐ nasal discharge (runny nose)
   2 ☐ nasal obstruction (plugged nose, stopped up nose, stuffiness)
   3 ☐ sneezing
   4 ☐ sore throat (scratchy throat)

3. WHEN DID YOUR SYMPTOMS START? ____________________

4. HOW WOULD YOU RATE YOUR SYMPTOM(S)?

a. sneezing
0 ☐ Absent
1 ☐ Mild
2 ☐ Moderate
3 ☐ Severe

b. malaise
0 ☐ Absent
1 ☐ Mild
2 ☐ Moderate
3 ☐ Severe

c. chilliness
0 ☐ Absent
1 ☐ Mild
2 ☐ Moderate
3 ☐ Severe

d. nasal discharge
0 ☐ Absent
1 ☐ Mild
2 ☐ Moderate
3 ☐ Severe

e. nasal obstruction
0 ☐ Absent
1 ☐ Mild
2 ☐ Moderate
3 ☐ Severe

f. sore throat
0 ☐ Absent
1 ☐ Mild
2 ☐ Moderate
3 ☐ Severe

g. cough
0 ☐ Absent
1 ☐ Mild
2 ☐ Moderate
3 ☐ Severe

h. headache
0 ☐ Absent
1 ☐ Mild
2 ☐ Moderate
3 ☐ Severe

SUM OF TOTAL POINTS (Absent=0; Mild=1; Moderate=2; Severe=3): ____________________

5. DOES THIS MEET CRITERIA FOR ARI? Criteria include: Must have at least 1 of the cold symptoms in Question 2 AND must have a total of at least 2 points on the Jackson scale.
   1 ☐ Yes 0 ☐ No

6. NASAL WASH APPT TIME ______________________

Collected By

- ☐ Amber Schemmel
- ☐ Joe Chase
- ☐ Mary Checovich
- ☐ Michele Gassman
- ☐ Shari Barlow
- ☐ Supriya Hayer

Jackson Scale

Participant ID ______________________

Date ______________________

1. DO YOU THINK YOU ARE COMING DOWN WITH A COLD and/or HAVE A COLD? ☐ Yes ☐ No

2. DO YOU HAVE ANY OF THE FOLLOWING?
- ☐ nasal discharge (runny nose)
- ☐ nasal obstruction (plugged nose, stopped up nose, stuffiness)
- ☐ sneezing
- ☐ sore throat (scratchy throat)

3. WHEN DID YOUR SYMPTOMS START? ______________________

4. HOW WOULD YOU RATE YOUR SYMPTOM(S)?

a. sneezing
- ☐ Absent
- ☐ Mild
- ☐ Moderate
- ☐ Severe

b. malaise
- ☐ Absent
- ☐ Mild
- ☐ Moderate
- ☐ Severe

c. chilliness
- ☐ Absent
- ☐ Mild
- ☐ Moderate
- ☐ Severe

d. nasal discharge
- ☐ Absent
- ☐ Mild
- ☐ Moderate
- ☐ Severe

e. nasal obstruction
- ☐ Absent
- ☐ Mild
- ☐ Moderate
- ☐ Severe

f. sore throat
- ☐ Absent
- ☐ Mild
- ☐ Moderate
- ☐ Severe

g. cough
- ☐ Absent
- ☐ Mild
- ☐ Moderate
- ☐ Severe

h. headache
- ☐ Absent
- ☐ Mild
- ☐ Moderate
- ☐ Severe

SUM OF TOTAL POINTS (Absent=0; Mild=1; Moderate=2; Severe=3): ______________________

5. DOES THIS MEET CRITERIA FOR ARI? Criteria include: Must have at least 1 of the cold symptoms in Question 2 AND must have a total of at least 2 points on the Jackson scale. ☐ Yes ☐ No

6. NASAL WASH APPT TIME ________________________________

Collected By

- ☐ Amber Schemmel
- ☐ Joe Chase
- ☐ Mary Checovich
- ☐ Michele Gassman
- ☐ Shari Barlow
- ☐ Supriya Hayer

ATTENTION (MAAS)

INSTRUCTIONS:

Below is a collection of statements about your everyday experience.

OK
98

Please indicate how frequently or infrequently you have each experience.

Please answer according to what really reflects your experience rather than what you think your experience should be.

Please think about each item separately from every other item.

		Almost Always (1)	Very Frequently (2)	Somewhat Frequently (3)	Somewhat Infrequently (4)	Very Infrequently (5)	Almost Never (6)
1	I could be experiencing some emotion and not be conscious of it until some time later.	○	○	○	○	○	○
2	I break or spill things because of carelessness, not paying attention, or thinking of something else.	○	○	○	○	○	○
3	I find it difficult to stay focused on what's happening in the present.	○	○	○	○	○	○
4	I tend to walk quickly to get where I'm going without paying attention to what I experience along the way.	○	○	○	○	○	○
5	I tend not to notice feelings of physical tension or discomfort until they really grab my attention.	○	○	○	○	○	○
6	I forget a person's name almost as soon as I've been told it for the first time.	○	○	○	○	○	○
7	It seems I am "running on automatic," without much awareness of what I'm doing.	○	○	○	○	○	○
8	I rush through activities without being really attentive to them.	○	○	○	○	○	○
9	I get so focused on the goal I want to achieve that I lose touch with what I m doing right now to get there.	○	○	○	○	○	○
10	I do jobs or tasks automatically, without being aware of what I'm doing.	○	○	○	○	○	○

PLEASE DO NOT WRITE IN THIS AREA

ATTENTION (MAAS)

		Almost Always 1	Very Frequently 2	Somewhat Frequently 3	Somewhat Infrequently 4	Very Infrequently 5	Almost Never 6
11	I find myself listening to someone with one ear, doing something else at the same time.	○	○	○	○	○	○
12	I drive places on 'automatic pilot' and then wonder why I went there.	○	○	○	○	○	○
13	I find myself preoccupied with the future or the past.	○	○	○	○	○	○
14	I find myself doing things without paying attention.	○	○	○	○	○	○
15	I snack without being aware that I'm eating.	○	○	○	○	○	○

PLEASE DO NOT WRITE IN THIS AREA

Participants

Participant ID ____________________

Screening ID ____________________

Cohort
- ☐ 1
- ☐ 2
- ☐ 3
- ☐ 4

Staff
- ☐ Amber Schemmel
- ☐ Mary Checovich
- ☐ Supriya Hayer

Participant Info

First Name ____________________

Middle ____________________

Last Name ____________________

Name Called ____________________

Home Phone ____________________

Cell Phone ____________________

Work Phone ____________________

Preferred Phone Number ☐ Home ☐ Cell ☐ Work

Phone Notes ____________________

Street ____________________

City ____________________

State ____________________

Zip ____________________

Email ____________________

MRN # ____________________

Date of Birth ____________________

Age ____________________

Gender ☐ Male ☐ Female

Enrollment

Randomization ☐ Control ☐ Meditation ☐ Exercise

Phone call for HCU ☐ Yes ☐ No

Class Assignment ☐ Tuesday ☐ Friday

Dropped Out ☐ Yes ☐ No

Date Dropped Out ________________________

Reason Dropped Out
☐ Time conflict
☐ Health issues
☐ Not interested
☐ Not willing
☐ Scheduling conflict
☐ Lost to followup
☐ Other

Other Reason Dropped Out ________________________

Appointment Scheduling

Baseline ________________________

Consent explained verbally, participant given time to read and ask questions ☐ Yes ☐ No

Flu Shot ________________________

Followup 1 ________________________

Follow-up #1 Visit Complete ☐ Yes ☐ No

Followup 2 ________________________

Follow-up #2 Visit Complete ☐ Yes ☐ No

Exit ________________________

Exit Visit Complete ☐ Yes ☐ No

OFF STUDY: Participant informed that participation is complete and that all contact info is the same (or notified of any changes) ☐ Yes ☐ No

Do you want to be contacted for future studies? ☐ Yes ☐ No

Visit Notes ________________________

Homework Booklets

Run-in ☐ Yes ☐ No

Run-in Homework ☐ Yes ☐ No

Baseline ☐ Yes ☐ No

November	☐ Yes ☐ No
December	☐ Yes ☐ No
January	☐ Yes ☐ No
February	☐ Yes ☐ No
March	☐ Yes ☐ No
April	☐ Yes ☐ No
May (Exit)	☐ Yes ☐ No
Exit	☐ Yes ☐ No
Homework Notes	__________________________________

Class Attendance

Intro Session	☐ Yes ☐ No
Week 1	☐ Yes ☐ No
Week 2	☐ Yes ☐ No
Week 3	☐ Yes ☐ No
Week 4	☐ Yes ☐ No
Week 5	☐ Yes ☐ No
Week 6	☐ Yes ☐ No
Retreat	☐ Yes ☐ No
Week 7	☐ Yes ☐ No
Week 8	☐ Yes ☐ No
Dropped Out of Class	☐ Yes ☐ No
Date Dropped Out	__________________________________
Continue to Get Logs	☐ Yes ☐ No
Reason Dropped Out	__________________________________
Notes	__________________________________

Flu Shot

Received Flu Shot	☐ Yes ☐ No
Date Flu Shot Administered	__________________________________
Flu Shot Notes	__________________________________

Cold/Flu

Cold/Flu Notes ________________

Jackson Scale Collected 1 ________________

Has Cold 1 ☐ Yes ☐ No

Schedule Nasal Wash 1 ☐ Yes ☐ No

Nasal Wash 1 Scheduled ________________

Why No Nasal Wash 1
- ☐ Called after 72 hours
- ☐ No weekend nasal washes available
- ☐ Participant too sick to come in
- ☐ Participant too busy to come in
- ☐ Participant out of town
- ☐ Holiday - CRU not open
- ☐ No Show
- ☐ Other

Nasal Wash Collected 1 ☐ Yes ☐ No

Envelope 1 ☐ Sent ☐ Received

Jackson Scale Collected 2 ________________

Has Cold 2 ☐ Yes ☐ No

Schedule Nasal Wash 2 ☐ Yes ☐ No

Nasal Wash 2 Scheduled ________________

Why No Nasal Wash 2
- ☐ Called after 72 hours
- ☐ No weekend nasal washes available
- ☐ Participant too sick to come in
- ☐ Participant too busy to come in
- ☐ Participant out of town
- ☐ Holiday - CRU not open
- ☐ No Show
- ☐ Other

Nasal Wash Collected 2 ☐ Yes ☐ No

Envelope 2 ☐ Sent ☐ Received

Jackson Scale Collected 3 ________________

Has Cold 3 ☐ Yes ☐ No

Schedule Nasal Wash 3 ☐ Yes ☐ No

Nasal Wash 3 Scheduled ________________

Why No Nasal Wash 3
- ☐ Called after 72 hours
- ☐ No weekend nasal washes available
- ☐ Participant too sick to come in
- ☐ Participant too busy to come in
- ☐ Participant out of town
- ☐ Holiday - CRU not open
- ☐ No Show
- ☐ Other

Nasal Wash Collected 3 ☐ Yes ☐ No

Envelope 3 ☐ Sent ☐ Received

Jackson Scale Collected 4 ______________________

Has Cold 4 ☐ Yes ☐ No

Schedule Nasal Wash 4 ☐ Yes ☐ No

Nasal Wash 4 Scheduled ______________________

Why No Nasal Wash 4
☐ Called after 72 hours
☐ No weekend nasal washes available
☐ Participant too sick to come in
☐ Participant too busy to come in
☐ Participant out of town
☐ Holiday - CRU not open
☐ No Show
☐ Other

Nasal Wash Collected 4 ☐ Yes ☐ No

Envelope 4 ☐ Sent ☐ Received

Jackson Scale Collected 5 ______________________

Has Cold 5 ☐ Yes ☐ No

Schedule Nasal Wash 5 ☐ Yes ☐ No

Nasal Wash 5 Scheduled ______________________

Why No Nasal Wash 5
☐ Called after 72 hours
☐ No weekend nasal washes available
☐ Participant too sick to come in
☐ Participant too busy to come in
☐ Participant out of town
☐ Holiday - CRU not open
☐ No Show
☐ Other

Nasal Wash Collected 5 ☐ Yes ☐ No

Envelope 5 ☐ Sent ☐ Received

Jackson Scale Collected 6 ______________________

Has Cold 6 ☐ Yes ☐ No

Schedule Nasal Wash 6 ☐ Yes ☐ No

Nasal Wash 6 Scheduled ______________________

Why No Nasal Wash 6
☐ Called after 72 hours
☐ No weekend nasal washes available
☐ Participant too sick to come in
☐ Participant too busy to come in
☐ Participant out of town
☐ Holiday - CRU not open
☐ No Show
☐ Other

Nasal Wash Collected 6 ☐ Yes ☐ No

Envelope 6 ☐ Sent ☐ Received

Jackson Scale Collected 7 ______________________

Has Cold 7 ☐ Yes ☐ No

Schedule Nasal Wash 7 ☐ Yes ☐ No

Nasal Wash 7 Scheduled ________________________________

Why No Nasal Wash 7
☐ Called after 72 hours
☐ No weekend nasal washes available
☐ Participant too sick to come in
☐ Participant too busy to come in
☐ Participant out of town
☐ Holiday - CRU not open
☐ No Show
☐ Other

Nasal Wash Collected 7 ☐ Yes ☐ No

Envelope 7 ☐ Sent ☐ Received

Jackson Scale Collected 8 ________________________________

Has Cold 8 ☐ Yes ☐ No

Schedule Nasal Wash 8 ☐ Yes ☐ No

Nasal Wash 8 Scheduled ________________________________

Why No Nasal Wash 8
☐ Called after 72 hours
☐ No weekend nasal washes available
☐ Participant too sick to come in
☐ Participant too busy to come in
☐ Participant out of town
☐ Holiday - CRU not open
☐ No Show
☐ Other

Nasal Wash Collected 8 ☐ Yes ☐ No

Envelope 8 ☐ Sent ☐ Received

Jackson Scale Collected 9 ________________________________

Has Cold 9 ☐ Yes ☐ No

Schedule Nasal Wash 9 ☐ Yes ☐ No

Nasal Wash 9 Scheduled ________________________________

Why No Nasal Wash 9
☐ Called after 72 hours
☐ No weekend nasal washes available
☐ Participant too sick to come in
☐ Participant too busy to come in
☐ Participant out of town
☐ Holiday - CRU not open
☐ No Show
☐ Other

Nasal Wash Collected 9 ☐ Yes ☐ No

Envelope 9 ☐ Sent ☐ Received

Jackson Scale Collected 10 ________________________________

Has Cold 10 ☐ Yes ☐ No

Schedule Nasal Wash 10 ☐ Yes ☐ No

Nasal Wash 10 Scheduled ________________________________

Why No Nasal Wash 10

- [ ] Called after 72 hours
- [ ] No weekend nasal washes available
- [ ] Participant too sick to come in
- [ ] Participant too busy to come in
- [ ] Participant out of town
- [ ] Holiday - CRU not open
- [ ] No Show
- [ ] Other

Nasal Wash Collected 10 — [ ] Yes [ ] No

Envelope 10 — [ ] Sent [ ] Received

Participants

Participant ID ______________________

Screening ID ______________________

Cohort
- ☐ 1
- ☐ 2
- ☐ 3
- ☐ 4

Staff
- ☐ Amber Schemmel
- ☐ Mary Checovich
- ☐ Supriya Hayer

Participant Info

First Name ______________________

Middle ______________________

Last Name ______________________

Name Called ______________________

Home Phone ______________________

Cell Phone ______________________

Work Phone ______________________

Preferred Phone Number 1 ☐ Home 2 ☐ Cell 3 ☐ Work

Phone Notes ______________________

Street ______________________

City ______________________

State ______________________

Zip ______________________

Email ______________________

MRN # ______________________

Date of Birth ______________________

Age ______________________

Gender 1 ☐ Male 2 ☐ Female

Enrollment

Randomization	☐ 1 ☐ 2 ☐ 3
Phone call for HCU	1 ☐ Yes 0 ☐ No
Class Assignment	4 ☐ Tuesday 5 ☐ Friday
Dropped Out	1 ☐ Yes 0 ☐ No
Date Dropped Out	______________
Reason Dropped Out	2 ☐ Time conflict<br>3 ☐ Health issues<br>4 ☐ Not interested<br>5 ☐ Not willing<br>6 ☐ Scheduling conflict<br>7 ☐ Lost to followup<br>8 ☐ Other
Other Reason Dropped Out	______________

Appointment Scheduling

Baseline	______________
Consent explained verbally, participant given time to read and ask questions	1 ☐ Yes 0 ☐ No
Flu Shot	______________
Followup 1	______________
Follow-up #1 Visit Complete	1 ☐ Yes 0 ☐ No
Followup 2	______________
Follow-up #2 Visit Complete	1 ☐ Yes 0 ☐ No
Exit	______________
Exit Visit Complete	1 ☐ Yes 0 ☐ No
OFF STUDY: Participant informed that participation is complete and that all contact info is the same (or notified of any changes)	1 ☐ Yes 0 ☐ No
Do you want to be contacted for future studies?	1 ☐ Yes 0 ☐ No
Visit Notes	______________

Homework Booklets

Run-in	1 ☐ Yes 0 ☐ No
Run-in Homework	1 ☐ Yes 0 ☐ No
Baseline	1 ☐ Yes 0 ☐ No

November	1 ☐ Yes	0 ☐ No
December	1 ☐ Yes	0 ☐ No
January	1 ☐ Yes	0 ☐ No
February	1 ☐ Yes	0 ☐ No
March	1 ☐ Yes	0 ☐ No
April	1 ☐ Yes	0 ☐ No
May (Exit)	1 ☐ Yes	0 ☐ No
Exit	1 ☐ Yes	0 ☐ No
Homework Notes	____________________	

Class Attendance

Intro Session	1 ☐ Yes	0 ☐ No
Week 1	1 ☐ Yes	0 ☐ No
Week 2	1 ☐ Yes	0 ☐ No
Week 3	1 ☐ Yes	0 ☐ No
Week 4	1 ☐ Yes	0 ☐ No
Week 5	1 ☐ Yes	0 ☐ No
Week 6	1 ☐ Yes	0 ☐ No
Retreat	1 ☐ Yes	0 ☐ No
Week 7	1 ☐ Yes	0 ☐ No
Week 8	1 ☐ Yes	0 ☐ No
Dropped Out of Class	1 ☐ Yes	0 ☐ No
Date Dropped Out	____________________	
Continue to Get Logs	1 ☐ Yes	0 ☐ No
Reason Dropped Out	____________________	
Notes	____________________	

Flu Shot

Received Flu Shot	1 ☐ Yes	0 ☐ No
Date Flu Shot Administered	____________________	
Flu Shot Notes	____________________	

Cold/Flu

Cold/Flu Notes ________________

Jackson Scale Collected 1 ________________

Has Cold 1 — 1 ☐ Yes 0 ☐ No

Schedule Nasal Wash 1 — 1 ☐ Yes 0 ☐ No

Nasal Wash 1 Scheduled ________________

Why No Nasal Wash 1
1 ☐ Called after 72 hours
2 ☐ No weekend nasal washes available
3 ☐ Participant too sick to come in
4 ☐ Participant too busy to come in
5 ☐ Participant out of town
6 ☐ Holiday - CRU not open
7 ☐ No Show
8 ☐ Other

Nasal Wash Collected 1 — 1 ☐ Yes 0 ☐ No

Envelope 1 — 1 ☐ Sent 0 ☐ Received

Jackson Scale Collected 2 ________________

Has Cold 2 — 1 ☐ Yes 0 ☐ No

Schedule Nasal Wash 2 — 1 ☐ Yes 0 ☐ No

Nasal Wash 2 Scheduled ________________

Why No Nasal Wash 2
1 ☐ Called after 72 hours
2 ☐ No weekend nasal washes available
3 ☐ Participant too sick to come in
4 ☐ Participant too busy to come in
5 ☐ Participant out of town
6 ☐ Holiday - CRU not open
7 ☐ No Show
8 ☐ Other

Nasal Wash Collected 2 — 1 ☐ Yes 0 ☐ No

Envelope 2 — 1 ☐ Sent 0 ☐ Received

Jackson Scale Collected 3 ________________

Has Cold 3 — 1 ☐ Yes 0 ☐ No

Schedule Nasal Wash 3 — 1 ☐ Yes 0 ☐ No

Nasal Wash 3 Scheduled ________________

Why No Nasal Wash 3
1 ☐ Called after 72 hours
2 ☐ No weekend nasal washes available
3 ☐ Participant too sick to come in
4 ☐ Participant too busy to come in
5 ☐ Participant out of town
6 ☐ Holiday - CRU not open
7 ☐ No Show
8 ☐ Other

Nasal Wash Collected 3 — 1 ☐ Yes 0 ☐ No

Envelope 3	1 ☐ Sent 0 ☐ Received
Jackson Scale Collected 4	________________________
Has Cold 4	1 ☐ Yes 0 ☐ No
Schedule Nasal Wash 4	1 ☐ Yes 0 ☐ No
Nasal Wash 4 Scheduled	________________________
Why No Nasal Wash 4	1 ☐ Called after 72 hours<br>2 ☐ No weekend nasal washes available<br>3 ☐ Participant too sick to come in<br>4 ☐ Participant too busy to come in<br>5 ☐ Participant out of town<br>6 ☐ Holiday - CRU not open<br>7 ☐ No Show<br>8 ☐ Other
Nasal Wash Collected 4	1 ☐ Yes 0 ☐ No
Envelope 4	1 ☐ Sent 0 ☐ Received
Jackson Scale Collected 5	________________________
Has Cold 5	1 ☐ Yes 0 ☐ No
Schedule Nasal Wash 5	1 ☐ Yes 0 ☐ No
Nasal Wash 5 Scheduled	________________________
Why No Nasal Wash 5	1 ☐ Called after 72 hours<br>2 ☐ No weekend nasal washes available<br>3 ☐ Participant too sick to come in<br>4 ☐ Participant too busy to come in<br>5 ☐ Participant out of town<br>6 ☐ Holiday - CRU not open<br>7 ☐ No Show<br>8 ☐ Other
Nasal Wash Collected 5	1 ☐ Yes 0 ☐ No
Envelope 5	1 ☐ Sent 0 ☐ Received
Jackson Scale Collected 6	________________________
Has Cold 6	1 ☐ Yes 0 ☐ No
Schedule Nasal Wash 6	1 ☐ Yes 0 ☐ No
Nasal Wash 6 Scheduled	________________________
Why No Nasal Wash 6	1 ☐ Called after 72 hours<br>2 ☐ No weekend nasal washes available<br>3 ☐ Participant too sick to come in<br>4 ☐ Participant too busy to come in<br>5 ☐ Participant out of town<br>6 ☐ Holiday - CRU not open<br>7 ☐ No Show<br>8 ☐ Other
Nasal Wash Collected 6	1 ☐ Yes 0 ☐ No
Envelope 6	1 ☐ Sent 0 ☐ Received
Jackson Scale Collected 7	________________________

Has Cold 7 | 1 ☐ Yes 0 ☐ No

Schedule Nasal Wash 7 | 1 ☐ Yes 0 ☐ No

Nasal Wash 7 Scheduled | __________________

Why No Nasal Wash 7
- 1 ☐ Called after 72 hours
- 2 ☐ No weekend nasal washes available
- 3 ☐ Participant too sick to come in
- 4 ☐ Participant too busy to come in
- 5 ☐ Participant out of town
- 6 ☐ Holiday - CRU not open
- 7 ☐ No Show
- 8 ☐ Other

Nasal Wash Collected 7 | 1 ☐ Yes 0 ☐ No

Envelope 7 | 1 ☐ Sent 0 ☐ Received

Jackson Scale Collected 8 | __________________

Has Cold 8 | 1 ☐ Yes 0 ☐ No

Schedule Nasal Wash 8 | 1 ☐ Yes 0 ☐ No

Nasal Wash 8 Scheduled | __________________

Why No Nasal Wash 8
- 1 ☐ Called after 72 hours
- 2 ☐ No weekend nasal washes available
- 3 ☐ Participant too sick to come in
- 4 ☐ Participant too busy to come in
- 5 ☐ Participant out of town
- 6 ☐ Holiday - CRU not open
- 7 ☐ No Show
- 8 ☐ Other

Nasal Wash Collected 8 | 1 ☐ Yes 0 ☐ No

Envelope 8 | 1 ☐ Sent 0 ☐ Received

Jackson Scale Collected 9 | __________________

Has Cold 9 | 1 ☐ Yes 0 ☐ No

Schedule Nasal Wash 9 | 1 ☐ Yes 0 ☐ No

Nasal Wash 9 Scheduled | __________________

Why No Nasal Wash 9
- 1 ☐ Called after 72 hours
- 2 ☐ No weekend nasal washes available
- 3 ☐ Participant too sick to come in
- 4 ☐ Participant too busy to come in
- 5 ☐ Participant out of town
- 6 ☐ Holiday - CRU not open
- 7 ☐ No Show
- 8 ☐ Other

Nasal Wash Collected 9 | 1 ☐ Yes 0 ☐ No

Envelope 9 | 1 ☐ Sent 0 ☐ Received

Jackson Scale Collected 10 | __________________

Has Cold 10 | 1 ☐ Yes 0 ☐ No

Schedule Nasal Wash 10 | 1 ☐ Yes 0 ☐ No

Nasal Wash 10 Scheduled | __________________

Why No Nasal Wash 10	1 ☐ Called after 72 hours<br>2 ☐ No weekend nasal washes available<br>3 ☐ Participant too sick to come in<br>4 ☐ Participant too busy to come in<br>5 ☐ Participant out of town<br>6 ☐ Holiday - CRU not open<br>7 ☐ No Show<br>8 ☐ Other
Nasal Wash Collected 10	1 ☐ Yes 0 ☐ No
Envelope 10	1 ☐ Sent 0 ☐ Received

MINDFULNESS SELF-EFFICACY SCALE

INSTRUCTIONS:

Fill in the circle below the answer that reflects how much you agree or disagree with each statement.

Try not to spend too much time on any one item. There are no right or wrong answers.

		Not at all	A little	Moderately	A lot	Completely
		0	1	2	3	4
1	I am able to think about what I am about to do before I act	○	○	○	○	○
2	When an unpleasant thought enters my mind, I can cope with it	○	○	○	○	○
3	When I relax, I can feel sensations in my body	○	○	○	○	○
4	I get easily overwhelmed by my emotions	○	○	○	○	○
5	I find it difficult to make new friends	○	○	○	○	○
6	I try to avoid uncomfortable situations even when they are really important	○	○	○	○	○
7	I am aware when I'm about to do something that could hurt me or someone else	○	○	○	○	○
8	Stopping myself from engaging in unwanted or hurtful behaviors is very difficult	○	○	○	○	○
9	I know that my thoughts don't have the power to hurt me	○	○	○	○	○
10	When I am stressed, I am aware of unpleasant body sensations	○	○	○	○	○
11	When I feel very emotional, it takes a long time for it to pass	○	○	○	○	○
12	I feel comfortable saying sorry when I feel I am in the wrong	○	○	○	○	○
13	It is ok for me to feel strong emotions	○	○	○	○	○
14	It is often too late when I realize I overreacted in a stressful situation	○	○	○	○	○
15	If something needs to be done, I am able to complete it within a reasonable time	○	○	○	○	○
16	I get so caught up in my thoughts that I end up feeling very sad or anxious	○	○	○	○	○

		0	1	2	3	4
	**MINDFULNESS, CONT'D**	**Not at all**	**A little**	**Moderately**	**A lot**	**Completely**
17	When I have unpleasant feelings in my body, I prefer to push them away	○	○	○	○	○
18	I believe that I can make my life peaceful	○	○	○	○	○
19	I can resolve problems easily with my partner (or best friend if single)	○	○	○	○	○
20	I can face my thoughts, even if they are unpleasant	○	○	○	○	○
21	I am tolerant with myself when I am repeating old habits that are no longer helpful	○	○	○	○	○
22	My actions are often controlled by other people or circumstances	○	○	○	○	○
23	I get caught up in unpleasant memories or anxious thoughts about the future	○	○	○	○	○
24	I can deal with physical discomfort	○	○	○	○	○
25	I feel I cannot love anyone	○	○	○	○	○
26	I am often in conflict with one (or more) family member	○	○	○	○	○
27	I avoid feeling my body when there is pain or other discomfort	○	○	○	○	○
28	I find it difficult to accept unpleasant experiences	○	○	○	○	○
29	I do things that make me feel good straightaway even if I will feel bad later	○	○	○	○	○
30	When I have a problem, I tend to believe it will ruin my whole life	○	○	○	○	○
31	When I feel physical discomfort, I relax because I know it will pass	○	○	○	○	○
32	Even when things are difficult I can feel happy	○	○	○	○	○
33	I can feel comfortable around people	○	○	○	○	○
34	Seeing or hearing someone with strong emotions is unbearable to me	○	○	○	○	○
35	If I get angry or anxious, it is generally because of others	○	○	○	○	○

INSTRUMENT SCORING

MEPARI 2 - ORDER OF DOCUMENTATION

PDF Page #	Binder Section	
3	1	Participant Database Contents/Attendance
12	2	BFI
15	3	Biomarker Data (Labs, BMI, BP)
18	4	Demographic
20	5	ESES
22	6	Expectancy
25	7	Feeling Loved
27	8	GPAQ
30	9	Healthcare Utilization/Weekly Check-in
35	10	Jackson
38	11	MAAS
41	12	MSES
44	13	PANAS
46	14	PHQ-9
48	15	Practice Logs (Exercise and Meditation)
51	15A	PSQI
54	14A	PSS-10
56	13A	SFS-12
59	12A	SIC
61	11A	SNI
65	10A	SPS
68	9A	Stanford Presenteeism
70	8A	TLFB
72	7A	WURSS/RIDL

7/15/2013

x

EMOTIONS

INSTRUCTIONS:

This scale consists of a number of words that describe different feelings and emotions.

Read each item and then fill in the circle of the appropriate answer which indicates to what extent you have felt this way **during the past few weeks**.

		1	2	3	4	5
		**Very slightly or not at all**	**A little**	**Moderately**	**Quite a bit**	**Extremely**
1	Interested	○	○	○	○	○
2	Distressed	○	○	○	○	○
3	Excited	○	○	○	○	○
4	Upset	○	○	○	○	○
5	Strong	○	○	○	○	○
6	Guilty	○	○	○	○	○
7	Scared	○	○	○	○	○
8	Hostile	○	○	○	○	○
9	Enthusiastic	○	○	○	○	○
10	Proud	○	○	○	○	○
11	Irritable	○	○	○	○	○
12	Alert	○	○	○	○	○
13	Ashamed	○	○	○	○	○
14	Inspired	○	○	○	○	○
15	Nervous	○	○	○	○	○
16	Determined	○	○	○	○	○
17	Attentive	○	○	○	○	○
18	Jittery	○	○	○	○	○
19	Active	○	○	○	○	○
20	Afraid	○	○	○	○	○

Watson D ; Clark LA ; Tellegen A. Development and validation of brief measures of positive and negative affect: the PANAS scales. Journal of Personality and Social Psychology 1988;54(6):1063-1070.
© Copyright David Watson and Lee Anna Clark

PHQ-9 SCREEN

INSTRUCTIONS:

Read each item carefully and fill in the circle which best describes how often you have been bothered by any of the following problems **over the last two weeks**.

		Not at all 0	Several days 1	More than half the days 2	Nearly every day 3
1	Little interest or pleasure in doing things	○	○	○	○
2	Feeling down, depressed, or hopeless	○	○	○	○
3	Trouble falling asleep, staying asleep, or sleeping too much	○	○	○	○
4	Feeling tired or having little energy	○	○	○	○
5	Poor appetite or overeating	○	○	○	○
6	Feeling bad about yourself, feeling that you are a failure, or feeling that you have let yourself or your family down	○	○	○	○
7	Trouble concentrating on things such as reading the newspaper or watching television	○	○	○	○
8	Moving or speaking so slowly that other people could have noticed. Or being so fidgety or restless that you have been moving around a lot more than usual	○	○	○	○
9	Thinking that you would be better off dead or that you want to hurt yourself in some way	○	○	○	○

If you answered anything besides "Not at all" for any of the previous statements, please read the following question carefully and fill in the appropriate response circle.

		Not difficult at all	Somewhat difficult	Very difficult	Extremely difficult
a	How difficult have these problems made it for you to do your work, take care of things at home, or get along with other people?	0 ○	1 ○	2 ○	3 ○

Log

Participant ID ________________________________

Start Date ________________________________

End Date ________________________________

Submitted ________________________________

Minutes

Day 1 Minutes 1 (moderate/formal) ________________________________

Day 1 Minutes 2 (vigorous / informal) ________________________________

Day 2 Minutes 1 (moderate/formal) ________________________________

Day 2 Minutes 2 (vigorous / informal) ________________________________

Day 3 Minutes 1 (moderate/formal) ________________________________

Day 3 Minutes 2 (vigorous / informal) ________________________________

Day 4 Minutes 1 (moderate/formal) ________________________________

Day 4 Minutes 2 (vigorous / informal) ________________________________

Day 5 Minutes 1 (moderate/formal) ________________________________

Day 5 Minutes 2 (vigorous / informal) ________________________________

Day 6 Minutes 1 (moderate/formal) ________________________________

Day 6 Minutes 2 (vigorous / informal) ________________________________

Day 7 Minutes 1 (moderate/formal) ________________________________

Day 7 Minutes 2 (vigorous / informal) ________________________________

Jr

PITTSBURGH SLEEP QUALITY INDEX

INSTRUCTIONS:

The following questions relate to your usual sleep habits during the past month only. Your answers should indicate the most accurate reply for the majority of days and nights in the past month.

Please answer all questions.

1 During the past month, what time have you usually gone to bed at night?

TIME

1 ○ AM
2 ○ PM

HOUR		MIN	
0	0	0	0
1	1	1	1
	2	2	2
	3	3	3
	4	4	4
	5	5	5
	6	6	6
	7	7	7
	8	8	8
	9	9	9

BED TIME

____

2 During the past month, how long (in minutes) has it usually taken you to fall asleep each night?

MIN	
0	0
1	1
2	2
3	3
4	4
5	5
6	6
7	7
8	8
9	9

NUMBER OF MINUTES

____

3 During the past month, what time have you usually gotten up in the morning?

TIME

1 ○ AM
2 ○ PM

HOUR		MIN	
0	0	0	0
1	1	1	1
	2	2	2
	3	3	3
	4	4	4
	5	5	5
	6	6	6
	7	7	7
	8	8	8
	9	9	9

GETTING UP TIME

____

4 During the past month, how many hours of actual sleep did you get at night? (This may be different than the number of hours you spent in bed.)

HOUR		MIN	
0	0	0	0
1	1	1	1
	2	2	2
	3	3	3
	4	4	4
	5	5	5
	6	6	6
	7	7	7
	8	8	8
	9	9	9

HOURS OF SLEEP PER NIGHT

____

For each of the remaining questions, check the one best response. Please answer all questions.

5 During the past month, how often have you had trouble sleeping because you . . .

	1 Not during the past month	2 Less than once a week	3 Once or twice a week	4 Three or more times a week
a) Cannot get to sleep within 30 minutes	○	○	○	○
b) Wake up in the middle of the night or early morning	○	○	○	○
c) Have to get up to use the bathroom	○	○	○	○
d) Cannot breathe comfortably	○	○	○	○
e) Cough or snore loudly	○	○	○	○
f) Feel too cold	○	○	○	○
g) Feel too hot	○	○	○	○
h) Had bad dreams	○	○	○	○
i) Have pain	○	○	○	○
j) Other reasons (describe below)	○	○	○	○
	0	1	2	3

Other reason(s)

____

____

PLEASE DO NOT WRITE IN THIS AREA

6 During the past month, how would you rate your sleep quality overall?

Very good	Fairly good	Fairly bad	Very bad
○	○	○	○

During the past month …	Not during the the past month	Less than once a week	Once or twice a week	Three or more times a week
7 How often have you taken medicine to help you sleep (prescribed or "over the counter")?	○	○	○	○
8 During the past month, how often have you had trouble staying awake while driving, eating meals, or engaging in social activity?	○	○	○	○

9 During the past month, how much of a problem has it been for you to keep up enough enthusiasm to get things done?

No problem at all	Only a very slight problem	Somewhat of a problem	A very big problem
○	○	○	○

© 1989, University of Pittsburgh. All rights reserved. Developed by Buysse,D.J., Reynolds,C.F., Monk,T.H., Berman,S.R., and Kupfer,D.J. of the University of Pittsburgh using National Institute of Mental Health Funding.
Buysse DJ, Reynolds CF, Monk TH, Berman SR, Kupfer DJ: Psychiatry Research, 28:193-213, 1989.

PLEASE DO NOT WRITE IN THIS AREA

PERCEIVED STRESS

INSTRUCTIONS:

The questions in this scale ask about your feelings and thoughts during the **last month**.

In each case, fill in the circle to indicate *how often* you felt or thought a certain way.

			0	1	2	3	4
			Never	Almost Never	Sometimes	Fairly Often	Very Often
	1	In the last month, how often have you been upset because of something that happened unexpectedly?	○	○	○	○	○
	2	In the last month, how often have you felt that you were unable to control the important things in your life?	○	○	○	○	○
	3	In the last month, how often have you felt nervous and "stressed"?	○	○	○	○	○
R	4	In the last month, how often have you felt confident about your ability to handle your personal problems?	○	○	○	○	○
R	5	In the last month, how often have you felt that things were going your way?	○	○	○	○	○
	6	In the last month, how often have you found that you could not cope with all the things that you had to do?	○	○	○	○	○
R	7	In the last month, how often have you been able to control irritations in your life?	○	○	○	○	○
R	8	In the last month, how often have you felt that you were on top of things?	○	○	○	○	○
	9	In the last month, how often have you been angered because of things that were outside of your control?	○	○	○	○	○
	10	In the last month, how often have you felt difficulties were piling up so high that you could not overcome them?	○	○	○	○	○

PLEASE DO NOT WRITE IN THIS AREA

Wisconsin Upper Respiratory Symptom Survey – 24

DAY 1 – Daily Symptom Report

DATE

MO.		DAY		YEAR	
0	0	0	0	0	0
1	1	1	1	1	1
	2	2	2	2	2
	3	3	3	3	3
	4		4	4	4
	5		5	5	5
	6		6	6	6
	7		7	7	7
	8		8	8	8
	9		9	9	9

TIME

○ AM
○ PM

HOUR		MIN	
0	0	0	0
1	1	1	1
	2	2	2
	3	3	3
	4	4	4
	5	5	5
	6	6	6
	7	7	7
	8	8	8
	9	9	9

0 Do you think that you are still sick with this respiratory infection? 1 ○ Yes 0 ○ No

Please fill in **one circle** for each of the following items:

	Not Sick	Very Mildly		Mildly		Moderately		Severely
	0	1	2	3	4	5	6	7
1 How sick do you feel **today**?	○	○	○	○	○	○	○	○

Please rate the **average severity** of your cold symptoms over the last 24 hours:

	Do not have this symptom	Very Mild		Mild		Moderate		Severe
	0	1	2	3	4	5	6	7
2 Runny nose	○	○	○	○	○	○	○	○
3 Plugged nose	○	○	○	○	○	○	○	○
4 Sneezing	○	○	○	○	○	○	○	○
5 Sore throat	○	○	○	○	○	○	○	○
6 Scratchy throat	○	○	○	○	○	○	○	○
7 Cough	○	○	○	○	○	○	○	○
8 Hoarseness	○	○	○	○	○	○	○	○
9 Head congestion	○	○	○	○	○	○	○	○
10 Chest congestion	○	○	○	○	○	○	○	○
11 Feeling tired	○	○	○	○	○	○	○	○
12 Headache	○	○	○	○	○	○	○	○
13 Body Aches	○	○	○	○	○	○	○	○
14 Fever	○	○	○	○	○	○	○	○

Over the last 24 hours, how much has your cold interfered with your ability to:

	Not at all	Very Mildly		Mildly		Moderately		Severely
	0	1	2	3	4	5	6	7
15 Think clearly	○	○	○	○	○	○	○	○
16 Sleep well	○	○	○	○	○	○	○	○
17 Breathe easily	○	○	○	○	○	○	○	○
18 Walk, climb stairs, exercise	○	○	○	○	○	○	○	○
19 Accomplish daily activities	○	○	○	○	○	○	○	○
20 Work outside the home	○	○	○	○	○	○	○	○
21 Work inside the home	○	○	○	○	○	○	○	○
22 Interact with others	○	○	○	○	○	○	○	○
23 Live your personal life	○	○	○	○	○	○	○	○

24 **Compared to yesterday**, I feel that my cold is ...

Very much better	Somewhat better	A little better	The same	A little worse	Somewhat worse	Very much worse
○	○	○	○	○	○	○
0	1	2	3	4	5	6

PLEASE DO NOT WRITE IN THIS AREA

Medication use during your cold or flu

During this cold/flu have you been PRESCRIBED antibiotics OR anti-viral medications by your doctor/ NP/ PA for your cold/flu? (i.e., for bronchitis, ear infection, influenza, sinus infection, strep throat, tonsillitis, viral pneumonia)

IF YES, what ANTIBIOTICS OR ANTI-VIRALS were you prescribed?

1 ○ Amoxicillin
2 ○ Augmentin
3 ○ Azithromycin (Z-pack)
4 ○ Ciprofloxacin
5 ○ Clarithromycin
6 ○ Clindamycin
7 ○ Doxycycline Hyclate
8 ○ Erythromycin
9 ○ Levofloxacilin (Levaquin)
10 ○ Moxifloxacin (Avelox)
11 ○ Oseltamavir (Tamiflu)
12 ○ Sulfamethoxazole-Trimethoprim
13 ○ Zanamivir (Relenza)
14 ○ ____________________
○ ____________________
○ ____________________

During this cold/flu have you taken any **over-the-counter medications** for your cold? Over-the-counter (OTC) drugs are medicines you can buy without a doctor's prescription.

We are only interested in OTC medications you took to help you deal with your cold symptoms. Examples to consider are pain relievers, decongestants, antihistamines, cough medicines, nasal sprays, multi-symptom cold medicines (regular and night-time), throat lozenges, or any products that you think may reduce the length or severity of your cold or affect your immune system. **Please do not include any drugs you take on a regular basis that are not related to a cold.**

IF YES, what medication(s) did you take?

1 ○ Acetaminophen (Tylenol)
2 ○ Acetaminophen PM (Tylenol PM)
3 ○ Anefrin (Afrin)
4 ○ Cold Relief Plus (Alka Seltzer Plus)
5 ○ Cough drops
6 ○ Daytime Cold and Flu (Dayquil)
7 ○ Emergen-C Vitamin C
8 ○ Ibuprofen (Advil/Motrin)
9 ○ Ibuprofen PM (Advil/Motrin)
10 ○ Mucus Relief (Mucinex)
11 ○ Mucus Relief D (Mucinex D)
12 ○ Mucus Relief PE (Mucinex PE)
13 ○ Naproxen Sodium (Aleve)
14 ○ Nighttime Cold and Flu (Nyquil)
15 ○ Wal-act (aka Actifed)
16 ○ Wal-born Plus (Airborne)
17 ○ Wal-dryl (Benadryl/diphenhydramine)
18 ○ Wal-fex (Allegra)
19 ○ Wal-finate (Alavert)
20 ○ Wal-flu (Theraflu)
21 ○ Wal-itin (Claritin/Loratidine)
22 ○ Wal-itin D (Claritin D)
23 ○ Wal-phed (Sudafed/pseudoephedrine)
24 ○ Wal-tussin (Robitussin)
25 ○ Wal-zyr (Zyrtec)
26 ○ ____________________
27 ○ ____________________
28 ○ ____________________

PLEASE DO NOT WRITE IN THIS AREA

ou

Your Health and Well-Being (SF12)

This survey asks for your views about your health.

This information will help keep track of how you feel and how well you are able to do your usual activities. *Thank you for completing this survey!*

For each of the following questions, please fill in the circle that best describes your answer.

1 In general, would you say your health is:

Excellent 1	Very good 2	Good 3	Fair 4	Poor 5
○	○	○	○	○

2 The following questions are about activities you might do during a typical day. Does **your health now limit you** in these activities? If so, how much?

	Yes, limited a lot 1	Yes, limited a little 2	No, not limited at all 3
a **Moderate activities**, such as moving a table, pushing a vacuum cleaner, bowling, or playing golf	○	○	○
b Climbing **several** flights of stairs	○	○	○

3 During the **past 4 weeks**, how much of the time have you had any of the following problems with your work or other regular daily activities **as a result of your physical health**?

	All of the time 1	Most of the time 2	Some of the time 3	A little of the time 4	None of the time 5
a **Accomplished less** than you would like	○	○	○	○	○
b Were limited in the **kind** of work or other activities	○	○	○	○	○

4 During the **past 4 weeks**, how much of the time have you had any of the following problems with your work or other regular daily activities **as a result of any emotional problems** (such as feeling depressed or anxious)?

	All of the time 1	Most of the time 2	Some of the time 3	A little of the time 4	None of the time 5
a **Accomplished less** than you would like	○	○	○	○	○
b Did work or other activities **less carefully than usual**	○	○	○	○	○

PLEASE DO NOT WRITE IN THIS AREA

5 During the **past 4 weeks**, how much did **pain** interfere with your normal work (including both work outside the home and housework)?

Not al all 1	A little bit 2	Moderately 3	Quite a bit 4	Extremely 5
○	○	○	○	○

6 These questions are about how you feel and how things have been with you **during the past 4 weeks.**

For each question, please give the one answer that comes closest to the way you have been feeling.

How much of the time during the **past 4 weeks...**

	All of the time 1	Most of the time 2	Some of the time 3	A little of the time 4	None of the time 5
a Have you felt calm and peaceful?	○	○	○	○	○
b Did you have a lot of energy?	○	○	○	○	○
c Have you felt downhearted and depressed?	○	○	○	○	○

7 During the **past 4 weeks**, how much of the time has your **physical health or emotional problems** interfered with your social activities (like visiting friends, relatives, etc.)?

All of the time 1	Most of the time 2	Some of the time 3	A little of the time 4	None of the time 5
○	○	○	○	○

Thank you for completing these questions!

SF-12v2™ Health Survey © 1994, 2002 by QualityMetric Incorporated and Medical Outcomes Trust. All Rights Reserved.
SF-12® a registered trademark of Medical Outcomes Trust.
(SF12v2 Standard, US Version 2.0)

PLEASE DO NOT WRITE IN THIS AREA

SOCIAL NETWORK INDEX

This questionnaire is concerned with how many people you see or talk to on a regular basis, including family, friends, workmates, neighbors, etc. Please read each question carefully and place a check next to the answer that best describes you. There are no right or wrong answers.

1 How would you describe your living situation?
1 O live alone
2 O live with my parents or other family members
3 O share with a roommate or live in dormitory setting
4 O live with spouse or significant other

2 Which best describes your **marital status**?
1 O single, never married or lived with someone in a marital-type relationship
2 O currently married or living with someone in a marital type relationship
3 O separated from my marital partner
4 O divorced, or formerly lived with someone in a marital-type relationship

3 Do you have any children? How many?

None 0	1 1	2 2	3 3	4 or more 4
O	O	O	O	O

If you have children answer all questions. *If not*, skip to 3c.

3a How many of your children live with you on at least 3 days per week on average?

None 0	1 1	2 2	3 or more 3
O	O	O	O

3b How many of your children do you see or talk to on the phone at least once every two weeks?

None 6	1 1	2 2	3 or more 3
O	O	O	O

3c How many children do you have in-person contact with in an average week? (Include your children, if applicable, **and** any other children, ages 0 to 17 years.)

None 0	1 to 5 1	6 to 19 2	20 or more 3
O	O	O	O

4 Are your parents living?

Both parents 1	Neither parent 2	Mother only 3	Father only 4
O	O	O	O

4a Do you see or talk to either of your parents at least once every 2 weeks?

Both parents 1	Neither parent 2	Mother only 3	Father only 4
O	O	O	O

5 If you are married, or in a marital type relationship, are **his/her parents** living?

Not applicable 1	Both parents 2	Neither parent 3	Mother only 4	Father only 5
○	○	○	○	○

5a Do you see or speak with **his/her parents** at least once every 2 weeks?

Both parents 1	Neither parent 2	Mother only 3	Father only 4
○	○	○	○

6 How many other relatives (other than your spouse, parents, or children) do you feel close to?

None 0	1 1	2 2	3 3	4 4	5 5	6 6	7 or more 7
○	○	○	○	○	○	○	○

6a How many of these relatives do you see or telephone at least once every 2 weeks?

None 0	1 1	2 2	3 3	4 4	5 5	6 6	7 or more 7
○	○	○	○	○	○	○	○

7 How many close friends do you have (meaning people you feel at ease with, can talk to about private matters, and can call for help)?

None 0	1 1	2 2	3 3	4 4	5 5	6 6	7 or more 7
○	○	○	○	○	○	○	○

7a How many of these friends do you see or talk to at least once every 2 weeks?

None 0	1 1	2 2	3 3	4 4	5 5	6 6	7 or more 7
○	○	○	○	○	○	○	○

8 Do you belong to a church, temple or other religious group?

○ NO 0 ○ YES 1

If YES,

8a During the last month how many times have you attended services?

None 0	1 1	2 2	3 3	4 4	5 5	6 6	7 or more 7
○	○	○	○	○	○	○	○

8b How many members of your church or religious group do you talk to at least once every 2 weeks (this includes at group meetings)?

None 0	1 1	2 2	3 3	4 4	5 5	6 6	7 or more 7
○	○	○	○	○	○	○	○

9 Do you belong to any other kinds of groups (e.g., social groups, unions, professional organizations, groups concerned with children [PTA, scouts], a charity or service group)?

○ NO ○ YES

0 1

If YES:

9a During the last month, how many times have you attended these group meetings or functions?

None	1	2	3	4	5	6	7 or more
O	O	O	O	O	O	O	O

9b How many members of the group(s) did you see or talk to at least once every 2 weeks (this includes at group meetings)?

None	1	2	3	4	5	6	7 or more
O	O	O	O	O	O	O	O

10 Are you currently employed, either full- or part-time?

O No O Yes, self-employed O Yes, by others

If YES:

10a If you supervise others, how many people do you manage?

None	1	2	3	4	5	6	7 or more
O	O	O	O	O	O	O	O

10b How many people at work (other than those you supervise) do you talk to at least once a week?

None	1	2	3	4	5	6	7 or more
O	O	O	O	O	O	O	O

11 Are you currently involved in volunteer work on a regular basis?

O NO O YES

If YES:

11a How many people involved in these volunteer projects do you talk to at least once a week?

None	1	2	3	4	5	6	7 or more
O	O	O	O	O	O	O	O

12 Do you attend classes (school, technical training, adult education) on a regular basis?

O NO O YES

If YES:

12a How many fellow students or teachers do you talk to at least once every 2 weeks (this would include at class meetings)?

None	1	2	3	4	5	6	7 or more
O	O	O	O	O	O	O	O

13 How many of your neighbors do you visit or talk to at least once every 2 weeks?

None	1	2	3	4	5	6	7 or more
O	O	O	O	O	O	O	O

Social Provisions Scale©

INSTRUCTIONS:

In answering the following questions, think about your current relationships with friends, family members, co-workers, community members, and so on.

Please indicate to what extent each statement describes your current relationships with other people.

		Strongly Disagree 1	Disability 2	Agree 3	Strongly Agree 4
1	There are people I can depend on to help me if I really need it.	○	○	○	○
2	I feel that I do not have close personal relationships with other people.	○	○	○	○
3	There is no one I can turn to for guidance in times of stress.	○	○	○	○
4	There are people who depend on me for help.	○	○	○	○
5	There are people who enjoy the same social activities I do.	○	○	○	○
6	Other people do not view me as competent.	○	○	○	○
7	I feel personally responsible for the well-being of another person.	○	○	○	○
8	I feel part of a group of people who share my attitudes and beliefs.	○	○	○	○
9	I do not think other people respect my skills and abilities.	○	○	○	○
10	If something went wrong, no one would come to my assistance.	○	○	○	○
11	I have close relationships that provide me with a sense of emotional security and well-being.	○	○	○	○
12	There is someone I could talk to about important decisions in my life.	○	○	○	○
13	I have relationships where my competence and skill are recognized.	○	○	○	○
14	There is no one who shares my interests and concerns.	○	○	○	○
15	There is no one who really relies on me for their well-being.	○	○	○	○
16	There is a trustworthy person I could turn to for advice if I were having problems.	○	○	○	○
17	I feel a strong emotional bond with at least one other person.	○	○	○	○
18	There is no one I can depend on for aid if I really need it.	○	○	○	○
19	There is no one I feel comfortable talking about problems with.	○	○	○	○
20	There are people who admire my talents and abilities.	○	○	○	○

PLEASE DO NOT WRITE IN THIS AREA

Social Provisions Scale, continued

		Strongly Disagree 1	Disability 2	Agree 3	Strongly Agree 4
21	I lack a feeling of intimacy with another person.	○	○	○	○
22	There is no one who likes to do the things I do.	○	○	○	○
23	There are people who I can count on in an emergency.	○	○	○	○
24	No one needs me to care for them.	○	○	○	○

PLEASE DO NOT WRITE IN THIS AREA

ATTENTION TO WORK

INSTRUCTIONS

Please describe your work experience in the **past month**.

This experience may be affected by many environmental as well as personal factors and may change from time to time.

For each of the following statements, please fill in the circle which best describes your work experience. Your answers should reflect an average across the past month.

If you are primarily a student or homemaker, please consider that your work.

		1 Strongly Disagree	2 Somewhat Disagree	3 Uncertain whether I Disagree or Agree	4 Somewhat Agree	5 Strongly Agree
1	The stresses of my job were hard to handle.	○	○	○	○	○
2	I was able to finish hard tasks in my work.	○	○	○	○	○
3	I was unable to take satisfaction in my work.	○	○	○	○	○
4	I felt hopeless about finishing my work.	○	○	○	○	○
5	I was able to focus on achieving my work goals.	○	○	○	○	○
6	I felt energetic enough to complete all my work.	○	○	○	○	○

1 ○ Not applicable *(indicate reason not applicable below)*

1 ○ Unemployed 2 ○ Other ______________________

They Don't need to enter this!

Alcohol and Tobacco Use Report Form (Timeline Followback)

ALCOHOL

Please use the calendar to write down how many drinks (standard drink) you had on each day during this two-week period.

1 Standard Drink

One 12 oz can/bottle of beer	One 5 oz glass of regular (12%) wine	1 ½ oz of hard liquor (e.g. rum, vodka, whiskey)	1 mixed or straight straight drink with 1 ½ oz hard liquor

TOBACCO

Please use the calendar to write down how much tobacco you used on each day during this two-week period. **If the tobacco is not in the form of cigarettes**, please check off one of the following:

1 ○ Chew 2 ○ Pipe 3 ○ Cigar 4 ○ Other: ____________________

Start Date (Day 1): _____/_____/_____ **End Date** (yesterday): _____/_____/_____

1 ○ **NONE, I have not used ALCOHOL or TOBACCO in the past 14 days**

Day 1 (14 days ago)	Day 2	Day 3	Day 4	Day 5	Day 6	Day 7
Date:	Date:	Date:	Date:	Date:	Date:	Date:
# of drinks:	# of drinks:	# of drinks:	# of drinks:	# of drinks:	# of drinks:	# of drinks:
# of cigarettes or other tobacco product:	# of cigarettes or other tobacco product:	# of cigarettes or other tobacco product:	# of cigarettes or other tobacco product:	# of cigarettes or other tobacco product:	# of cigarettes or other tobacco product:	# of cigarettes or other tobacco product:

Day 8	Day 9	Day 10	Day 11	Day 12	Day 13	Day 14 (yesterday)
Date:	Date:	Date:	Date:	Date:	Date:	Date:
# of drinks:	# of drinks:	# of drinks:	# of drinks:	# of drinks:	# of drinks:	# of drinks:
# of cigarettes or other tobacco product:	# of cigarettes or other tobacco product:	# of cigarettes or other tobacco product:	# of cigarettes or other tobacco product:	# of cigarettes or other tobacco product:	# of cigarettes or other tobacco product:	# of cigarettes or other tobacco product:

This page will not be scanned (we will rip it

PLEASE DO NOT WRITE IN THIS AREA

088

Appendix D: Lab Protocols

IL-6 ELISA SOP/IL-8 ELISA SOP/ IP-10 ELISA SOP

(Mary Hayney updated 03/14/2012)

OptEIA Sets (BD Biosciences)

1) Dilute Capture Antibody in Coating Buffer according to lot specific instructions.

2) Add 100 µL of Capture Antibody dilution to appropriate wells.

3) Seal plate and incubate overnight at 4˚C.

4) Set out all reagents and allow to reach room temperature (RT) before use. Thaw samples if necessary.

5) Prepare Wash Buffer then rinse plate using the "Wash 3" program. Bang dry plate on absorbent paper to remove residual buffer.

6) Add 200 µL of Assay Diluent and apply an adhesive strip over the plate. Incubate at RT for 1 hr.

7) Repeat wash procedure from step 5.

8) Prepare Standard serial dilutions:
   a) Prepare a 200 pg/mL standard from the stock standard.
   b) Pipette 100 µL of Assay Diluent into wells B1/B2 – H1/H2.
   c) Pipette 100 µL of 200pg/mL standard into wells A1/A2 and B1/B2.
   d) Mix solution in wells B1/B2 by drawing and expelling with the pipette 10X.
   e) Draw up 100 µL from wells B1/B2 and add to next two wells mixing as before.
   f) Continue serial dilutions until wells G1/G2. After mixing, draw up 100 µL from both wells and discard.
   g) Assay Diluent in wells H1/H2 serves as the zero standard.

9) Add 100 µL of sample or control to assigned wells and apply an adhesive strip over the plate. Incubate at RT for 2 hr.

10) Rinse plate using the "Wash 5" program and bang dry plate.

11) Add 100 µL of prepared Working Detector to each well and apply an adhesive strip over the plate. Incubate at RT for 1hr.

12) Rinse plate using the "Wash 7" program and bang dry plate.

13) Add 100 µL of Substrate Solution to each well. Incubate at RT for 30 min. in the dark with no seal.

14) Add 50 µL of Stop Solution to each well. Read within 30 min. at 450 nm and a λ correction at 570. In the Soft Max software select option "Endpoint L1-L2." Select 450 as L1 and 570 as L2. On the plate reader, select 450 as λ1 and 570 as λ2. Read plate and export data to Excel worksheet.

Viral Identification

Identification of viral etiological agents will be carried out in UW Pediatrics Viral Detection Laboratory, where a high-throughput multiplex PCR-based respiratory viral detection assay has been developed.

Viral Identification

Consultant James Gern MD has access to an accurate and efficient system to identify viral etiological agents most commonly implicated in acute respiratory infection.

Purpose - To better understand the contribution of specific respiratory viruses to common cold illnesses, and to determine whether treatments in the protocol improve outcomes of specific viral infections, a practical and accurate assay is needed for detecting hundreds of virus strains in thousands of samples that are anticipated in this study.

Methods - To accomplish this goal, UW Pediatrics Viral Detection Laboratory implemented a high throughput (multiplexed, automated, 96-well format, 3 hr completion time) platform technology (PLx) that utilized a novel PCR chemistry (MultiCode® PLx, EraGen Biosciences, Middleton, WI). PLx simplifies molecular detection through the use of an expanded genetic alphabet (isoguanosine and isocytidine), allowing reaction products to be labeled and captured without many of the steps needed for standard protocols (sample clean-up, washing, transfer).

To implement the Multicode assay, all respiratory viral sequences in GenBank (> 2 million bases) were identified and analyzed, and the results were used to design Multicode primers for the simultaneous detection of all respiratory viruses. Hundreds of candidate primers were made and then tested against a panel of 140 cloned viral target cDNAs at 20 copies per reaction for the best specificity and sensitivity. This work yielded 19 primer sets for all target viruses. Each primer set was specific for the intended target and highly sensitive, detecting 20 copies of target cDNA or less, with a typical signal/noise ratio of 10 to 50.
The performance and practicality of this Respiratory Multicode Assay (RMA) has been assessed by analyzing two sets of specimens. The first set consisted of 105 clinical specimens that were positive for HRV, RSV, Flu, PIV or Ad by traditional techniques. By RMA, all target viruses were detected with an overall rate of 91.4%. The second set of specimens consisted of 103 nasal mucus samples from 5-year old children with asthma and respiratory symptoms. The RMA detected viruses in 76 specimens (73.8%) compared to only 24 (23.3%) by traditional techniques. HRVs were the most frequent viruses detected (40/76). These results show that this high throughput and comprehensive PLx-based assay will improve the practicality and accuracy of detecting respiratory viruses in large epidemiologic studies.
After its completion, RMA has been used for successfully analyzing ~30000 nasal samples (references are listed below).

1. Lee, W. M., C. Kiesner, T. Pappas, I. Lee, K. Grindle, T. Jartti, B. Jakiela, R. F. Lemanske, Jr., P. A. Shult, and J. E. Gern. 2007. A diverse group of previously unrecognized human rhinoviruses are common causes of respiratory illnesses in infants. PLoS One 2:e966.

2. Jartti, T., W. M. Lee, T. Pappas, M. Evans, R. F. Lemanske, Jr., and J. E. Gern. 2008. Serial viral infections in infants with recurrent respiratory illnesses. Eur Respir J 32:314-20.

3. Matthew, J., L. M. Pinto Pereira, T. E. Pappas, C. A. Swenson, K. A. Grindle, K. A. Roberg, R. F. Lemanske, W. M. Lee, and J. E. Gern. 2009. Distribution and seasonality of rhinovirus and other respiratory viruses in a cross-section of asthmatic children in Trinidad, West Indies. Ital J Pediatr 35:16.

4. Bizzintino, J., W.-M. Lee, I. A. Laing, F. Vang, T. Pappas, G. Zhang, A. C. Martin, G. C. Geelhoed, P. C. McMinn, J. Goldblatt, J. E. Gern, and P. N. Le Souëf. 2010. Association between human rhinovirus C and severity of acute asthma in children. European Respiratory Journal In press.

5. Denlinger, L. C., R. L. Sorkness, W.-M. Lee, M. D. Evans, M. Wolff, S. Mathur, C. Swenson, G. Crisafi, K. Gaworski, T. E. Pappas, R. Vrtis, E. A. Kelly , J. E. Gern, and N. N. Jarjour. 2010. Lower Airway Rhinovirus Burden and the Risk of Asthma Exacerbation. J Allergy Clin Immunol Submitted.

6. Kloepfer, K. M., W.-M. Lee, J. P. Olenec, T. E. Pappas, G. Liu, R. Vrtis, and J. E. Gern. 2010. Increased H1N1 Infection Rate in Asthmatic Children. In preparation.

C-Reactive protein (CRP), Procalcitonin (PCT)
(will be added)

Neutrophil counts

<u>Procedure for Neutrophil Count in Nasal Wash</u>

<u>Equipment and Reagents</u>

1. C-Chip™ DHC-N01 plastic disposable Neubauer hemocytometer (inCyto / Digital Biotechnology)
2. Wet house enclosure - metal cover with filter paper inside that can be moistened for humidity control.
3. Brightfield microscope
4. Mechanical cell counter or automated cell analyzer.
5. 12x75mm test tubes and Pipetman pipettes
6. 0.1% toluidine blue stain from Newcomer Supply

Procedure

1. Vortex the sample.
2. Using a Pipetman, transfer 50 uL to a pourover tube.
3. Add 50 uL 0.1% toluidine blue stain. Let sit 15-20 min.
4. Vortex the stained dilution of the sample.
5. Using an InCyto Neubauer hemocytometer, place one of the small tear-off tabs from the bar code label on one side or the other of the chamber for identification (as shown in picture below).
6. Use a capillary tube or pipette to plate or *charge* well-mixed specimen on both sides of the hemocytometer. See arrows on figure below. Be careful not to over- or undercharge the chamber or introduce air bubbles.
7. Allow the plated hemocytometer to sit under a *wet house* for a few minutes for cells to settle.

> *TIP: When a sample—especially one with high cellularity—sits in the chamber too long, cells tend to start lining up on the grid lines and may affect accuracy of count. Try a larger dilution and replate.*

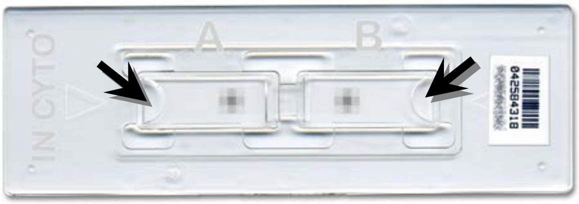

InCyto plastic disposable hemocytometer with built in fixed cover glass. Arrows indicate charging wells.

8. Focus counting grid under low power (10×), and then using high dry objective (45× or 20×), count and differentiate the WBCs only.
9. Count the neutrophils only. If the cell has two evenly sized lobes resembling an eosinophil, is mononuclear or has disintegrated do NOT count in with the neutrophils.

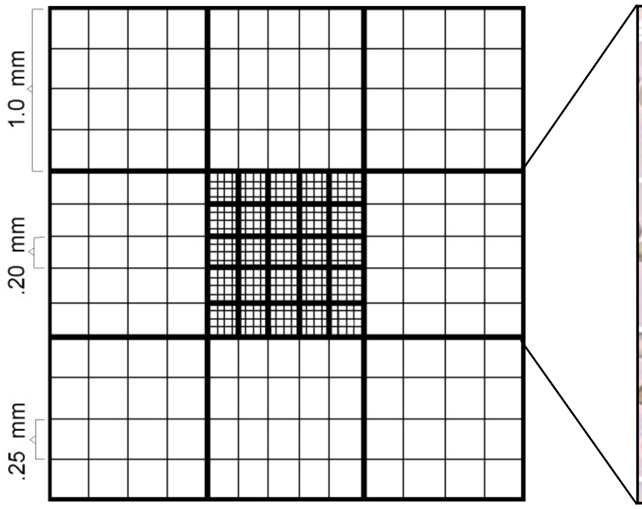

Counting surface depth = 0.1 mm
Total volume per side = 0.9 µL
(9 sq.mm. x 0.1 mm)

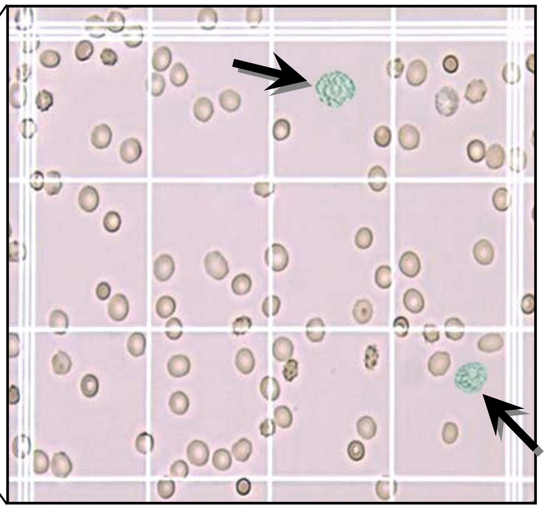

Brightfield look at portion of counting grid with cells present. Arrows point at two white blood cells; the rest are red blood cells.

10. Examine hemocytometer grid under low power (10×) to determine a practical area to count.
11. If cells are overlapping, make a dilution using Sysmex Cell-Pack® or isotonic saline. 1:5, 1:10, or 1:20 are convenient dilutions to make using a Pipetman or other device**.** Note any additional dilutions made on your worksheet.

TIP: Phase-Contrast microscope may be employed when red cells difficult to discern from white cells. Under phase lighti RBCs look smooth, dim, with a dark outer rim bordered b light halo; whereas, WBCs look grainy and often have overall bright appearance and halo, and are larger.

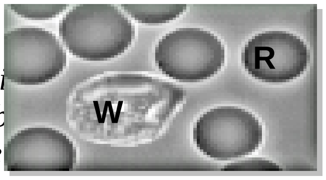

Precision or side-to-side agreement should fall within the ranges given in the table below for either nucleated cell. If agreement fails: 1) count additional area and reassess; 2) plate a new chamber and recount; 3) make a new dilution and start over.

**Hemocytometer Side-to-Side Agreement**			
**# Cells Counted** (raw count over any area)	**Agreement**	**# Cells Counted** (raw count over any area)	**Agreement**
1-20	± 40%	101-1000	± 10%
21-100	± 20%	>1000 *	± 5%
* If the number of cells on a side is >1000 the sample should be diluted and			

12. Use Result Entry or the following formula to calculate neutrophil concentrations:

$$\textbf{Cells / µL} = \frac{\textbf{\# Cells Counted} \times \textbf{Dilution}}{\textbf{Area Counted} \times \textbf{Depth (0.1mm)}}$$

The *Result Entry* computer program has a built in calculator that can be used when entering results, but it has limitations (e.g., it does not allow separate dilutions, for RBCs and WBCs, which may be necessary in some cases to achieve more accurate results).

Analyte	Result
DIAG	
CCDIL	2
**SUPCLR**	**\NDONE**
NCC2A	20
NCC2B	26
NCC2SQ	1
**NCC2**	230
RBC2A	200
RBC2B	180
RBC2SQ	0.2
**RBC2**	9500

NCC2 and RBC2 are automatically calculated based on the data you input into the fields opposite analytes highlighted in yellow. To bypass the automated calculation, leave the yellow fields blank and input your manual calculation directly into the NCC2 and RBC2 result fields. Yellow items are non-reportable.

13. After the calculation, delete numbers in yellow fields. Using Result Entry, enter SEECOM for color and clarity and NDONE for all analytes except RMKS.
14. **Using the dilution factor of 2**, report the # of neutrophils in the RMKS.
    Ex: 23/uL Neutrophils
15. Preview Results and if ok, Release All.

Appendix F: Data and Safety Monitoring Plan

CRU DSMP Sheet
(uploaded into ARROW application with CRU Application)

NCCAM DSMP

Data and Safety Monitoring Plan

As submitted to UW IRB HSC March 21, 2012

I. Study Identification Information

A. Study Title: Meditation or Exercise for Preventing Acute Respiratory Infection (MEPARI-2)

B. Name of Principal Investigator: Bruce Barrett MD PhD

II. Study Overview

A. Brief Description of the Purpose of the Study: The primary goal of this project is to determine whether behavioral training in mindfulness meditation or moderate intensity sustained exercise can lead to reductions in acute respiratory infection (ARI) illness, such as common cold and influenza. Our preliminary findings suggest substantial benefit of these interventions in terms of reduced incidence, duration and severity of ARI illness, with corresponding reductions in days of work lost to illness. If the proposed research confirms these findings, there may be major implications for public and private health-related policy and practice, as well as for scientific knowledge regarding health maintenance and disease prevention.

B. Adherence Statement: The Data and Safety Monitoring Plan outlined below will adhere to the protocol approved by the University of Wisconsin – Madison Health Sciences Institutional Review Board (UW HS-IRB) and the Clinical Research Unit (CRU - formerly Clinical and Translational Research Core) Review Committee.

III. Confidentiality

A. Protection of Subject Privacy: Screening of potential participants and consent and enrolling of participants will be carried out privately, via telephone or in-person, in a private location. Similarly, collection of biological samples (blood draw and nasal wash) will be done with full privacy. Nasal self-swabs will be collected in the privacy of each participant's home. The exercise and meditation interventions, however, will be done in group session, hence should not be considered private. Potential participants will be informed of these aspects of the study.

All personal identifiers will be destroyed once data collection and analysis have been completed. For those who formally agree that we may keep their name and contact information for potential future research opportunities, the code list linking individual to study data will be destroyed.

B. Database Protection: All project data will be kept in password-protected security-ensured databases. Project personnel will be required to enter separate personal logins and passwords both for computer workstations specifically designated for the project and for the database user authentication process. Personally identifiable information (name, address, date of birth, adverse effect reports, etc.) required for longitudinal study participant management will be stored in an encrypted database, on a local hard drive physically separate from the database storing research outcome data. Permission to view study participant information will be available only to the Principal Investigator or his designee, as regulated by the user authentication procedure. Data analysts will be restricted to viewing outcome data, and will not be able to access personal identifiers. The database used to perform confidential study participant tracking and reimbursement auditing functions will be accessible only to the PI and to the involved research specialists, following UW HS-IRB requirements.

Identifying information will be recorded either on a single detachable sheet of paper, kept in a locked filing cabinet, or in a separate password-protected security database, which will not have direct links to process or outcome data. Project databases will generate an unseen unique identifying code for each participant, separate from the study participant number used on data collection instruments, to connect the participant's multiple data elements. Association of that code with the study participant's identifying information will reside in an encrypted database separate from the study's research data, ensuring that no records in the dataset used for analysis will contain any data that could identify the participant. All computerized databases will be protected with passwords, with access restricted to appropriate study personnel.

C. Confidentiality During AE Reporting: AE reports and annual summaries will not include subject-identifiable material. Each report will have only the study identifier associated with that participant.

IV. Adverse Event Information

A. Definition: An adverse event (AE) is any untoward medical occurrence in a subject temporally associated with participation in the clinical study or with use of the experimental agent being studied. An adverse finding can include a sign, symptom, abnormal assessment (laboratory test value, vital signs, electrocardiogram finding, etc.) or any combination of these.
A Serious Adverse Event (SAE) is any adverse event that results in one or more of the following outcomes:

- Death
- A life-threatening event
- Inpatient hospitalization or prolongation of existing hospitalization
- A persistent or significant disability/incapacity
- A congenital anomaly or birth defect
- Important medical event based upon appropriate medical judgment

B. Classification of AE Severity: AEs will be labeled according to severity which is based on their impact on the patient. An AE will be termed ‘mild’ if it does not have a major impact on the patient, ‘moderate’ if it causes the patient some minor inconvenience and ‘severe’ if it causes a substantial disruption to the patient’s well being. However, due to the nature of this research study there are no “expected” adverse events.

C. AE Attribution Scale: AEs will be categorized according to the likelihood that they are related to the study intervention. Specifically, they will be labeled either definitely, probably, possibly or unrelated to the study intervention.

D. Expected Risks: Due to the nature of this study there are no “expected” adverse events or known risks.

E. SAE Reporting: SAEs that are unanticipated, serious, and/or possibly related to the study intervention will be reported to the Data and Safety Monitoring Committee (DSMC), HS-IRB, CRU, and NCCAM in accordance with requirements. Anticipated SAEs or those unrelated to the study intervention will be reported to the same individuals/entities in accordance with requirements.

V. Data Quality and Safety Review Plan and Monitoring

A. Data Quality and Management

1) Description of Plan for Data Quality and Management: The study team will review all data collected on an ongoing basis for data completeness and accuracy as well as for protocol compliance. The procedures by which data were collected and verified during the preliminary trial described above were strong, but will be improved further for the proposed research. Specifically, we will develop a computer-assisted weekly monitoring system to improve identification, verification and monitoring of ARI illness. This security-protected web-based data collection system will be developed and overseen by UW Department of Family Medicine (DFM) programmer, Don Thomson, and administered by a DFM data manager to be hired and trained. Real-time inspection of incoming data will allow early identification and correction of any potential errors. Blinding of the person implementing this system to experimental group will reduce potential bias arising from self-report. Implication of strict criteria for defining the beginning and end of each ARI illness episode will further strengthen data integrity. Data that is not directly entered by participants (eg. monthly self-report questionnaires) will be scanned directly into a comma delimited database, with resulting electronic data compared to data on paper by hand. For data that is entered by project personnel, verification will be performed by someone other than the individual originally collecting the data, or by double-data entry. These procedures will facilitate verification of all primary and secondary endpoint data against original source documents. Statistical analysis will not be carried out until all data is complete.

B. Frequency of Data Review for this Study: Study progress and safety will be reviewed during bi-weekly co-investigator meetings (more frequently if needed). Progress reports, including patient recruitment, retention/attrition, and AEs will be provided to the Data Safety and Monitoring Committee at the end of each 8-week intervention, and then again at the conclusion of each yearly cohort.

C. Subject Accrual and Compliance

1) Measurement and reporting of subject accrual, adherence to inclusion/ exclusion criteria: Review of the rate of subject accrual, adherence to inclusion/exclusion criteria will occur after each cohort is enrolled.

2) Measurement and reporting of participant compliance to treatment protocol: Each participant will be assigned a study representative who will serve as his/her primary contact, to build a rapport and enhance protocol adherence. The primary contact cannot feasibly be blinded to allocation, which could potentially lead to bias. Thus, we will employ blinded-to-allocation personnel to assist participants with documenting and classifying ARI illness, sick days, and clinic visits. For most participants, weekly computer-assisted self-reports will be used to assess ARI illness. For those without internet access, weekly telephone calls will be scheduled. As soon as an ARI illness episode is verified by criteria, the participant will self-administer a nasal swab, and arrange a clinic visit where nasal wash will be obtained. During ARI episodes, participants will fill out respiratory infection daily logs, including both WURSS-24 self-assessments and questions documenting health care utilization and days lost to work.

   Adherence to meditation protocol during the 8-week program will be assessed by attendance records, teacher ratings of class participation, and daily practice logs. The meditation and exercise surveys will serve as additional intervention checks in that scores are expected to change more among those randomized to these interventions than to control groups. Continued adherence to meditation practice after the 8-week session will be assessed during bi-weekly phone contact during the 6-month observation period. Integrity and consistency of both the meditation and exercise behavioral training sessions will be assured by the use of a structured training manual and by employment of the same instructors throughout the project.

D. Designation of an Independent Monitoring Committee: A U.W.-based Data and Safety Monitoring Committee will be implemented upon funding, with commitment from three previous members already obtained. They include four UW faculty: Paul Hutson, PhD (Pharmacy), Nasia Safdar, MD (Infectious Disease), Tom Cook, PhD (Biostatistics) and Margo Hoover-Regan MD (pediatric oncology, clinical trials). Each member is independent of the principal investigator and co-investigators. Commencement of the DSMC and approval by both our IRB and NCCAM will occur after notification of funding and prior to the accrual of any research participants.

E. Safety Review Plan: Study progress and safety will be reviewed during bi-weekly co-investigator meetings (more frequently if needed). Progress reports, including patient recruitment, retention/attrition, and AEs will be provided to the Data Safety and Monitoring Committee at the end of each 8-week intervention and then again at the conclusion of each yearly cohort. While there are no “expected” adverse events in this trial, any “unexpected” adverse events will be monitored in a timely fashion, and reported promptly to appropriate people.

VI. Informed Consent

Written informed consent will be obtained from each participant at entry into the study. Informed consent is obtained by the following process:

- The participant will be asked to review the study consent form.
- The project coordinator will meet with the participant to review the form, to confirm the participant’s understanding of the study, and to answer any questions that the person might have.
- Once the participant demonstrates understanding of the study and agrees to participate in the study, the consent will be signed in the presence of the study coordinator.
- As outlined on the consent form, all participants will be free to withdraw consent at any time.

.

Appendix E: Study Procedures

Schedule of Assessments

Questionnaire Cover Sheets

Study Visit Checklists

Telephone Screening Card

Physician's Orders

Nasal Lavage Information Sheet

Nasal Wash Lab Work Information Sheet

PRC Protocol Summary

CRU Application

Information Summary Sheet

Informed Consent Form

Study Visit Checklists (these will be developed specific to each visit, below is an example)

MEPARI-2 Study Visit Checklist

BASELINE/MAIN STUDY VISIT

☐ REVIEW HOMEWORK/ SURVEYS
- Confirm subject ID on surveys
- PHQ-9 Score (ineligible if >14)

☐ BLOOD DRAW, NASAL WASH & VITAL SIGNS

☐ CONFIRM ELIGIBILITY FOR MAIN STUDY
- if ineligible pay $50 **RECORD CHECK #** __________

☐ INFORMED CONSENT (if eligible)
- Copy of informed consent provided to subject

☐ QUESTIONNAIRES
- **Staff Administered**: TLFB, GPAQ
- **Self-Administered**: SIC, SF-12, PSQI, MAAS

☐ RANDOMIZATION: ____________& PARTICIPANT NUMBER: _______________
- Record randomized class type and assigned class timeslot in folder ____ and on master clipboard

☐ PROVIDE INSTRUCTIONS TO UW HEALTH RESEARCH PARK (if applicable)
- Provide randomized class schedule/map, highlight assigned class timeslot

☐ THINKING AHEAD (POST) QUESTIONNAIRE (ALL GROUPS)
- Make sure you fill out the right questionnaire according to their randomized group

☐ PROVIDE **WURSS COLD SURVEYS** FOLDER & INSTRUCTIONS

☐ PROVIDE VISUAL AND VERBAL INSTRUCTIONS FOR NASAL SELF-SWAB
- Provide take home collection kits

☐ PROVIDE COLD **STUDY DATES TO REMEMBER** SCHEDULE

☐ PAY $50 FOR COMPLETING MAIN STUDY VISIT **RECORD CHECK #** __________

☐ SCHEDULE FLU SHOT/ WEEK 9 FU VISITS (provide appointment card)

FLU SHOT/ WEEK 9 Appt Time/Date: __________________

☐ Appointment Entered in Database ______ (initials)

____
Signature of person completing visit

____
Date

Questionnaire Cover Sheets

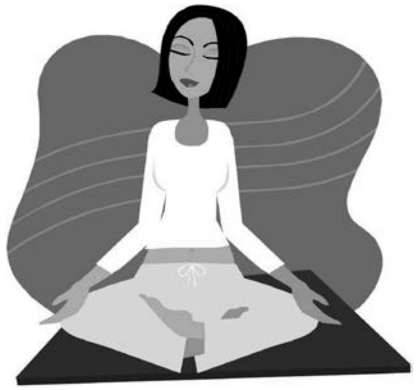

Meditation or Exercise for Preventing Acute Respiratory Infection (MEPARI-2) Research Study

Department of Family Medicine
University of Wisconsin – Madison

Study Instruments

STUDY VISIT: ☐ Run-in ☐ Baseline ☐ Week 9 ☐ 1 mo post
☐ 4 mo post ☐ Study Exit

TODAY'S DATE: |___|___| / |___|___| / |___|___|___|___|
MONTH DAY YEAR

PARTICIPANT ID: ___________________________________

This packet will take approximately 30 minutes for you to complete. Please take your time answering these questions and provide a response to ALL questions. In order for your answers to be most helpful to us, we need you to complete the entire packet. It is important that you try to be as honest and accurate as you can.

The answers you give us will be kept confidential. The results will be used for study purposes only and your name will never be connected to the results.

Please ask your study coordinator if you have any questions.

THANK YOU!

Study Personnel ONLY

Staff Initials: ___________

Date Reviewed: ___________

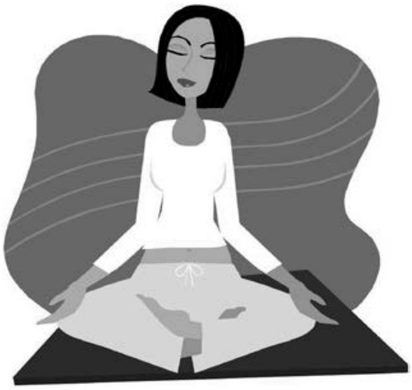

Meditation and Exercise for Preventing Acute Respiratory Infection (MEPARI-2) Research Study

Department of Family Medicine
University of Wisconsin – Madison

MONTHLY QUESTIONNAIRES

MONTH (alpha i.e. JAN for January): | ___ | ___ |___ |

YEAR: | ___ | ___ | ___ | ___ |

TODAY'S DATE: | ___ | ___ | / | ___ | ___ | / | ___ | ___ | ___ | ___ |
MONTH DAY YEAR

PARTICIPANT ID: ___________________________________

This packet will take approximately 30 minutes for you to complete. Please take your time answering these questions and provide a response to ALL questions. In order for your answers to be most helpful to us, we need you to complete the entire packet. It is important that you try to be as honest and accurate as you can.

The answers you give us will be kept confidential. The results will be used for study purposes only and your name will never be connected to the results.

Please ask your study coordinator if you have any questions.

THANK YOU!

Study Personnel ONLY

Staff Initials: __________

Date Reviewed: ___________

Nasal Lavage Information Sheet

Five cc/nostril of warmed lavage fluid is delivered via pipette sequentially to each nostril while the head is tipped back. The fluid is then expelled into a sterile dish when the head is tipped forward. With the collection dish still in place, compression is applied to one and then the other nostril while the subject blows the opposite nostril free of mucous.

Procedure:

1) Pre-warm 10 ml of Hank's Solution (w/o phenol red) Nasal Wash solution.
2) Provide with tissues and place a dental bib on the Subject. Put on gloves and mask (mask is optional).
3) Prior to each Subject's first lavage, describe the procedure so the Subject is familiar with the entire sequence and can anticipate each next step.
4) Have the Subject hold a sterile petri dish in his/her dominant hand and instruct that the plate be held from the bottom to allow easy removal of the lid.
5) Instruct Subject to tilt-back the head sufficiently to retain the instilled lavage fluid in the nares. Suggest a 20-second trial practice of breath-holding while doing a guttural stop initiated by making a "K" sound while pressing the tongue to the roof of soft palate.
6) While the head is tipped back, use the pipette aid to dispense 5 ml of solution into each nostril.
7) Remove the cover of the petri dish and instruct Subject to bring the dish up to just below the nose and to then come forward with the head, allowing the fluid to drain into the petri dish. Take care not to allow the petri dish to come in contact with the mouth or saliva.
8) Assist Subject by applying gentle pressure to one nostril at a time, allowing blowing and expelling of any remaining fluid from the nasal cavity.
9) Lightly scrape the edge of nose with the petri dish to remove mucus. If fluid is excessively mucoid, you may need to suction the specimen from the petri dish, directly under the nose, while the Subject holds the dish. This allows more complete recovery of any nasal mucus.
10) Maintaining sterile technique, the specimen is pipetted into a 10 ml transport tube and capped tightly. The recovered volume and the nasal mucus index is noted and recorded on the symptom sheet.
11) This tube is then taken to the Lab for processing.

Appendix G: Recruitment Materials

(uploaded into ARROW application)

Appendix H: Statistical Analysis (MEPARI-2)

March 30, 2012

(1) Zero Inflated Regression Model (Censored Inflated Regression Model)

The design of this study provides ample opportunity for subjects to report - or not report - acute respiratory infections (ARI). Individual ARI episodes can exist (incidence) or not exist, a logistic or binary measurement, and can come in a variety of severities and magnitudes (a continuous or linear measurement). The proportion of people who do not experience ARI (zero measurements) is expected to be between 25% and 75% of the sample. Modeling such data while ignoring the censoring, zero-inflation, and overdispersion may result in biased parameter estimates. According to Lambert (1992), the most common approach to modeling such distributions, is to assume a logistic regression model for the "zero, non-zero" values of the outcome and either a Poisson or Censored distribution for the model. Because the zeros are accounted for the logistic portion of the model, the counts or values portion can reflect, more accurately, the non-zero distribution. We proposed a censored inflated regression model, where two equations are estimated, one binary (no occurrence of a cold) and one for the continuous measures of severity and duration. The continuous model is for patients who are above the censoring point, in our case zero. The binary is for those patients above versus below the censoring point, and is a logistic regression model (i.e., it predicts those above versus below the censoring point). Mplus Version 6.12 (Muthen and Muthen, 2011) will be used to construct our censored-inflated normal models. Details regarding various zero inflated models (ZIM) may be accessed in Ridout, Demetrio, and Hinde (1998), Prasad (2009), and Lachenbruch (2002).

References

Lachenbruch, P A., (2002). Analysis of data with excess zeros. *Statistical Methods in Medical Research*, 11, 297-302.
Lambert, D. (1992). Zero-inflated Poisson regression, with an application to defects in manufacturing. *Technometrics*, 34, 1-14.
Ridout, M.S., Demetrio, C.G.B. and Hinde, J.P. (1998) Models for counts data with many zeros. *Proceedings of the XIXth International Biometric Conference*, Cape Town, Invited Papers, pp. 179-192.
Prasad, J, P. (2009*). Zero-inflated censored regression models: An application with episode of care data*. Brigham Young University, Department of Statistics.
Muthen, L. K., and Muthen, B. O. (1998-2011). Mplus user’s guide. Third Edition. Los Angeles, CA: Muthen and Muthen.

(2) Potential Indirect Effects (Mediation)

Mediational analysis is a method that can help researchers understand the mechanisms underlying the phenomena they study. The basic mediational framework involves a three variable system in which an initial independent variable affects a mediational variable, which, in turn, affects an outcome variable (Baron & Kenny, 1986). The aim of mediational analysis is to determine whether the relation between the treatment conditions and the outcome variables is due, wholly or in part, to mediator conditions. While the Baron and Kenny (1986) approach (BK) is somewhat straightforward, it is complicated by the inclusion of longitudinal models. To address this challenge, we propose remodeling our longitudinal models as hierarchical models, with the repeated measures nested under each subject. Krull and MacKinnon (2001) have recently demonstrated an expansion of the Baron-Kenny approach to encompass hierarchical models. The BK approach may be incorporated into our proposed secondary analysis modeling strategy for assessing longitudinal data.

To demonstrate the BK approach for a repeated measures model, we demonstrate the point estimate of the mediated effect first for single-level data, which requires the estimation of a regression equation predicting the outcome measure (Y_i) from the initial variable (X_i)

$$(1)\quad Y_i = \beta_0 + \beta_c X_i + r_i$$

and the estimation of a regression equation predicting the outcome measure from the initial variable and the mediator (*Mi*) $Y_i = \beta_0 + \beta_{c'} X_i + \beta_b M_i + r_i.$

The difference between the estimates of the coefficients associated with the initial variable (X_i) in these two equations, $\beta_c - \beta_{c'}$, estimates the mediated effect as the extent to which the mediator accounts for the relationship between the initial and outcome variables (Judd & Kenny, 1981).

Finally, calculating point estimates of mediated effects requires estimating a regression equation predicting the mediator from the initial variable

$$(3)\quad M_i = \beta_0 + \beta_a X_i + r_i$$

A straightforward reformulation of these single-level equations to the multilevel framework allows for the analogous estimation of mediated effects in repeated measures multilevel data. This reformulation is show here for the ith repeated measures nested under the jth subject:

Equation 1 $Y_{ij} = \beta_0 + \beta_c X_j + r_{ij}$ Level 1: $Y_{ij} = \beta_{0j} + r_{ij}$
Level 2: $\beta_{0j} = \gamma_{00} + \gamma_c X_j + u_{0j}$

Equation 2 $Y_{ij} = \beta_0 + \beta_{c'} X_j + \beta_b M_j + r_{ij}$ Level 1: $Y_{ij} = \beta_{0j} + r_{ij}$
Level 2: $\beta_{0j} = \gamma_{00} + \gamma_{c'} X_j + \gamma_b M_{ij} + u_{0j}$

Equation 3 $M_j = \beta_0 + \beta_a X_j + r_j$

This model includes both initial and mediator variables at the subject-level (j). For such a model, Equation 1 is formulated as for the previous model, with the X_j variable predicting the group intercept in the Level 2 equation. Equation 2 includes both the group level initial variable X_j and the subject-level mediator M_j in the subject-level equation. In this set of multilevel equations, only the intercept term has been specified as a random coefficient.

R. M. Baron and D. A. Kenny. The moderator-mediator variable distinction in social psychological research: Conceptual, strategic, and statistical considerations. Journal of. *Personality and Social Psychology* 51:1173-1182, 1986.

J. L. Krull and D. P. MacKinnon. Multilevel modeling of individual and group level mediated effects. *Multivariate Behavior Research* 36 (2):249-277, 2001.

C. M Judd and D. A. Kenny. Process analysis: Estimating mediation in treatment evaluations. *Evaluation Review* 5:602-619, 1981.

(3) Intention to Treat and Missing Data

To address the difficulty of incomplete data, an "intention to treat" (ITT) analysis will be conducted, including data on all subjects who were randomized to intervention or control groups. The ITT approach: 1) preserves the effects of randomization, and 2) addresses the practical impact of interventions better than per protocol analyses. Because of potential consequences of missing data, it is a major goal of our trial to keep missing values to minimal. Inevitably, however, there will be some missing values, both in terms of sporadically missing items from those that complete the protocol, and from people who withdraw or are lost to follow-up. Several ad hoc methods for dealing with missing data in clinical trials have been used and were considered.(1-5)

To deal with issues involved with participants who withdraw or are lost to follow-up, we propose dividing our subjects into two groups; protocol completers and non-completers. Variables based on these groupings will then be assessed for meeting the assumptions of either MCAR using Little's test (7) or MAR, using procedures proposed by Pottoff, et., al (8). Contingent upon achieving either MCAR or MAR, the following imputation strategy will be used for our ITT analysis. If the missing values are NMAR, then standard methods of analysis are not valid and usually a sensitivity analysis is recommended. Stage 1 imputation will proportionally randomize protocol non-completers to either a "no ARI episode" (zero) designation or to a designation of ≥ 1 ARI episodes, with variable duration and global severity. In the first MEPARI study, 66 of 149 people (44.3%) randomized had at least one ARI illness episode. Stage 2 will then use multiple imputation methods (9) to impute the specific missing values of AUC and duration for those subject imputed to a non-zero ARI illness situation. The issue of sporadically missing data from protocol completers is most relevant to the primary outcome of global severity, assessed as area-under-the-time-severity curve, where the X-axis = duration and Y-axis = severity. For sporadically missing WURSS-24 data, potential patterns of missing data will be assessed for MAR (8), and if applicable, imputation will be done using an expectancy maximizing multiple imputation strategy.(4) Any and all decisions and data manipulations will be made by analysts who remain blinded to allocation.

Reference List

1. Auleley GR, Giraudeau B, Baron G, Maillefert JF, Dougados M, Ravaud P. The methods for handling missing data in clinical trials influence sample size requirements. Journal of Clinical Epidemiology 2004;57:447-53.
2. Carpenter J, Pocock S, Lamm CJ. Coping with missing data in clinical trials: a model-based approach applied to asthma trials. Statistics in Medicine 2002;21:1043-66.
3. Wood AM, White IR, Thompson SG. Are missing outcome data adequately handled? A review of published randomized controlled trials in major medical journals. Clinical Trials 2004;1:368-76.
4. Schafer JL. Analysis of Incomplete Multivariate Data. London: Chapman & Hall, 1997.
5. Chakraborty, H., and Gu, H. (2009). A mixed model approach for Intent-to-Treat analysis in longitudinal clinic trails with missing values. RTI Press publication No. MR-0009-0903. Research Triangle Park, NC: RTI International.
6. Hedeker, D. and Gibbons, R. D. (1997). Application of random-effects pattern-mixture models for missing data in longitudinal studies. *Psychological Methods*, 2(1), 64-78.
7. Little, R. J. A. (1988). A test of missing completely at random for multivariate data with missing values. *Journal of the American Statistical Association,* 83(404), 1198-1202.
8. Potthoff, R., F., Tudor, G. E., Pieper, K. S., and Hasselblad, V. (2006). Can one assess whether missing data are missing at random in medical studies? *Statistical Methods in Medical Research*, 15, 213-234.
9. Schafer, J. L., and Graham, J. W., (2002). Missing data: Our view of the state of the art. *Psychological Methods*, 7(2), 147-177.

(4) Sample Size and Power Analyses

The planned study size of n=396 consenting individuals randomized to 3 groups leading to n=360 people completing the protocol (n=120 per group) is based on the results of our preliminary trial.1 For primary efficacy analyses, null hypotheses will be rejected if interventions are superior to control at a $p \leq 0.025$, using one-sided testing, based on two-way contrasts between: 1) meditation vs. control and 2) exercise vs. control. The $p \leq 0.025$ cut-off is chosen to be conservative and to allay concerns related to multiple comparison considerations. One-sided testing is supported by our own data and by available scientific literature. Given these parameters and our preliminary data, the sample size of n=120 per group should provide adequate power. However, when taking into account the zero-inflated and skewed nature of ARI illness data, and potential missing data and intention to treat considerations, actual power may be less. Given limitations on resources available, and a desire to minimize chances of both Type 1 and Type 2 errors, we feel that the proposed sample size will be adequate for this phase 2 randomized controlled human subjects clinical trial.

The power graphics below for global severity (AUC) and total days of illness (duration) are based on data from the first MEPARI trial. The first two are based on the zero-inflated model, which will be the model for testing of main hypotheses regarding primary outcomes (Mediation and Exercise versus control). The next six graphics show power curves based on simple t-test contrasts of the zero-inflated distributions.

Based on a log transformation of AUC of zero-inflated distribution

Approximately 100 patients per group will be needed for 1-tailed (0.025 alpha) test.

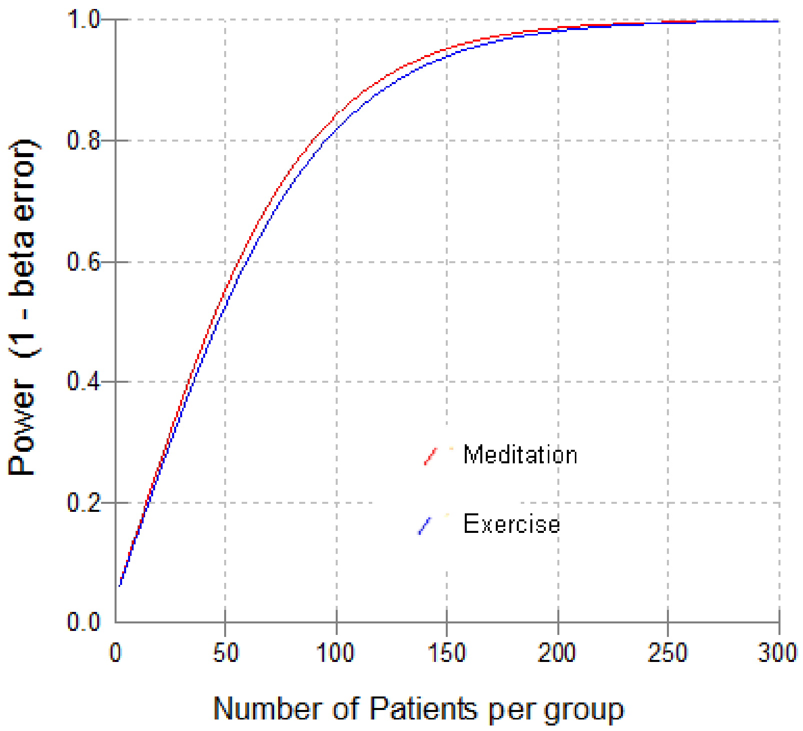

Based on a log transformation of duration of zero-inflated distribution

Approximately 106 patients per group will be required for 1-tailed (0.025 alpha) test.

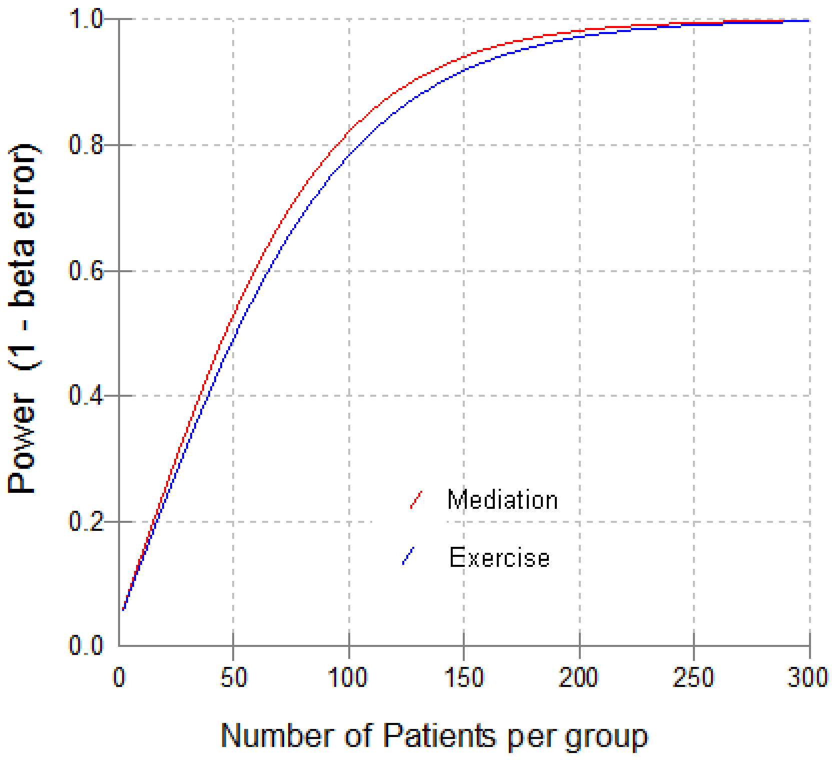

Two-Sample t Test for Mean Difference

Area Under the Curve- Exercise Vs Conttrol

Two-Sample t Test for Mean Difference

Fixed Scenario Elements	
**Distribution**	Normal
**Method**	Exact
**Number of Sides**	1
**Mean Difference**	109.5
**Standard Deviation**	535.2
**Nominal Power**	0.8
**Null Difference**	0

Computed N Per Group

Index	Alpha	Actual Power	N Per Group
**1**	0.050	0.801	297
**2**	0.025	0.800	376

Two-Sample t Test for Mean Difference

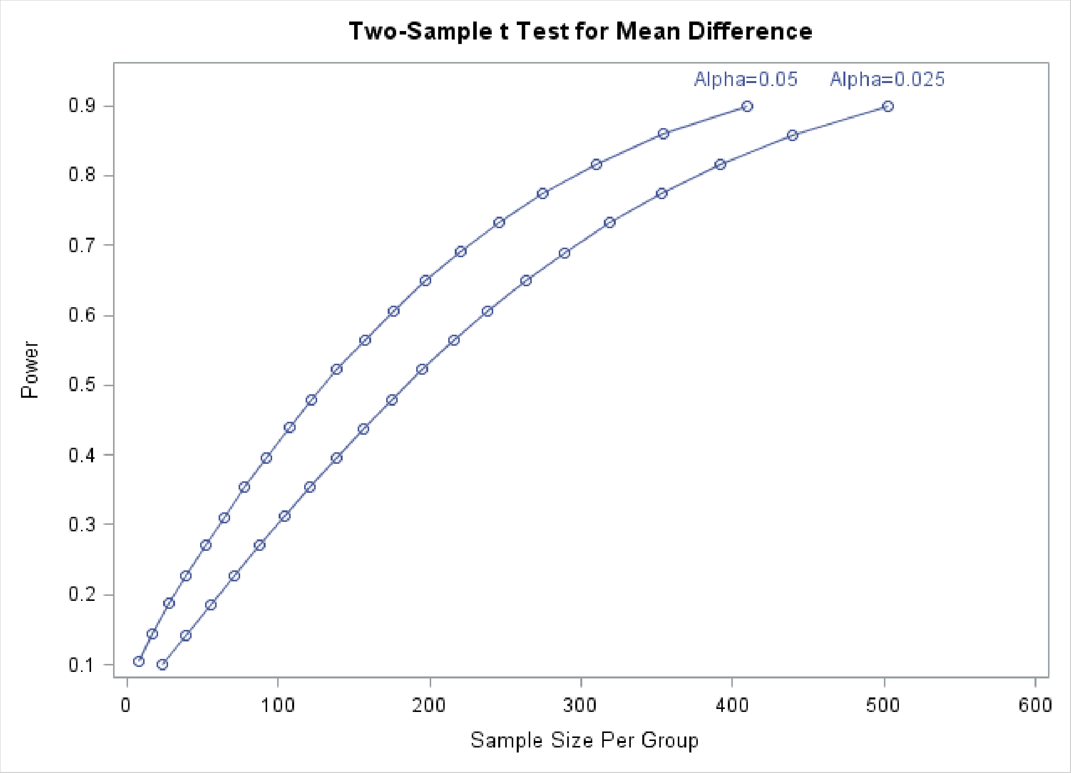

Duration of Illness- Exercise Vs Control

Two-Sample t Test for Mean Difference

Fixed Scenario Elements	
**Distribution**	Normal
**Method**	Exact
**Number of Sides**	1
**Mean Difference**	3.76
**Standard Deviation**	9.95
**Nominal Power**	0.8
**Null Difference**	0

Computed N Per Group

Index	Alpha	Actual Power	N Per Group
**1**	0.050	0.803	88
**2**	0.025	0.800	111

Two-Sample t Test for Mean Difference

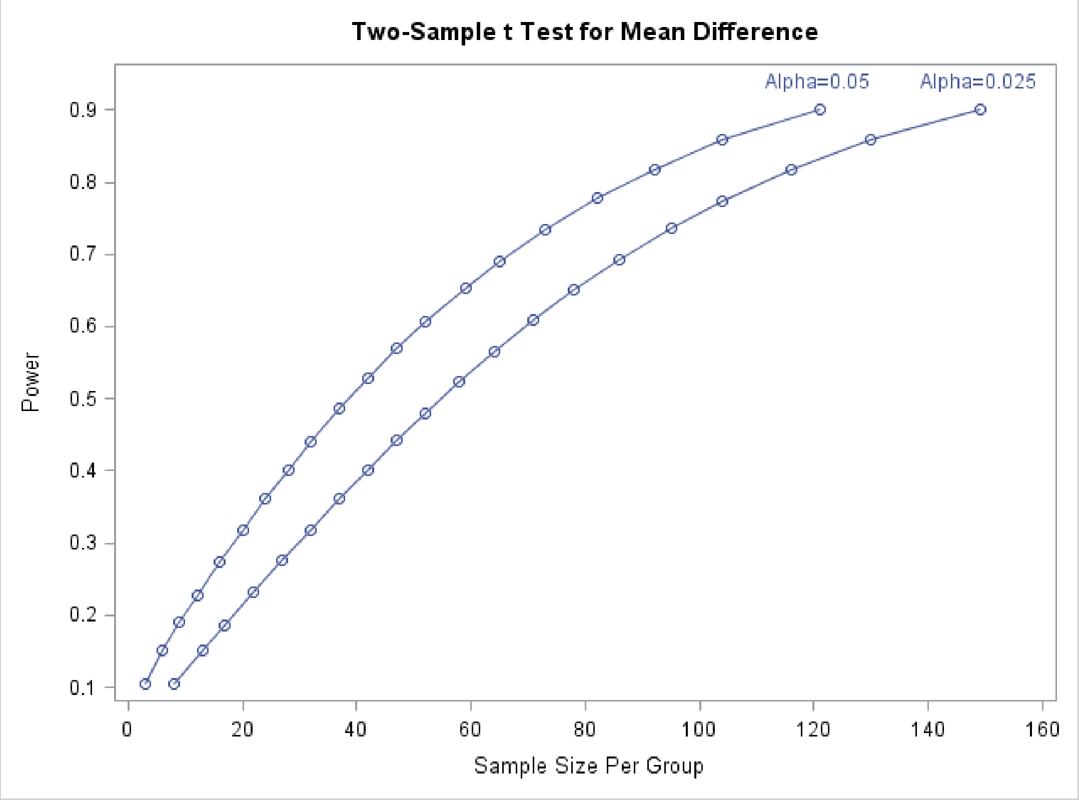

Area Under the Curve - Meditation Vs Control

Two-Sample t Test for Mean Difference

Fixed Scenario Elements	
**Distribution**	Normal
**Method**	Exact
**Number of Sides**	1
**Mean Difference**	214.4
**Standard Deviation**	400.9
**Nominal Power**	0.8
**Null Difference**	0

Computed N Per Group

Index	Alpha	Actual Power	N Per Group
**1**	0.050	0.801	44
**2**	0.025	0.801	56

Two-Sample t Test for Mean Difference

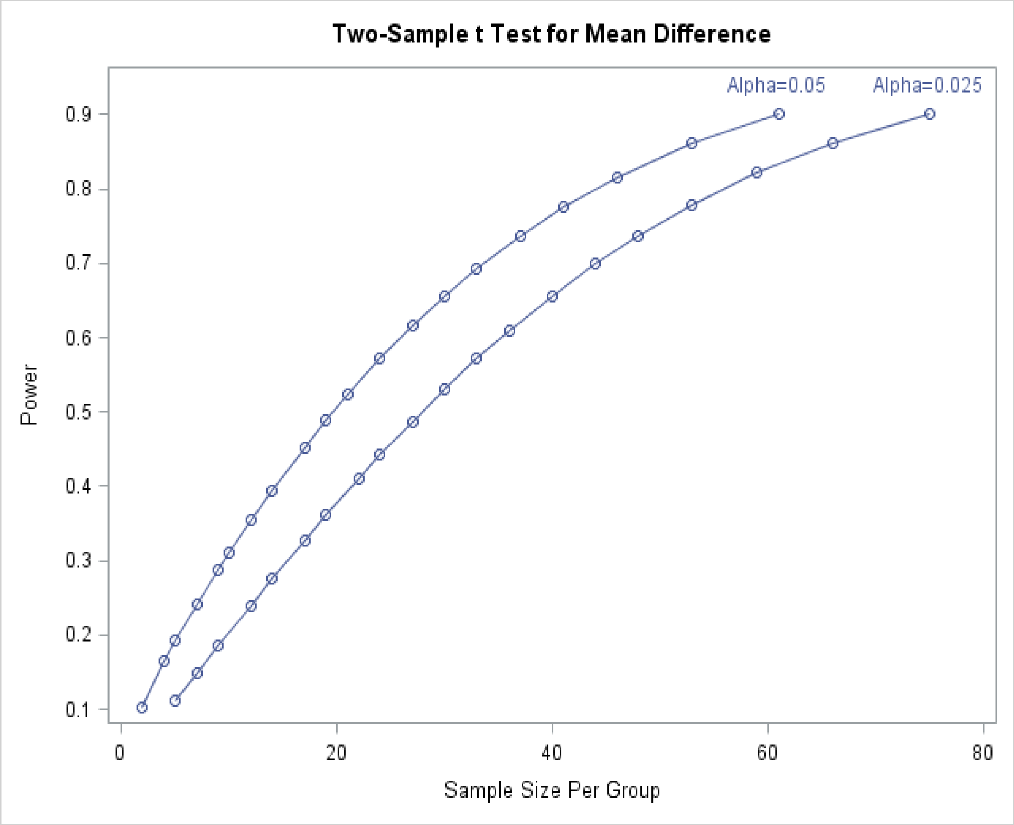

Duration of Illness - Meditation Vs Control

Two-Sample t Test for Mean Difference

Fixed Scenario Elements	
**Distribution**	Normal
**Method**	Exact
**Number of Sides**	1
**Mean Difference**	3.85
**Standard Deviation**	10.54
**Nominal Power**	0.8
**Null Difference**	0

Computed N Per Group

Index	Alpha	Actual Power	N Per Group
**1**	0.050	0.802	94
**2**	0.025	0.801	119

Two-Sample t Test for Mean Difference

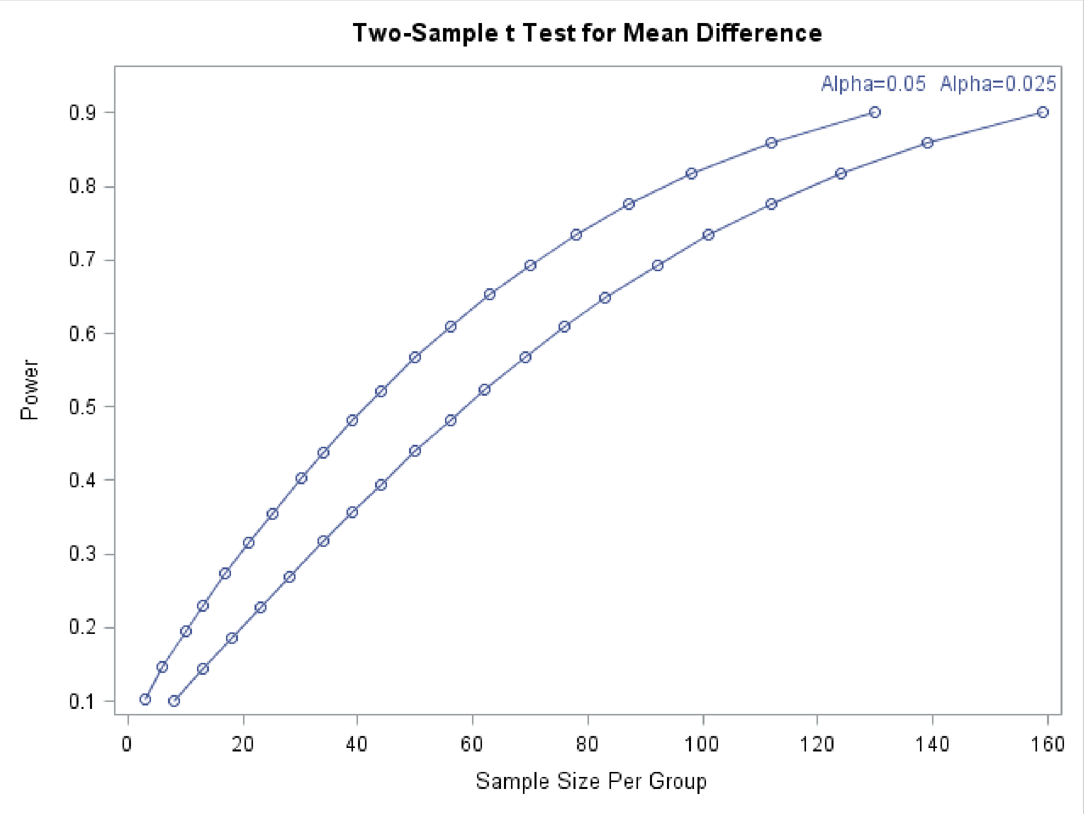

Area Under the Curve - Meditation Vs Exercise

Two-Sample t Test for Mean Difference

Fixed Scenario Elements	
**Distribution**	Normal
**Method**	Exact
**Number of Sides**	1
**Mean Difference**	104.9
**Standard Deviation**	453.8
**Nominal Power**	0.8
**Null Difference**	0

Computed N Per Group

Index	Alpha	Actual Power	N Per Group
**1**	0.050	0.801	233
**2**	0.025	0.800	295

Two-Sample t Test for Mean Difference

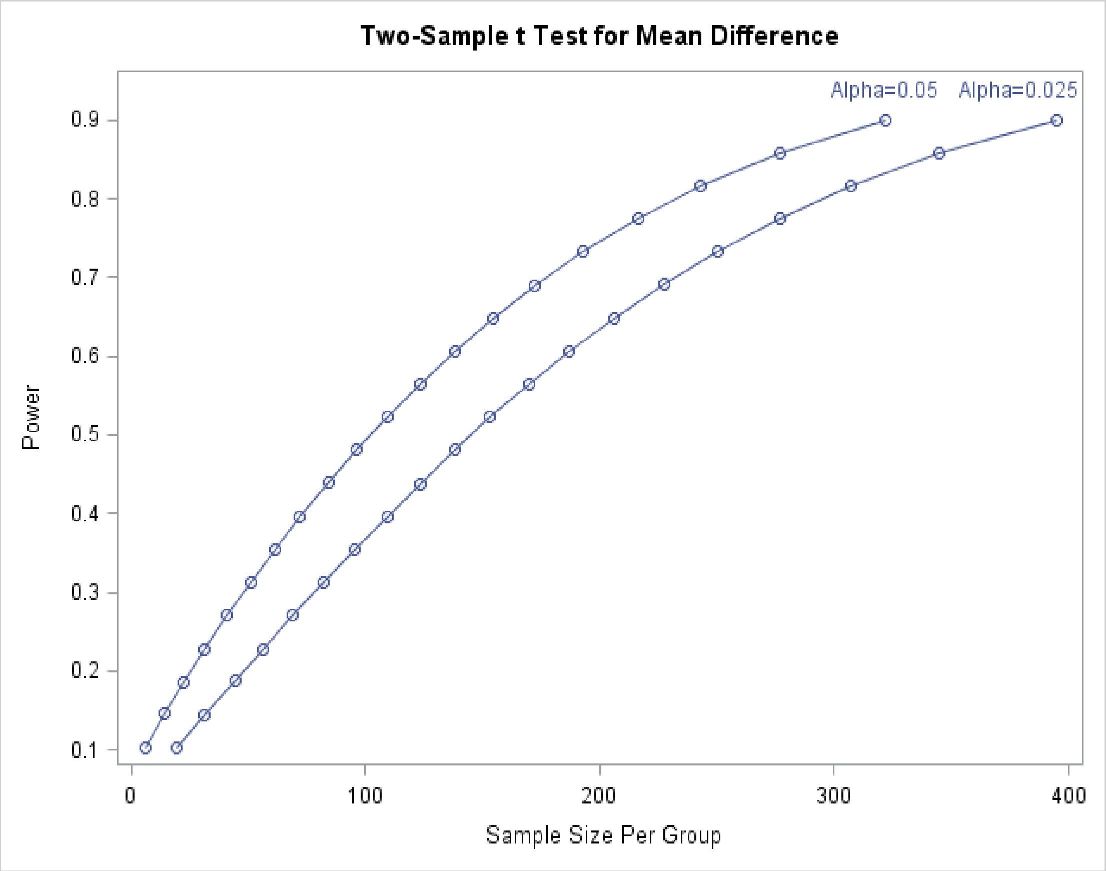

Duration of Illness - Meditation Vs Exercise

Two-Sample t Test for Mean Difference

Fixed Scenario Elements	
**Distribution**	Normal
**Method**	Exact
**Number of Sides**	1
**Mean Difference**	0.09
**Standard Deviation**	9.25
**Nominal Power**	0.8
**Null Difference**	0

Computed N Per Group

Index	Alpha	Actual Power	N Per Group
**1**	0.050	0.800	130617
**2**	0.025	0.800	165821

Two-Sample t Test for Mean Difference

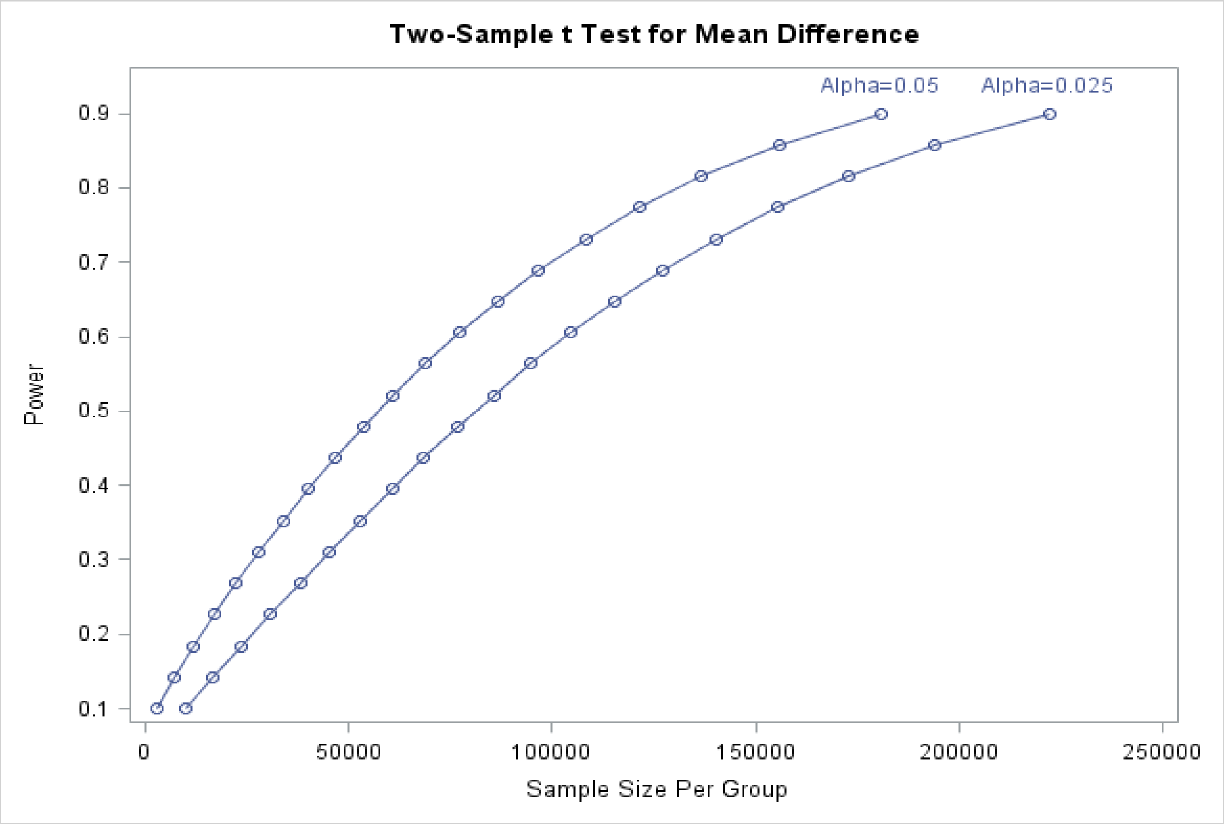

Appendix I: Study Log (Version dated 2-22-2015)

STUDY LOG: DECISIONS, MODIFICATIONS and CLARIFICATIONS

**Investigator:** Bruce Barrett MD PhD	**Protocol:** Meditation or Exercise for Preventing Acute Respiratory Infection (MEPARI-2)	**Protocol: 1 R01 AT006970-01**

Date	Item	Description
January 27, 2011	Proposal	MEPARI-2 R01 application submitted to NIH
June 6, 2011	NIH Review	Reviewed by MESH study section at NIH
October, 2011	NCCAM review	Reviewed by Council at NCCAM NIH
March 1, 2012	Start date	Requested start date
March 20, 2012	IRB application	March 16 protocol submitted to UW IRB human subjects committee
March 30, 2012	NCCAM responses	March 30 protocol with Response to Review sent to NCCAM. Changes include: change to permuted variable-sized block randomization (5.2); specifying secondary hypotheses (7.2.6); adding a paragraph about intention-to-treat (7.2.9); modifying Appendix H
April 13, 2012	IRB response	Response to IRB-pre-review. Changes include: new pregnancy section (8.6.1) for vulnerable populations; revised consent forms; revised data and safety monitoring plan; clarified phone screen and consent procedure forms.
April 23, 2012	IRB approval	Health Sciences Protocol IRB: 2012-0207
May 24, 2012	NCCAM response	Randomization code management plan changes
May 11, 2012	Co-I meeting => IRB modification	HgA1C results will be provided to all participants
June 7, 2012	Protocol clarification	ARI surveillance, absenteeism & health care use will be monitored starting early September, at the beginning and not the end of 8-week trainings
June 29, 2012	Co-I meeting => Protocol modifications & clarifications	Neutrophils will be captured for ARI, but not for baseline or standardized followup. Vital signs from CRU visits will be entered in database ; blood pressure will be analyzed as secondary outcome. Pro-inflammatory cytokines will be anal yzed as secondary outcomes
July 18, 2012	Protocol finalized	Protocol finalized with reference to July 9 publication of results of first MEPARI trial in *Annals of Family Medicine*
July 30, 2012	Trial registration	Trial protocol registered with clinicaltrials.gov, registration number NCT01654289
Sept. 28, 2012	Co-I meeting => Clarification of analysis plan	Intention to treat (ITT): all ARI episodes, missed work, etc that occur after randomization envelopes are opened. For cohort 1 (2012-13) the following date will serve as cut-off for ITT analysis: Sept. 3. Per protocol (PP): Only people who attend at least 5 of the 9 exercise or mindfulness sessions will be included. For cohort 1 (2012-13) the following date will serve as cut-off for PP analysis: Oct. 22.
April 26, 2013	Co-I meeting => Clarification of cut-off date	For Cohort 1, the monitoring period will end at 11:59 p.m. on May 19, 2013. Any ARI illness episodes ongoing at that time will continue to be monitored for up to 14 days of illness duration, and included in primary efficacy analysis. Associated ARI-related absenteeism and health care utilization will also be assessed and included in analysis.

Appendix I: Study Log (Version dated 2-22-2015)

STUDY LOG: DECISIONS, MODIFICATIONS and CLARIFICATIONS

**Investigator:** Bruce Barrett MD PhD	**Protocol:** Meditation or Exercise for Preventing Acute Respiratory Infection (MEPARI-2)	**Protocol: 1 R01 AT006970-01**
**Date**	**Item**	**Description**
July 27, 2013	PHQ-9 administration	The Westat site monitor noted that a single sentence in the approved 7-18-12 protocol specifies the PHQ-9 is to be administered at the run-in visit and again at the baseline visit. For Cohort 1, it was administered only at the run-in visit. The decision was made that remaining cohorts will continue to have the PHQ-9 administered only once at baseline, during the run-in visit.
Sept. 6, 2013	Co-I meeting => Clarification of analysis plan	Intention to treat (ITT): all ARI episodes, missed work, etc. that occur after randomization envelopes are opened. For cohort 2 (2013-14) Sept. 7, 2013, will serve as cut-off for ITT analysis. Only ARI starting on or after Sept. 7, 2013, will be included. Per protocol (PP): Only people who attend at least 5 of the 9 exercise or mindfulness sessions will be included. For cohort 2 (2013-14) Oct. 21, 2013, will serve as cut-off for PP analyses. Only those ARIs that start on or after Oct. 21, 2013, will be included in PP analyses.
Oct 18, 2013	Co-I meeting => Clarification of analysis plan	Intention to treat (ITT): We will count all ARI episodes, missed work, etc. that occur after randomization envelopes are opened for each individual; we will not use standard dates to apply across all participants. This clarification was decided upon by full CoI committee after Dr Barrett discussed the issue with study advisor Dr David DeMets on October 8. This does not affect PP analysis, where cohort-specific standard dates will be used as described above.
Nov 8, 2013	Co-I meeting => Clarification of retention plan	After some back-and-forth with IRB regarding an individual who changed her mind about willingness to accept flu shot, it was agreed to continue original plan, with clarification, "Unless there are safety concerns or other sufficiently strong reasons, the study team will strive to retain and monitor all randomized participants. Changing status in relation to eligibility criteria will not be grounds for withdrawing participants. "
Feb 6, 2014	Co-I meeting => Procalcitonin to cease	Virtually all of the procalcitonin results collected from Cohort 1 participants were reported as undetectable, even during ARI episodes. Due to substantive cost and because we can't usefully analyze these results, it was decided at the Jan 31 Co-I meeting to cease processing procalcitonin as a study biomarker. Confirmed by NIH NCCAM study officer John Glowa in emails 2-6-14
March 28, 2014	Co-I meeting => Clarification of cut-off date	For Cohort 2, the monitoring period will end at 11:59 p.m. on May 18, 2014. Any ARI illness episodes ongoing at that time will continue to be monitored for up to 14 days of illness duration, and included in primary efficacy analysis. Associated ARI-related absenteeism and health care utilization will also be assessed and included in analysis.
August 15, 2014	Protocol modification	Per Westat's recommendations, deleted Procalcitonin from StudyProtocol document.
August 27, 2014	Nasal wash collection	Clarified standard operating procedures for nasal wash collection process.

Appendix I: Study Log (Version dated 2-22-2015)

STUDY LOG: DECISIONS, MODIFICATIONS and CLARIFICATIONS

**Investigator:** Bruce Barrett MD PhD	**Protocol:** Meditation or Exercise for Preventing Acute Respiratory Infection (MEPARI-2)	**Protocol: 1 R01 AT006970-01**
**Date**	**Item**	**Description**
Sept. 26, 2014	Co-I meeting => Clarification of analysis plan	Intention to treat (ITT): all ARI episodes, missed work, etc. that occur after randomization envelopes are opened. Per protocol (PP): Only people who attend at least 5 of the 9 exercise or mindfulness sessions will be included FOR AT LEAST 60 MINUTES. For cohort 3 (2014-15) Oct. 20, 2014, will serve as cut-off for PP analyses. Only those ARIs that start on or after Oct. 20, 2014, will be included in PP analyses.
Oct. 17, 2014	Co-I meeting Clarification of data mgmt.	Missed hours of volunteer work will not be counted as missed "work" in analyses.
Dec. 5, 2014	Co-I meeting Clarification of data mgmt..	Will exclude from analysis all follow-up 1 visit data (biomarkers and November/December questionnaires) for Participant #368 who was in a serious car accident.
Jan. 9, 2015	Co-I meeting Clarification of data mgmt..	Will exclude from analysis all follow-up 1 visit data (biomarkers and November/December questionnaires) for Participant #401 who was diagnosed with breast cancer.
May 8, 2015	Co-I meeting => Clarification of cut-off date	For Cohort 3, the monitoring period will end at 11:59 p.m. on May 17, 2015. Any ARI illness episodes ongoing at that time will continue to be monitored for up to 14 days of illness duration, and included in primary efficacy analysis. Associated ARI-related absenteeism and health care utilization will also be assessed and included in analysis.
May 29, 2015	Accelerometry & breath-counting (ABC) in 4th cohort	Optional ABC sub-study. Willing participants in the 4th cohort will do accelerometry assessment and breath-counting tasks at baseline, then at two post-intervention follow-up points, as described in Appendix J. Protocol modification approved by NCCIH, and submitted to IRB for review.
August 28, 2015	Co-I meeting => Clarification of analysis plan	Intention to treat (ITT): all ARI episodes, missed work, etc. that occur after randomization envelopes are opened. Per protocol (PP): Only people who attend at least 5 of the 9 exercise or mindfulness sessions will be included FOR AT LEAST 60 MINUTES. For cohort 4 (2015-16) Oct. 19, 2015, will serve as cut-off for PP analyses. Only those ARIs that start on or after October 19, 2015, will be included in PP analyses.
February 19, 2016	Co-I meeting => Clarification of cut-off date	For Cohort 4, the monitoring period will end at 11:59 p.m. on May 15, 2016. Any ARI illness episodes ongoing at that time will continue to be monitored for up to 14 days of illness duration, and included in primary efficacy analysis. Associated ARI-related absenteeism and health care utilization will also be assessed and included in analysis.

APPENDIX J: **MEPARI-2 Sub-Study Protocol**
Accelerometry and Breath Counting (ABC)

Bruce Barrett MD PhD et al.

May 29, 2015

Background

Currently in the MEPARI 2 study, we are assessing exercise and meditation practice with weekly online self-reports. Participants randomized to receive meditation training document the number of minutes of formal and informal practice for each day of that week. Those in the exercise group similarly document the number of minutes of moderate and strenuous exercise by self-report each week. People in the control and exercise group do not report minutes of meditation practice, and those in the control and meditation group do not report minutes of exercise. However, participants in all three groups fill out the Mindfulness Attention Awareness Scale (MAAS) and Global Physical Activity Questionnaire (GPAQ) at baseline, and then approximately every 2 months throughout the study. To date, the study has not included any "objective" measures of mindfulness or exercise. Adding in breath-counting and accelerometry measures for willing participants in the final cohort will strengthen the study by providing some objective evidence of sustained mindful attention (breath-counting) and physical activity (accelerometry), before and after interventions, in all three study groups. Resulting data will: 1) be correlated with self-reported practice data, and 2) be used for between-group exploratory efficacy analyses. Adding these measures for all willing participants will provide potentially valuable data, but will not interfere with or change the original aims or outcome measures for this NIH R01 funded phase 2 trial. The sponsoring entity at NIH, the National Center for Complementary and Integrative Health, has approved this optional add-on sub-study.

Recruitment

Up to 100 participants will be recruited from those being enrolled in the MEPARI 2 Run-In study. At the end of the first run-in visit, participants will be given information about the sub-study and asked if they would like to participate. If they would like to participate, they will either set up another visit at another date/time, or be taken to a different room where they can consent and enroll at that time. Since participants will be approached for this optional sub-study during the run-in phase, before randomization envelopes are opened, this will ensure approximate equal numbers of participants being assessed by accelerometry and breath-counting in each of the three study groups, and will guard against any bias which might arise when participants know to which group they have been assigned.

Inclusion/Exclusion Criteria

All participants who enroll in the run-in phase of the MEPARI-2 study are eligible to participate in the sub-study. Only those that successfully complete run-in tasks and are enrolled in the main MEPARI-2 study will continue to be in the ABC sub-study.

Enrollment/Study visits
Participants will be consented at the Clinical Research Unit at the University of Wisconsin – Madison. There will be three ABC sub-study visits that will be arranged to coincide with MEPARI 2 visits: 1) run-in enrollment (baseline); 2) at the regular flu shot visit in November or early December, (first follow-up), and 3) at the second follow-up visit in March or early April. Each visit will take approximately 45 minutes to one hour. At each visit, the participant will complete a breath counting exercise and will be given an accelerometer to take with them and wear for seven days.

Breath Counting Exercise
Participants will be instructed to "be aware of the movement of breath" and count their breaths in cycles from one to nine repeatedly for 15 minutes. With breaths 1 – 8, they press one laptop key, and on breath 9, they press another, permitting an ongoing assessment of counting accuracy. If they lose count, participants are instructed to press a third key to indicate a self-caught error, and then begin counting again at 'one' with the next breath. Software developed and validated by the Richard Davidson laboratory (Center for Investigating Health Minds) will assess the accuracy of breath-counting. These methods have been used in several previous studies, and are described in a published validation paper by Daniel Levinson et al.[1]

Every ~90 seconds (range 60 – 120 seconds) a set of three probes appears in succession on the laptop screen. Two probes, using 6-point Likert scale response ranges, ask participants about their attentional state: "just now where was your attention?" (with responses ranging from "completely on task" to "completely off-task"), and "how aware were you of where your attention was?" (with responses ranging from "completely aware" to "completely unaware"). Third probe asks participants about their actual breath count at that moment ("what was your count?"). At that point, the participant goes back to counting breaths in 9 breath cycles.

Accelerometers
Actigraph accelerometers (Actigraph wGT3X-BT) [2] will be programmed before they are given to participants, with participant's screening ID, and initialized to collect data continuously in 1-second epochs. Participants will be provided with a soft belt to hold the accelerometer, and instructed to wear the accelerometer on their hipline during all waking hours (before they get dressed until they go to bed at night). They will be instructed to remove it when bathing, showering or swimming.

Accelerometers that are given out at the run-in visit will be collected when the participant returns for their baseline visit (between 4 – 21 days). If the participant decides not to participate, forgets the accelerometer, etc., they will be sent a self-addressed stamped envelope to return it or study personnel will collect it from the participant. At the flu shot and follow up 2 visits, participants will be given a self-addressed stamped envelope to return the accelerometer after they have worn it for 7 days.

Participant Payment

Participants will be paid $20 after accelerometers are collected at the baseline visit, flu shot visit and follow up 2 visit. If they complete all 3 visits, they will be paid $60 total. This is in addition to the approved compensation structure for the ongoing MEPARI-2 study.

Data security and integrity

Identifying information will be stored in a password-protected secure study database, which will not have direct links to process or outcome data. All other project data will be kept in password-protected security-ensured databases. Project personnel will be required to enter separate personal logins and passwords both for computer workstations specifically designated for the project and for the database user authentication process. Project databases will generate an unseen unique identifying code for each participant, separate from the study participant number used on data collection instruments, to connect the participant's multiple data elements. Association of that code with the study participant's identifying information will reside in an encrypted database separate from the study's research data, ensuring that no records in the dataset used for analysis will contain any data that could identify the participant.

Data analysts will be restricted to viewing outcome data, and will not be able to access personal identifiers. The database used to perform confidential study participant tracking and reimbursement auditing functions will be accessible only to the PI, the programmer, and to the involved research specialists, in conformity with UW HS-IRB requirements.

Data Analysis

Accelerometer data will be processed using the sojourn-3 axis method [3] to calculate minutes spent in sedentary, light, low-moderate, high-moderate, and vigorous levels of physical activity. A major advantage of this method of analysis is that it improves the classification of sedentary behavior and light intensity activities compared to single axis sojourns or traditional cut-point classification systems. Briefly, this method analyzes data in 1-second epochs using all three axes to identify bout intervals (starting and stopping activity), determine which intervals qualify as activity or inactivity and estimate metabolic equivalent (MET) values of each bout. This method does not use cut-off points for activity categories because it incorporates all three axes and a neural network to determine the wearer's movement pattern. Resulting MET values will then be broken into activity categories accordingly: <1.5 METs: sedentary, 1.5-2.99 METs: light, 3-6 METs: moderate and >6 METs: vigorous.

Both average sedentary time and minutes spent during sustained sedentary behavior will be calculated and used for the sedentary assessments. Sustained sedentary time is operationally defined as <1.5 METs sustained for at least 20 consecutive minutes. This is an adaptation from Ellingson and colleagues [4] examining shorter time intervals for sustained sedentary behavior to more thoroughly examine sedentary behavior.

Physical activity and sedentary time will be correlated to self-reports from the GPAQ using Pearson's *r* product-moment correlation coefficients and Spearman's ρ or log-transformations when data is non-normal (often the case for higher intensities of physical activity). Exploratory analyses will be performed comparing these measures of physical activity behaviors and the primary outcome variables from the main study.

Analysis of the breath-counting exercise is somewhat more straightforward, as there are only 15 minutes of data to contend with for each of the three observation periods. The primary indicator is the proportion of breath cycles that are accurately recorded (8 breaths keyed correctly followed by one breath keyed correctly). Counting accuracy will be calculated as the number of correct count sets divided by the total number of count sets, i.e., 100% – (# of incorrect ongoing 9-counts + # of incorrect count probe responses + # of self-caught miscounts) / (# of ongoing 9-counts + # of count probe responses + # of self-caught miscounts). Experience sampling during breath counting will yield a set of mind wandering ratings and a set of meta-awareness ratings. Each set will be averaged to index state mind wandering and state meta-awareness, respectively. For analyses of ratings accompanying correct vs. incorrect count probes, participants without data in both categories (e.g., never off count at probe) will be excluded.

Both accelerometry and breath-counting data will be compared to several self-report indicators, and will be assessed as an indicator of mindfulness (breath-counting) and physical activity (accelerometry). Breath-counting accuracy, mind-wandering and meta-awareness scores will be compared to self-report questionnaires rating mindfulness (MAAS), mindful self-efficacy (MSES), and positive and negative emotion (PANAS) for all participants, and to meditation logs for those receiving mindfulness trainings. Accelerometry data will be compared to exercise self-efficacy (ESES) and physical activity (GPAQ) scores for all participants, and to exercise logs for those in that group. Exploratory analyses will be undertaken to determine whether randomization to training in exercise or mindfulness influences accelerometry or breath-counting scores, and if so, whether those changes are sustained. The basic analysis structure described in the main protocol will not be changed. All results from this optional add-on sub-study will be interpreted with caution.

1. Levinson DB, Stoll EL, Kindy SD, Merry HL, Davidson RJ: A mind you can count on: validating breath counting as a behavioral measure of mindfulness. Front Psychol 2014, 5: 1202.

2. Welk GJ, McClain JJ, Eisenmann JC, Wickel EE: Field validation of the MTI Actigraph and BodyMedia armband monitor using the IDEEA monitor. Obesity (Silver Spring) 2007, 15: 918-928.

3. Lyden, Kate, Sarah Kozey Keadle, John Staudenmayer, and Patty S. Freedson. 2014. "A Method to Estimate Free-Living Active and Sedentary Behavior from an Accelerometer:" Medicine & Science in Sports & Exercise 46 (2): 386–97. doi:10.1249/MSS.0b013e3182a42a2d.

4. Ellingson, Laura D., Morgan R. Shields, Aaron J. Stegner, and Dane B. Cook. 2012. "Physical Activity, Sustained Sedentary Behavior and Pain Modulation in Women with Fibromyalgia." The Journal of Pain 13 (2): 195–206. doi:10.1016/j.jpain.2011.11.001.
