# Supplementary material for: Meditation or exercise for preventing acute respiratory infection (MEPARI-2): A randomized controlled trial
Source: PLoS One. 2018 Jun 22;13(6):e0197778. doi: 10.1371/journal.pone.0197778 (PMC6014660; doi:10.1371/journal.pone.0197778)
Supplement: S2 Checklist — (DOCX) [file pone.0197778.s005.docx]

**The TIDieR (Template for Intervention Description and Replication) Checklist*:**


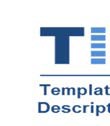

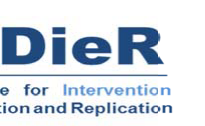


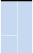
Information to include when describing an intervention and the location of the information

m

| **Meditation or exercise for preventing acute respiratory infection (MEPARI-2): A randomized controlled trial Where located **** | | | | |
| --- | --- | --- | --- | --- |
| **Item Item: MINDFULNESS MEDITATION INTERVENTION GROUP**  **number** | Primary paper (page or appendix number) | | Other † (details) | |
| **BRIEF NAME** |  | |  | |
| **1.** Provide the name or a phrase that describes the intervention. | Cover page; Line 51; Line 125 | | Clinicaltrials.gov  NCT01654289 | |
| **WHY** |  | |  | |
| **2.** Describe any rationale, theory, or goal of the elements essential to the intervention. | Line 83-99 | | Clinicaltrials.gov  NCT01654289 and Protocol 5.29.15 | |
| **WHAT** |  | |  | |
| **3.** Materials: Describe any physical or informational materials used in the intervention, including those  provided to participants or used in intervention delivery or in training of intervention providers.  Provide information on where the materials can be accessed (e.g. online appendix, URL). | Lines 124-131 | | Clinicaltrials.gov  NCT01654289  and Protocol 5.29.15 | |
| **4.** Procedures: Describe each of the procedures, activities, and/or processes used in the intervention,  including any enabling or support activities. | Lines 124-131 | | Clinicaltrials.gov  NCT01654289 and Protocol 5.29.15 | |
| **WHO PROVIDED** |  | |  | |
| **5.** For each category of intervention provider (e.g. psychologist, nursing assistant), describe their  expertise, background and any specific training given. | Lines 125-6; 437-8. | | Protocol 5.29.15 (Section 5.1.1) | |
| **HOW** |  | |  | |
| **6.** Describe the modes of delivery (e.g. face-to-face or by some other mechanism, such as internet or  telephone) of the intervention and whether it was provided individually or in a group. | Lines 124-131  Cite #17 | | Protocol 5.29.15 (Section 5.1.1) | |
| **WHERE** |  | |  | |
| **7.** Describe the type(s) of location(s) where the intervention occurred, including any necessary  infrastructure or relevant features. | Lines 124-131 | | Protocol 5.29.15 (Section 5.1.1) | |
| **WHEN and HOW MUCH** | | |  |  |
| **8.** Describe the number of times the intervention was delivered and over what period of time including  the number of sessions, their schedule, and their duration, intensity or dose. | | | Lines 124-131  Cite #17 | Protocol 5.29.15 (Section 5.1.1) |
| **TAILORING** | | |  |  |
| **9.** If the intervention was planned to be personalised, titrated or adapted, then describe what, why,  when, and how. | | | N/A |  |
| **MODIFICATIONS** | | |  |  |
| **10.ǂ** If the intervention was modified during the course of the study, describe the changes (what, why,  when, and how). | | | N/A |  |
| **HOW WELL** | | |  |  |
| **11.** Planned: If intervention adherence or fidelity was assessed, describe how and by whom, and if any  strategies were used to maintain or improve fidelity, describe them. | | | Lines 136-42; 218-22 | Protocol 5.29.15 (Section 5.4.2)) |
| **12.ǂ** Actual: If intervention adherence or fidelity was assessed, describe the extent to which the  intervention was delivered as planned. | | | Lines 136-42; 218-22 |  |

** **Authors** - use N/A if an item is not applicable for the intervention being described. **Reviewers** – use ‘?’ if information about the element is not reported/not sufficiently reported.

† If the information is not provided in the primary paper, give details of where this information is available. This may include locations such as a published protocol or other published papers (provide citation details) or a website (provide the URL).

ǂ If completing the TIDieR checklist for a protocol, these items are not relevant to the protocol and cannot be described until the study is complete.

- We strongly recommend using this checklist in conjunction with the TIDieR guide (see *BMJ* 2014;348:g1687) which contains an explanation and elaboration for each item.
- The focus of TIDieR is on reporting details of the intervention elements (and where relevant, comparison elements) of a study. Other elements and methodological features of studies are covered by other reporting statements and checklists and have not been duplicated as part of the TIDieR checklist. When a **randomised trial** is being reported, the TIDieR checklist should be used in conjunction with the CONSORT statement (see www.consort‐statement.org) as an extension of **Item 5 of the CONSORT 2010 Statement.**

When a **clinical trial protocol** is being reported, the TIDieR checklist should be used in conjunction with the SPIRIT statement as an extension of **Item 11 of the SPIRIT 2013 Statement** (see www.spirit‐statement.org). For alternate study designs, TIDieR can be used in conjunction with the appropriate checklist for that study design (see www.equator‐network.org).

**The TIDieR (Template for Intervention Description and Replication) Checklist*:**


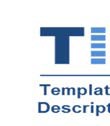

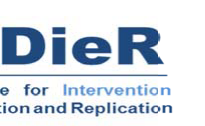


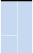
Information to include when describing an intervention and the location of the information

m

| **Meditation or exercise for preventing acute respiratory infection (MEPARI-2): A randomized controlled trial Where located **** | | | | |
| --- | --- | --- | --- | --- |
| **Item Item: EXERCISE INTERVENTION GROUP**  **number** | Primary paper (page or appendix number) | | Other † (details) | |
| **BRIEF NAME** |  | |  | |
| **1.** Provide the name or a phrase that describes the intervention. | Cover page; Line 51-2; Line 126 | | Clinicaltrials.gov  NCT01654289 | |
| **WHY** |  | |  | |
| **2.** Describe any rationale, theory, or goal of the elements essential to the intervention. | Line 83-99 | | Clinicaltrials.gov  NCT01654289 and Protocol 5.29.15 | |
| **WHAT** |  | |  | |
| **3.** Materials: Describe any physical or informational materials used in the intervention, including those  provided to participants or used in intervention delivery or in training of intervention providers.  Provide information on where the materials can be accessed (e.g. online appendix, URL). | Lines 124-131 | | Clinicaltrials.gov  NCT01654289  and Protocol 5.29.15 | |
| **4.** Procedures: Describe each of the procedures, activities, and/or processes used in the intervention,  including any enabling or support activities. | Lines 124-131 | | Clinicaltrials.gov  NCT01654289 and Protocol 5.29.15 | |
| **WHO PROVIDED** |  | |  | |
| **5.** For each category of intervention provider (e.g. psychologist, nursing assistant), describe their  expertise, background and any specific training given. | Lines 128-9; 437-8. | | Protocol 5.29.15 (Section 5.1.1) | |
| **HOW** |  | |  | |
| **6.** Describe the modes of delivery (e.g. face-to-face or by some other mechanism, such as internet or  telephone) of the intervention and whether it was provided individually or in a group. | Lines 124-131 | | Protocol 5.29.15 (Section 5.1.1) | |
| **WHERE** |  | |  | |
| **7.** Describe the type(s) of location(s) where the intervention occurred, including any necessary  infrastructure or relevant features. | Lines 124-131 | | Protocol 5.29.15 (Section 5.1.1) | |
| **WHEN and HOW MUCH** | | |  |  |
| **8.** Describe the number of times the intervention was delivered and over what period of time including  the number of sessions, their schedule, and their duration, intensity or dose. | | | Lines 124-131 | Protocol 5.29.15 (Section 5.1.1) |
| **TAILORING** | | |  |  |
| **9.** If the intervention was planned to be personalised, titrated or adapted, then describe what, why,  when, and how. | | | N/A |  |
| **MODIFICATIONS** | | |  |  |
| **10.ǂ** If the intervention was modified during the course of the study, describe the changes (what, why,  when, and how). | | | N/A |  |
| **HOW WELL** | | |  |  |
| **11.** Planned: If intervention adherence or fidelity was assessed, describe how and by whom, and if any  strategies were used to maintain or improve fidelity, describe them. | | | Lines 136-7; 218-22 | Protocol 5.29.15 (Section 5.4.2)) |
| **12.ǂ** Actual: If intervention adherence or fidelity was assessed, describe the extent to which the  intervention was delivered as planned. | | | Lines 136-7; 218-22 |  |

** **Authors** - use N/A if an item is not applicable for the intervention being described. **Reviewers** – use ‘?’ if information about the element is not reported/not sufficiently reported.

† If the information is not provided in the primary paper, give details of where this information is available. This may include locations such as a published protocol or other published papers (provide citation details) or a website (provide the URL).

ǂ If completing the TIDieR checklist for a protocol, these items are not relevant to the protocol and cannot be described until the study is complete.

- We strongly recommend using this checklist in conjunction with the TIDieR guide (see *BMJ* 2014;348:g1687) which contains an explanation and elaboration for each item.
- The focus of TIDieR is on reporting details of the intervention elements (and where relevant, comparison elements) of a study. Other elements and methodological features of studies are covered by other reporting statements and checklists and have not been duplicated as part of the TIDieR checklist. When a **randomised trial** is being reported, the TIDieR checklist should be used in conjunction with the CONSORT statement (see www.consort‐statement.org) as an extension of **Item 5 of the CONSORT 2010 Statement.**

When a **clinical trial protocol** is being reported, the TIDieR checklist should be used in conjunction with the SPIRIT statement as an extension of **Item 11 of the SPIRIT 2013 Statement** (see www.spirit‐statement.org). For alternate study designs, TIDieR can be used in conjunction with the appropriate checklist for that study design (see www.equator‐network.org).

**The TIDieR (Template for Intervention Description and Replication) Checklist*:**


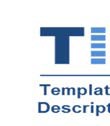

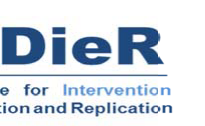


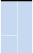
Information to include when describing an intervention and the location of the information

m

| **Meditation or exercise for preventing acute respiratory infection (MEPARI-2): A randomized controlled trial Where located **** | | | | |
| --- | --- | --- | --- | --- |
| **Item Item: CONTROL GROUP**  **number** | Primary paper (page or appendix number) | | Other † (details) | |
| **BRIEF NAME** |  | |  | |
| **1.** Provide the name or a phrase that describes the intervention. | Cover page; Line 52; Line 127 | | Clinicaltrials.gov  NCT01654289 | |
| **WHY** |  | |  | |
| **2.** Describe any rationale, theory, or goal of the elements essential to the intervention. | Line 83-99 | | Clinicaltrials.gov  NCT01654289 and Protocol 5.29.15 | |
| **WHAT** |  | |  | |
| **3.** Materials: Describe any physical or informational materials used in the intervention, including those  provided to participants or used in intervention delivery or in training of intervention providers.  Provide information on where the materials can be accessed (e.g. online appendix, URL). | N/A | | Clinicaltrials.gov  NCT01654289  and Protocol 5.29.15 | |
| **4.** Procedures: Describe each of the procedures, activities, and/or processes used in the intervention,  including any enabling or support activities. | N/A | | Clinicaltrials.gov  NCT01654289 and Protocol 5.29.15 | |
| **WHO PROVIDED** |  | |  | |
| **5.** For each category of intervention provider (e.g. psychologist, nursing assistant), describe their  expertise, background and any specific training given. | N/A | | Protocol 5.29.15 (Section 5.1.1) | |
| **HOW** |  | |  | |
| **6.** Describe the modes of delivery (e.g. face-to-face or by some other mechanism, such as internet or  telephone) of the intervention and whether it was provided individually or in a group. | N/A | | Protocol 5.29.15 (Section 5.1.1) | |
| **WHERE** |  | |  | |
| **7.** Describe the type(s) of location(s) where the intervention occurred, including any necessary  infrastructure or relevant features. | N/A | | Protocol 5.29.15 (Section 5.1.1) | |
| **WHEN and HOW MUCH** | | |  |  |
| **8.** Describe the number of times the intervention was delivered and over what period of time including  the number of sessions, their schedule, and their duration, intensity or dose. | | | N/A | Protocol 5.29.15 (Section 5.1.1) |
| **TAILORING** | | |  |  |
| **9.** If the intervention was planned to be personalised, titrated or adapted, then describe what, why,  when, and how. | | | N/A |  |
| **MODIFICATIONS** | | |  |  |
| **10.ǂ** If the intervention was modified during the course of the study, describe the changes (what, why,  when, and how). | | | N/A |  |
| **HOW WELL** | | |  |  |
| **11.** Planned: If intervention adherence or fidelity was assessed, describe how and by whom, and if any  strategies were used to maintain or improve fidelity, describe them. | | | Lines 135-6. | Protocol 5.29.15 (Section 5.4.2)) |
| **12.ǂ** Actual: If intervention adherence or fidelity was assessed, describe the extent to which the  intervention was delivered as planned. | | | Lines 135-6 |  |

** **Authors** - use N/A if an item is not applicable for the intervention being described. **Reviewers** – use ‘?’ if information about the element is not reported/not sufficiently reported.

† If the information is not provided in the primary paper, give details of where this information is available. This may include locations such as a published protocol or other published papers (provide citation details) or a website (provide the URL).

ǂ If completing the TIDieR checklist for a protocol, these items are not relevant to the protocol and cannot be described until the study is complete.

- We strongly recommend using this checklist in conjunction with the TIDieR guide (see *BMJ* 2014;348:g1687) which contains an explanation and elaboration for each item.
- The focus of TIDieR is on reporting details of the intervention elements (and where relevant, comparison elements) of a study. Other elements and methodological features of studies are covered by other reporting statements and checklists and have not been duplicated as part of the TIDieR checklist. When a **randomised trial** is being reported, the TIDieR checklist should be used in conjunction with the CONSORT statement (see www.consort‐statement.org) as an extension of **Item 5 of the CONSORT 2010 Statement.**

When a **clinical trial protocol** is being reported, the TIDieR checklist should be used in conjunction with the SPIRIT statement as an extension of **Item 11 of the SPIRIT 2013 Statement** (see www.spirit‐statement.org). For alternate study designs, TIDieR can be used in conjunction with the appropriate checklist for that study design (see www.equator‐network.org).
